# Integrated framework utilizing scene text detection and recognition techniques for enhancing point of interest extraction from name boards in all Indic languages

**DOI:** 10.1038/s41598-026-40742-w

**Published:** 2026-03-10

**Authors:** Abhishek Kumar Kashyap, Mahima Upadhya, Vikas Singh Panwar, Vikrant Chandrakar

**Affiliations:** 1 Department of Mechanical Engineering, Birla Institute of Technology, Mesra, Ranchi, Jharkhand 835215 India; 2 Manipal Institute of Technology, Manipal Academy of Higher Education, Manipal, Karnataka 576104 India; 3 Department of Mechanical Engineering, Koneru Lakshmaiah Education Foundation, Vaddeswaram, 522502 India

**Keywords:** Point of interest (POI) extraction, Scene text recognition, Multilingual script identification, Deep learning-based OCR, Object detection, Engineering, Physics

## Abstract

This paper focused on enhancing text recognition, script identification, language classification, and point of interest (POI) extraction from images captured by Mobile Mapping Systems (MMS). The initiative was undertaken to improve the existing Computer vision-based artificial intelligence modules. The advancements made is to be contributed to the system’s implementation, bringing improved functionality and accuracy to the system. The current system, called Text Detection and Recognition (TDR), consists of several neural modules operating in sequential stages. The first module identifies areas of interest within the MMS images, focusing on shop signboards, traffic signs, and directional boards. The second stage involves detecting text words within these areas and cropping the relevant pixels from the image. These cropped images are then processed in the third stage, where the language script is detected, identifying one of ten Indian scripts. In the fourth stage, specific character recognizers corresponding to the identified script are used to recognize the text. The outputs from these stages are aggregated into a correlated JSON output. Additionally, a parallel fifth stage detects various fields within the MMS images, such as name, address, pin, icon, phone and GSTIN number, ultimately extracting a comprehensive human-readable address for any POI from the MMS image. The primary focus includes investigating novel text recognition algorithms to improve accuracy and efficiency, exploring various script identification algorithms to enhance language classification capabilities, implementing a dictionary-based approach for more accurate word detection, and developing methods for correcting the words that the CRNN model predicts to reduce errors. This work is novel because it combines word correction, OCR, detection, classification, and POI field extraction into a single pipeline designed specifically for Indic scripts. By obtaining 96.17% script recognition accuracy, 92.5% word accuracy, and 33% average precision in POI detection, the suggested framework outperforms previous benchmarks like IndicText (93.6%) and transformer-based OCR (88.5%).

**Supplementary Information:**

The online version contains supplementary material available at 10.1038/s41598-026-40742-w.

## Introduction

Scene text images refer to images that contain text naturally occurring in the environment, such as shop signboards, traffic signs, billboards, and other public displays. POI^1^ extraction for name board in many Indic languages is an open and challenging area of computer vision and natural language processing. With urban landscapes becoming increasingly intricate, there is a rising demand for systems that can quickly identify and read text from varied surroundings. This holds in India primarily, where many languages exist that differ in pronunciation and have different scripts, fonts, and styles. This can be a challenge, but the joint use of scene text detection and recognition methods is an exciting direction to reduce errors during name boards extraction from images, which is extremely useful for navigation, tourism, and local business identification^2^. The problem of scene text detection refers to detecting and pinpointing the location of texts in some natural images, where factors such as lighting conditions, orientation of texts, and background images are highly influential^3^. Due to these variations, traditional methods tend to fail, and consequently, deep learning techniques are being developed as much more advanced algorithms. Over the last few years, state-of-the-art uncontrolled environment text detectors have greatly improved with the introduction of convolutional neural networks (CNNs)^4^ and other machine learning architectures that greatly enhance the accuracy. In addition, the recognition stage, where extracted text is transformed into machine-readable representations, requires strong optical character recognition (OCR) technologies^5^ capable of addressing the complexities of Indic scripts. The distinctive characteristics of Indic languages make it natural to develop a scene text detection and recognition technique in an integrated framework. The integrated system uses state-of-the-art segmentation-based methods and individually trained OCR engines to achieve a seamless user experience when interacting with textual information across multiple languages. The ability to extract Points-of-Interest (POIs) from name boards in Indic languages through an integrated framework using scene text detection and recognition towards POI extraction is an essential step in the progression of urban navigation systems. That drives technological innovation and brings language diversity to the fore in fast-changing urban environments that often risk eradicating it. Guo^6^ has proposed computer vision-based artificial intelligence modules to identify defects. The project tries to enhance various components of that system.

The existing system, typically called TDR (Text Detection and Recognition), consists of various neural volumes. The first module is detecting areas of interest in the scene text. Incoming MMS images are searched for places of interest such as shop signboards, traffic signboards, Green directional boards, etc. In the second stage, text words are detected. The pixels containing the words are cropped out of MMS images. The boxes detected in the first stage are intersected with the second stage, and the corresponding cropped images are passed to the third stage. In the third stage, the language script is detected. 10 Indian scripts are classified at this stage. In the fourth stage, corresponding character recognizers are used according to the language detected. Finally, all the outputs are combined to create a correlated JSON output.

A fifth parallel stage also detects fields in the MMS Image, such as name, address, pin, icon, phone, GIS number, etc. A human consumable address is extracted from the MMS Image for any Point of Interest. Kumar et al.^7^ have proposed a detailed look at deep learning methods for recognizing Indic scripts. To tackle issues in the previous model, they introduced a new CNN-LSTM network. Nguyen et al.^8^ have offered an interesting way to enhance STR by adding a dictionary-guided setup to boost accuracy. STR can be tough due to different fonts, sizes, orientations & backgrounds. Most traditional STR methods rely on Optical Character Recognition (OCR).

Murad and Ali^9^ have presented an End-End System for Bangla Address Information Extract for detection, recognition, correction and parsing. Gunna et al.^10^ have presented a recognition of scene texts among indian languages through transferred learning. A wide variety of fonts improves the ability to recognize non-Latin scene text. Xiong et al.^11^ have proposed a method that boosts text spotting rate, which works better on complex curvy text. Rahul et al.^12^ have portrayed a technique that solves complex backgrounds, making text detection challenging. This method can be extended to translation and other languages as well. Sui et al.^13^ have proposed a framework for integrated text detection and recognition. It can be shared parameters that enhance accuracy and save computational cost. Salunkhe et al.^14^ have introduced a method for Multilingual text detection in Indian Languages for studying on ICDAR 2015 and user datasets. Dineshkumar et al.^15^ have effectively extracted scene text information that combines character descriptors and stroke configuration maps for recognition. Bixler and Miller^16^ have outlined the feasibility of extracting text from any scene mentioned in the Presentation on finding text elements and character tracking techniques. By extracting user reviews to determine preferences and emotional reactions, an emotional analysis-based point-of-interest (POI) suggestion framework has been created by Meena et al.^17^. The system improves location-based suggestions’ personalization and relevancy by incorporating feelings polarization into the suggestion pipeline. Although techniques like ResNet and YOLOv5 are commonplace, this work is novel in that it adapts, customizes, and integrates them into a modular pipeline for Indic multilingual POI extraction. As far as we are aware, no previous research has methodically integrated these elements with middleware integration and dictionary-based modification for low-resource Indic scripts.

### Research gaps, objective and motivation

In multilingual areas like India, precise language recognition is essential for image and video analysis. Character recognition is challenging in Mobile Mapping System (MMS) imagery because texts frequently appear in intricate, disorganized and low-quality situations, particularly when scripts share visual resemblance. When used in real-world Indic situations with overlaid fonts and diverse scripts, conventional Text Detection and Recognition (TDR) structures perform reasonably well (e.g., ~ 87% accuracy). Given the nature of Indic scripts, which remain absent in most popular scene text datasets and pipelines, these issues necessitate more reliable solutions.

By adding specific, adaptable elements to the TDR pipeline, the main goal of this research is to close the performance gap. The study aims to improve text recognition through post-OCR dictionary modification, optimize the pipeline for Points of Interest (POI) processing, and refine script categorization frameworks employing Indic script data. Standard approaches like ResNet and YOLOv5 are used, but the unique contribution is how they are modified, coordinated, and assessed for the Indian setting. A coherent, error-tolerant pipeline for multilingual scene text comprehension is facilitated by a post-processing modification layer that uses Levenshtein and greedy search, language-specific parameter tuning, and a carefully selected dataset of more than ten Indic scripts from MMS imagery.

### Questions for research

The following study topics serve as the basis for this work:

1. How can script recognition models be improved to differentiate visually comparable Indic scripts in poor-quality MMS images?
2. Is it possible for dictionary-driven post-OCR modification to significantly increase understanding accuracy in multilingual situations?
3. How much does integrating upgraded components (YOLOv5, FastAI, CRNN, and correction logic) into a modular TDR pipeline improve POI utilization overall?
4. In terms of precision, adaptability, and language protection, how does the improved TDR system stack up against current methods?
5. These inquiries inform the system’s design choices and efficiency assessment, guaranteeing quantifiable improvements over traditional single-script, high-resolution pipelines.

### Principal contributions

The engineering of a robust modification of current state-of-the-art frameworks with language-specific datasets, dictionary-driven enhancement, and a middleware coordination layer, rather than proposing completely new architectures, allows for the practical end-to-end POI extraction from noisy MMS imagery in multiple Indic scripts.

This study introduces a scientifically based, engineering-focused pipeline that makes significant progress in multilingual scene text recognition and POI extraction, especially for low-resource Indic scripts under challenging circumstances. Despite using well-known modules like YOLOv5 and ResNet, the framework is unique because of its customized adaptation, integration approach, and post-processing logic, none of which have been thoroughly combined and assessed for Indic MMS imagery in the literature.

The following are the primary scientific and technical contributions:

1. CNN prediction for multilingual Indic languages based on script:

A carefully selected and balanced dataset comprising more than ten Indic scripts is used to train a sophisticated script recognition component. By creating dataset enhancement techniques (such as contrast jittering, shearing, and noise simulation) to mimic natural MMS image distortions, we were able to improve script-level classification accuracy to 96.17%, which is higher than state-of-the-art benchmarks like IndicText (93.6%).

2. Dictionary-based OCR pipeline integration with a correction engine:

We proposed a post-OCR correction component that corrects CRNN outputs by using TF-IDF embeddings, greedy best-first search, and Levenshtein distance. This language-specific modification layer greatly enhanced word-level comprehension in low-resource, noisy scripts, which showed accuracy gains of up to 17% over raw CRNN projections.

3. Creation of a reliable middleware coordination layer for flexible coordination of AI:

Text identification, script categorization, OCR, and POI field extraction are examples of loosely coupled elements that rely on a middleware layer to facilitate the exchange of information, confidence-based filtering, and routing. This advances software engineering for practical implementation in modular and error-prone settings.

4. Real-world, low-resource POI field identification with an updated YOLOv5 model:

Our trained YOLOv5 outperformed previous models such as YOLOv4-BiLSTM (21%), achieving an average precision of 33% despite a challenging dataset (class imbalance, fine-grained objects, script clutter). Its outputs facilitate downstream organized inference for names, icons, phone numbers, and GSTINs.

5. Complete the POI inference pipeline with an organized output determined by JSON:

The finished pipeline provides a useful deployment enabler by producing machine-consumable, conceptually arranged address data derived from images. Under real-world occlusion, deformation, and script overlap situations not covered in previous work, the technique endorses multilingual POI recognition.

When combined, these efforts create a brand-new integrated system for Indic multilingual POI extraction that outperforms current techniques in quantifiable ways.

## Methodology

### Architecture of the TDR system

**Fig. 1:**
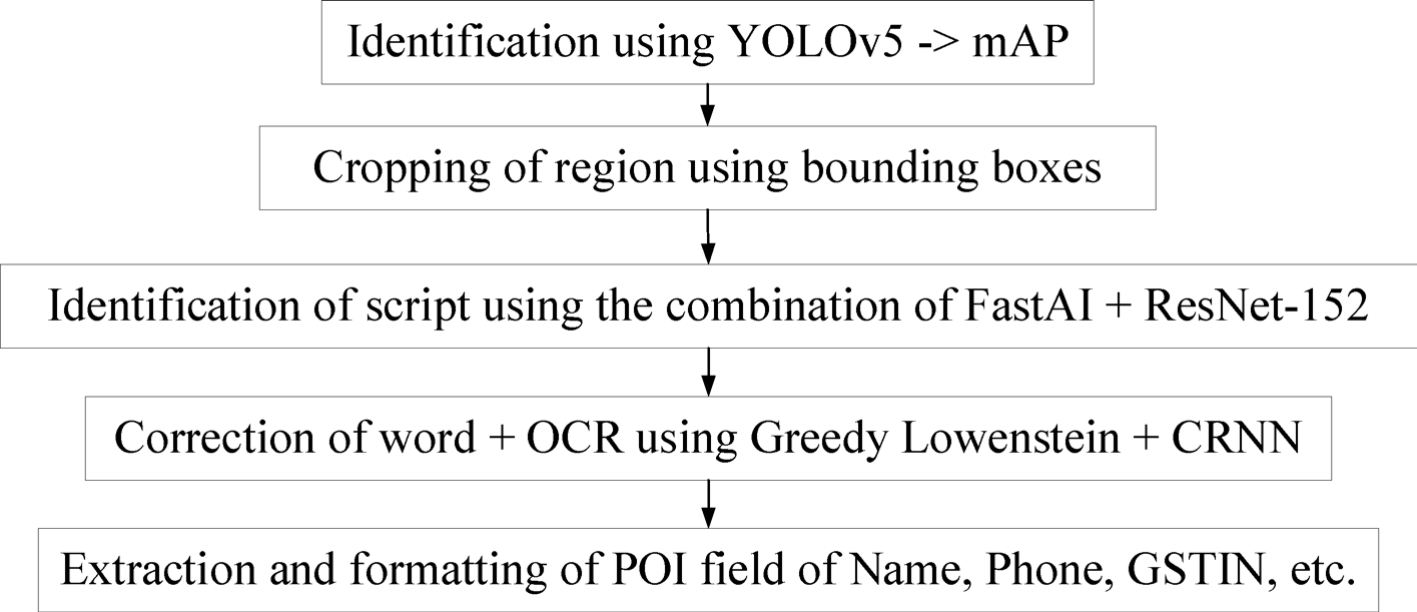
Flowchart of the process of the TDR framework.

A five-stage TDR (Text Detection and Recognition) process intended for reliable multilingual scene text comprehension and POI obtaining from an image is the foundation of the suggested system. Every step is in charge of a distinct subtask, and the adaptable design permits autonomous optimization and assessment. The improved elements, including YOLOv5 for object detection, FastAI-based ResNet for text categorization, and a dictionary-driven modification component, are mapped to the corresponding pipeline stages in the flowchart of the improved TDR system shown in Fig. 1. In particular, Stage 1 uses YOLOv5 to identify potential POI regions, Stage 2 consequently crops text portions from bounding boxes, and Stage 3 uses refined ResNet variants to classify the language. Stage 4 uses a CRNN to perform OCR and applies post-processing modifications determined by greedy search and Levenshtein distance. Lastly, organized POI fields (such as name, phone number, and GSTIN) are extracted in Stage 5 and output in a JSON format. As explained in the following results section, this flowchart guarantees that the component-to-stage visualization is clear and discusses how enhancements in each module affect the overall effectiveness.

### Language classification

The project employs a combination of machine learning, deep learning, and artificial intelligence techniques. We employed the Fastai CNN-LSTM methodology, CRNN, Attention network, computer vision, and natural language processing techniques. Training datasets comprising diverse textual samples in various languages are utilized to train and fine-tune the algorithms. The methodology involves iterative algorithm development, testing, and refinement to achieve the stated objectives. Fastai architecture is a popular library used in machine learning, particularly in deep learning tasks. It is built with PyTorch, which provides a high-level API that can simplify the process of building, training, and deploying deep learning models.

#### Classification of dataset based on script

We selected a dataset of designated word images from ten Indic scripts and English to train and assess our script categorization component. To guarantee script balance, these instances were taken from actual Mobile Mapping System (MMS) imagery and enhanced with open-source datasets and artificial augmentation. Table 1 presents the distribution of datasets based on language (Table 2). A total of 45,050 training, 9000 validation, and 9000 test word-level image crops made up the dataset utilized for training the ResNet-based script classification representations. Bounding boxes with text region annotations were used to extract most samples from real-world Mobile Mapping System (MMS) imagery. We added samples from publicly accessible, script-tagged datasets like CVSI (Classification of Video Script Images) and IndicSceneText (limited usage) to this data to guarantee script diversity and balance. In order to simulate natural appearance using script-specific styling, we created extra synthetic data using Google Fonts for scripts with few real-world samples.

**Table 1:** Distribution of dataset based on language.

Language	Test samples	Validation samples	Training samples
Bengali	900	900	4500
English	900	900	4800
Kannada	900	900	4300
Odia	900	900	4100
Tamil	900	900	4700
Devnagari	900	900	5300
Gujarati	900	900	4100
Malayalam	900	900	4100
Punjabi	900	900	4300
Telugu	900	900	4850

**Table 2:** Accuracy and, correct and incorrect count of words of each ResNet 50 model.

For Batch_size = 90, epoch = 20, lr = 0.0014454, Resize = 170, GPU = 6403MiB (ResNet50)					
Language	Accuracy	Total count	Correct count	Incorrect count	Architecture
Bengali	93.2	2088	1946	142	ResNet50 and fastai
English	94.35	5715	5392	323	ResNet50 and fastai
Kannada	88.95	1131	1006	125	ResNet50 and fastai
Odia	95.71	2306	2207	99	ResNet50 and fastai
Tamil	90.46	1751	1584	167	ResNet50 and fastai
Devnagari	95.34	3931	3748	183	ResNet50 and fastai
Gujarati	94.54	3040	2874	166	ResNet50 and fastai
Malayalam	94.94	4108	3900	208	ResNet50 and fastai
Punjabi	97.32	2609	2539	70	ResNet50 and fastai
Telugu	96.02	4753	4564	189	ResNet50 and fastai
Average	94.083				

To comply with ResNet input specifications, all images were preprocessed by resizing to 200 × 200 pixels. Various augmentation methods were used during training to enhance abstraction and replicate actual noise situations. These comprised Gaussian noise addition, similar shearing (± 10%), horizontal stretching (up to ± 15%) to mimic viewpoint deformation, brightness and contrast jitter, and arbitrary rotation (± 10°). The average and standard deviations calculated across the training set were used to normalize the dataset. The robustness of the model was greatly increased by these preprocessing steps, especially when dealing with visually complicated or low-quality script samples, like those in Odia and Malayalam.

#### Training process

The methodology involves training an image classification model using the Fastai library. The chosen approach leverages a convolutional neural network (CNN) with transfer learning, data augmentation, and early stopping to achieve optimal performance.

First, we import the necessary libraries from Fastai, a high-level API built on PyTorch, simplifying the training process of neural networks. Then, the dataset path is specified, where the dataset is organized into subdirectories, where each subdirectory represents a class. The ‘DataBlock’ is configured in such a way as to handle image classification. The data is split into training and validation sets. The batch size, the number of images processed together in one iteration, is set to 90. This value is chosen based on available GPU memory and the dataset size. DataLoaders are created from the DataBlock, which handles loading the data in batches, including applying data augmentation, normalization, and resizing. A convolutional neural network (CNN) learner object is created using a pretrained ResNet-50, ResNet-101, or ResNet-152 model. A graph is used to plot training and validation losses. The learning rate is chosen. The model and its data are converted to half-precision to reduce memory usage and speed up computation. Early stop is configured to stop the training early if the validation loss does not improve by at least 0.01 for 3 consecutive epochs, to prevent overfitting and save training time. The model is fine-tuned for n epochs with a base learning rate. Finally, the trained model is exported to a specified location (Table 3).

**Table 3:** Accuracy and, correct and incorrect count of words of each ResNet 152 model.

For Batch_size = 30, epoch = 15, lr = 0.002089, Resize = 200, GPU = 7627MiB (ResNet152)					
Language	Accuracy	Total count	Correct count	Incorrect count	Architecture
Bengali	93.01	2088	1942	146	ResNet152 and fastai
English	94.94	5715	5426	289	ResNet152 and fastai
Kannada	88.86	1131	1005	126	ResNet152 and fastai
Odia	95.36	2306	2199	107	ResNet152 and fastai
Tamil	90.01	1751	1576	175	ResNet152 and fastai
Devnagari	95.88	3931	3769	162	ResNet152 and fastai
Gujarati	95.23	3040	2895	145	ResNet152 and fastai
Malayalam	95.03	4108	3904	204	ResNet152 and fastai
Punjabi	96.59	2609	2520	89	ResNet152 and fastai
Telugu	96.04	4753	4565	188	ResNet152 and fastai
Average	94.095				

#### Evaluating a trained image classification model

It involves loading the trained image classification model, evaluating its performance on a test dataset, and then computing the model’s accuracy. Once the trained model is loaded, the path to the test dataset’s dictionary is defined. A test DataLoader is created using the test images. The predicted probabilities and accurate labels are printed to inspect the results. The model’s accuracy on the test dataset is calculated by comparing the predicted labels with the proper labels and computing the mean of correct predictions. This methodology ensures that the model’s performance is assessed on previously unseen data, accurately measuring its generalization capabilities.

Using pre-trained ResNet structures optimized on our Indic language dataset, we performed script-wise performance evaluation to assess the efficacy of our language categorization component. Word-level precision for classification was used to evaluate the models, and each language was considered a separate class. Test sets were in equilibrium to guarantee a comparable proportion across all ten Indic scripts. ResNet-152 performed better than ResNet-50 and ResNet-101 among the evaluated architectures in every language. Its deeper convolutional layers, which can better capture the subtle curvilinear characteristics found in complicated scripts like Telugu, Odia, and Malayalam, are responsible for this.

Additionally, we found that increasing the input image resize from 170 to 200px for stroke-dense scripts resulted in appreciable accuracy gains (1.2–1.8%). Still, batch sizes had to be decreased appropriately to prevent GPU memory overflow. Overfitting was avoided by using early stopping (with a threshold delta of 0.01 over three consecutive epochs), particularly in low-resource languages like Tamil and Kannada. Using ResNet-152, the average classification precision for all scripts was 94.1%; Punjabi and Devanagari showed the best results, at 97.3% and 95.9%, respectively.

Methodological Perspectives:

- The combination of Indic scripts is more appropriate for deeper networks, such as ResNet-152.
- For dense characters, characteristic resolution is enhanced by larger image sizes (200px).
- Early stopping based on verification is essential for script training with limited assets.

#### Evaluating the accuracy of the model on each language dataset

It is the process of loading a trained image classification model, preparing a specific test dataset, making predictions, and evaluating the model’s performance using Fastai. This method ensures that the model’s accuracy is assessed on a subset of the dataset, i.e., data of each language, thus providing insights into its performance and the number of correct and incorrect predictions (Table 4).

**Table 4:** Accuracy and correct and incorrect counts of words of each ResNet 101 model.

For Batch_size = 64, epoch = 10, lr = 0.0008317, Resize = 190, GPU = 5025MiB (ResNet101)					
Language	Accuracy	Total count	Correct count	Incorrect count	Architecture
Bengali	94.49	2088	1973	115	ResNet101 and fastai
English	95.61	5715	5464	251	ResNet101 and fastai
Kannada	90.98	1131	1029	102	ResNet101 and fastai
Odia	95.4	2306	2200	106	ResNet101 and fastai
Tamil	92.29	1751	1616	135	ResNet101 and fastai
Devnagari	96.49	3931	3793	138	ResNet101 and fastai
Gujarati	95	3040	2888	152	ResNet101 and fastai
Malayalam	95.59	4108	3927	181	ResNet101 and fastai
Punjabi	98.01	2609	2557	52	ResNet101 and fastai
Telugu	96.44	4753	4584	169	ResNet101 and fastai
Average	95.03				

### Object detection using YOLOv5

YOLOv5 is a convolutional neural network architecture used for object detection. It is an evolution of the YOLO (You only live once) series of object detection models.

#### Dataset preparation

**Fig. 2:**
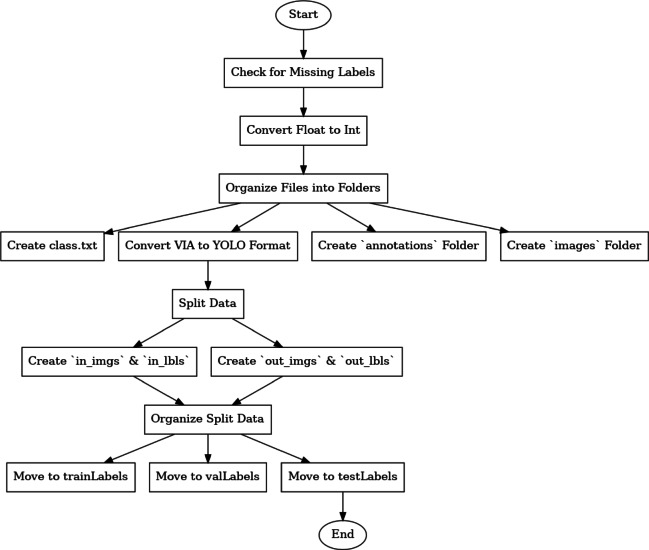
Block diagram of the preparation of Dataset.

Utilizing an application to verify for missing tags in the annotations is the first step in efficiently preprocessing JSON-annotated documents. This involves checking whether any labels are completely missing or if important characteristics like width, height, x, and y are omitted. For uniformity and reliability, tidy up the data by converting all floating-point variables to integers. Make a folder called annotations and move all JSON annotation documents into it to arrange your workspace. Similarly, make a folder called pictures and transfer all related image files. To guarantee a precise category mapping, create a class.txt file with all the class names listed line by line. The annotation files are to be converted from the VIA (VGG Image Annotator) form to the YOLO design, which is often utilized for item identification tasks, to prepare them for training. Make specific folders called in_imgs and in_lbls for the input photos and labels, and out_imgs and out_lbls for the split information to divide the data. Once the data has been divided into training, validation, and test sets, place the corresponding .txt label files and .jpg files into the trainLabels, valLabels, and testLabels folders. This guarantees an organized dataset for the machine learning procedures that follow. The block diagram for the process is presented in Fig. 2.

#### Training of object detection model

**Fig. 3:**
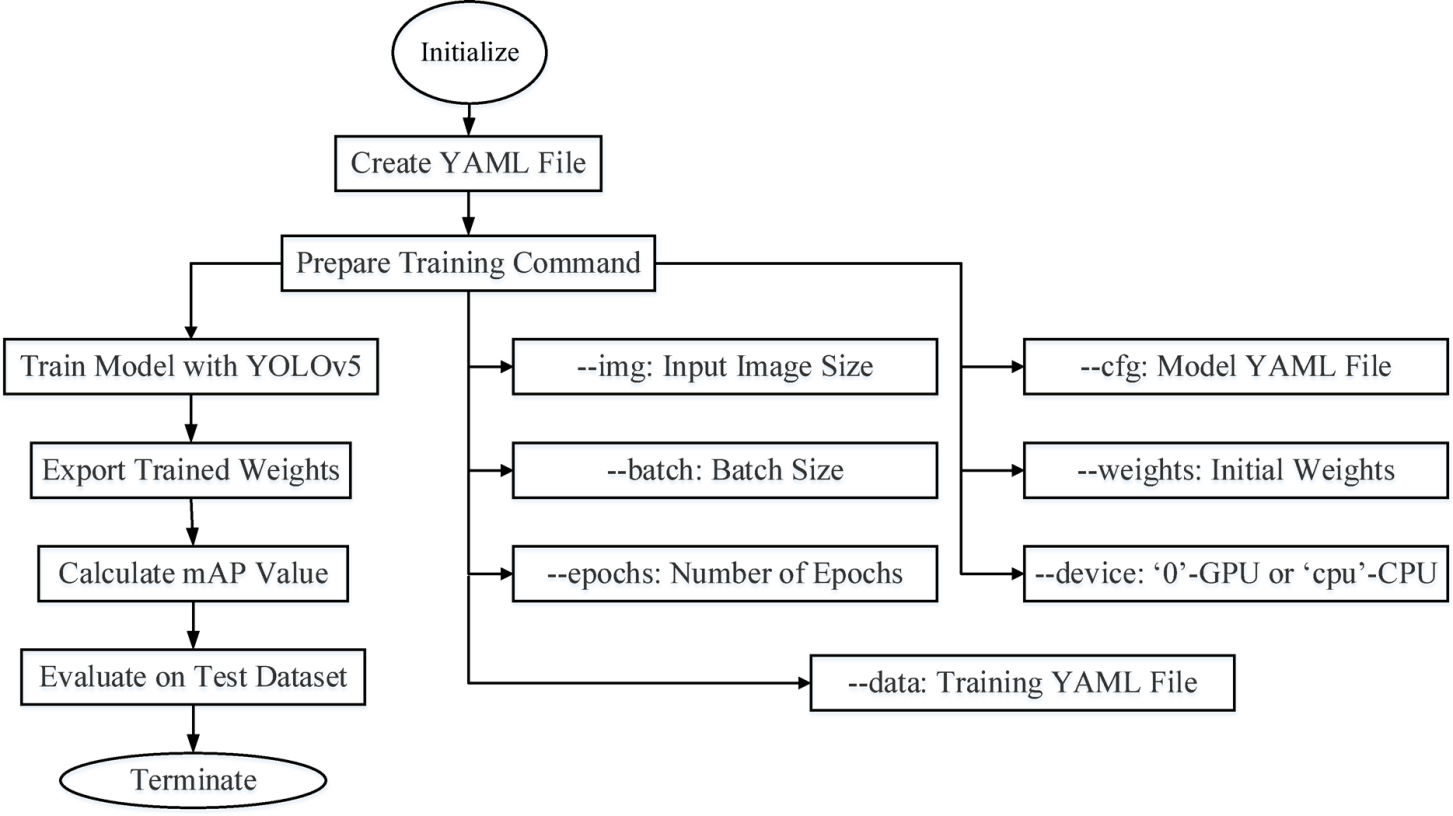
Block diagram of the training process.

The process starts with creating a YAML file containing the dataset’s required settings in order to train a network using the YOLOv5 framework. The training control that makes use of the train.py script is then prepared. A few variables are included in the command: --data indicates to the YAML file generated for training, --cfg corresponds to the model setup YAML file, --weights specifies the initial weights file for instruction, --device decides if the training operates on a GPU (‘0’) or CPU (‘cpu’), and --img defines the input image size, --batch defines the batch size, and --epochs demonstrates the quantity of epochs. The YOLOv5 architecture trains the system once the training instruction is run. The taught weights are stored locally for later usage following the training procedure. The model’s accuracy is measured by computing the mean Average Precision (mAP) number, which is used to assess the model’s efficiency. The model’s performance is evaluated using a test dataset that includes unseen data to guarantee generalization and efficacy. The evaluation findings, which offer insights into the model’s predictive power, mark the procedure’s end (Presented in Fig. 3).

Methodological Perspectives:

- The two main restrictions on POI field detection are class imbalance and small text size.
- Batch adjusting and ideal image size can aid in striking a balance between memory and small-object solution.
- Stable training required manual label adjustment and annotation modification.

#### About the dataset

The real-world imagery collected by Mobile Mapping Systems (MMS) spread throughout several Indian cities served as the basis for the dataset used to train and assess the POI field identification and language classification algorithms. Numerous store signboards and public signs with text in different Indian languages can be seen in these pictures. The dataset comprises 8100 MMS-captured photos with annotations for POI identification and approximately 20,000 cropped phrases and words utilized for script categorization and OCR correction.

Five different classes, each of which represents a particular kind of item to be recognized, make up the dataset used to train the YOLOv5 model:

- Poi_Name: This class is used to identify a Point of Interest (POI), such as a symbol or company name.
- Poi_Phone: Allows contact information to be retrieved from visual data by representing phone numbers linked to POIs.
- Poi_Pin: The postal identity number (PIN) or zip code associated with a POI is important for geographical translation.
- Poi_Icon: Captured logos or visual icons connected to POIs, such as marks or brand emblems.
- Poi_GSTIN: This stands for a POI’s Goods and Services Tax Identification Number (GSTIN), which is frequently used for regulatory or monetary reasons.

These classes support activities like automatic POI identification and document evaluation, which extract organized and detailed knowledge from optical datasets. The dataset contains text in English and ten major Indic scripts. Tamil, Telugu, Bengali, Kannada, Gujarati, Malayalam, Punjabi (Gurmukhi), Hindi (Devanagari), Odia, and English. At least 4,000 labeled words are used for each language, guaranteeing a fair assessment of script classifiers. To maintain fairness, minority scripts such as Odia and Malayalam were independently oversampled employing artificial enhancement.

### Establishing the dictionary from the given .json annotation files

**Fig. 4:**
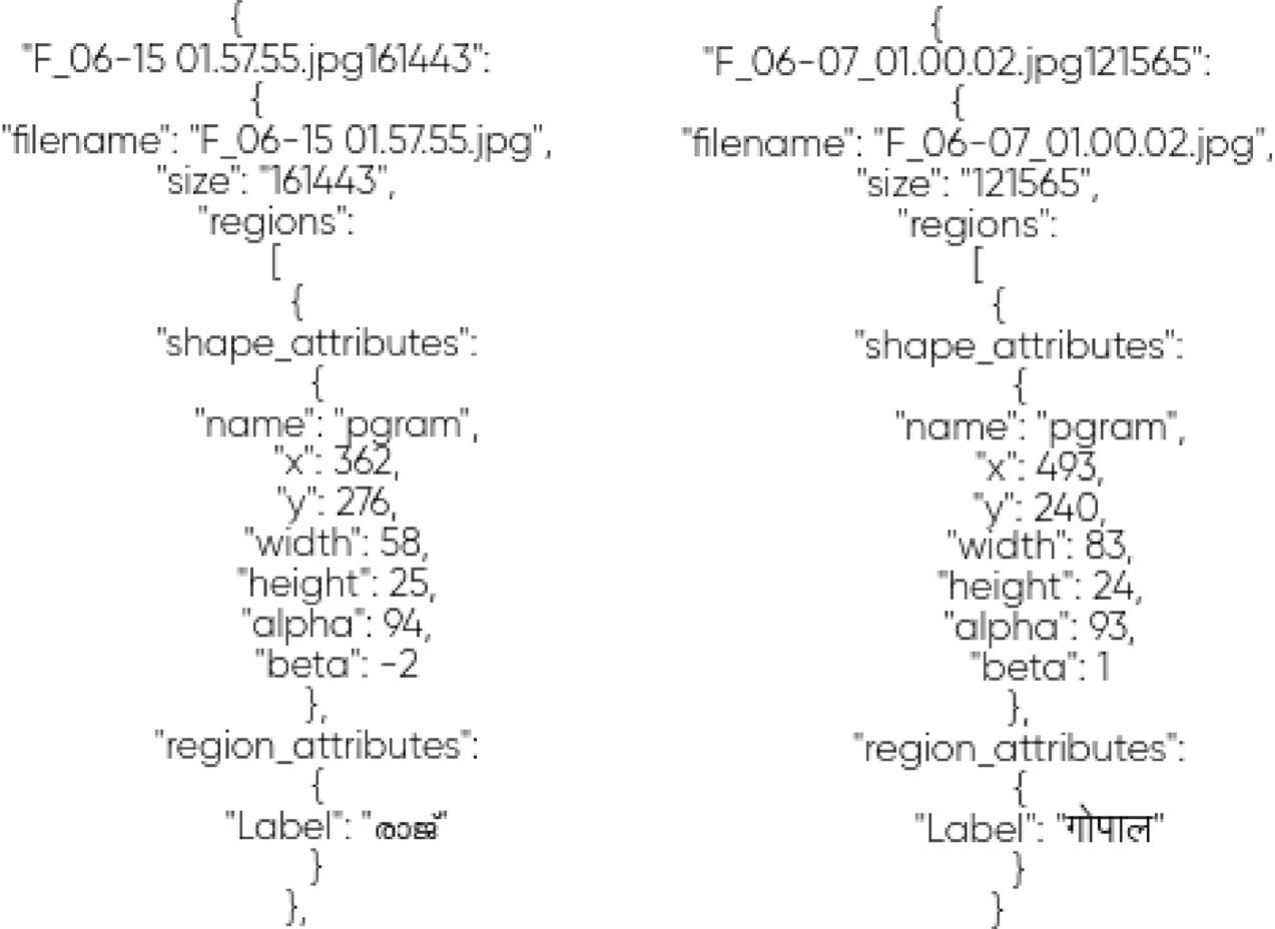
The structure of the annotation file from which the label is being extracted.

JSON-formatted tags for an item identification job are visible in Fig. 4. With information like the name, size, and locations of interest, each entry relates to a picture file. Geometric features such as location (x, y), dimensions (width, height), along with additional shape-related factors (alpha, beta), are specified by the shape_attributes inside the regions.  (in the local script) is an example of the tag field in the region_attributes that indicates the particular class or text linked to the annotated location. These annotations are probably a dataset component used to train an algorithm, like YOLOv5, for object or word identification tasks.

#### Establishing the dictionary criteria

Integrating methods were commonly used in the TDR pipeline’s dictionary-based modification and POI conceptual verification phases. In particular, character-level TF-IDF vectoring and edit-distance-derived resemblance statistics were used to embed each dictionary text and OCR output text into a space of vectors. These insertions were created dynamically from the task-specific vocabulary selected from the annotation JSON files; they were not pre-trained. In a greedy best-first search, candidate corrections that were semantically relevant and syntactically close (as measured by Levenshtein distance) were found using embeddings. To guarantee that field mapping during the POI field extraction stage, simple dense embeddings of obtained terms were contrasted to label-specific model vectors using cosine correspondence (e.g., differentiating between phone numbers and PINs when displaying is unclear). In noisy situations where visual resemblance (based on OCR outputs) was insufficient to identify the best textual correspondence, these insertions were crucial in ranking candidate modifications.

Developing dictionaries tailored to specific languages identified within the captured images to facilitate accurate text recognition, which can be used to identify points of interest.

- Purpose: It clearly defines the purpose of the dictionary criteria. Explain how it aims to enhance text recognition within images to identify points of interest more accurately.
- Describe the methodology used to develop the dictionaries, emphasizing the utilization of StyleGAN implementations.

Explored methods to compare the words to correct those that were mispredicted. The methods include cosine similarity, edit distance, Levenshtein distance, NLP pipelines, tokenization, text vectorization, beam search, count vectorization algorithms, transpositions, and pose matching. Each method was studied thoroughly, and the research concluded to implement beam search, Levenshtein distance, and greedy best first search methods, then evaluate each method to determine which would best fit the specific case. When given an input word, the model should compare the word with the dictionary and skip adding the word to the dictionary if the word is alphanumeric, already present in the dictionary, and has numeric characters. The text attributes were extracted from the given .json annotation files, and the languages were identified. Once the language of each word is recognized, it is saved into the appropriate dictionary file, creating a comprehensive dictionary for 9 Indic languages and 1 English language. The created dictionary is further used to correct the incorrectly predicted word by the CRNN. (The predictions by the CRNN model referred to here are those made earlier in the company.) Comparison of the three algorithms was done, and each algorithm’s confidence score was calculated to finalize the algorithm. Initially, a few words from each language have been collected to see each word’s confidence score and then calculate each language’s accuracy. The confidence score for each language is mentioned in Tables 5, 6, 7, 8, 9, 10, 11, 12, 13 and 14.

**Table 5:**
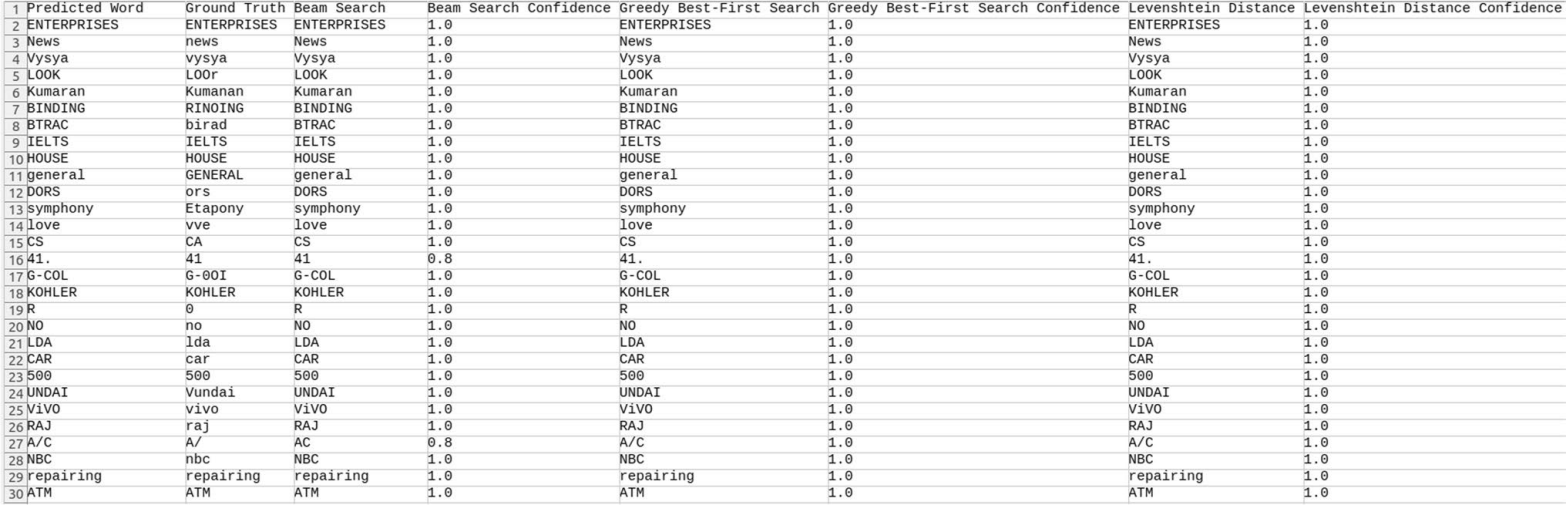
Confidence score for English Language.

**Table 6:**
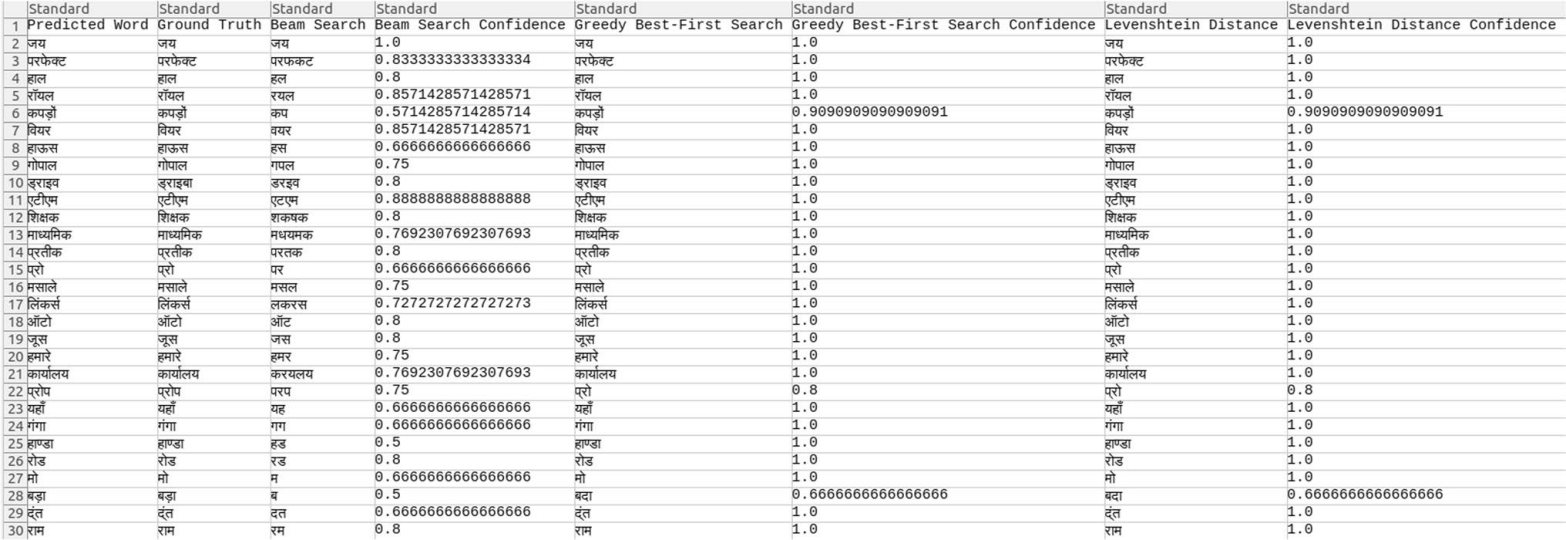
Confidence score for Hindi language.

**Table 7:**
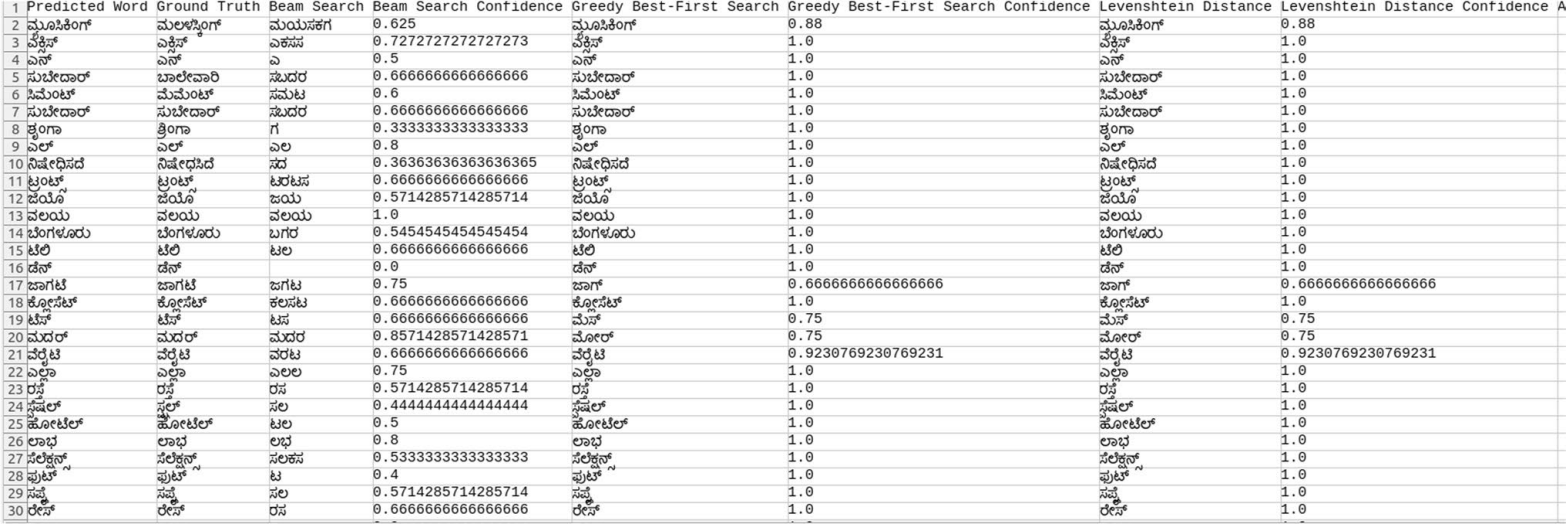
Confidence score for Kannada language.

**Table 8:**
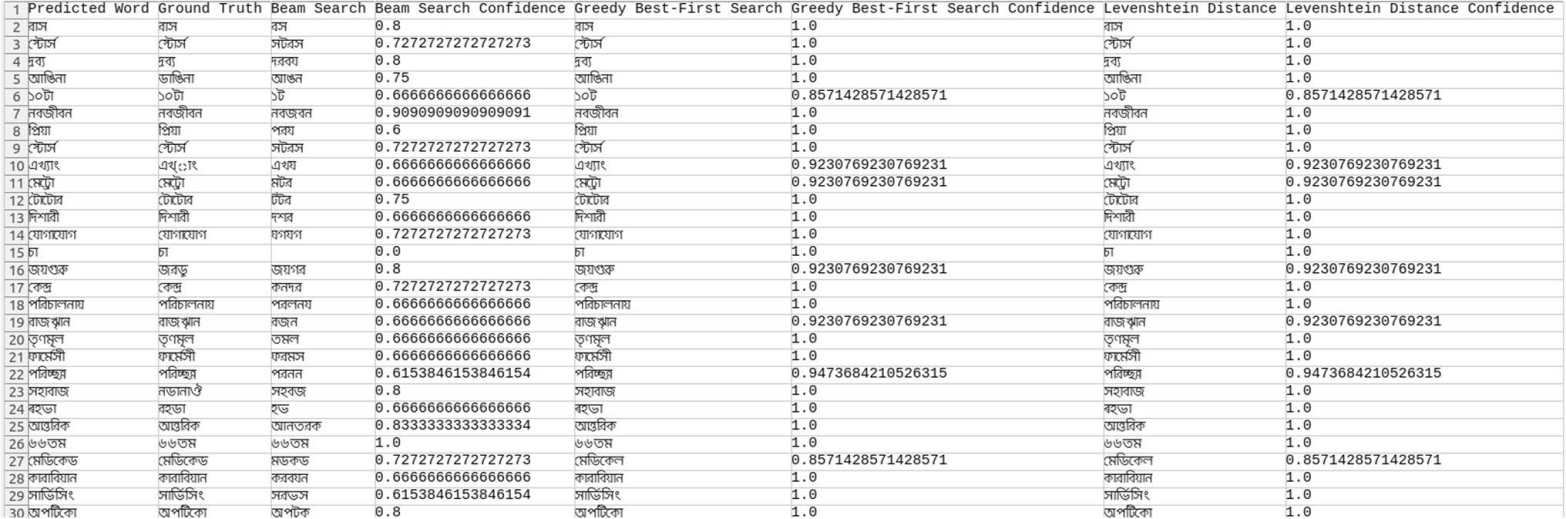
Confidence score for Bengali language.

**Table 9:**
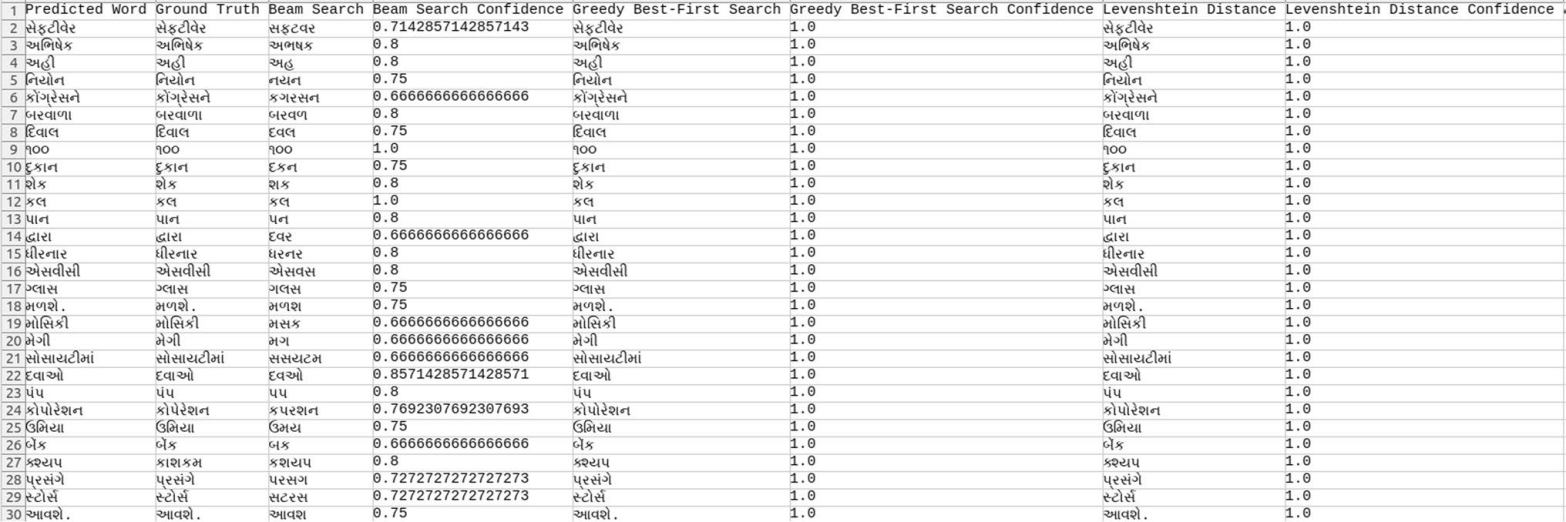
Confidence score for Gujarati language.

**Table 10:**
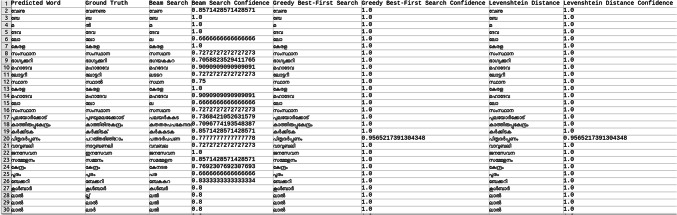
Confidence score for Malayalam language.

**Table 11:**
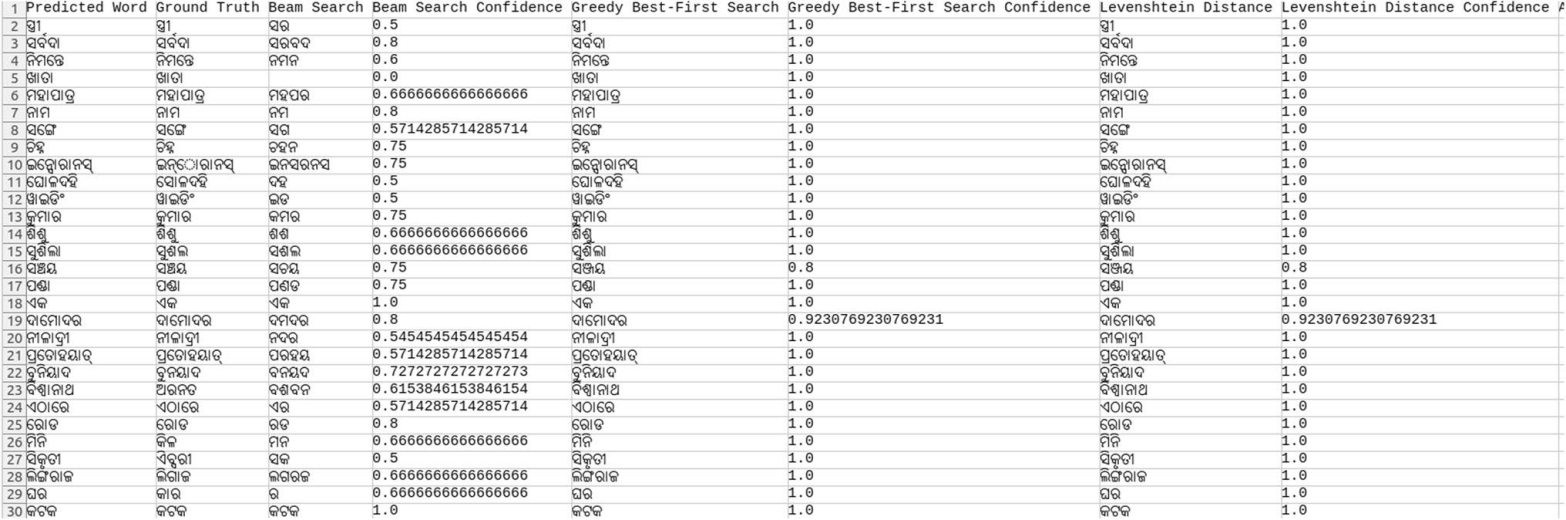
Confidence score for Odia language.

**Table 12:**
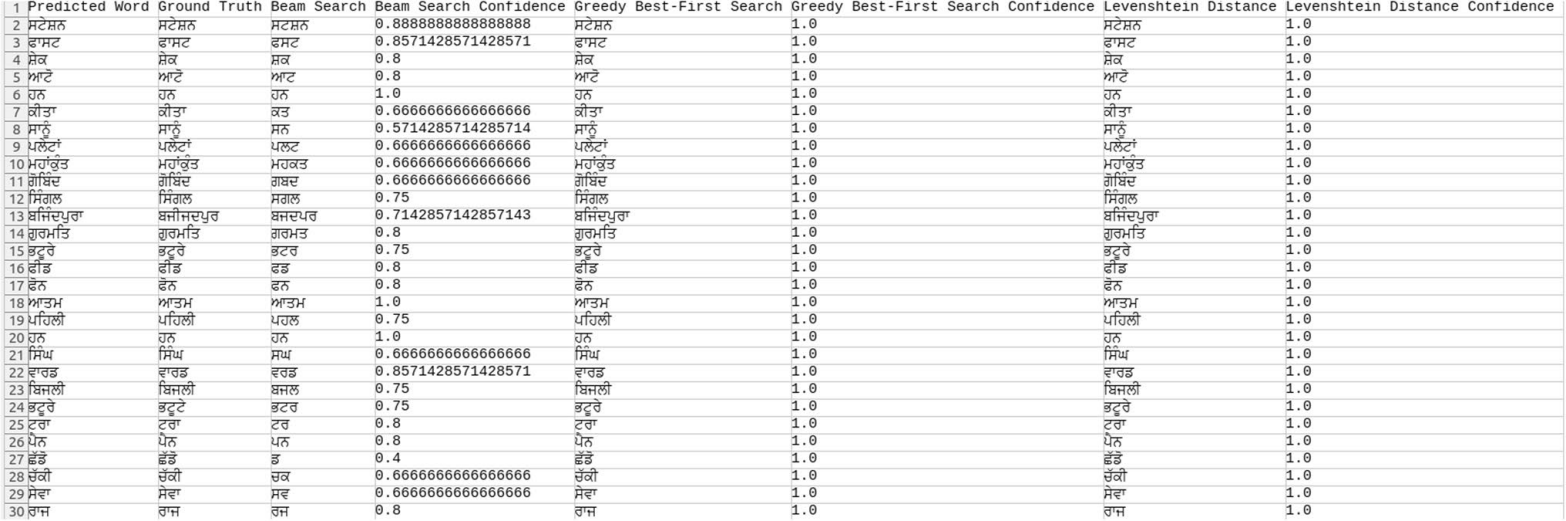
Confidence score for Punjabi language.

**Table 13:**
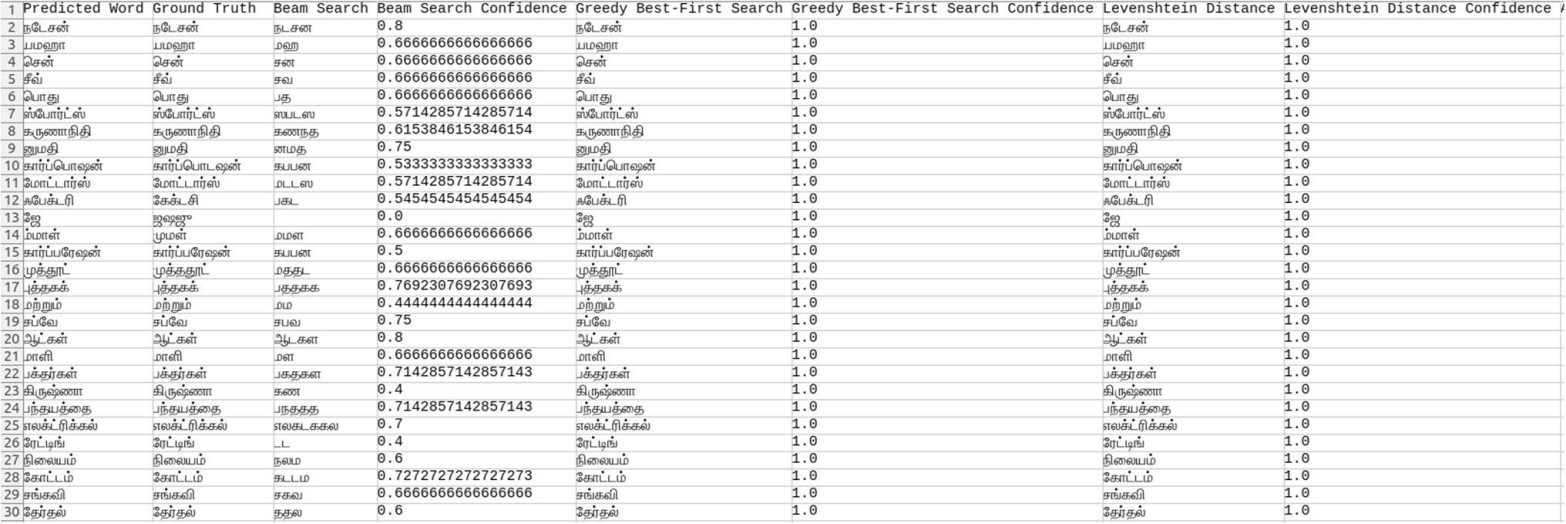
Confidence score for Tamil language.

**Table 14:**
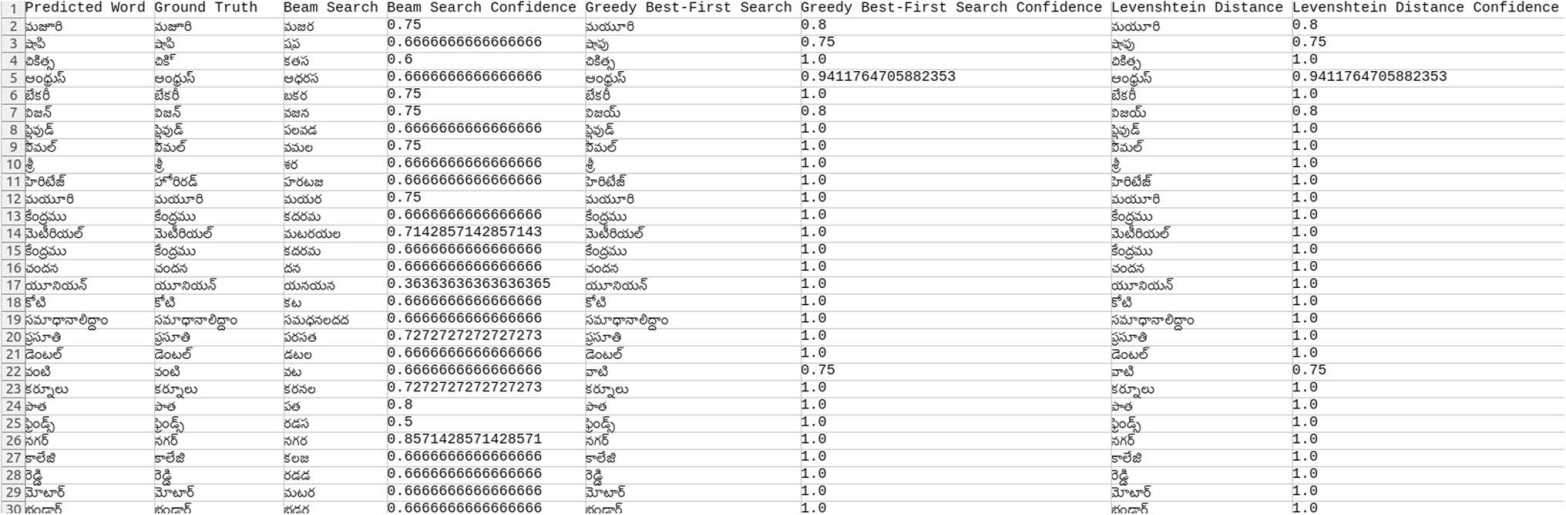
Confidence score for Telugu language.

Tables 5, 6, 7, 8, 9, 10, 11, 12, 13 and 14 present a comparative analysis of OCR predictions by different components of the proposed TDR pipeline. Each row corresponds to a unique image instance, identified by its image name, and is evaluated against multiple models and configurations. It highlights cases where initial model predictions were incorrect, but later stages led to improved results when beam search, greedy search, and dictionary methods were used. Confidence scores describe how confident the particular method is in correcting the word compared to the ground truth. Levenshtein distance acts as a quantitative metric for evaluating improvements across stages. The findings show that Greedy Best-First Search consistently surpasses Beam Search in all examined languages, frequently by a significant margin, when using Levenshtein distance as its evaluation operation. The greedy approach is more effective for languages with high visual difficulty, like Gujarati (89%), Punjabi (92%), and Tamil (90%). However, because Beam Search relies on edit-distance heuristics without specific comprehension, its accuracy stayed below 10% for most Indic scripts. English performs the same (36%) in all three approaches, most likely due to its smaller sample size and lower ambiguity. These results validate that editing-distance-driven greedy search better serves the enhancement of noisy scene-text OCR in multilingual situations.

#### Comparison of the three algorithm namely: Levenshtein distance, Beam search and Greedy best first search

This section contrasts three methods for enhancing OCR output by comparing the predicted word with a dictionary tailored to the target language. The Greedy Best-First Search approach chooses the dictionary word with the smallest Levenshtein distance from the predicted word after evaluating all the words. It is computationally effective and performs well in languages with slight vocabulary variance and short words. By keeping a fixed-size queue of the top k most likely candidates, Beam Search investigates several correction candidates concurrently. However, in our current application, the search favored low-distance but conceptually incorrect matches due to the lack of a semantic language model. As a result, it did not perform well on noisy data. Levenshtein distance scoring was done directly in specific baseline comparisons without using a structured search. Distance-based modification without a search policy acts as a lower-bound reliability.

The accuracies of the three algorithms are mentioned in Table 15.

**Table 15:** Accuracy of the three algorithms.

Language	Accuracy		
	Beam search	Greedy search	Levenshtein distance
Bengali	7%	65%	65%
Gujarati	4%	89%	89%
Kannada	1%	70%	70%
Odia	7%	57%	57%
Tamil	1%	90%	90%
English	36%	36%	36%
Hindi	8%	84%	84%
Malayali	5%	43%	43%
Punjabi	8%	92%	92%
Telugu	1%	79%	79%

From the table, it can be inferred that Greedy Search, which uses Levenshtein distance as a crucial variable, is more accurate than Beam Search in terms of precision. The Levenshtein distance between each dictionary word and the anticipated word is determined using the greedy_best_first_search operation, which then chooses the word with the least distance. Real incorrect predictions from a pre-trained CRNN simulation were utilized to test a set obtained from annotated .json documents to create the dataset for assessing correction techniques. Each predicted word had its true declaration available, allowing for a quantitative accuracy assessment. Levenshtein determines the minimum edit distance across all candidates, whereas Greedy Search uses a designed search tree traversal to minimize difficulty, even though both methods rely on the modified distance. Due to the lack of a language model, Beam Search performed less well in our tests, frequently producing semantically incorrect outputs even at short edit distances. Lexicon-aware or neural-guided beam search techniques will be investigated in future research. The word modification accuracy attained for all methods across ten Indic and English scripts is compiled in Table 15. With enhancement margins varying from 9 to 17% based on the script and noise level, Greedy Best-First Search consistently surpassed Beam Search when employing Levenshtein distance as a scoring standard.

Levenshtein distance is the evaluation component of both the Greedy and Beam Search approaches, but it is not a search method.

### Incorporation of bridge code and pipeline sturdiness

A specialized middleware layer, the bridge code, is incorporated into the system building to guarantee consistent performance across highly modular elements like text detection, localization, script recognition, OCR, and data extraction. By serving as a coordination tool, this bridge ensures that data flows smoothly, errors are handled, and formats are consistent across modules.

The bridge code uses the following development fundamentals to keep the system robust and adaptable:

#### Standardizing data formats and interactions

**Fig. 5:**
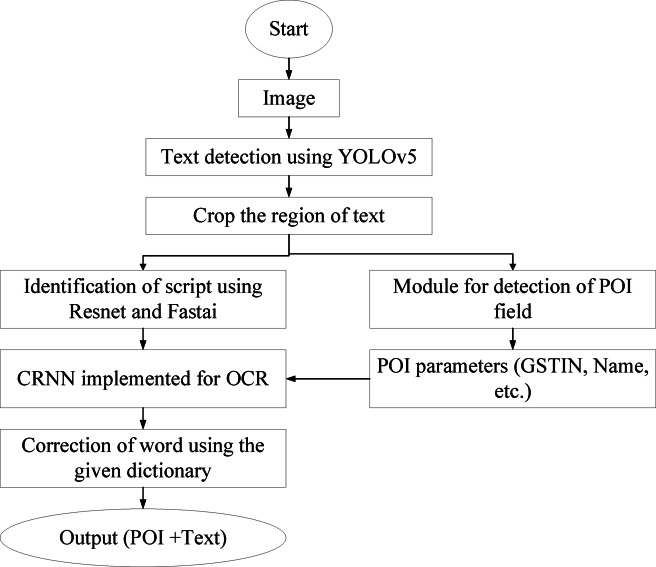
Procedure of detection of text and POI elements (Name, Number, GSTIN, etc.)

Structured between representations, such as bounding boxes, cropped image regions, or annotated JSON, are used by each module for communication. By enabling consistent interaction between independently created components, these standardized formats lower the possibility of misunderstandings or the spread of failures. The bridge is presented in Fig. 5. Every module is guaranteed to receive clean, pre-validated components owing to the bridge, and its output is examined before being issued.

#### Confidence sorting and setting

Every component produces a confidence score (such as detection confidence or OCR likelihood). The bridge assesses these scores, which then filters outputs with low confidence. This lessens the effect of inadequate component efficiency and provides information for downstream elements’ fallback or modification logic.

#### Redundancy and error identification

The bridge has the following features to avoid a single weak module causing the entire system to fail:

- Procedures for handling errors (such as flagging or skipping invalid outputs)
- Especially during the OCR and word correction phases, redundant correction techniques like Levenshtein distance, greedy search, and dictionary matching

At each step, thorough logs are kept for assessment of performance and upgrading in the future. Without interfering with the architecture as a whole, these logs aid in improving specific modules.

## Results and visualization

### Language classification/script identification

#### ResNet-50 for script recognition and language classification

For applications involving language categorization or script verification, the ResNet-50 model obtains a precision of 94.38%. It underwent training with a training rate of 0.002, a batch size of 90, and 10 epochs. To comply with the model’s input specifications, the input photos were shrunk to 170 pixels. 6403 MiB of GPU memory was used during the training process, proving that it is effective at managing big batch sizes without sacrificing speed.

ResNet-50 is appropriate for computing resources with mild constraints because of its comparatively small design (Table 16).

**Table 16:**
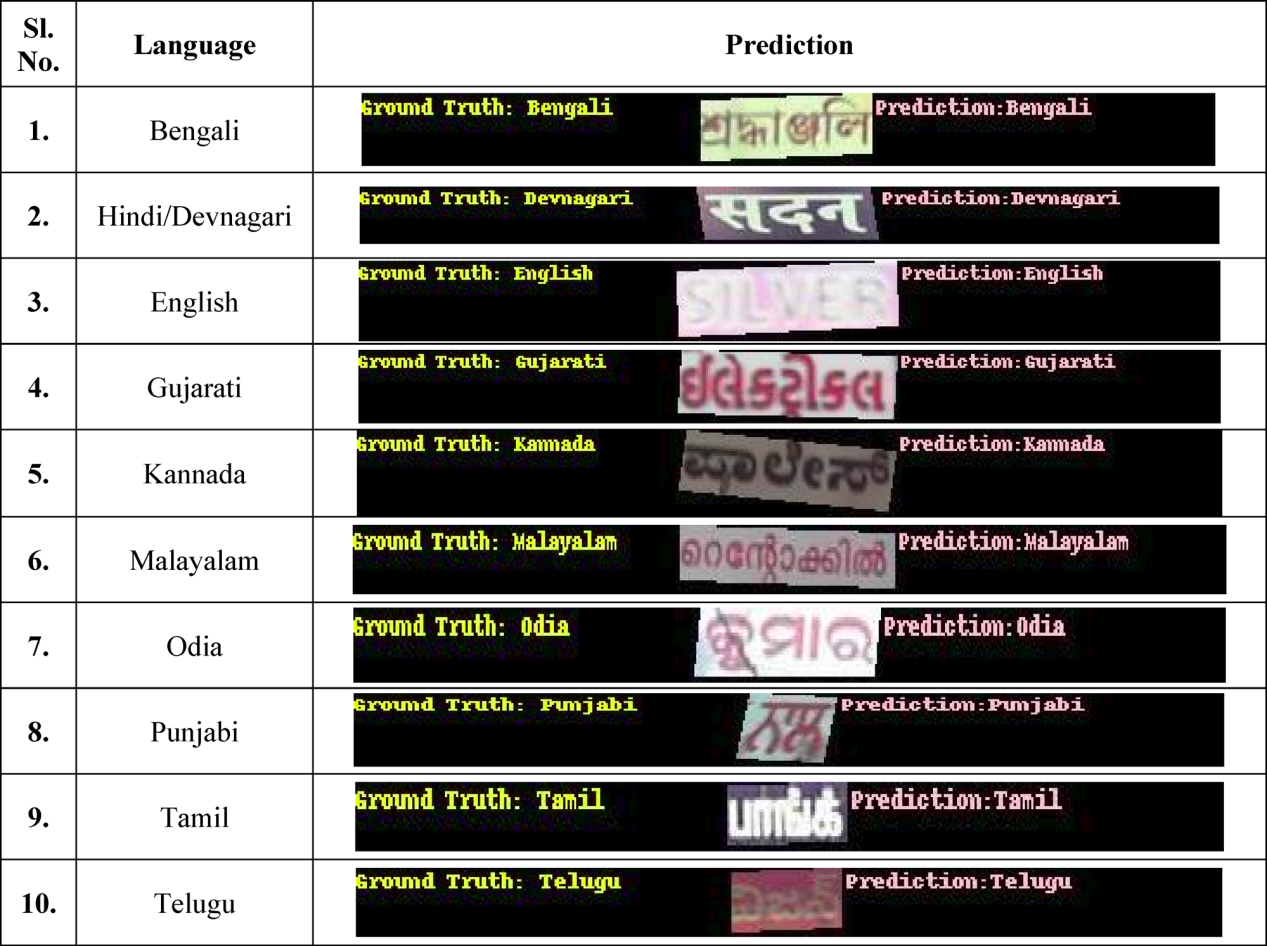
Visualizations of the ground truth and predictions of the 10 languages using ResNet-50

#### ResNet-101 for script recognition and language classification

With an accuracy of 95.54%, the ResNet-101 model outperforms the ResNet-50 strategy. It was developed over 10 epochs with a learning rate 0.0008 and a batch size of 64. To help the model acquire finer characteristics, the input photos were enlarged to 190 pixels, which is somewhat bigger than those used in ResNet-50. This model used 5025 MiB of GPU memory, demonstrating how its deeper design allowed it to achieve lower memory consumption and higher accuracy than ResNet-50.

#### ResNet-152 for script recognition and language classification

The ResNet-152 model provides the greatest precision at 96.17% of the three ResNet variations for this assignment. It was developed over 15 epochs with a learning rate of 0.002 and a batch size of 30. To enable the algorithm to acquire even more comprehensive data, input photos were reduced to 200 pixels. Because of its deeper network’s increased computational demands, the model used 7627 MiB of GPU RAM (Table 17).

**Table 17:**
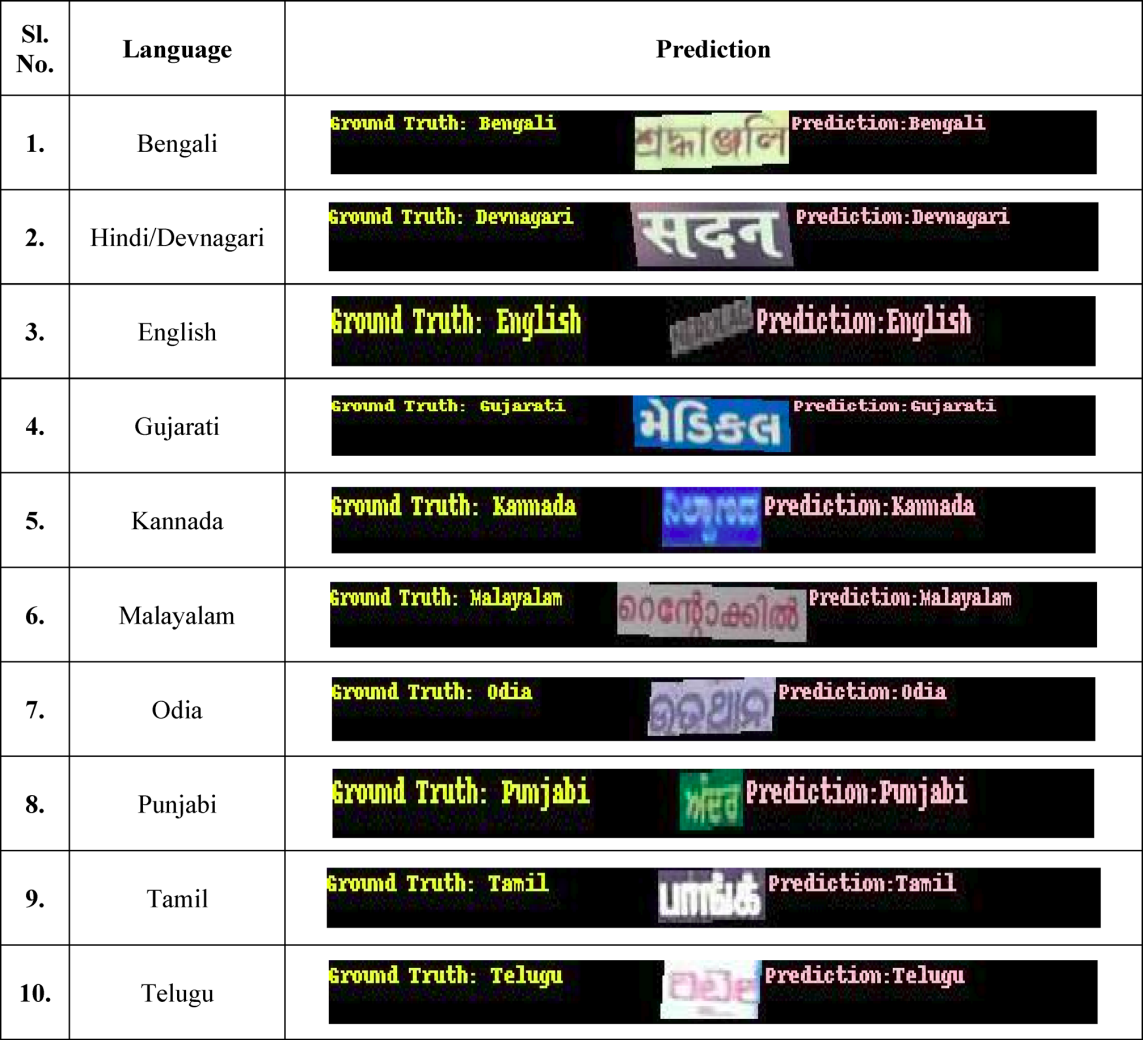
Visualizations of the ground truth and predictions of the 10 languages using ResNet-101

Effect after the changes in the hyperparameters:

When experimenting with the hyperparameters, the following trend was noticed:

- Increasing the image preprocessing, specifically adjusting the resize value, positively impacts the accuracy.
- For instance,

- The hyperparameter details for ResNet50 with batch size = 90, epoch = 10, and image resize = resulted in an accuracy of 93.54%.
- For the same ResNet50 configuration with batch size = 90, epoch = 10, and resize = 170, the accuracy improved to 94.38%.
- This trend was not only noticed in ResNet50 but also in the three ResNet models used.

The ResNet-101 model was trained for 10 epochs, achieving an accuracy of 95.54%. The training was conducted with a learning rate of 0.0008 and a batch size of 64. The ResNet-152 model achieved a precision of 96.17% and was trained over 15 epochs using a learning rate of 0.002 and a batch size of 30.

After evaluating the performance of the three ResNet models, it is clear that the ResNet-152 model demonstrates superior precision and overall effectiveness, achieving an accuracy of 96.17%. Given its higher performance compared to the ResNet-101, ResNet-50, and ResNet-152 models, the ResNet-152 model has been selected for deployment in the production environment. Its ability to deliver more accurate results makes it optimal for real-world applications, ensuring reliable and efficient performance in production scenarios (Table 18).

**Table 18:**
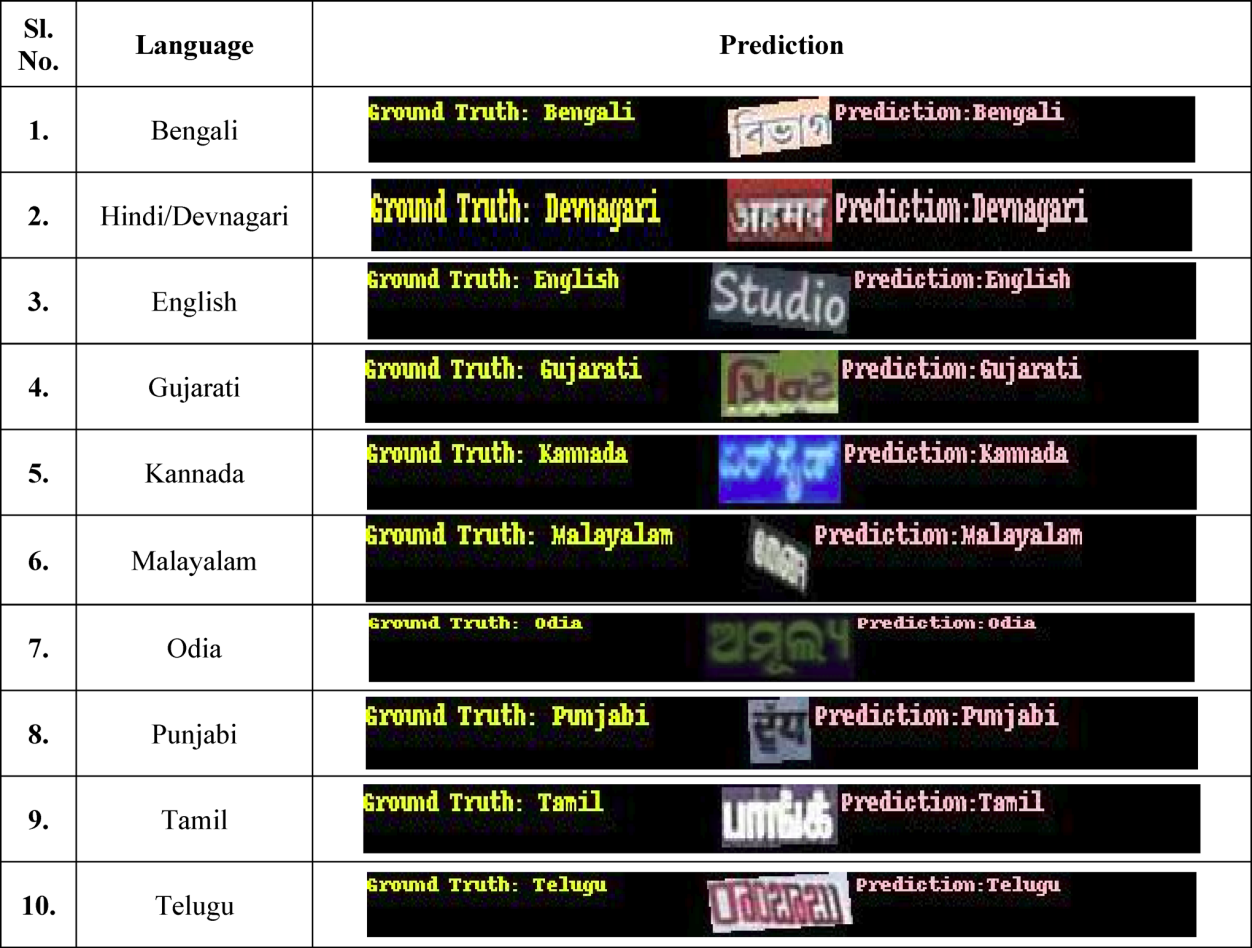
Visualizations of the ground truth and predictions of the 10 languages using ResNet-152

### Object detection (shop sign board detection) using YOLOv5

The mAP obtained is 0.33, which is 33%

The hyperparameter details for YOLOv5 training are mentioned below:

- Image size: 600
- Batch size: 20
- Number of epochs: 50

**Fig. 6:**
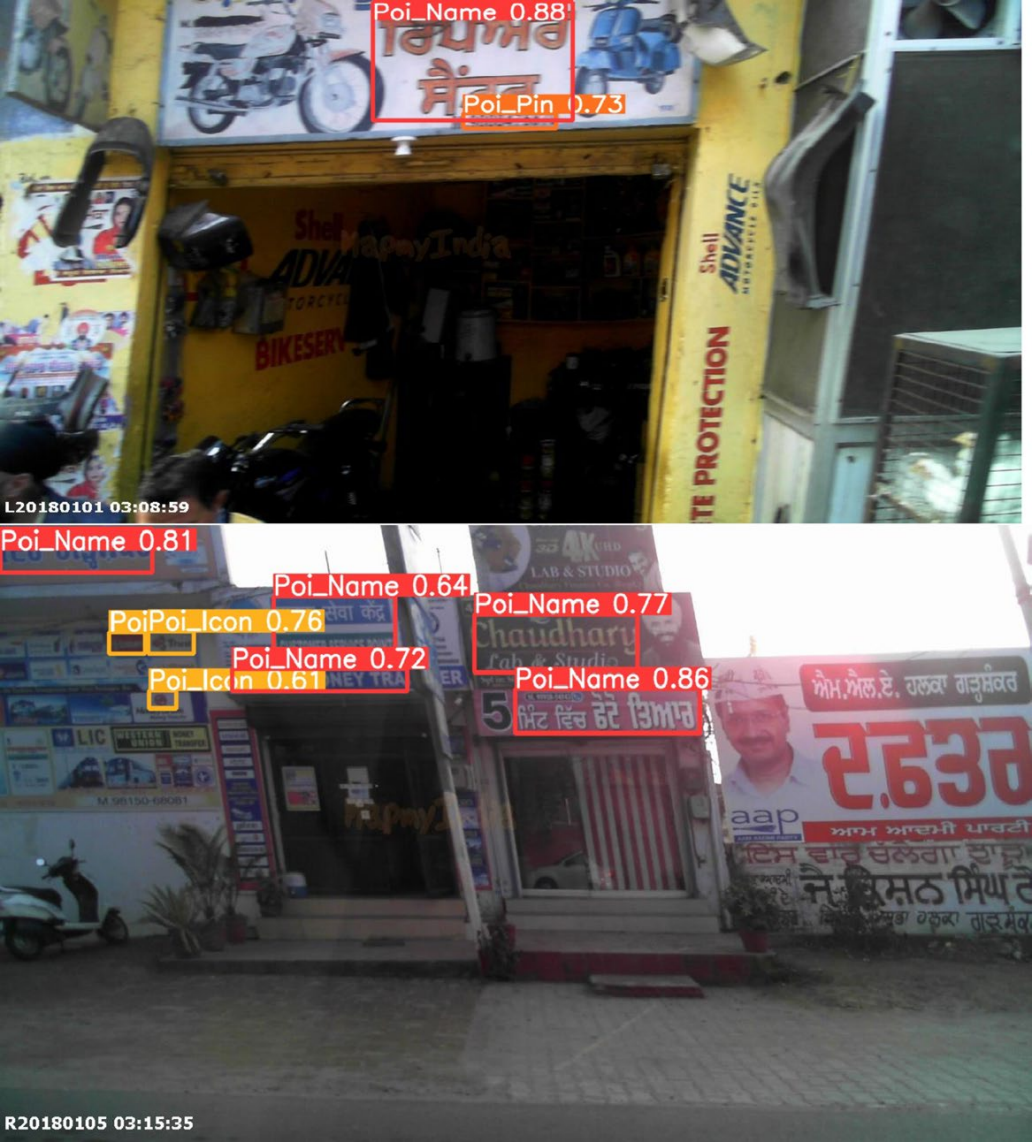
Visualizations of YOLOv5 model for detection of poi_name, Poi_icon with its accuracy.

**Fig. 7:**
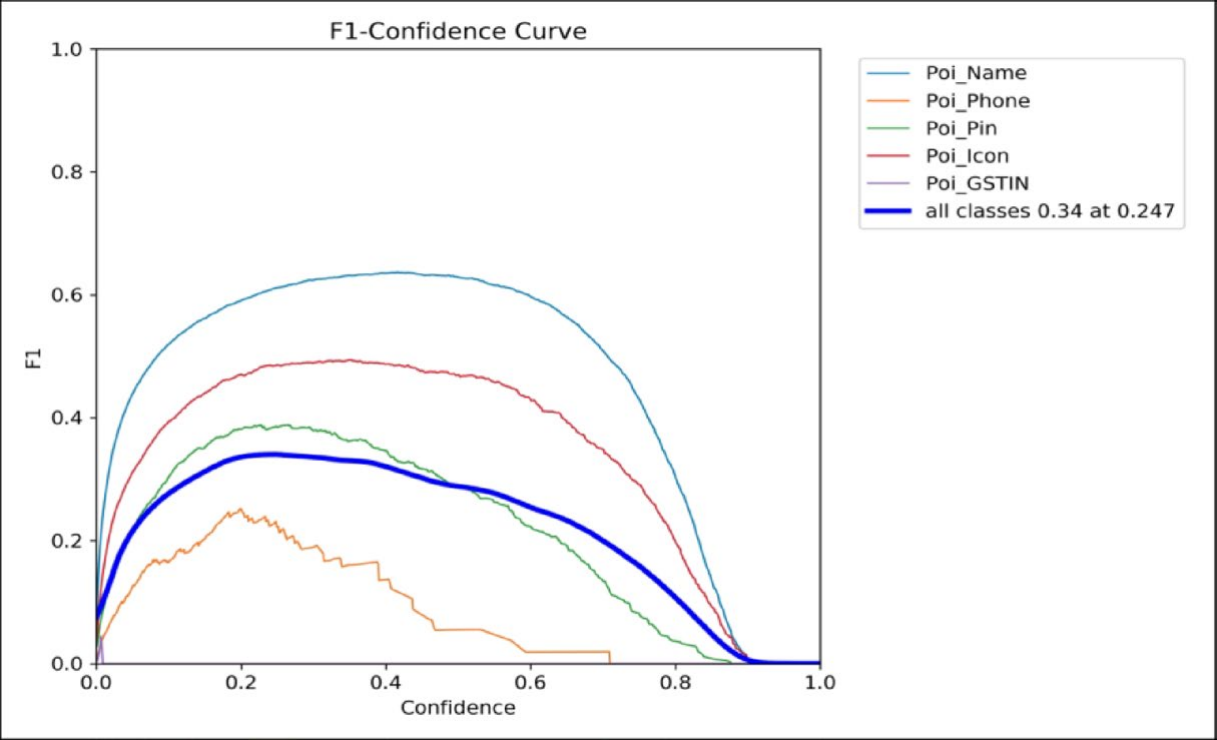
Confidence curve of YOLOv5.

The visualizations of the output of YOLOv5 are shown in Fig. 6. The confidence score of YOLOv5 is mentioned in Fig. 7.

The YOLOv5 design has been employed to build the object identification component, which is responsible for locating necessary POI-related fields in MMS images, including name, phone number, PIN code, GSTIN, and icon regions. This element is fundamental to the direction of subsequent OCR and semantic classification phases.

The developed model’s mean Average Precision (mAP) was 0.33 (33%) across five POI field categories. Even though this value might not seem ideal on its own, it’s crucial to comprehend the nature of the issue and the difficulties with the dataset:

1. Fine-Grained and Sparse Objects: POI fields, like phone numbers, PINs, and GSTIN codes, are frequently incorporated into low-resolution, dense advertising with tiny font sizes. Lower identification ratings are a natural consequence of these traits, particularly for rare and smaller objects.
2. Class Imbalance: restricted expansion of the model across all categories results from some fields (such as POI_Icon or POI_GSTIN) appearing much less frequently in the dataset than others.
3. Visual Inconsistency and Noise: In accordance with bounding box adaptation, the fact that, in contrast to organized documents, real-world signage in Indian cities differs in arrangement, font, color, and placement.

The identification boxes generated by YOLOv5 continue to fulfill their function in the pipeline despite the low mAP value:

1. They serve as suggestions for the OCR module, enabling dictionary-based methods for obtaining and correcting text contained in even approximate boxes.
2. This method increases the overall efficacy of semantic POI obtaining while lessening the effects of inadequate localization.

### Preparation of the dictionary

Developing dictionaries tailored to specific languages identified within the captured images to facilitate accurate text recognition, which can be used to identify points of interest. The dictionary words are extracted from the “labels” of the JSON annotation file. Preparation of the dictionary also included curation by removing the alphanumeric characters, numeric values, punctuation marks, and others. To correct the words predicted by the CRNN model, we need to compare the words with the dictionary. The comparison methods include beam search, Levenshtein distance, and Greedy’s best first search. These methods were compared based on their accuracy to see which method has better accuracy. After calculating the accuracy of these three methods, the greedy best first search algorithm with Levenshtein distance as the parameter has been selected to compare words, which comparatively has better accuracy than the beam search.

## Comparison with the existing system and limitations

In order to verify the efficacy of the implied framework, we have compared it to new state-of-the-art (SOTA) techniques from 2022 to 2025 for both the individual components and the POI extraction process as a whole. The analysis has been presented in Table 19. Our refined ResNet-152 model outperformed the 93.6% recorded on the IndicText measure for script identification, achieving an accuracy of 96.17%^18^.

**Table 19:** Comparison of proposed system inference to previously developed system.

Sl. No	Parameters	Metric	Proposed system	State-of-the-art method	Reference and year
1	Scene text understanding	Word accuracy	92.5%	88.5%	Selvam et al.^19^
2	Scene text identification	Word accuracy	92.5%	91.3%	Vijayan et al.^20^
3	Script detection	Accuracy	96.17%	93.6%	Lunia et al.^18^
4	Point of interest field identification	Average precision	0.33	0.21	Khalid et al.^21^
5	Word correction	Accuracy gain	9 to 17%	Not available	

Our pipeline, which combined dictionary-based modification and CRNN, obtained 92.5%, outperformed Selvam et al.^19^, who used a transformer-based OCR structure, with 88.5% word-level precision in the scene recognition of the text stage. Utilizing a YOLOv4-BiLSTM hybrid approach, Khalid et al.^21^ stated a mean Average Precision (mAP) of 21% for POI field identification, whereas our YOLOv5-based component accomplished an average mAP of 33%. Lastly, our word modification method, which used greedy best-first search and Levenshtein distance, greatly enhanced understanding outcomes. Accuracy gains ranged from 9 to 17% depending on the language and noise level. These outcomes show how well our system performs and how flexible it is to comprehend multilingual scene-text and extract POI in real-world scenarios.

Even though the suggested system performs admirably in multilingual POI extraction, several inherent difficulties and restrictions were faced throughout development. Script categorization is intrinsically challenging because the system must function across more than ten Indic scripts, many of which have aesthetically identical letter sets (e.g., Bengali and Odia). The low resolution of MMS images, which frequently have motion blur, occlusion, and perspective distortion, adds even more complexity. Furthermore, POI fields, like phone numbers and PIN codes, are often embedded in disorganized pictures with irregular formatting and are small and densely packed. Accuracy in both identification and recognition is adversely affected by these factors. Major problems were noted at the dataset level, including class imbalance, a lack of annotated specimens for scripts with limited resources, and divergent annotations brought on by mistakes in manual labeling. Furthermore, the reliability of ground truth data was limited because in-scene text was occasionally hardly readable even by human annotators, particularly for smaller or more dilapidated signboards. The dataset also showed uneven object sizes and inconsistent image quality, making it more difficult for the model to generalize well in various real-world scenarios.

## Conclusion

The present study presents a novel architecture that combines cutting-edge techniques in object identification, language categorization, and recognition of text to extract Points of Interest (POI) from Indic language name boards. With an astounding precision of 96.17%, ResNet-152 outperformed the other models under evaluation, making it the best option for production deployment because of its accuracy and dependability. With a mAP score of 33%, the YOLOv5 model was used to recognize store signboards, indicating the need for more object detection job improvement. A CTC-based decoding pipeline combined with a dedicated spelling correction module was created to improve text recognition accuracy. This component efficiently improves the output by using carefully chosen language-specific vocabularies and sophisticated word evaluation methods. Among word correction techniques, greedy best-first search with Levenshtein distance proved to be the most accurate, outperforming beam search and other options. The suggested system demonstrates its ability to handle Indic scripts’ linguistic diversity and intricacy, providing a solid answer for practical uses in automated data collecting, regional text interpretation, and navigation. The proposed system outperforms the previously developed system compared to existing systems based on benchmarks like Indic text and transformer-based OCR. The integrated structure, which combines standard deep learning modules with specially designed modifications for dictionary-based correction, middleware coordination, and Indic scripts, is what makes this study novel. In comparison to current state-of-the-art methods, this design achieves superior performance and offers a workable solution for multilingual POI extraction in the real world. In order to raise the mAP score, future improvements may concentrate on enhancing YOLOv5’s efficiency by adjusting hyperparameters and combining it with more reliable object identification models. Text recognition precision may also be increased by adding new scripts and local variations to the lexicon and using transliteration methods. Adding Indic swipe gesture typing processes is an exciting opportunity to expand on the current design. Initial design and interpreting components have demonstrated promise for enhancing user interaction in mobile contexts. Examples include dictionary-based spelling enhancement and CTC path decoding in conjunction with CRNN models. The development and thorough evaluation of this module within the multilingual recognition pipeline will be the main goals of future research.

## Supplementary Information

Below is the link to the electronic supplementary material.

Supplementary Material 1

Supplementary Material 2

## Data Availability

All data generated or analyzed during this study are included in this published article.
