# Supplementary material for: Integrated framework utilizing scene text detection and recognition techniques for enhancing point of interest extraction from name boards in all Indic languages
Source: Sci Rep. 2026 Mar 10;16:12907. doi: 10.1038/s41598-026-40742-w (PMC13096107; doi:10.1038/s41598-026-40742-w)
Supplement: Supplementary file 1 — Supplementary Material 1 [file 41598_2026_40742_MOESM1_ESM.zip › Codes for training and testing/Evaluating Model Predictions Against Ground Truth Labels.pdf]

Evaluating Model Predictions Against Ground Truth Labels

March 6, 2025

[ ]:
```
The intent of the script is to evaluate the accuracy of a trained model on a test dataset.
It loads a pre-trained model, prepares a test dataloader
with labeled test images, generates predictions,
and calculates the accuracy of the predictions against the true labels.
```

[ ]:
```
learner = load_learner('/path/to/trained_weight.pth')
```

[ ]:
```
test_path = '/path/to/test/data'
```

[ ]:
```
test_files = get_image_files(test_path)
```

[ ]:
```
test_dl = learn.dls.test_dl(test_files, with_labels=True)
```

[ ]:
```
preds, targets = learn.get_preds(dl=test_dl, with_input=False, with_decoded=False, with_loss=False)
```

[ ]:
```
print(preds)
```

[ ]:
```
print(targets)
```

[ ]:
```
 predicted_labels = preds.argmax(dim=1)
```

[ ]:
```
predicted_labels = predicted_labels.numpy()
targets = targets.numpy()
```

[ ]:
```
accuracy = (predicted_labels == targets).mean()
print(f"Accuracy: {accuracy:.4f}")
```
