# Supplementary material for: Integrated framework utilizing scene text detection and recognition techniques for enhancing point of interest extraction from name boards in all Indic languages
Source: Sci Rep. 2026 Mar 10;16:12907. doi: 10.1038/s41598-026-40742-w (PMC13096107; doi:10.1038/s41598-026-40742-w)
Supplement: Supplementary file 1 — Supplementary Material 1 [file 41598_2026_40742_MOESM1_ESM.zip › Codes for training and testing/Image Classification with FastAI and ResNet.pdf]

Image Classification with FastAI and ResNet

March 6, 2025

[ ]:
```
The intent of the script is to perform image classification
using the FastAI library with a ResNet50 model.
It includes data preparation, model creation, learning rate finding,
mixed precision training (FP16), early stopping, fine-tuning, and model export.
```

[ ]:
```
path = Path('/path/to/train/images')
```

[ ]:
```
#Set up Datablock for image classification Problem

dblock = DataBlock(blocks=(ImageBlock, CategoryBlock),
                   get_items=get_image_files,
                   splitter=RandomSplitter(valid_pct=0.2, seed=42),
                   get_y=parent_label,
                   item_tfms=Resize(200),
                   batch_tfms=[*aug_transforms(size=200), Normalize.
 ↪from_stats(*imagenet_stats)])
```

[ ]:
```
#Define batch size

batch_size = 90
```

[ ]:
```
dls = dblock.dataloaders(path, bs=batch_size)
```

[ ]:
```
# the resnet models can be switched between resnet50,resnet101, and resnet152)
learn = cnn_learner(dls, resnet50, metrics=accuracy, cbs=ShowGraphCallback())
```

[ ]:
```
learn.lr_find()
```

[ ]:
```
learn = learn.to_fp16()
```

[ ]:
```
from fastai.callback.tracker import EarlyStoppingCallback
```

[ ]:
```
early_stop = EarlyStoppingCallback(monitor='valid_loss', min_delta=0.01,␣
 ↪patience=3)
```

[ ]:
```
learn.fine_tune(epochs=15, base_lr=0.0014454397605732083, cbs=early_stop)
```

[ ]:

```
learn.export('/path/to/save/trained_weight.pth')
```
