# Supplementary material for: Integrated framework utilizing scene text detection and recognition techniques for enhancing point of interest extraction from name boards in all Indic languages
Source: Sci Rep. 2026 Mar 10;16:12907. doi: 10.1038/s41598-026-40742-w (PMC13096107; doi:10.1038/s41598-026-40742-w)
Supplement: Supplementary file 1 — Supplementary Material 1 [file 41598_2026_40742_MOESM1_ESM.zip › Codes for training and testing/Test Dataset Evaluation with Incorrect Image Saving-1.pdf]

Test Dataset Evaluation with Incorrect Image Saving

March 6, 2025

```
[ ]: The script's intent is to evaluate a trained model's performance on a test␣
     ↪dataset
     and save incorrectly predicted images to a specified directory for further␣
     ↪analysis.
     It calculates accuracy, counts correct and incorrect predictions,
     and moves misclassified images to a designated folder.
```

```
[ ]: learner_path = '/pth/to/trained_model_weight.pth'
```

```
[ ]: learn = load_learner(learner_path)
```

```
[ ]: test_path = '/path/to/test/data'
```

```
[ ]: incorrect_dir = '/path/to/save/incorrect/images'
```

```
[ ]: os.makedirs(incorrect_dir, exist_ok=True)
```

```
[ ]: test_files = get_image_files(test_path)
```

```
[ ]: test_dl = learn.dls.test_dl(test_files, with_labels=True)
```

```
[ ]: preds, targets = learn.get_preds(dl=test_dl, with_input=False,␣
     ↪with_decoded=False, with_loss=False)
```

```
[ ]: predicted_labels = preds.argmax(dim=1).numpy()
     targets = targets.numpy()
```

```
[ ]: accuracy = (predicted_labels == targets).mean()
     print(f"Accuracy: {accuracy:.4f}")
```

```
[ ]: correct_count = int(accuracy * len(targets))
     incorrect_count = len(targets) - correct_count
```

```
[ ]: for file, predicted_label, true_label in zip(files, predicted_labels, targets):
         if predicted_label != true_label:
             # Copy incorrect file to the incorrect directory
             shutil.copy(file, os.path.join(incorrect_dir, os.path.basename(file)))
```

[ ]:
```
print(f"Incorrectly predicted images have been saved to {incorrect_dir}.")
```
