# Supplementary figures and images for: Integrated framework utilizing scene text detection and recognition techniques for enhancing point of interest extraction from name boards in all Indic languages (part 2 of 2)
Source: Sci Rep. 2026 Mar 10;16:12907. doi: 10.1038/s41598-026-40742-w (PMC13096107; doi:10.1038/s41598-026-40742-w)

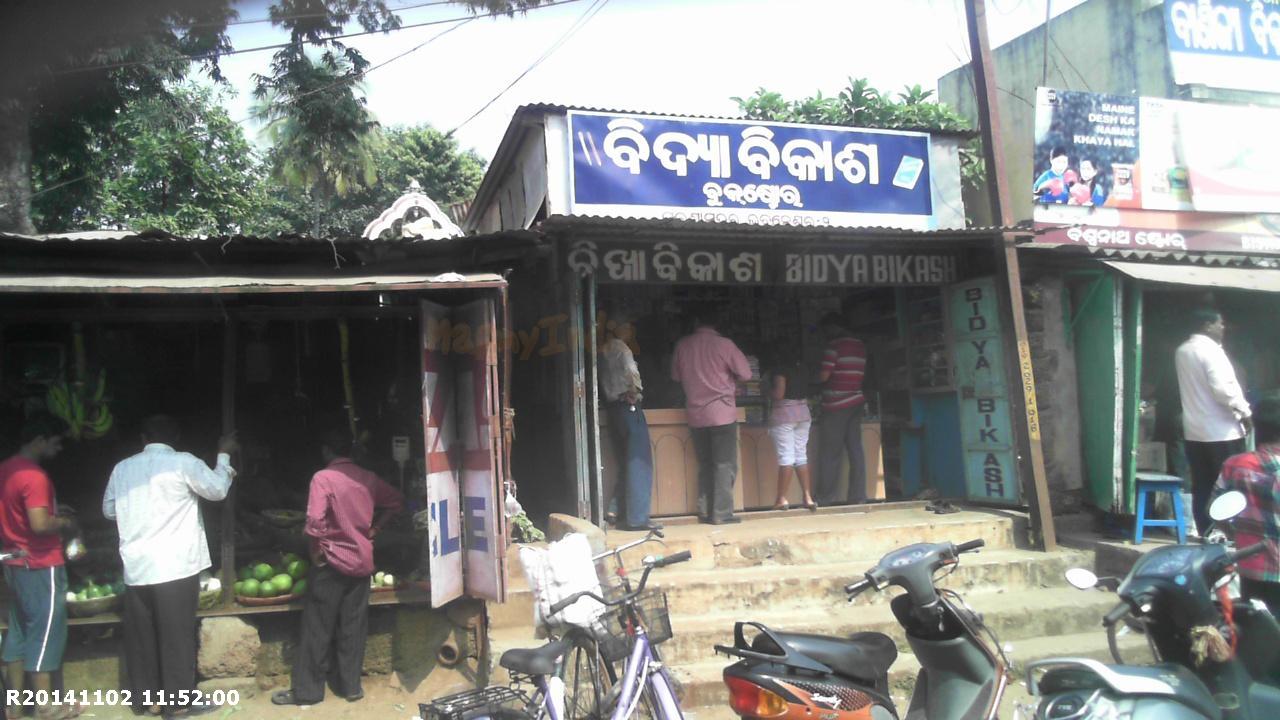

Supplement: Supplementary file 2 — Supplementary Material 2 [file 41598_2026_40742_MOESM2_ESM.zip › sample_data_yolov5/R_11-02_11.52.00.jpg]

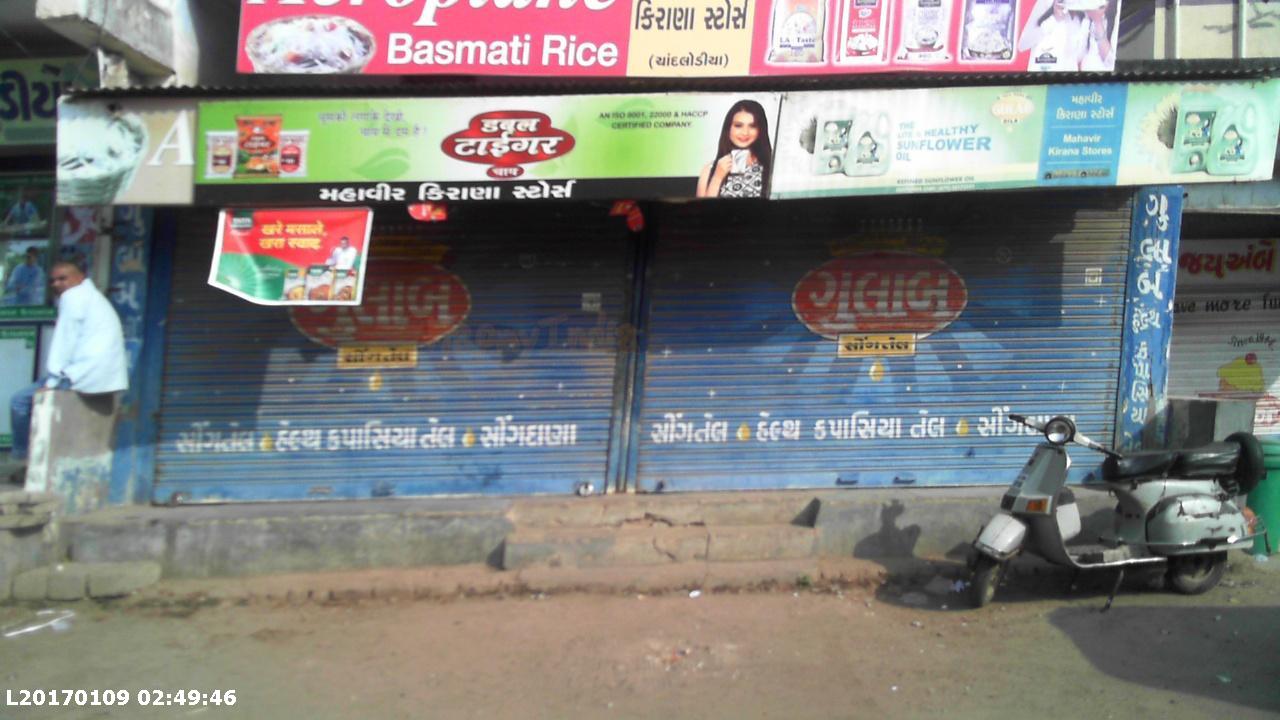

Supplement: Supplementary file 2 — Supplementary Material 2 [file 41598_2026_40742_MOESM2_ESM.zip › sample_data_yolov5/01-09 02.49.46.jpg]

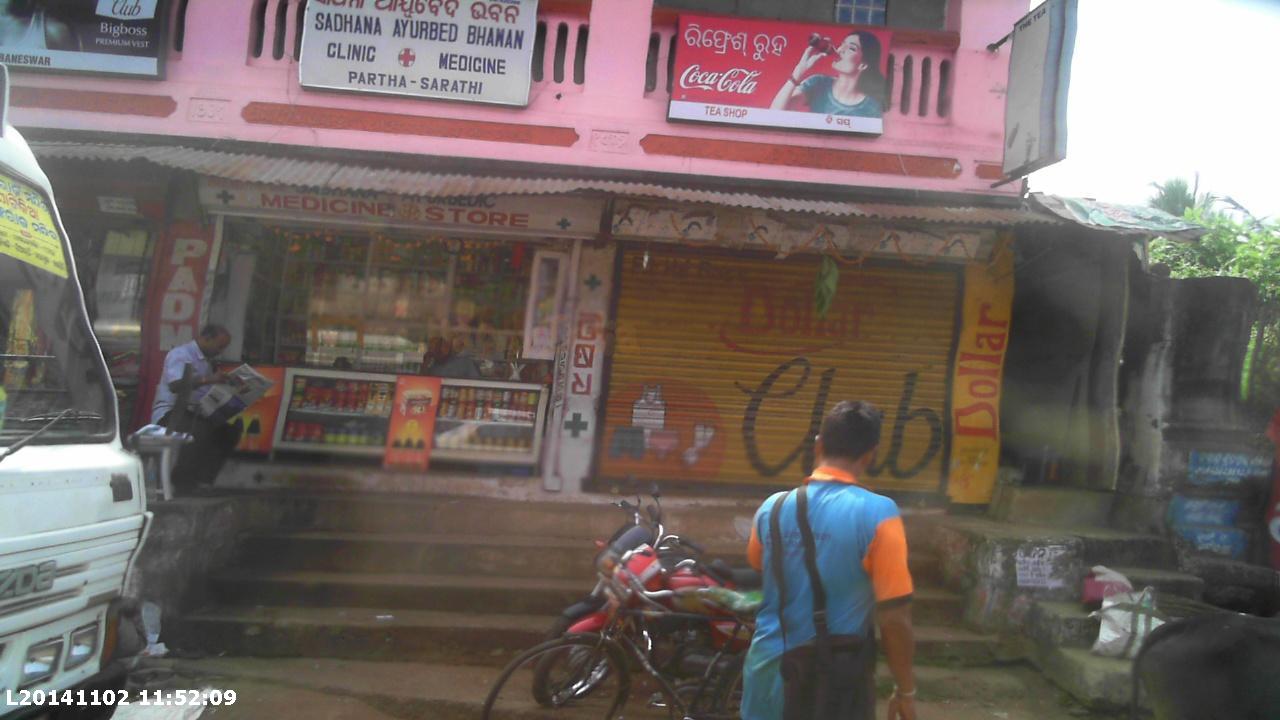

Supplement: Supplementary file 2 — Supplementary Material 2 [file 41598_2026_40742_MOESM2_ESM.zip › sample_data_yolov5/L_11-02_11.52.09.jpg]

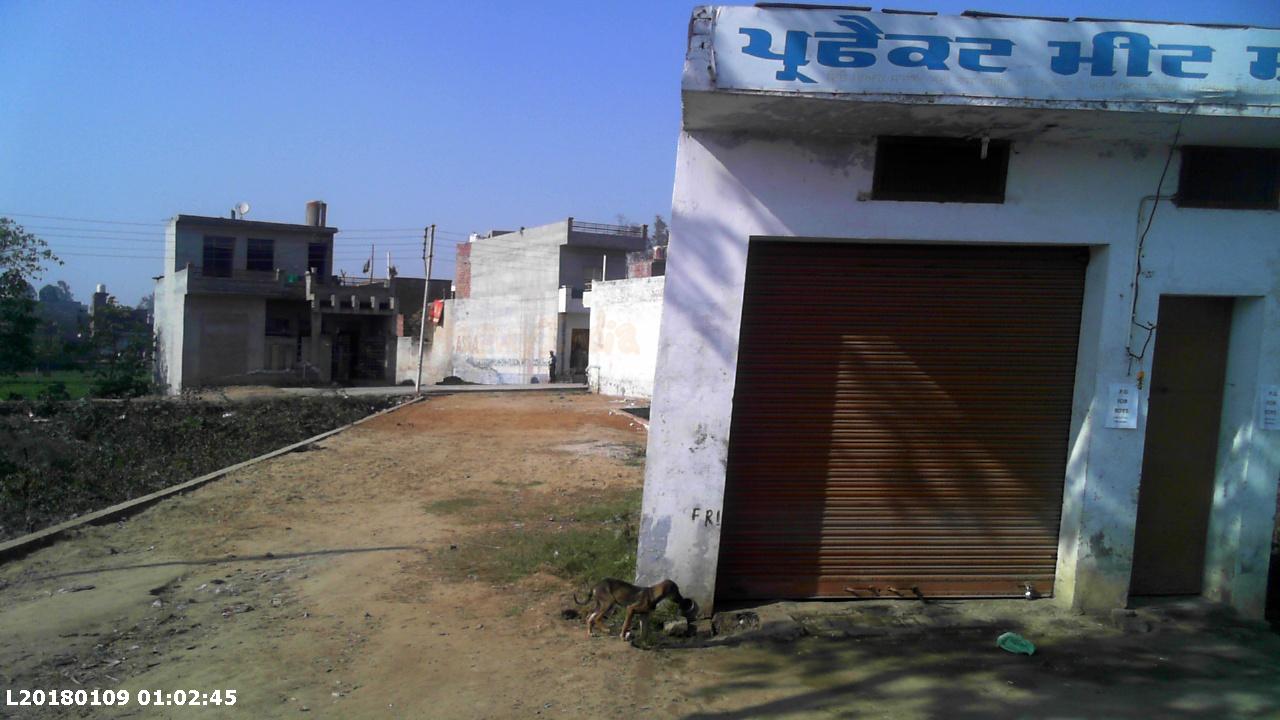

Supplement: Supplementary file 2 — Supplementary Material 2 [file 41598_2026_40742_MOESM2_ESM.zip › sample_data_yolov5/01-09 01.02.45.jpg]

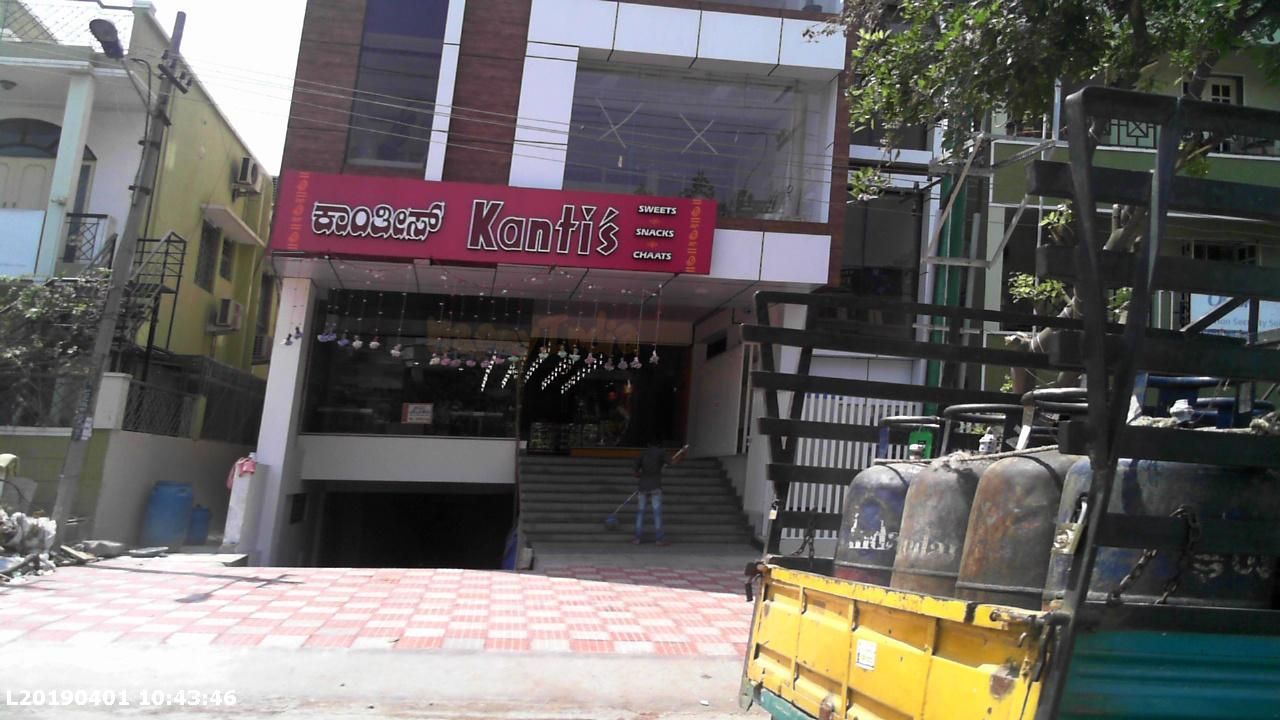

Supplement: Supplementary file 2 — Supplementary Material 2 [file 41598_2026_40742_MOESM2_ESM.zip › sample_data_yolov5/04-01_10.43.46.jpg]

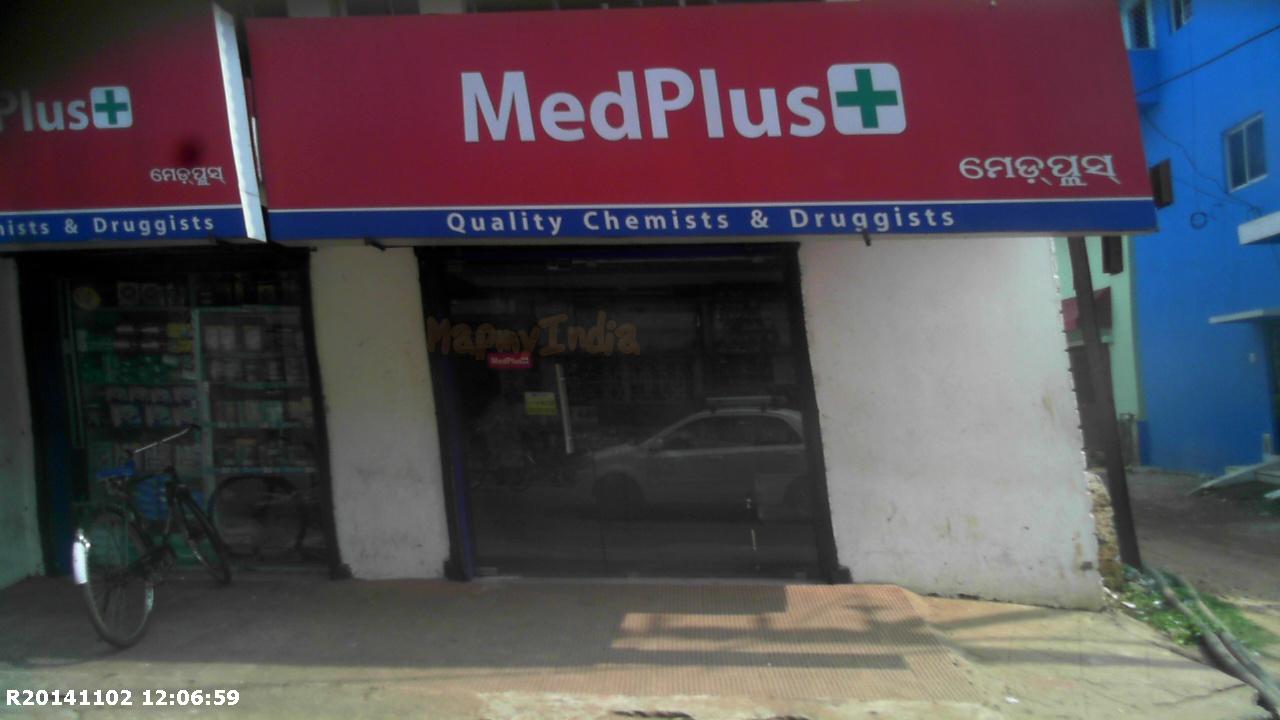

Supplement: Supplementary file 2 — Supplementary Material 2 [file 41598_2026_40742_MOESM2_ESM.zip › sample_data_yolov5/R_11-02_12.06.59.jpg]

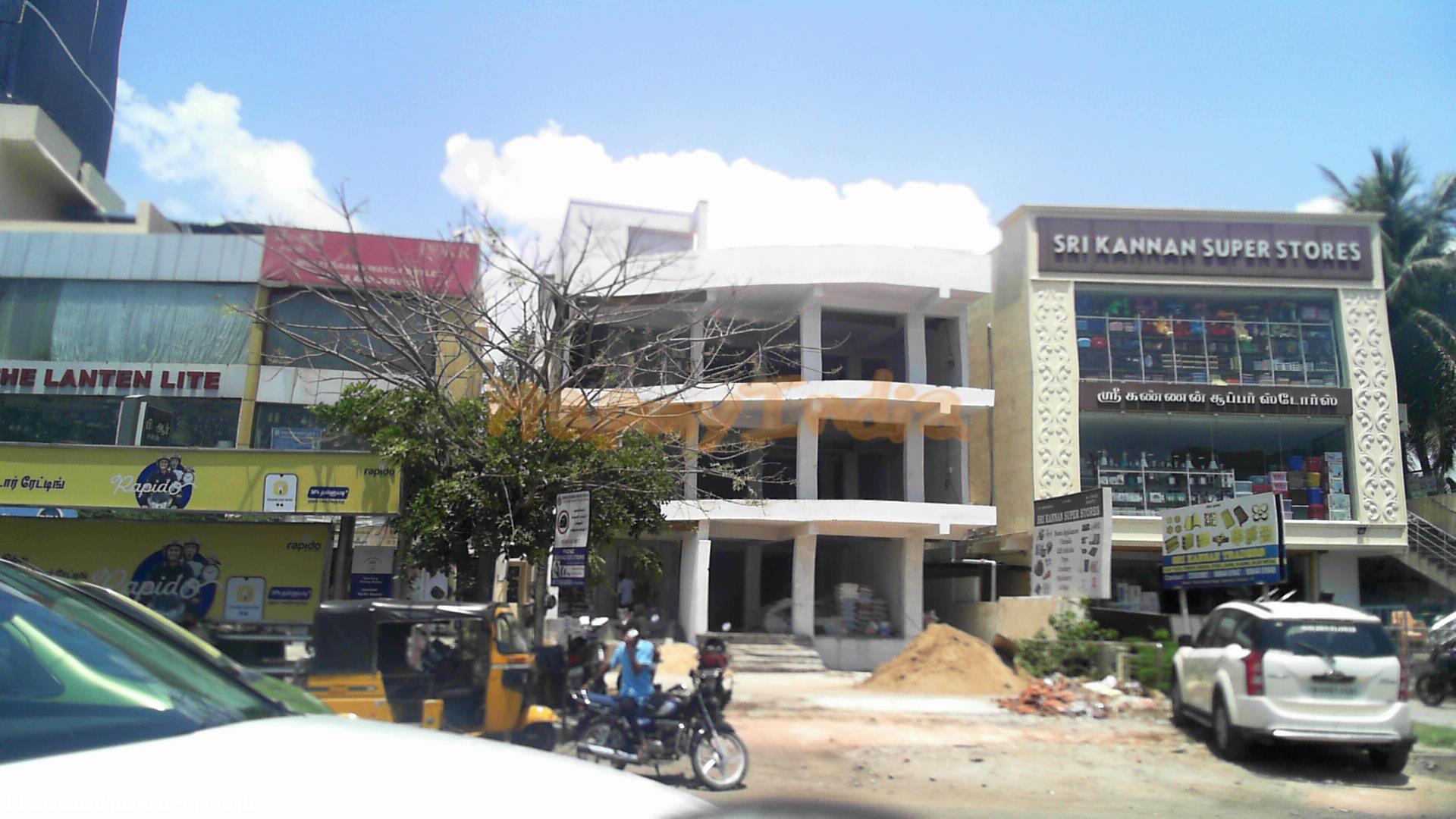

Supplement: Supplementary file 2 — Supplementary Material 2 [file 41598_2026_40742_MOESM2_ESM.zip › sample_data_yolov5/LCOM60810042021112519.jpg]

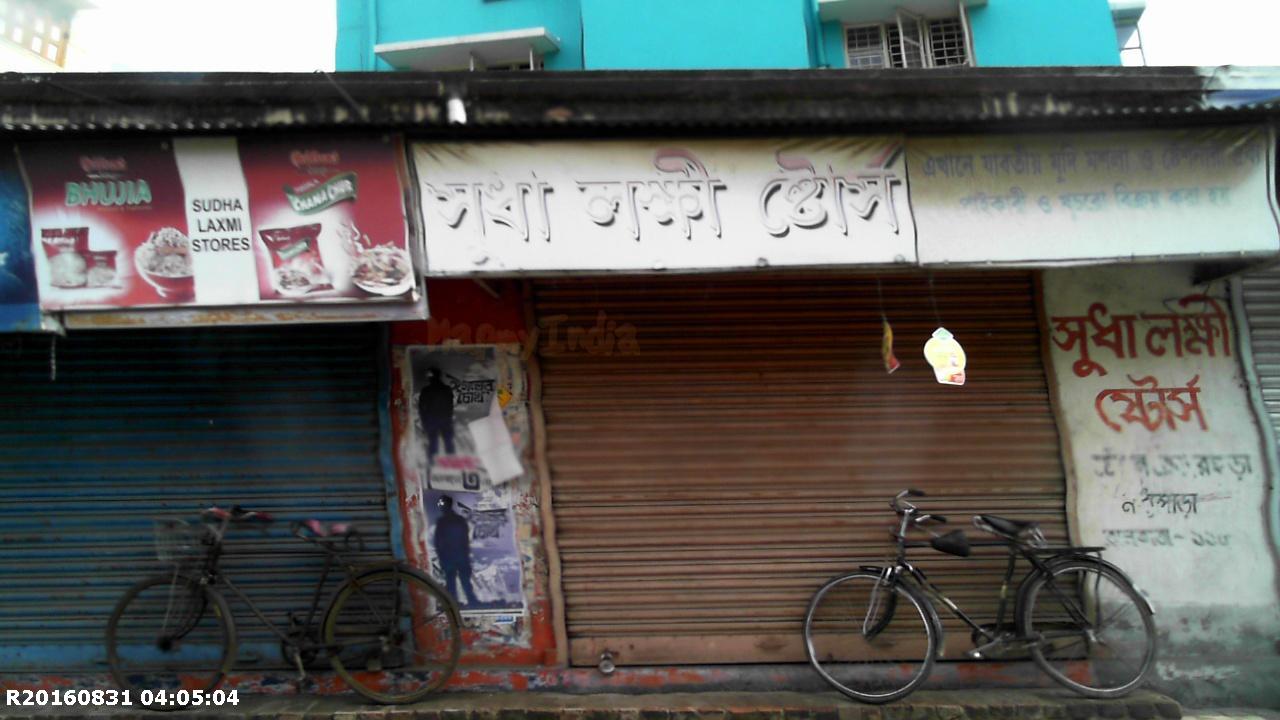

Supplement: Supplementary file 2 — Supplementary Material 2 [file 41598_2026_40742_MOESM2_ESM.zip › sample_data_yolov5/R_08-31_04.05.04.jpg]

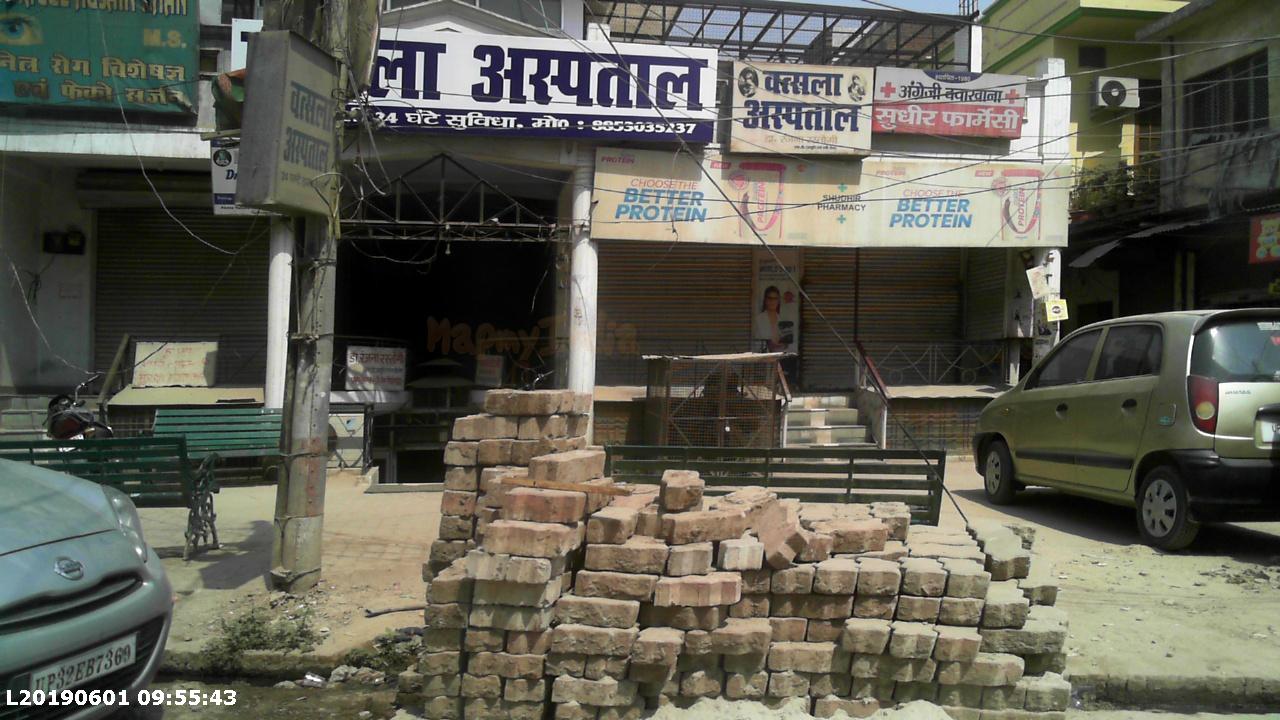

Supplement: Supplementary file 2 — Supplementary Material 2 [file 41598_2026_40742_MOESM2_ESM.zip › sample_data_yolov5/L_06-01_09.55.43.jpg]

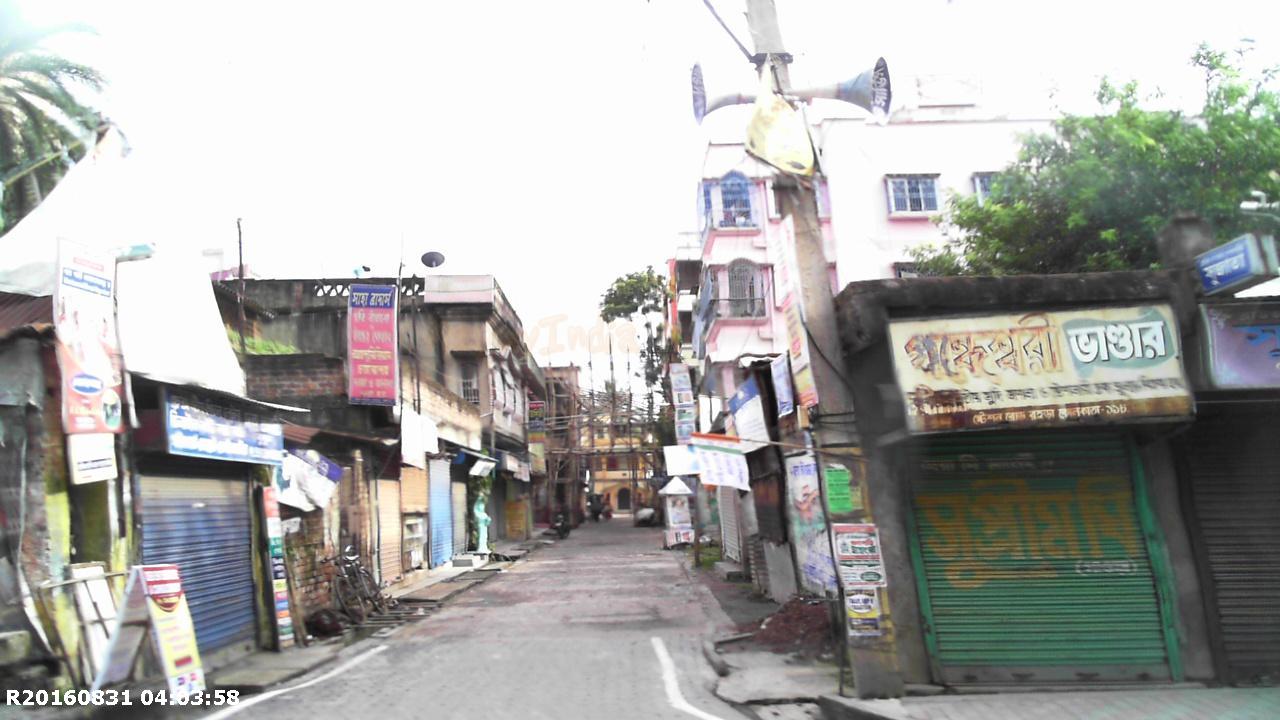

Supplement: Supplementary file 2 — Supplementary Material 2 [file 41598_2026_40742_MOESM2_ESM.zip › sample_data_yolov5/R_08-31_04.03.58.jpg]

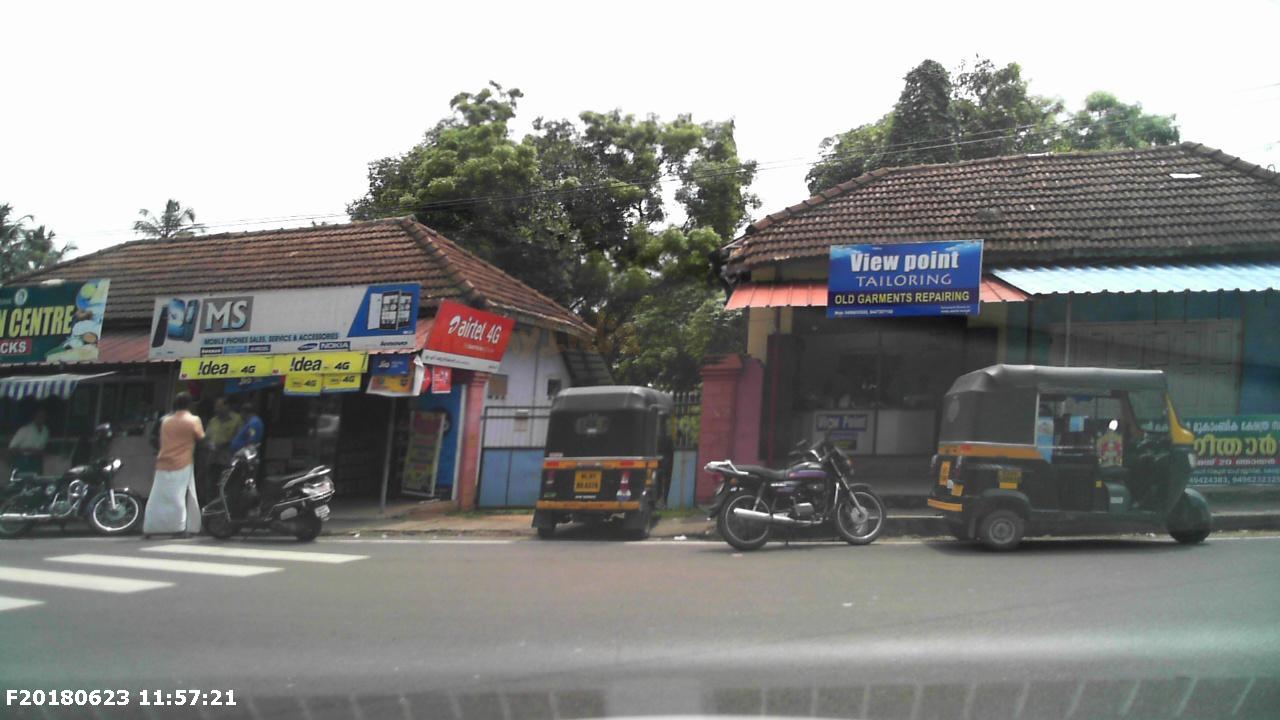

Supplement: Supplementary file 2 — Supplementary Material 2 [file 41598_2026_40742_MOESM2_ESM.zip › sample_data_yolov5/F_06-23_11.57.21.jpg]

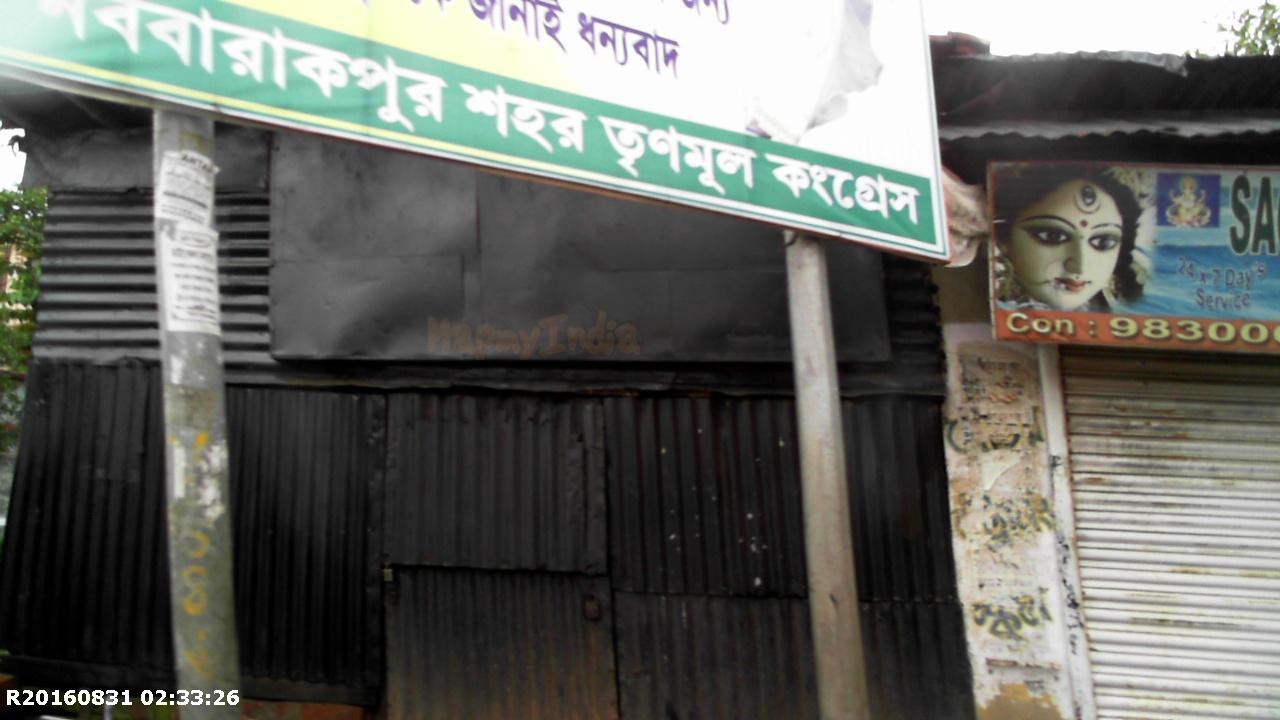

Supplement: Supplementary file 2 — Supplementary Material 2 [file 41598_2026_40742_MOESM2_ESM.zip › sample_data_yolov5/R_08-31_02.33.26.jpg]

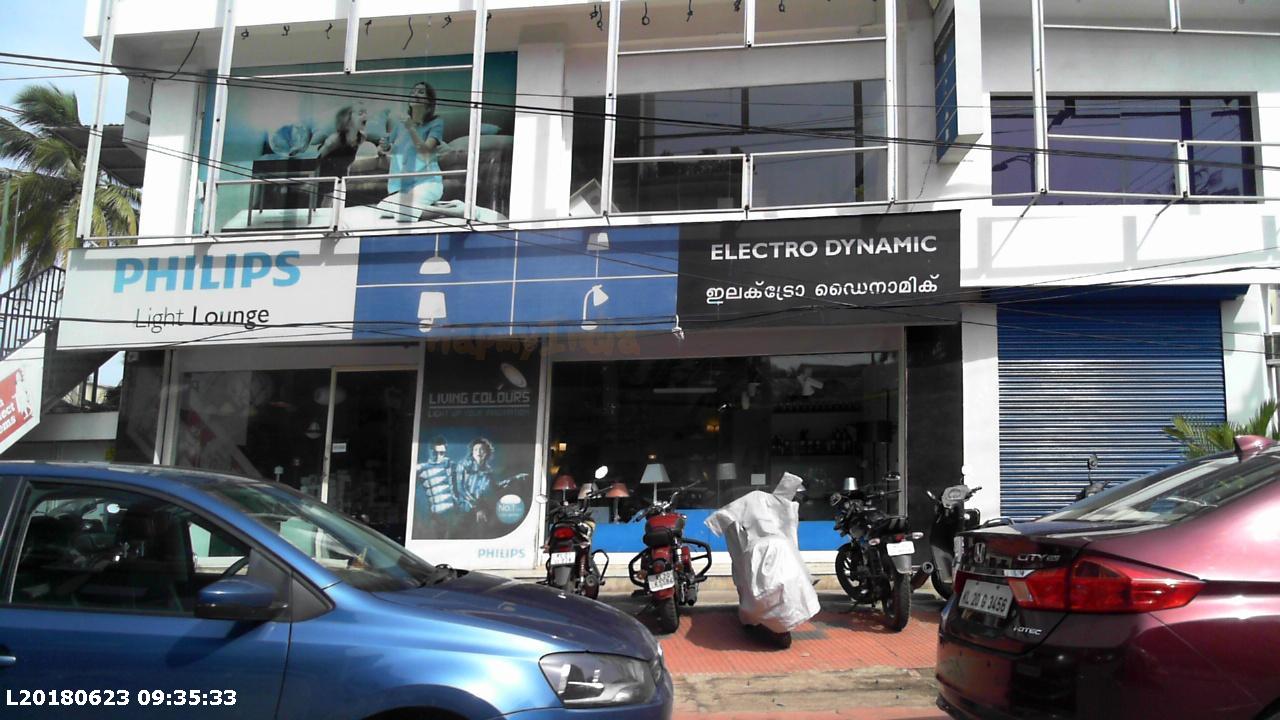

Supplement: Supplementary file 2 — Supplementary Material 2 [file 41598_2026_40742_MOESM2_ESM.zip › sample_data_yolov5/L_06-23_09.35.33.jpg]

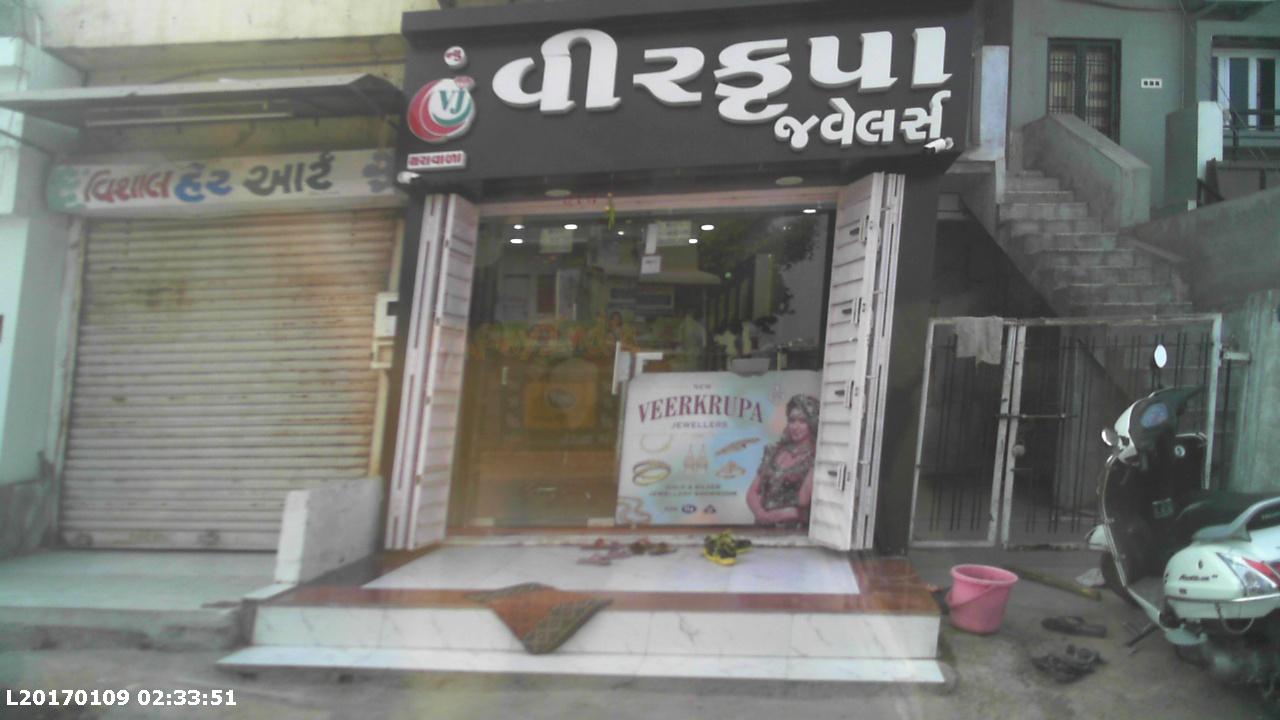

Supplement: Supplementary file 2 — Supplementary Material 2 [file 41598_2026_40742_MOESM2_ESM.zip › sample_data_yolov5/01-09 02.33.51.jpg]

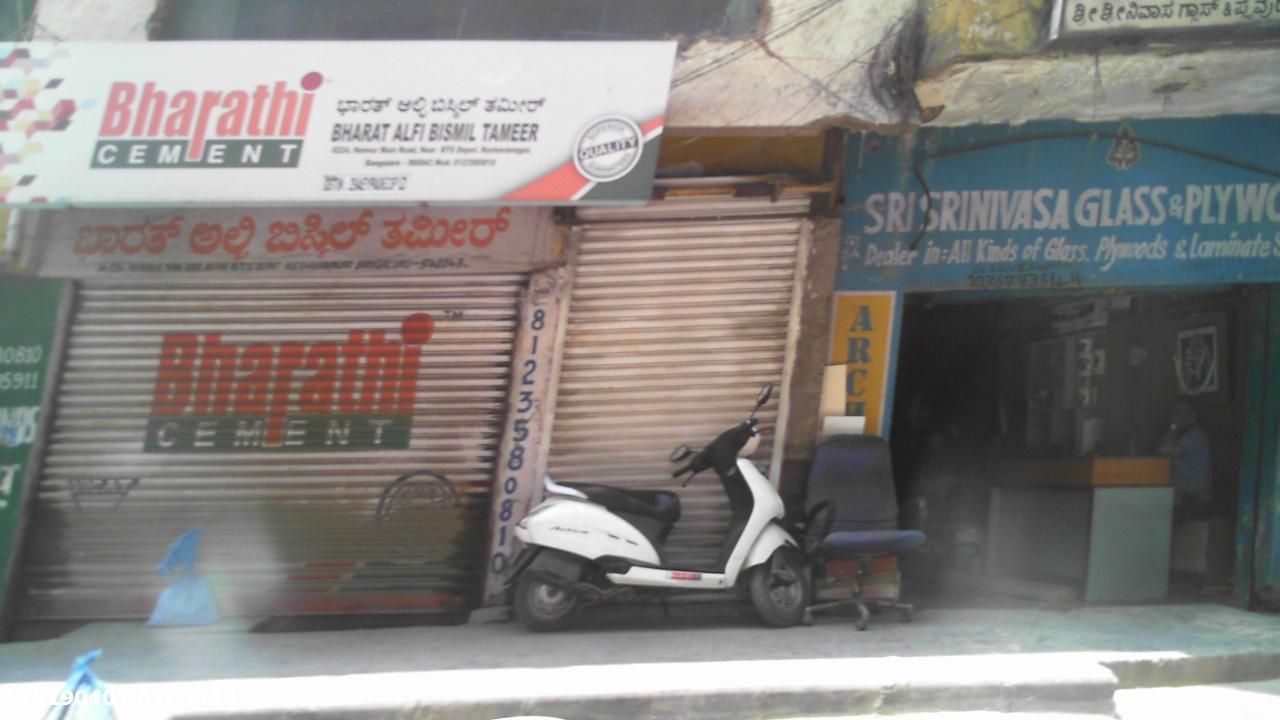

Supplement: Supplementary file 2 — Supplementary Material 2 [file 41598_2026_40742_MOESM2_ESM.zip › sample_data_yolov5/04-01_01.20.44.jpg]

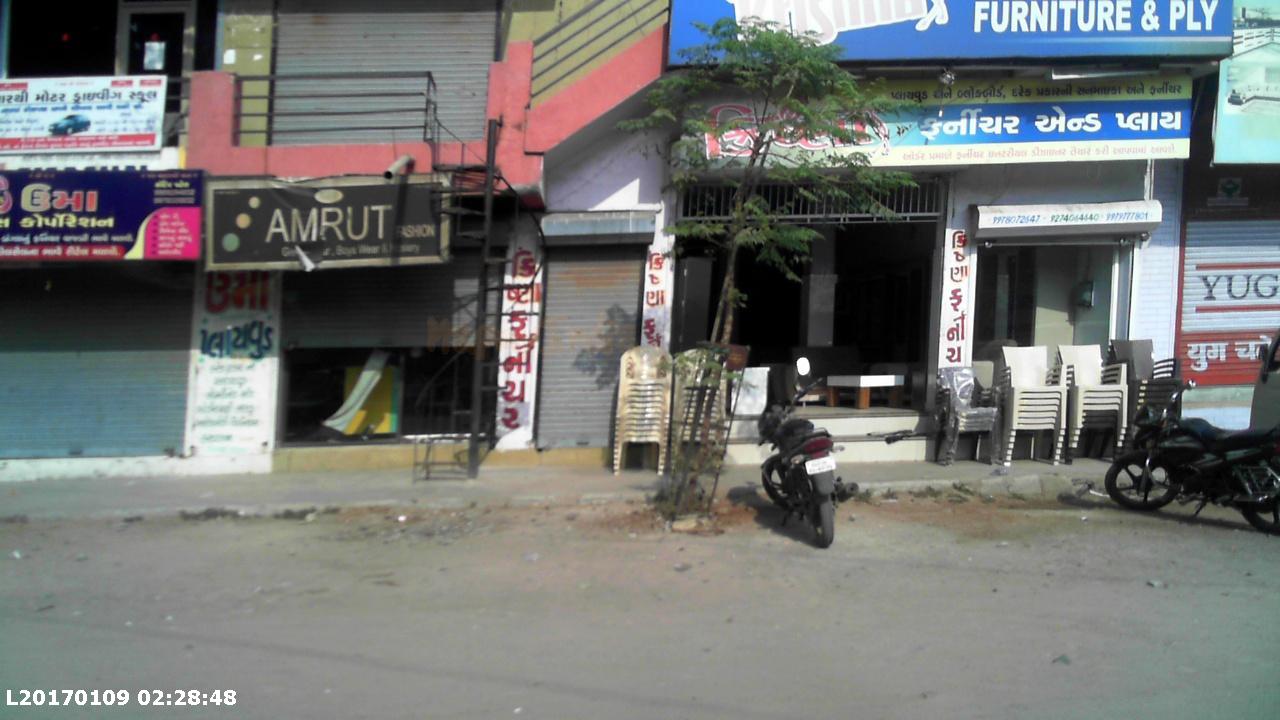

Supplement: Supplementary file 2 — Supplementary Material 2 [file 41598_2026_40742_MOESM2_ESM.zip › sample_data_yolov5/01-09 02.28.48.jpg]

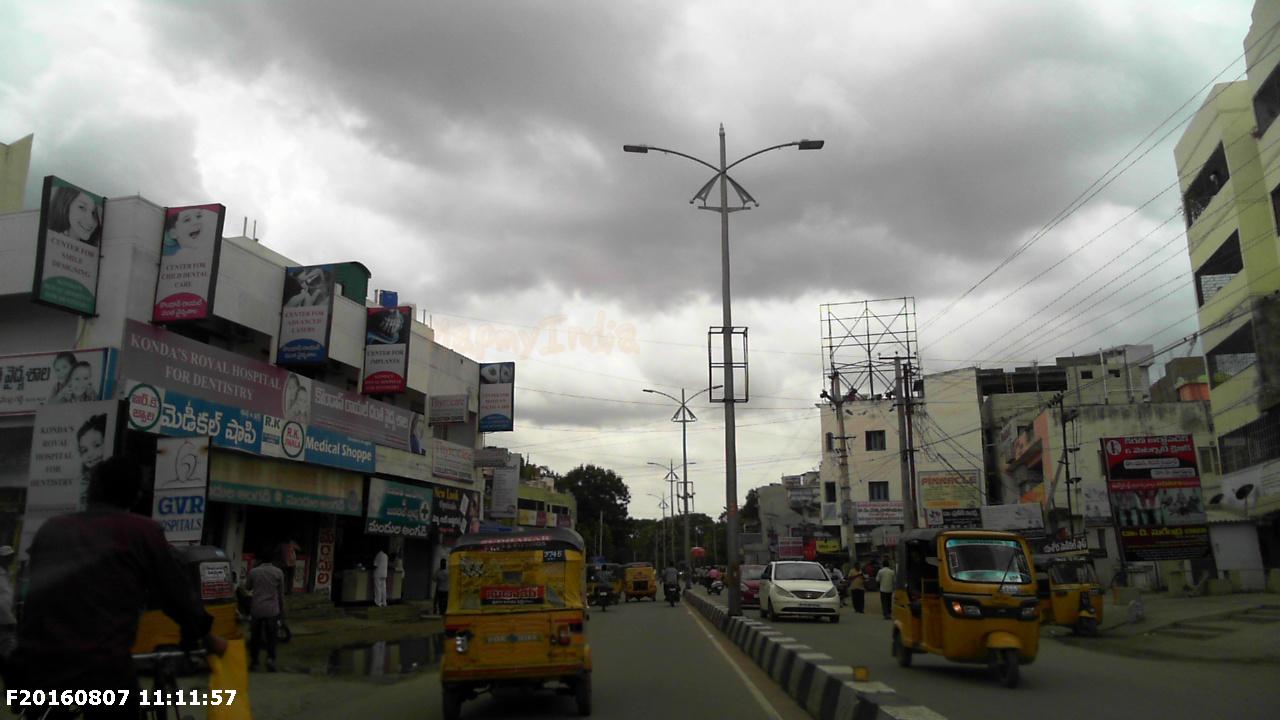

Supplement: Supplementary file 2 — Supplementary Material 2 [file 41598_2026_40742_MOESM2_ESM.zip › sample_data_yolov5/08-07 11.11.57.jpg]

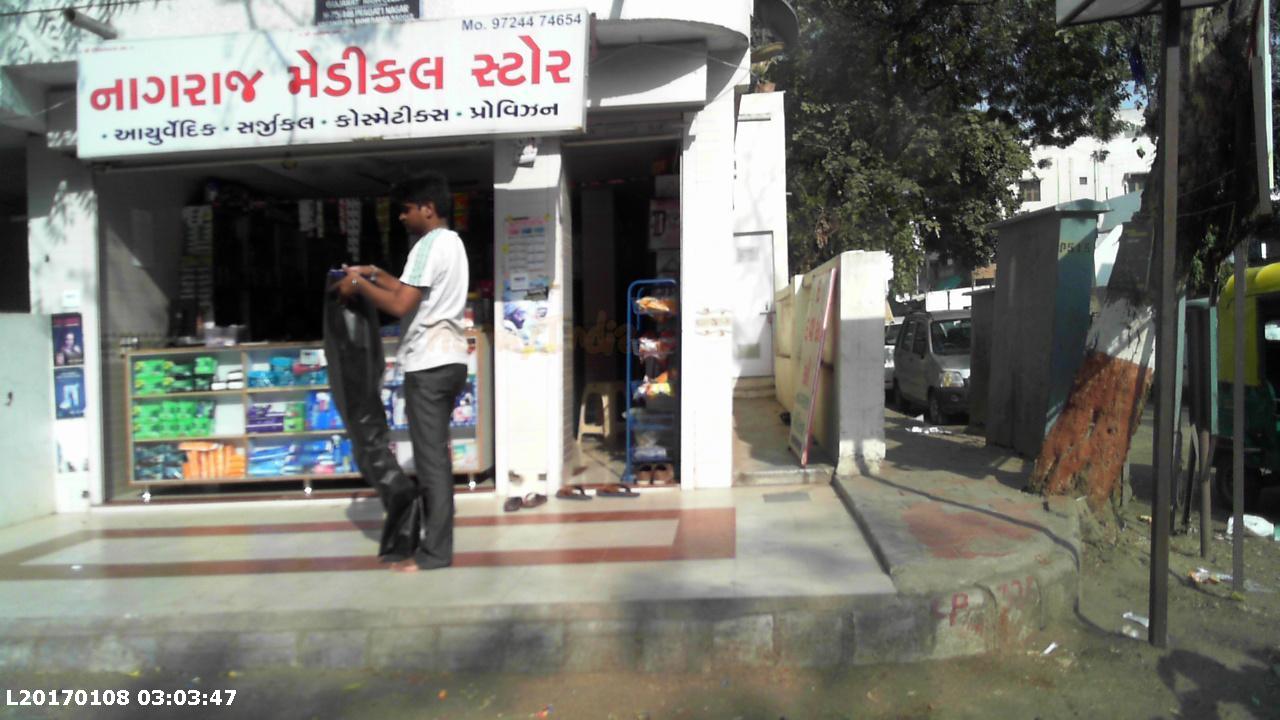

Supplement: Supplementary file 2 — Supplementary Material 2 [file 41598_2026_40742_MOESM2_ESM.zip › sample_data_yolov5/01-08 03.03.47.jpg]

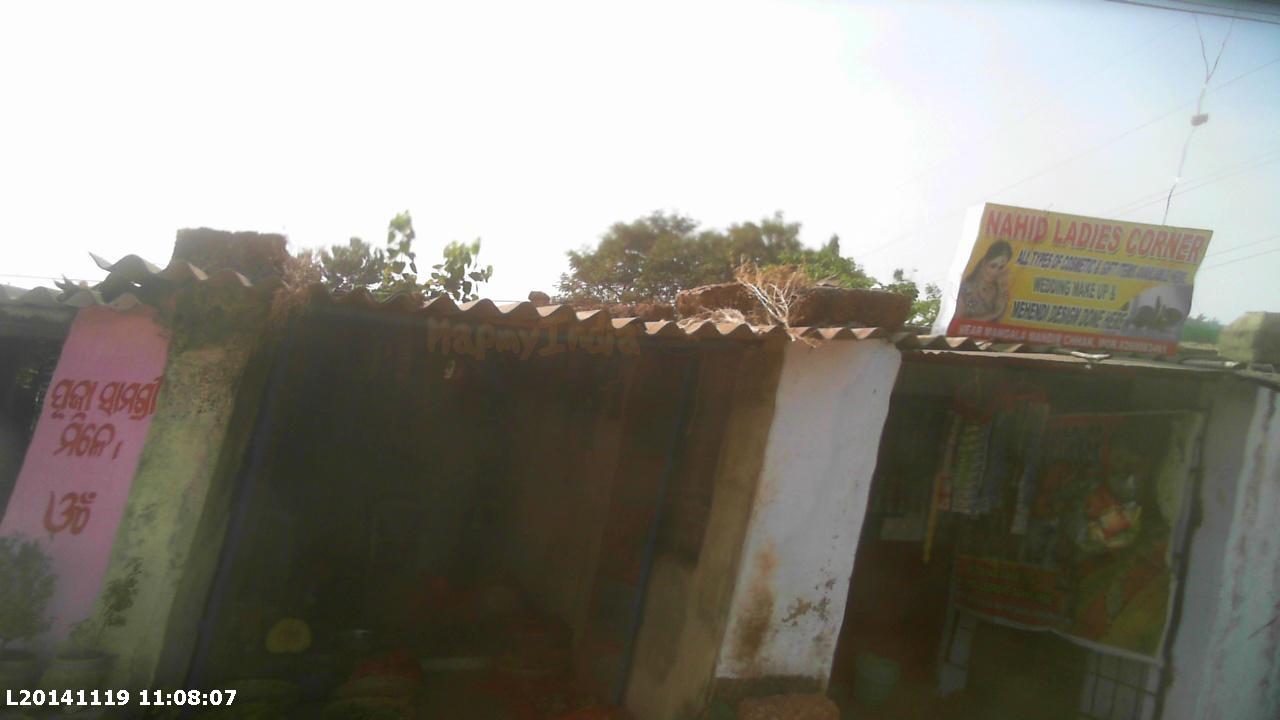

Supplement: Supplementary file 2 — Supplementary Material 2 [file 41598_2026_40742_MOESM2_ESM.zip › sample_data_yolov5/L_11-19_11.08.07.jpg]

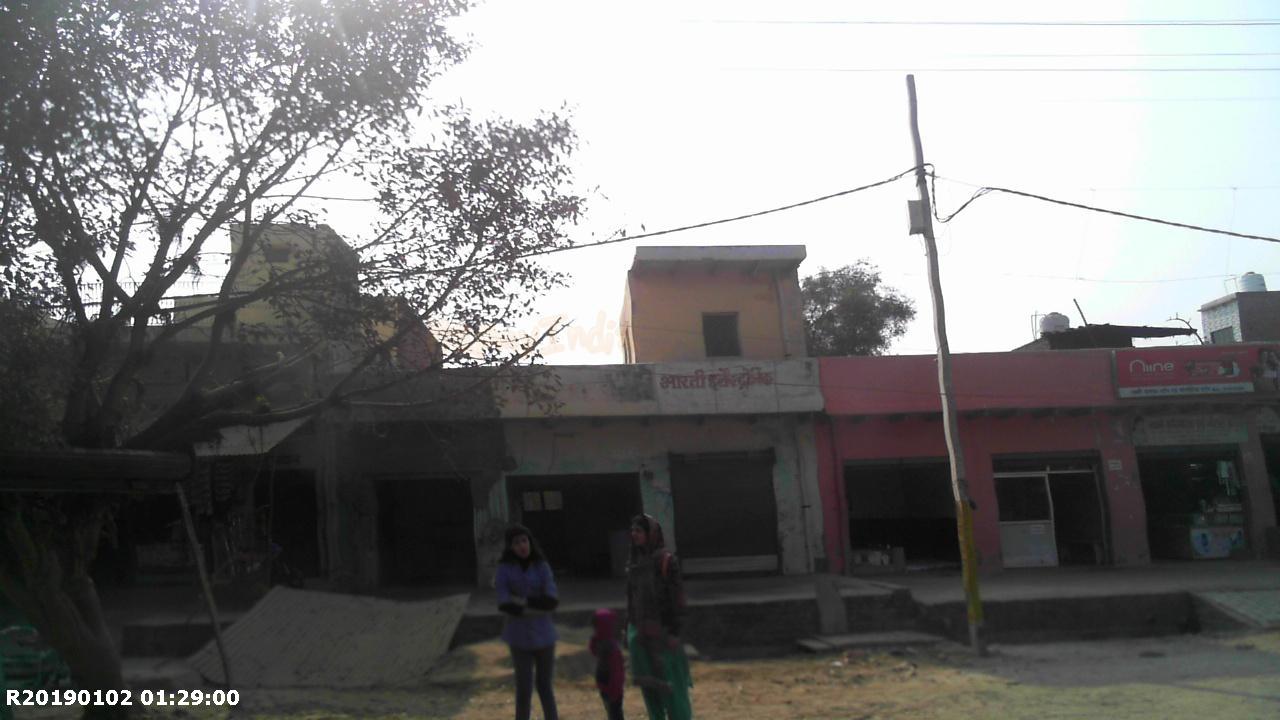

Supplement: Supplementary file 2 — Supplementary Material 2 [file 41598_2026_40742_MOESM2_ESM.zip › sample_data_yolov5/R_01-02_01.29.00.jpg]

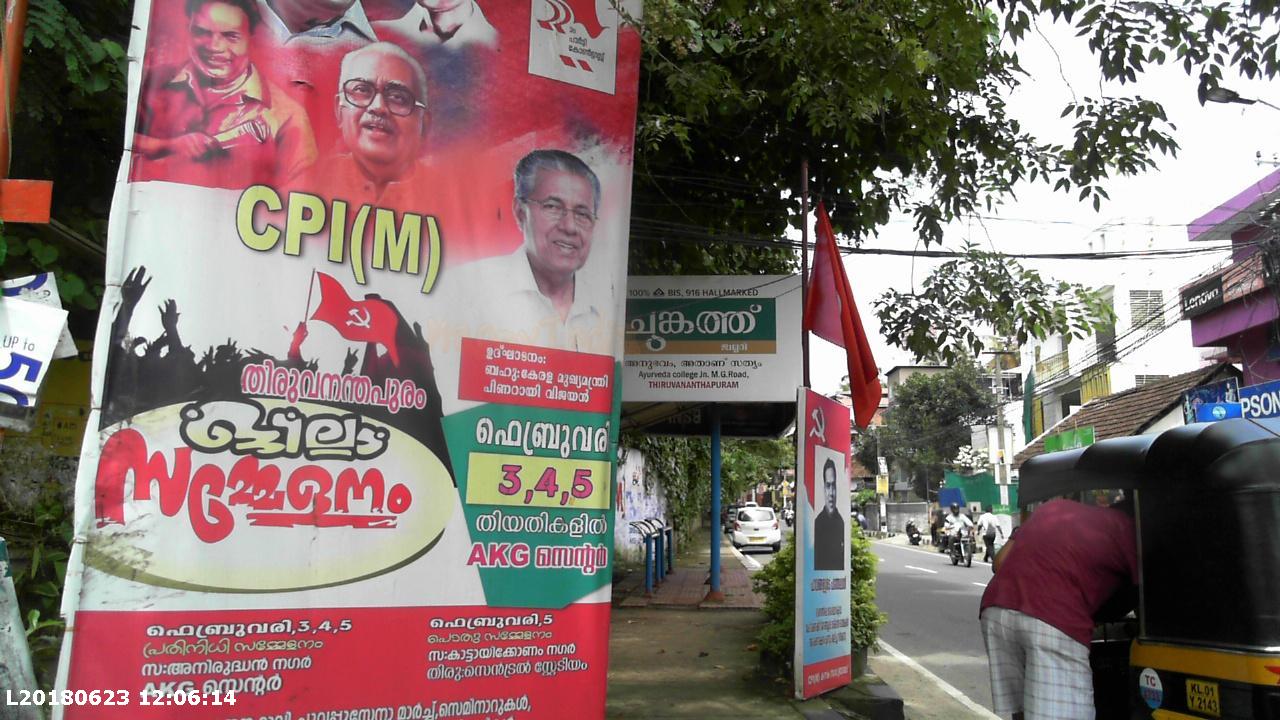

Supplement: Supplementary file 2 — Supplementary Material 2 [file 41598_2026_40742_MOESM2_ESM.zip › sample_data_yolov5/L_06-23_12.06.14.jpg]

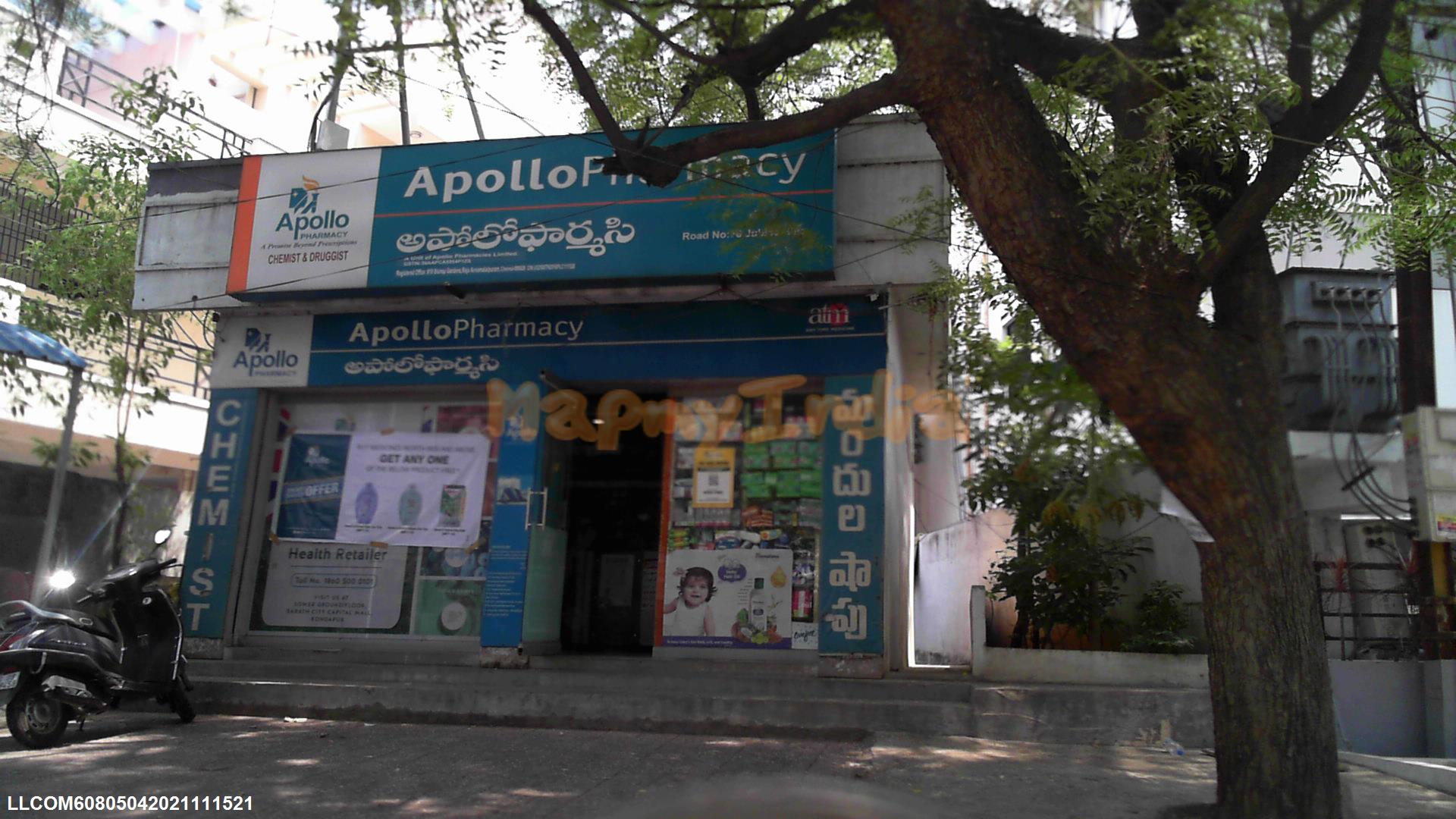

Supplement: Supplementary file 2 — Supplementary Material 2 [file 41598_2026_40742_MOESM2_ESM.zip › sample_data_yolov5/LCOM60805042021111521.jpg]

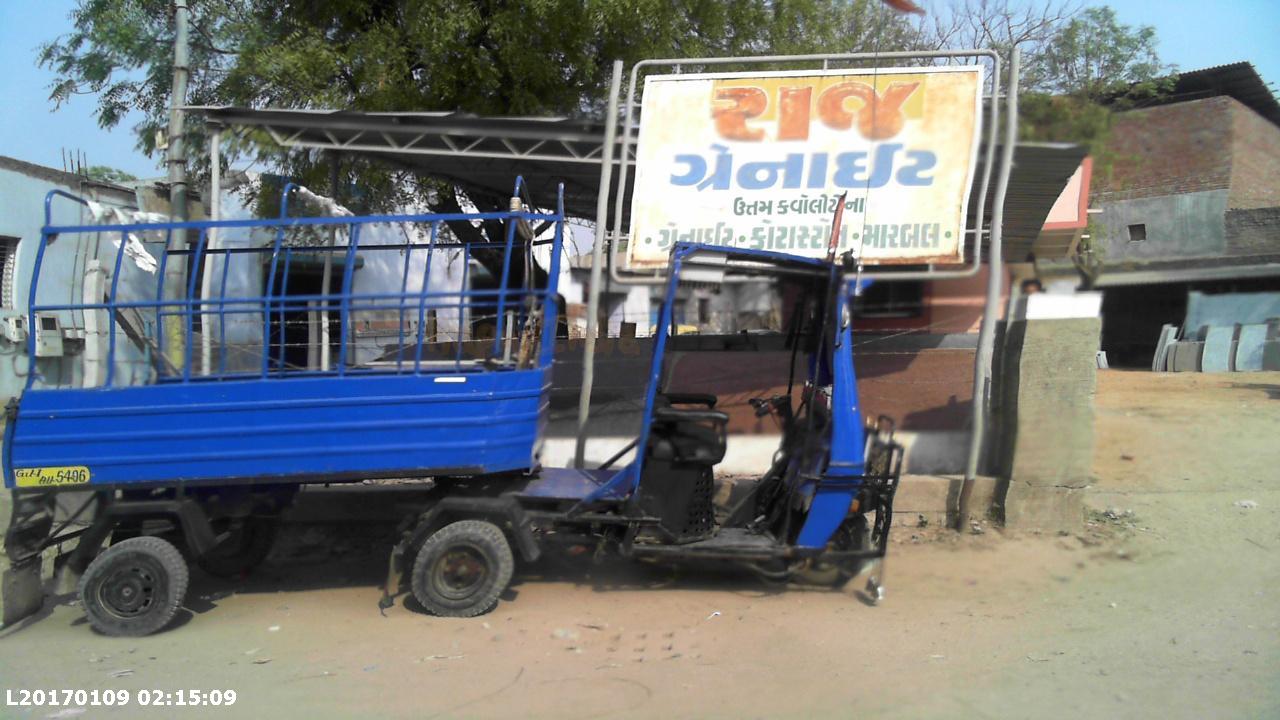

Supplement: Supplementary file 2 — Supplementary Material 2 [file 41598_2026_40742_MOESM2_ESM.zip › sample_data_yolov5/01-09 02.15.09.jpg]

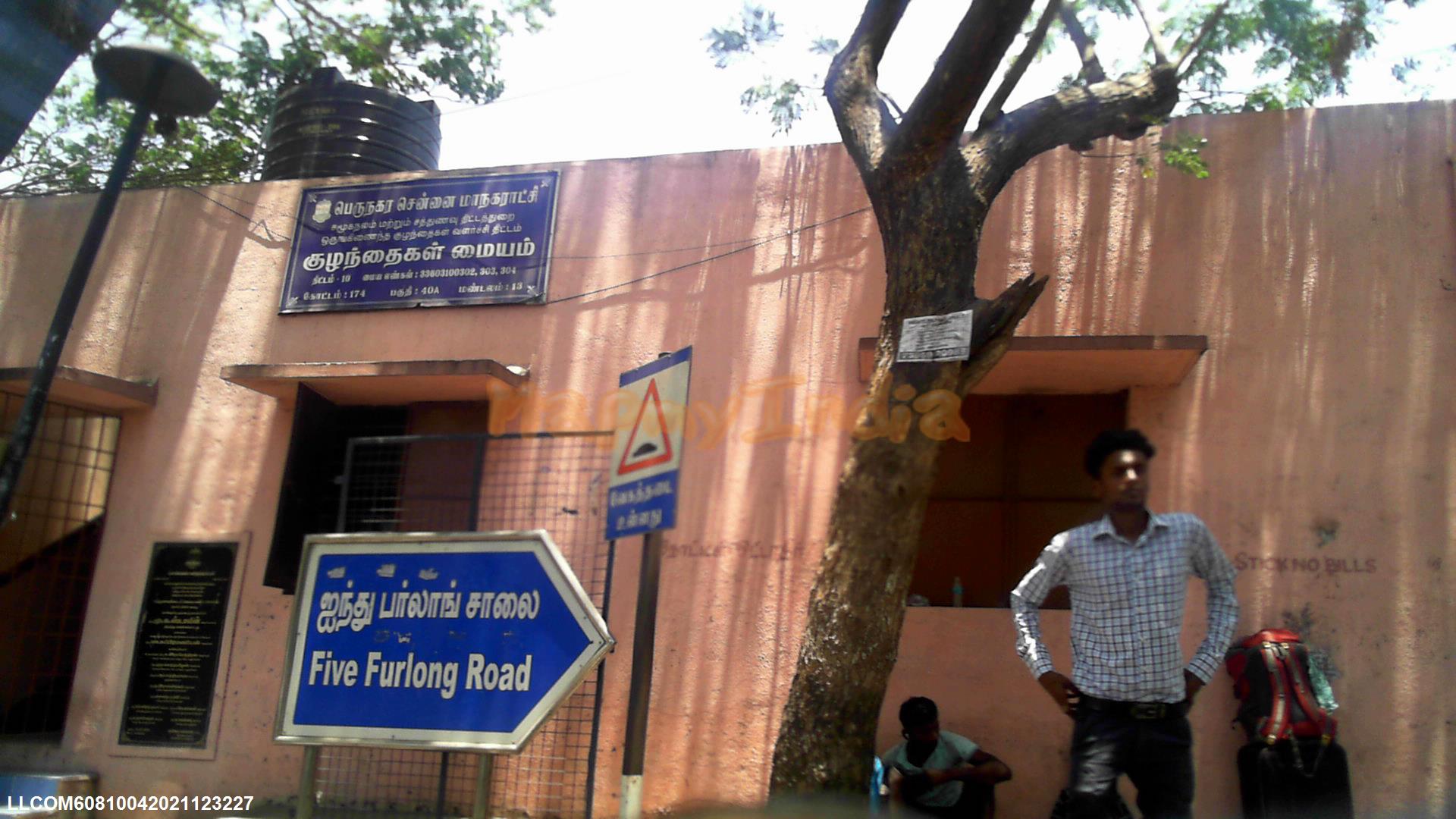

Supplement: Supplementary file 2 — Supplementary Material 2 [file 41598_2026_40742_MOESM2_ESM.zip › sample_data_yolov5/LCOM60810042021123227.jpg]

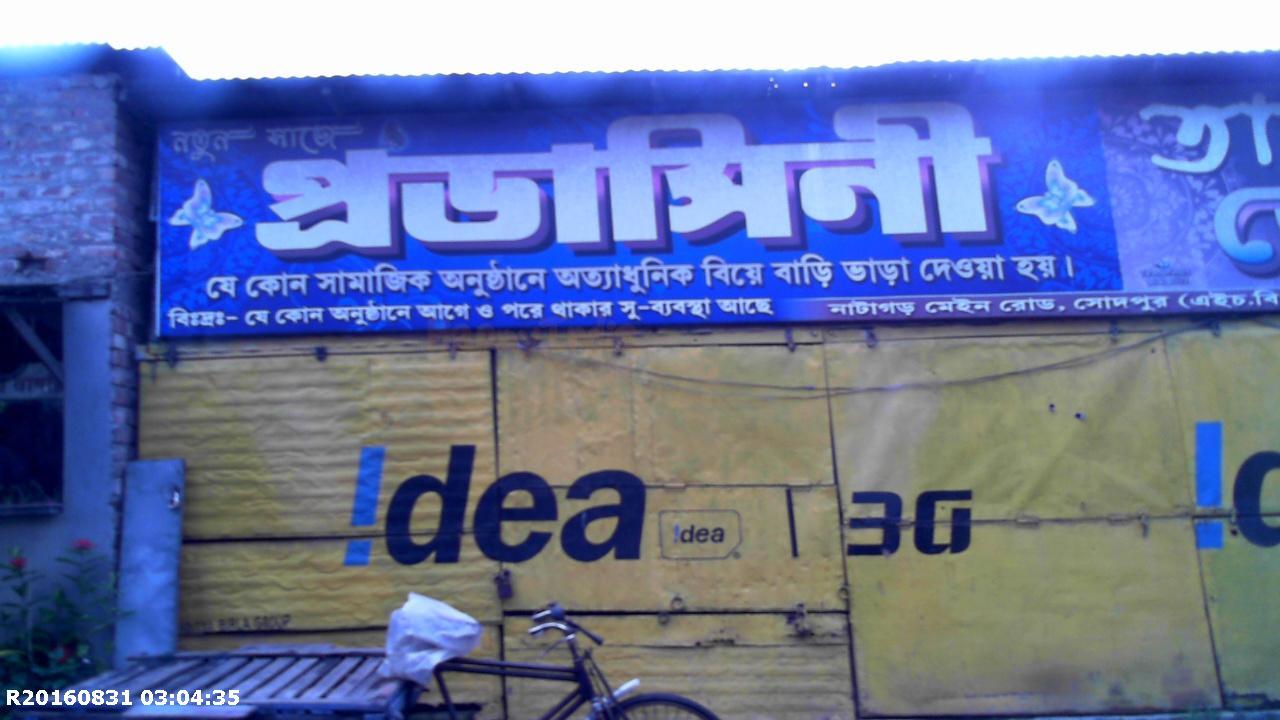

Supplement: Supplementary file 2 — Supplementary Material 2 [file 41598_2026_40742_MOESM2_ESM.zip › sample_data_yolov5/R_08-31_03.04.35.jpg]

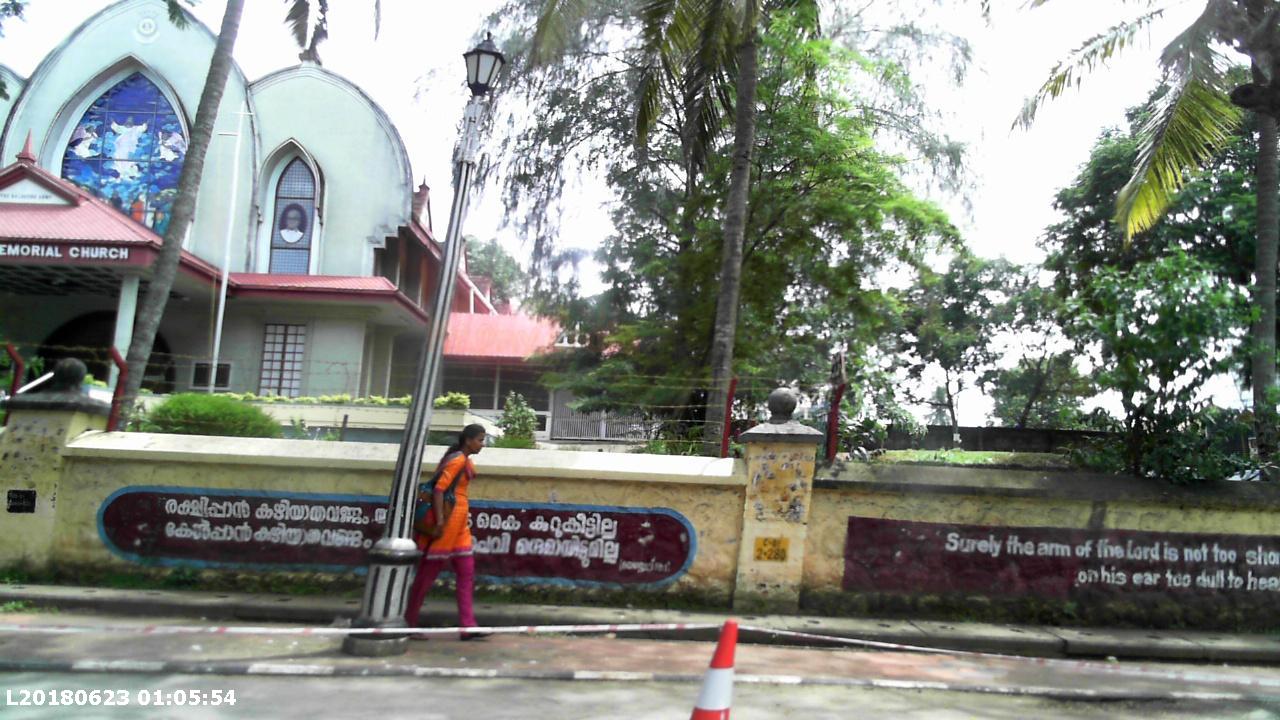

Supplement: Supplementary file 2 — Supplementary Material 2 [file 41598_2026_40742_MOESM2_ESM.zip › sample_data_yolov5/L_06-23_01.05.54.jpg]

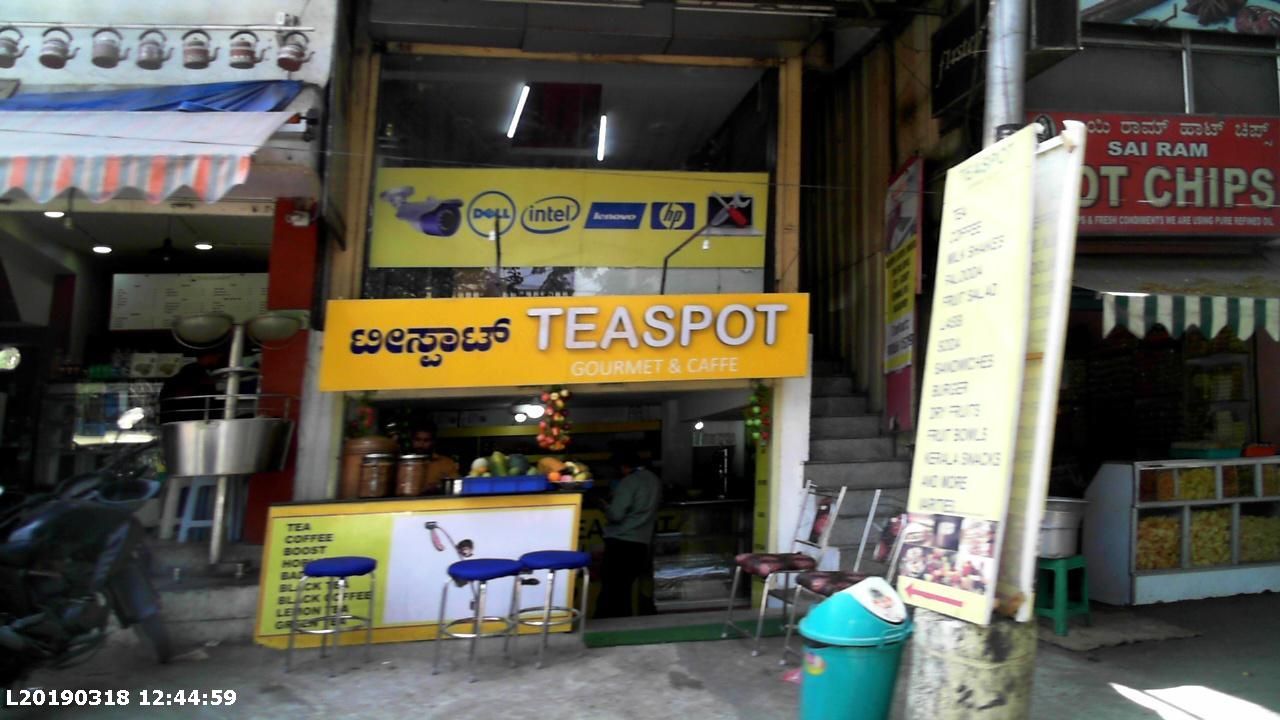

Supplement: Supplementary file 2 — Supplementary Material 2 [file 41598_2026_40742_MOESM2_ESM.zip › sample_data_yolov5/03-18_12.44.59.jpg]

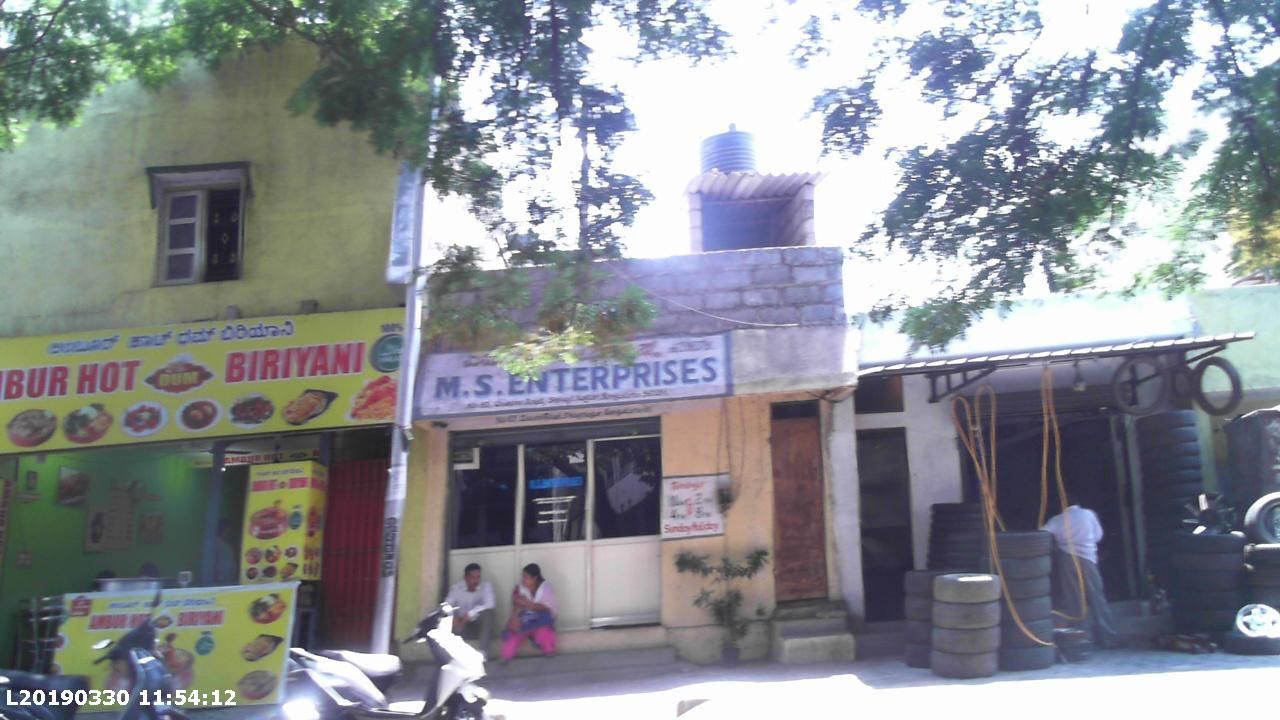

Supplement: Supplementary file 2 — Supplementary Material 2 [file 41598_2026_40742_MOESM2_ESM.zip › sample_data_yolov5/03-30_11.54.12.jpg]

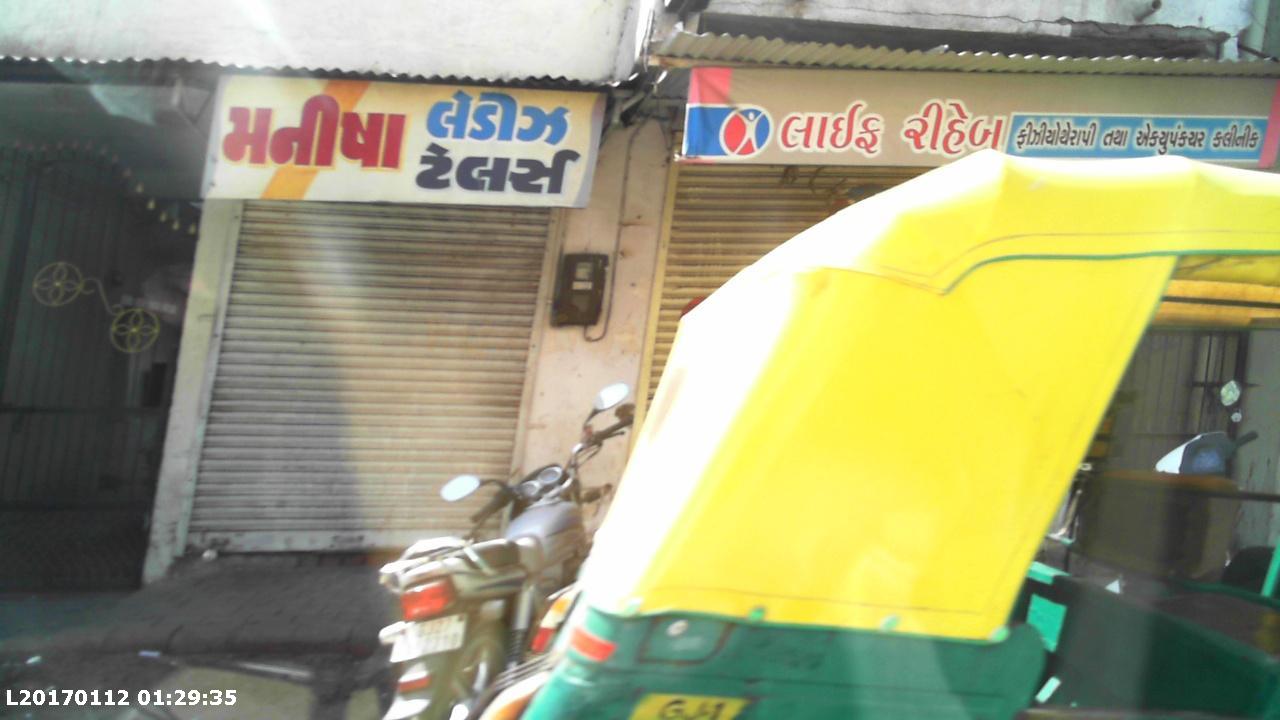

Supplement: Supplementary file 2 — Supplementary Material 2 [file 41598_2026_40742_MOESM2_ESM.zip › sample_data_yolov5/01-12 01.29.35.jpg]

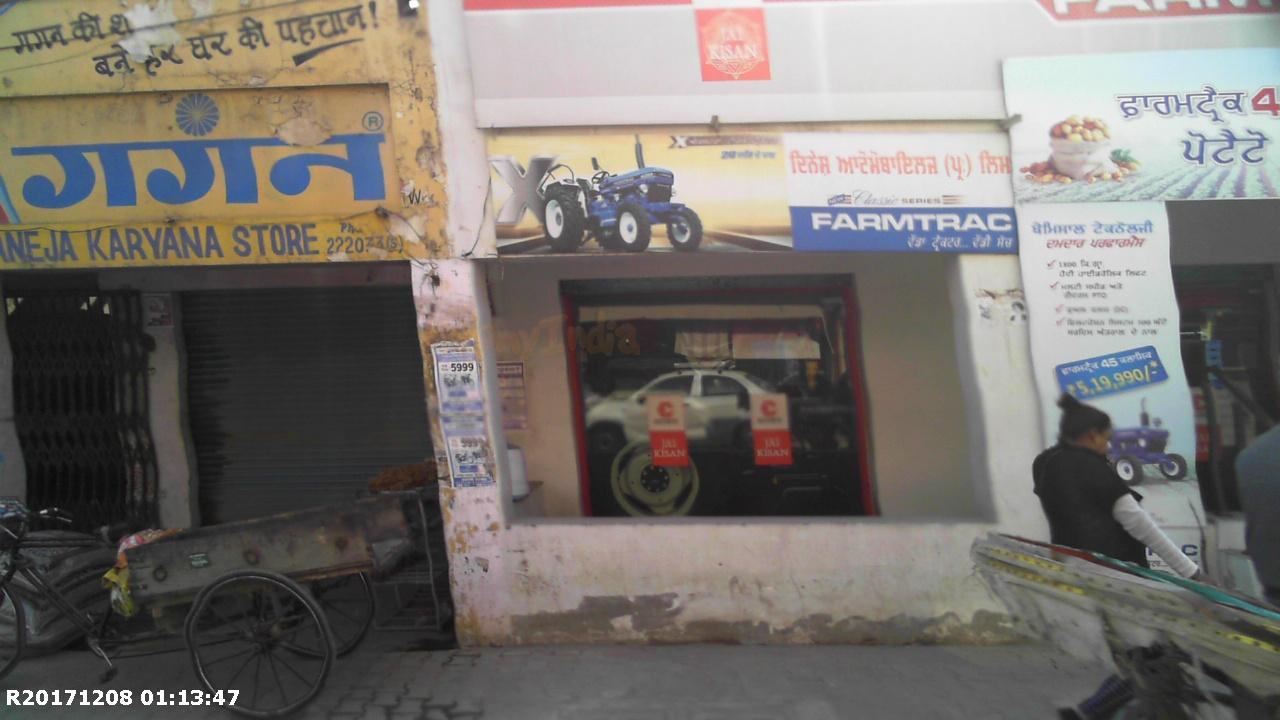

Supplement: Supplementary file 2 — Supplementary Material 2 [file 41598_2026_40742_MOESM2_ESM.zip › sample_data_yolov5/12-08 01.13.47.jpg]

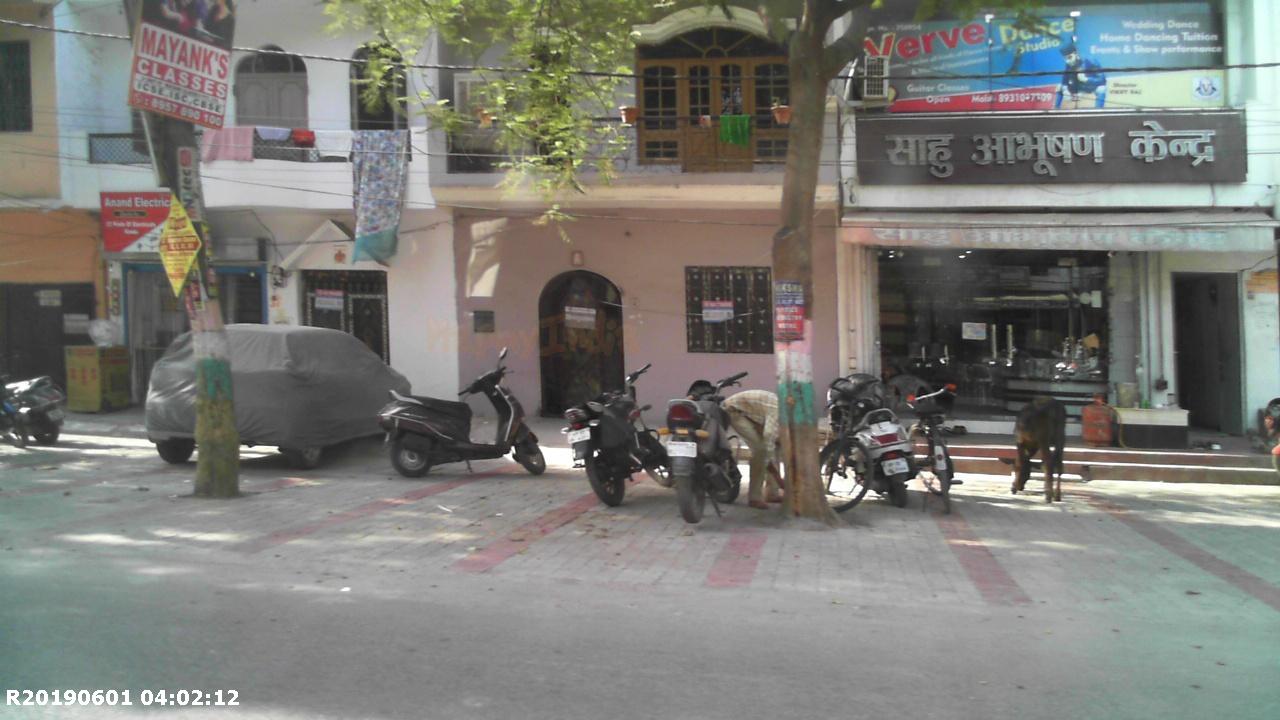

Supplement: Supplementary file 2 — Supplementary Material 2 [file 41598_2026_40742_MOESM2_ESM.zip › sample_data_yolov5/R_06-01_04.02.12.jpg]

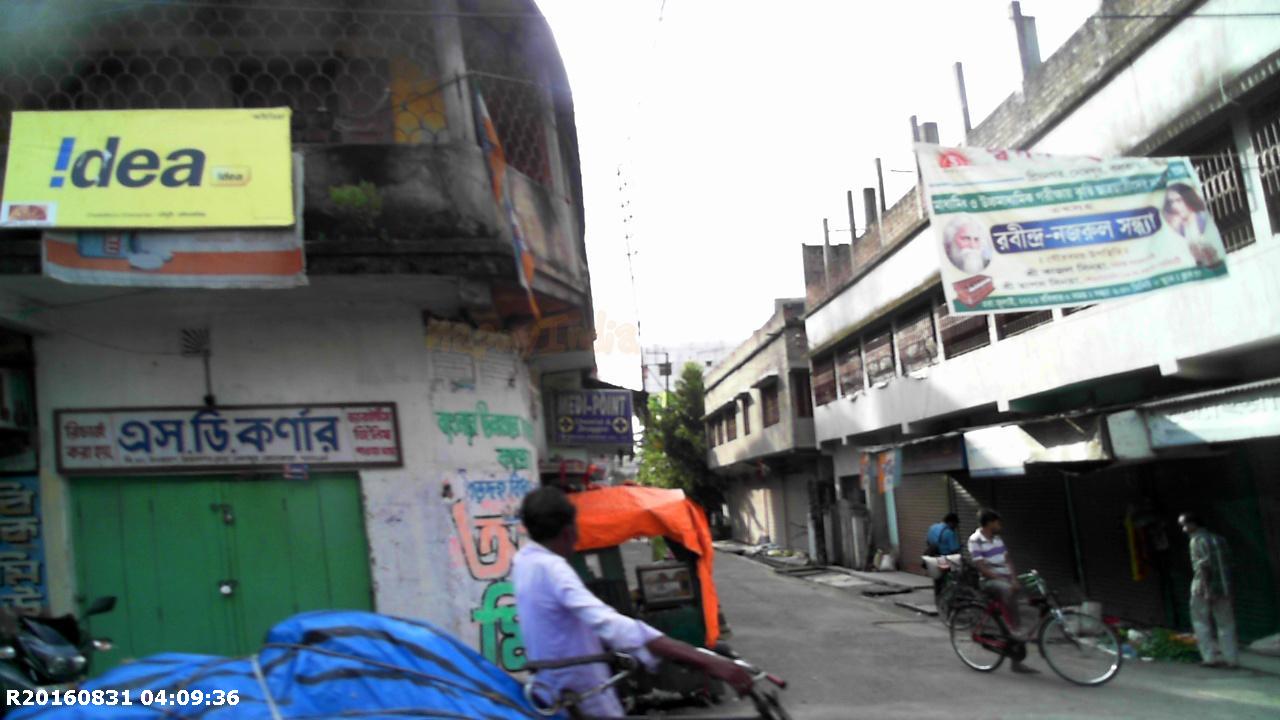

Supplement: Supplementary file 2 — Supplementary Material 2 [file 41598_2026_40742_MOESM2_ESM.zip › sample_data_yolov5/R_08-31_04.09.36.jpg]

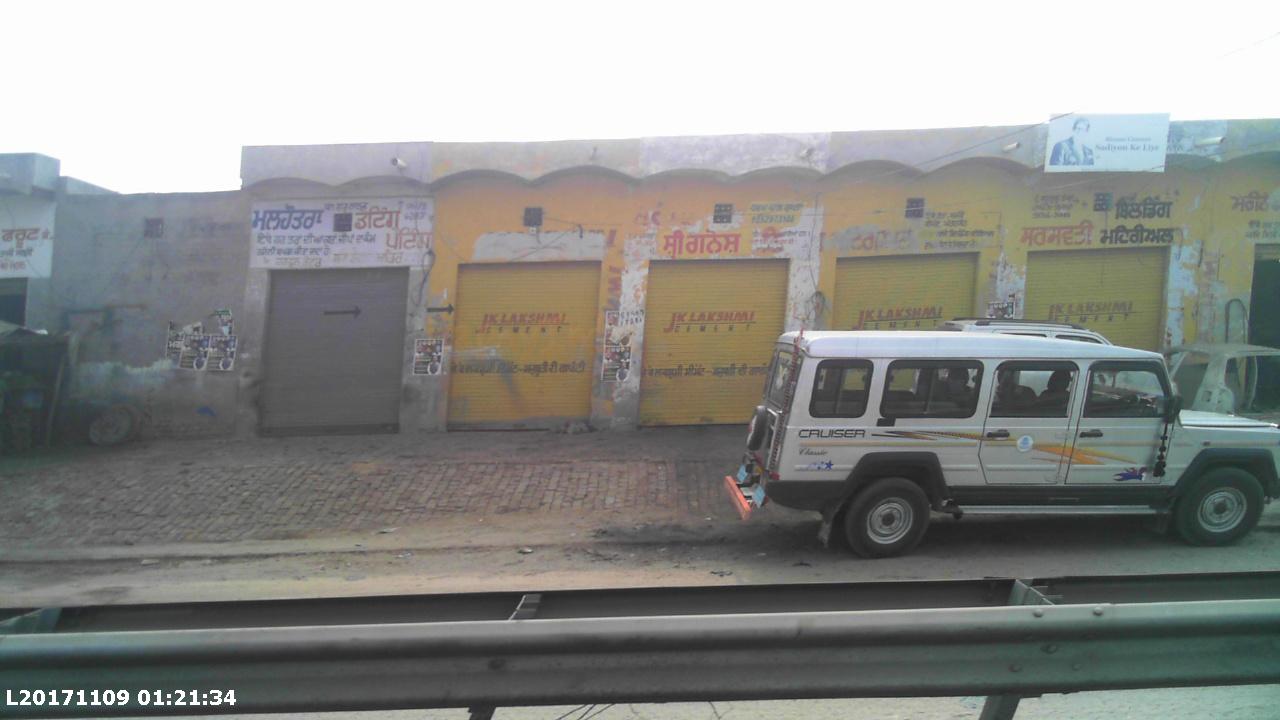

Supplement: Supplementary file 2 — Supplementary Material 2 [file 41598_2026_40742_MOESM2_ESM.zip › sample_data_yolov5/11-09 01.21.34.jpg]

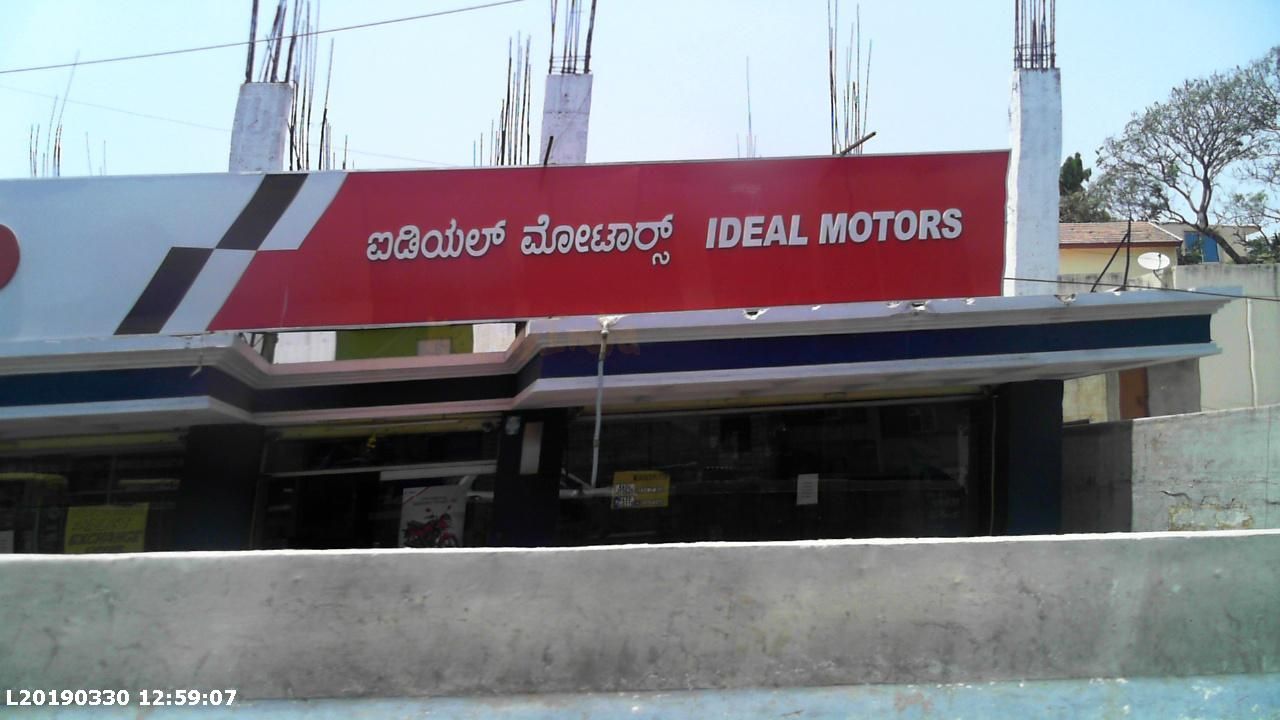

Supplement: Supplementary file 2 — Supplementary Material 2 [file 41598_2026_40742_MOESM2_ESM.zip › sample_data_yolov5/03-30_12.59.07.jpg]

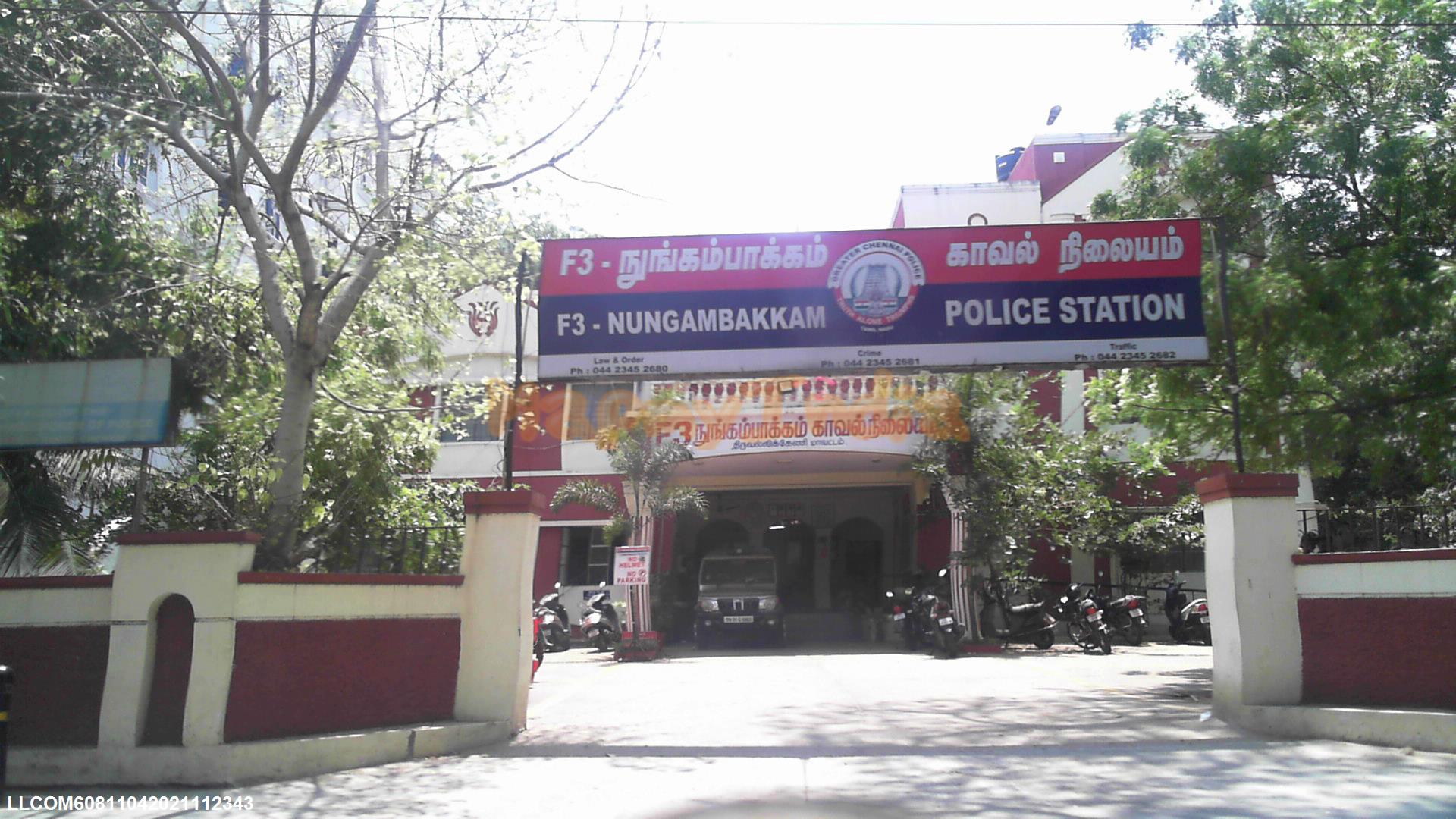

Supplement: Supplementary file 2 — Supplementary Material 2 [file 41598_2026_40742_MOESM2_ESM.zip › sample_data_yolov5/LCOM60811042021112343.jpg]

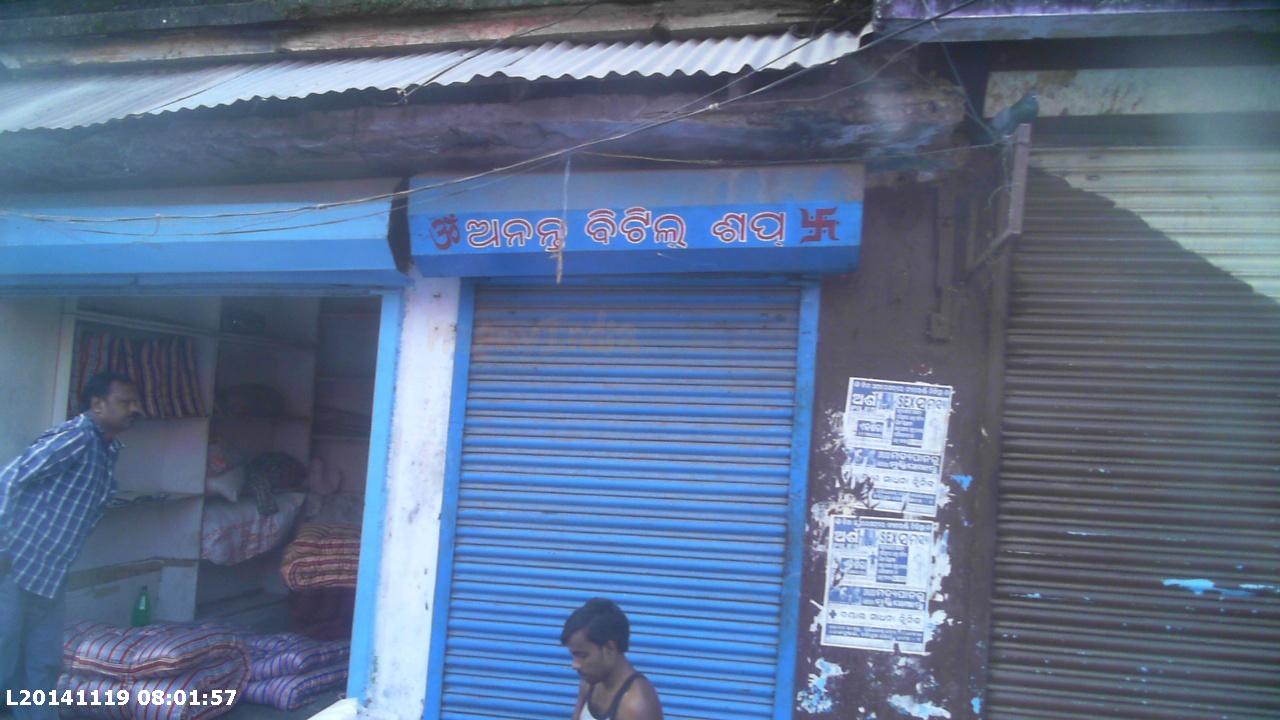

Supplement: Supplementary file 2 — Supplementary Material 2 [file 41598_2026_40742_MOESM2_ESM.zip › sample_data_yolov5/L_11-19_08.01.57.jpg]

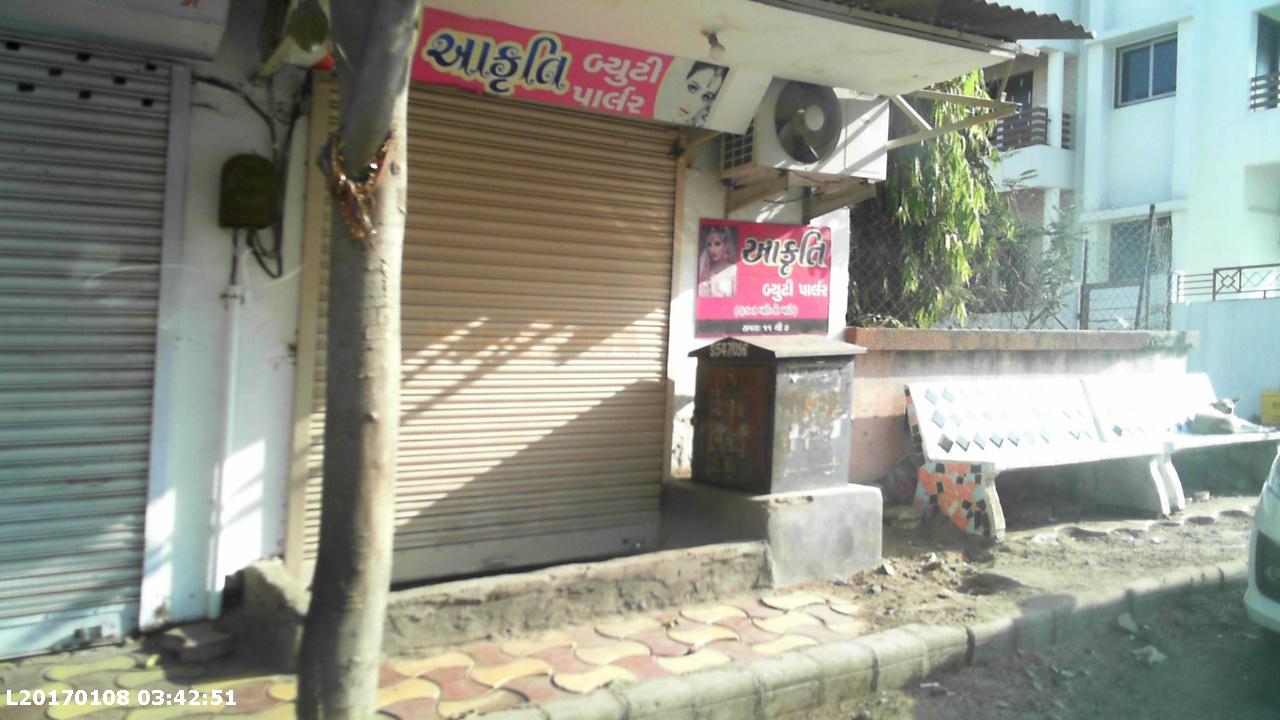

Supplement: Supplementary file 2 — Supplementary Material 2 [file 41598_2026_40742_MOESM2_ESM.zip › sample_data_yolov5/01-08 03.42.51.jpg]

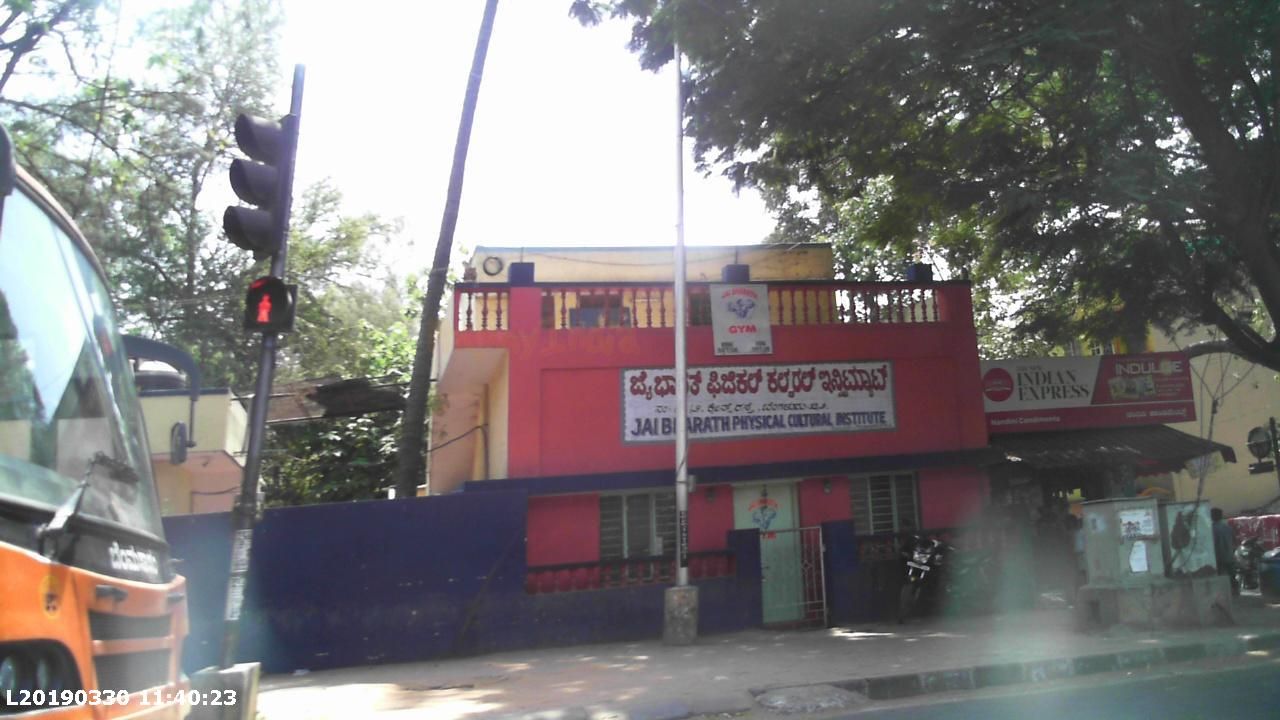

Supplement: Supplementary file 2 — Supplementary Material 2 [file 41598_2026_40742_MOESM2_ESM.zip › sample_data_yolov5/03-30_11.40.23.jpg]

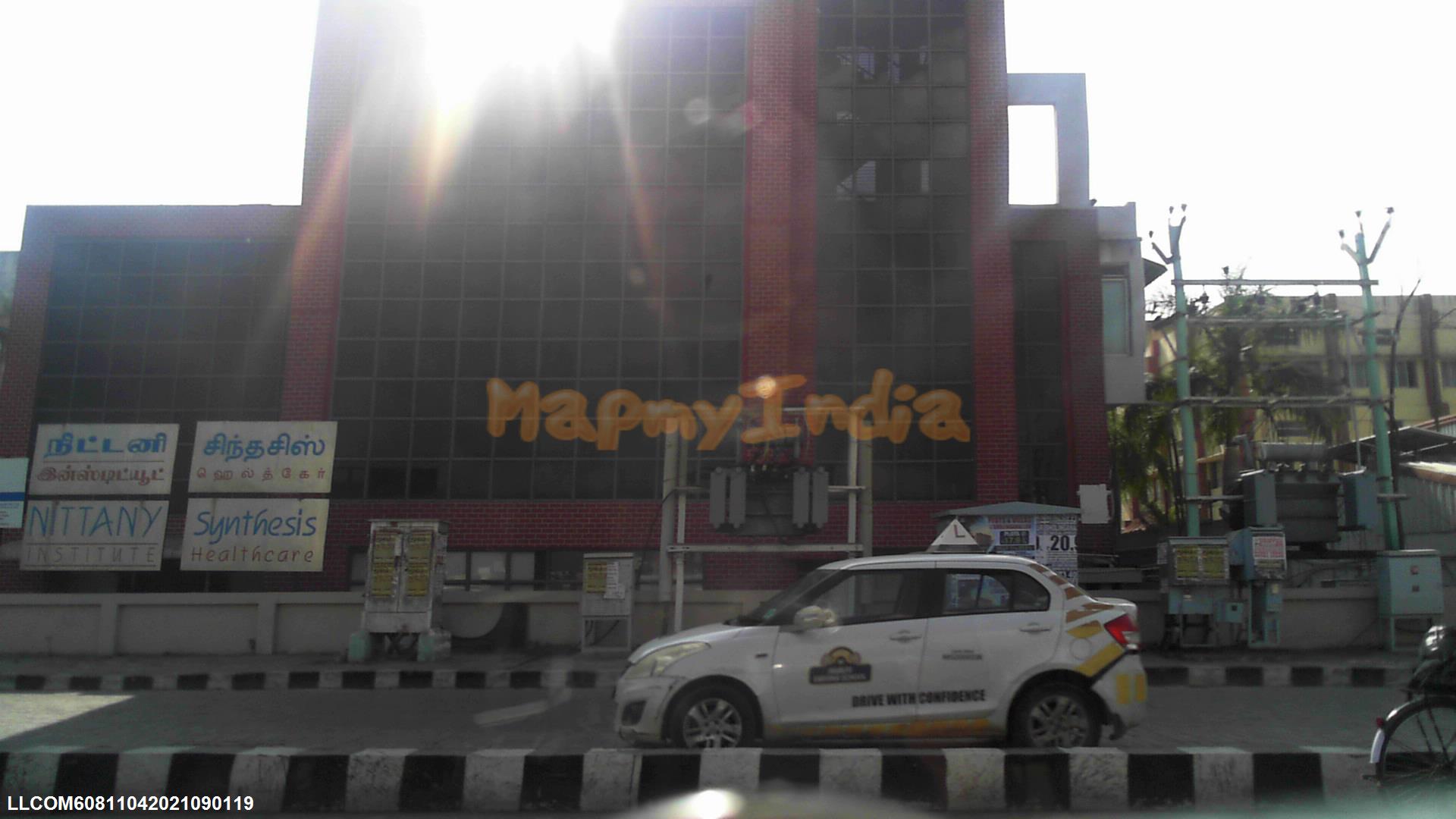

Supplement: Supplementary file 2 — Supplementary Material 2 [file 41598_2026_40742_MOESM2_ESM.zip › sample_data_yolov5/LCOM60811042021090119.jpg]

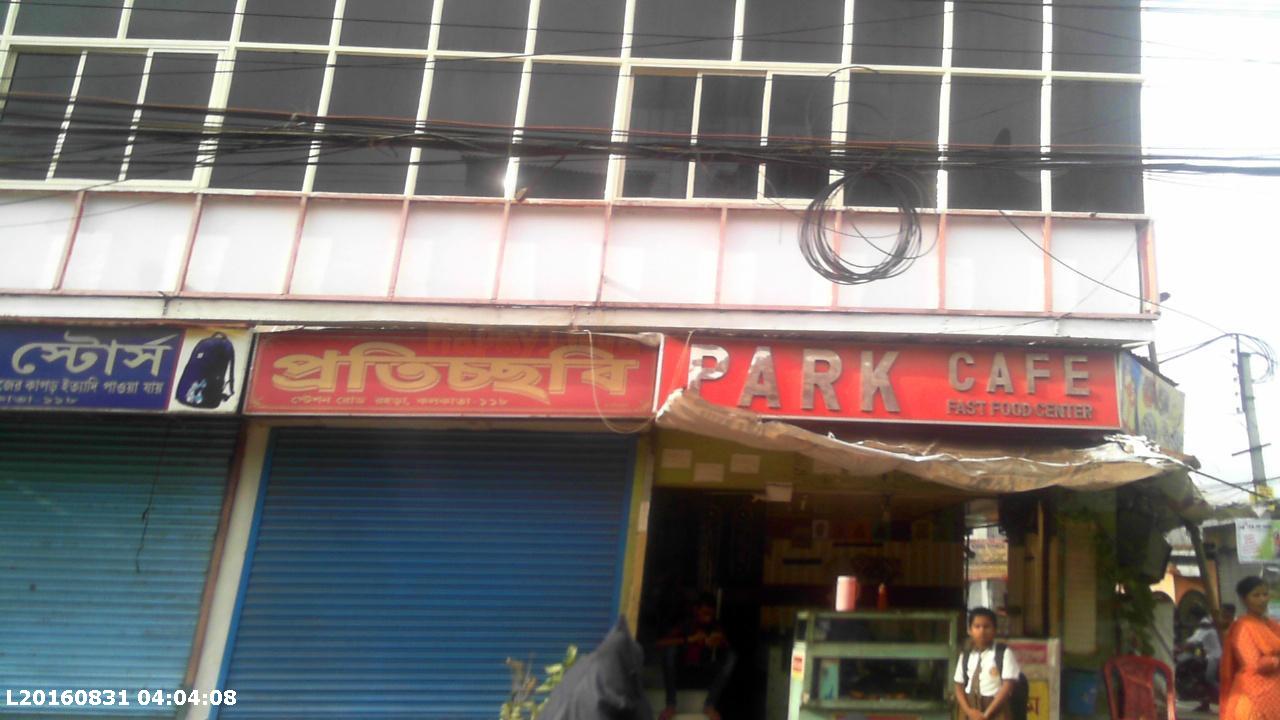

Supplement: Supplementary file 2 — Supplementary Material 2 [file 41598_2026_40742_MOESM2_ESM.zip › sample_data_yolov5/L_08-31_04.04.08.jpg]

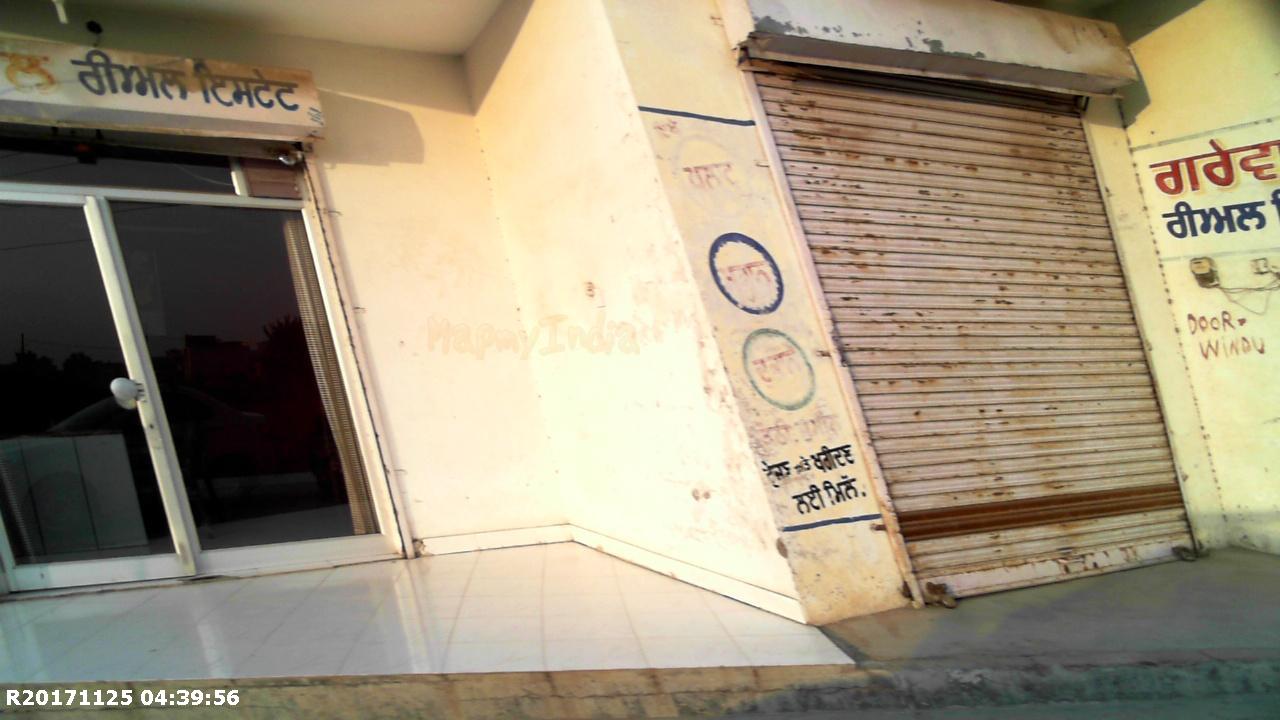

Supplement: Supplementary file 2 — Supplementary Material 2 [file 41598_2026_40742_MOESM2_ESM.zip › sample_data_yolov5/11-25 04.39.56.jpg]

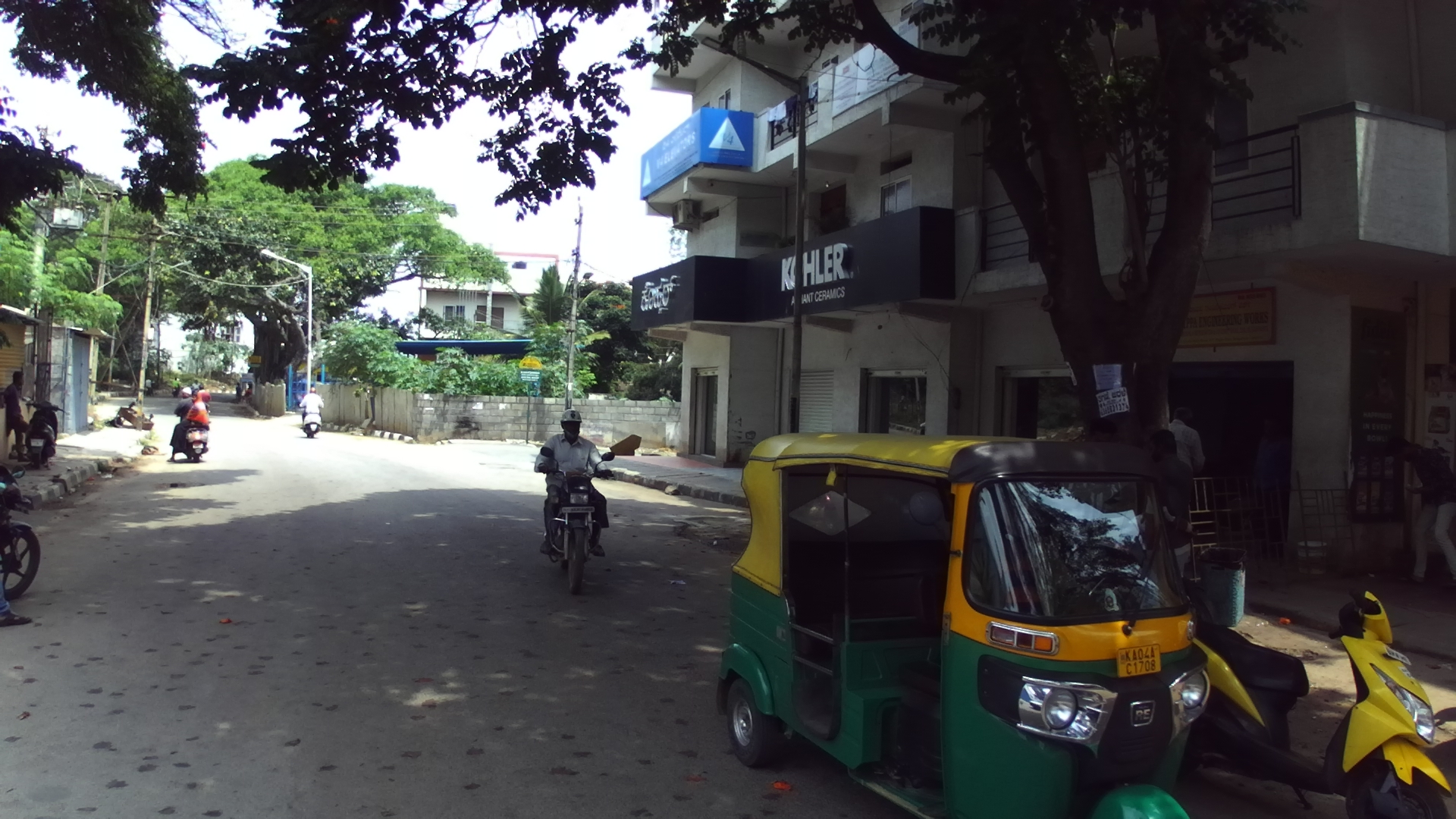

Supplement: Supplementary file 2 — Supplementary Material 2 [file 41598_2026_40742_MOESM2_ESM.zip › sample_data_yolov5/T1_051020_114319_24376_zed_l_407.jpg]

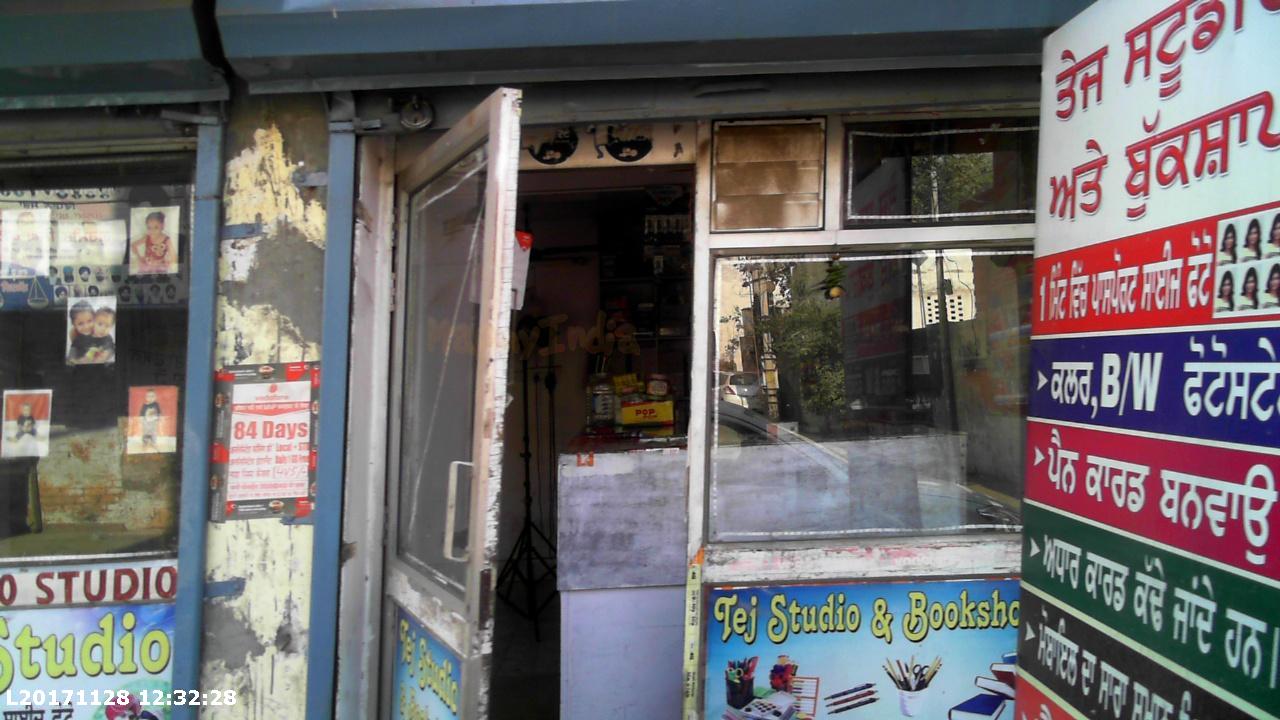

Supplement: Supplementary file 2 — Supplementary Material 2 [file 41598_2026_40742_MOESM2_ESM.zip › sample_data_yolov5/11-28 12.32.28.jpg]

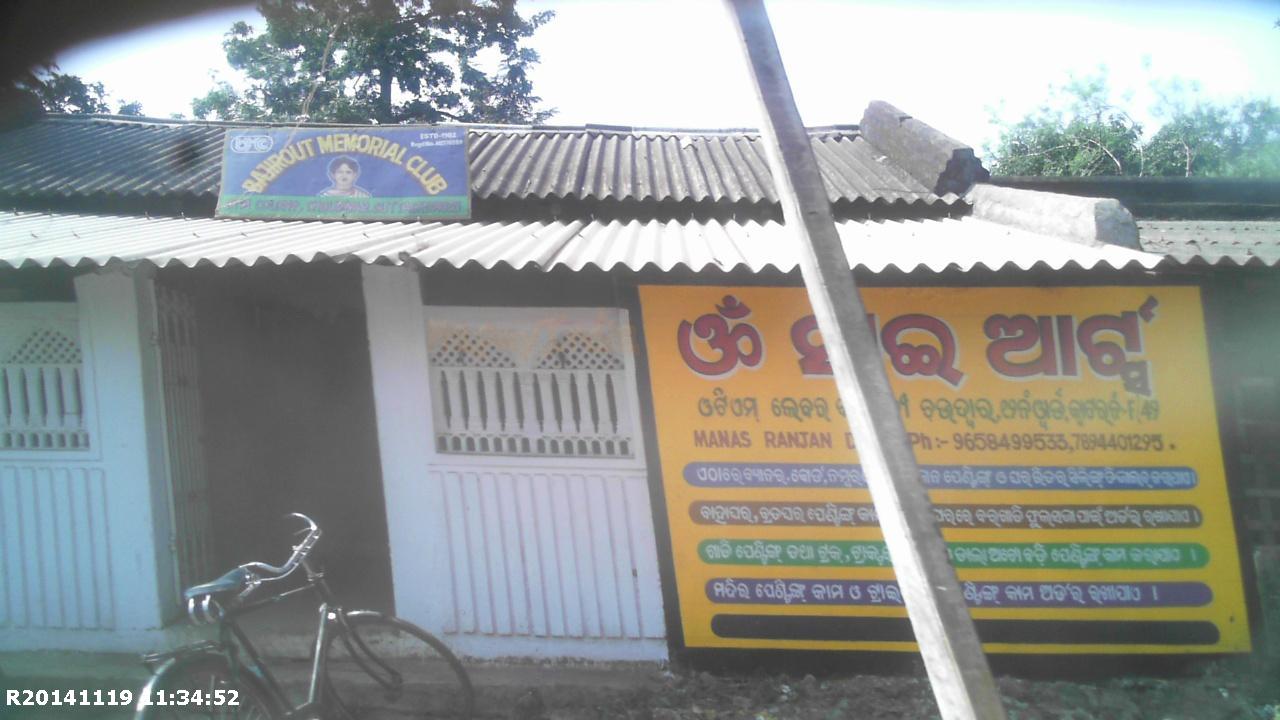

Supplement: Supplementary file 2 — Supplementary Material 2 [file 41598_2026_40742_MOESM2_ESM.zip › sample_data_yolov5/R_11-19_11.34.52.jpg]

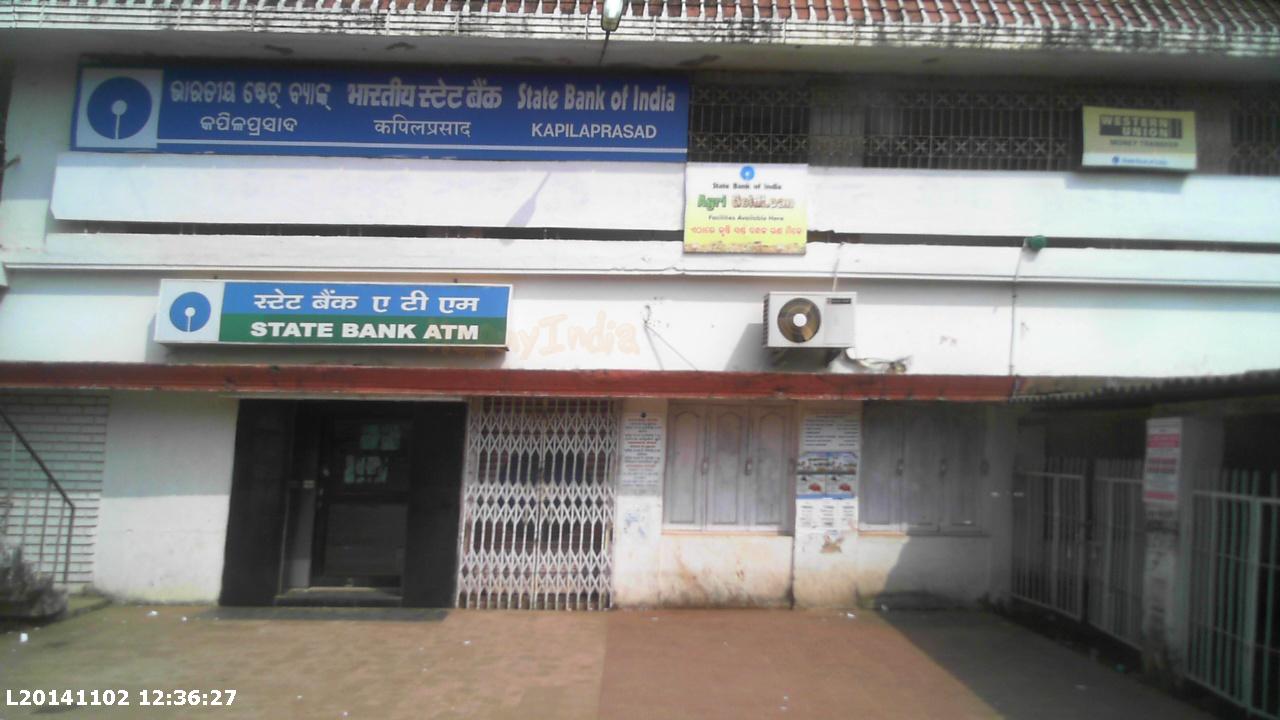

Supplement: Supplementary file 2 — Supplementary Material 2 [file 41598_2026_40742_MOESM2_ESM.zip › sample_data_yolov5/L_11-02_12.36.27.jpg]

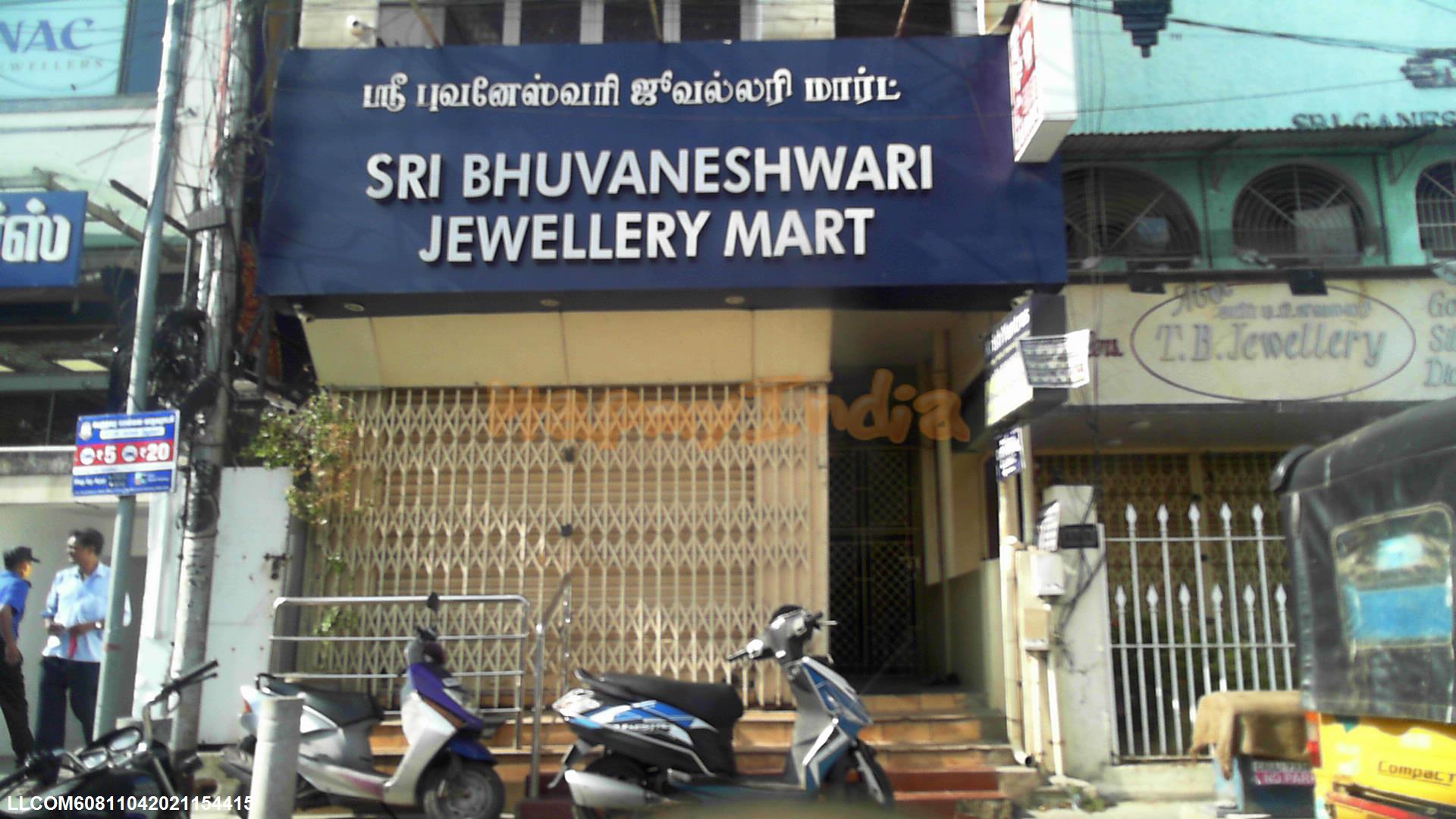

Supplement: Supplementary file 2 — Supplementary Material 2 [file 41598_2026_40742_MOESM2_ESM.zip › sample_data_yolov5/LCOM60811042021154415.jpg]

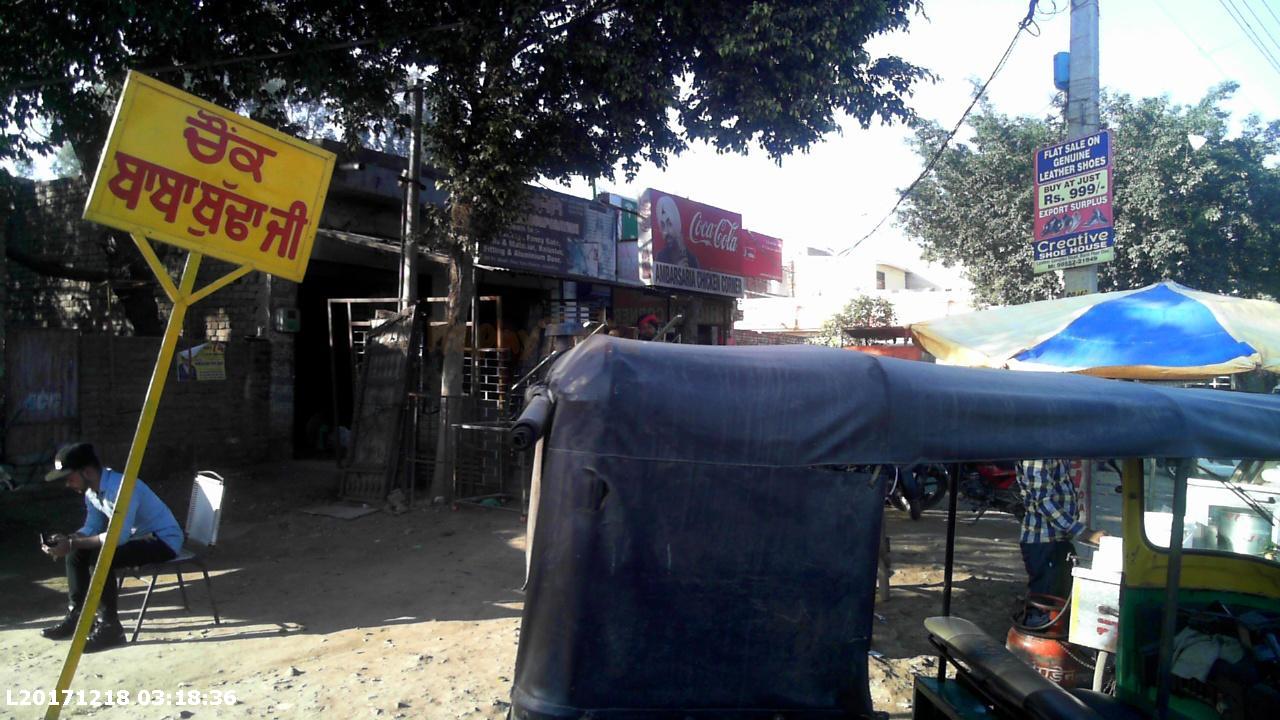

Supplement: Supplementary file 2 — Supplementary Material 2 [file 41598_2026_40742_MOESM2_ESM.zip › sample_data_yolov5/12-18 03.18.36.jpg]

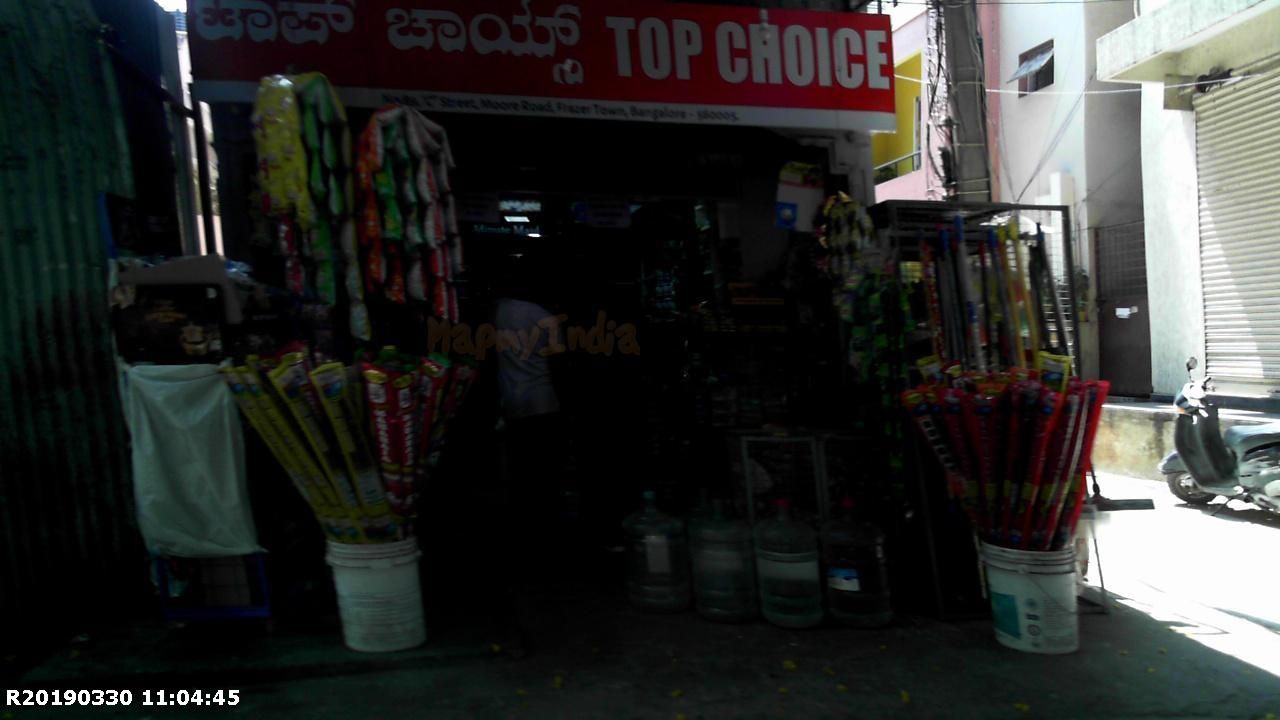

Supplement: Supplementary file 2 — Supplementary Material 2 [file 41598_2026_40742_MOESM2_ESM.zip › sample_data_yolov5/03-30_11.04.45.jpg]

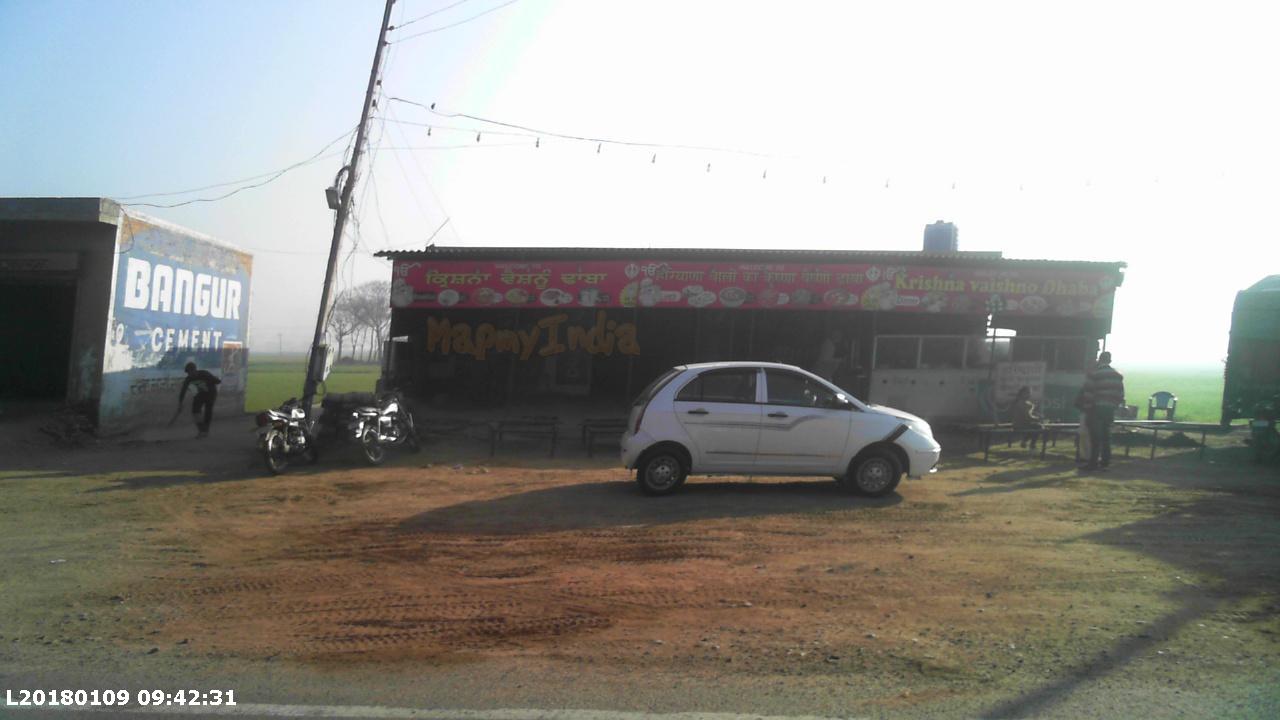

Supplement: Supplementary file 2 — Supplementary Material 2 [file 41598_2026_40742_MOESM2_ESM.zip › sample_data_yolov5/01-09 09.42.31.jpg]

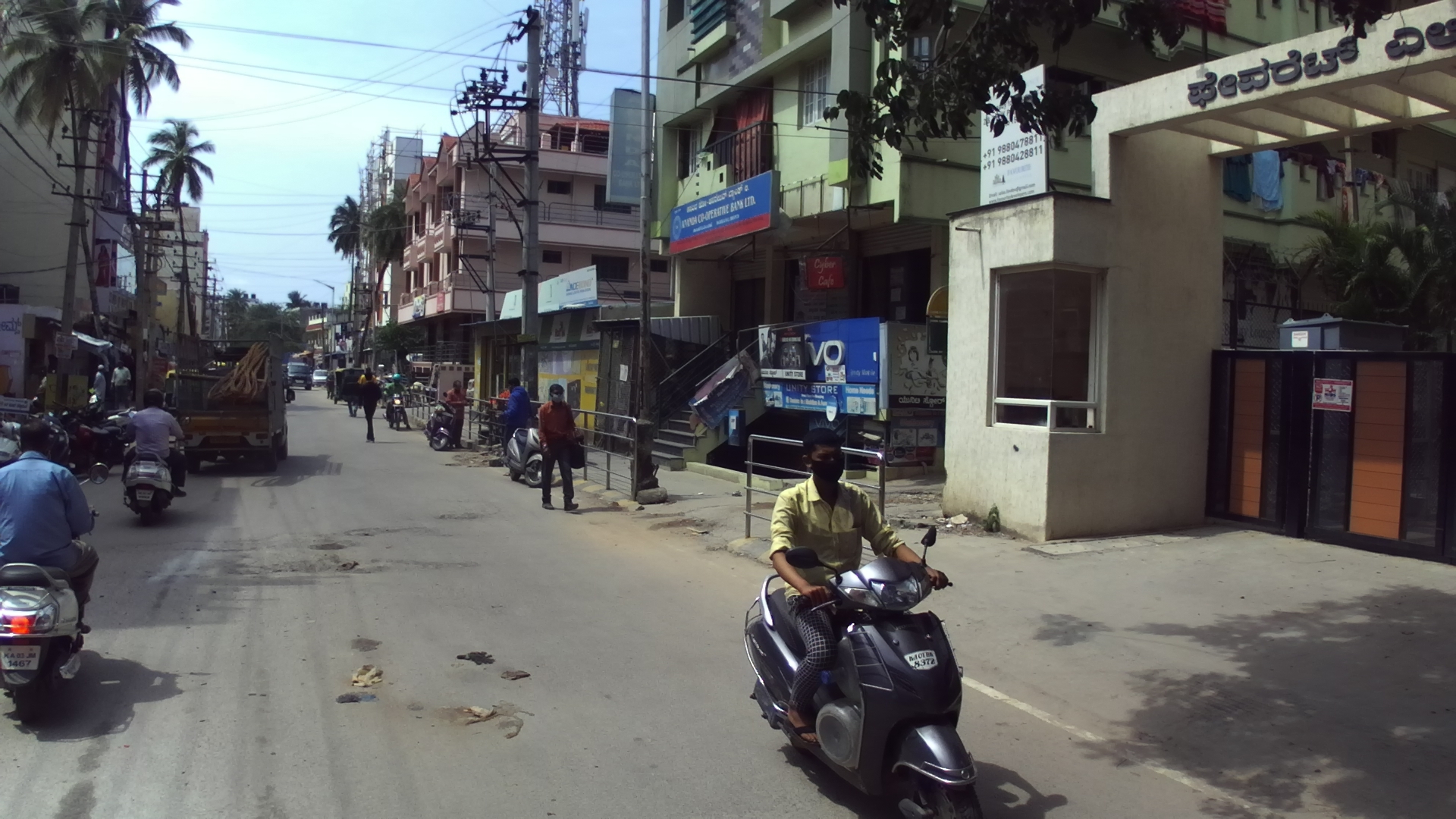

Supplement: Supplementary file 2 — Supplementary Material 2 [file 41598_2026_40742_MOESM2_ESM.zip › sample_data_yolov5/T1_051020_104353_24376_zed_l_760.jpg]

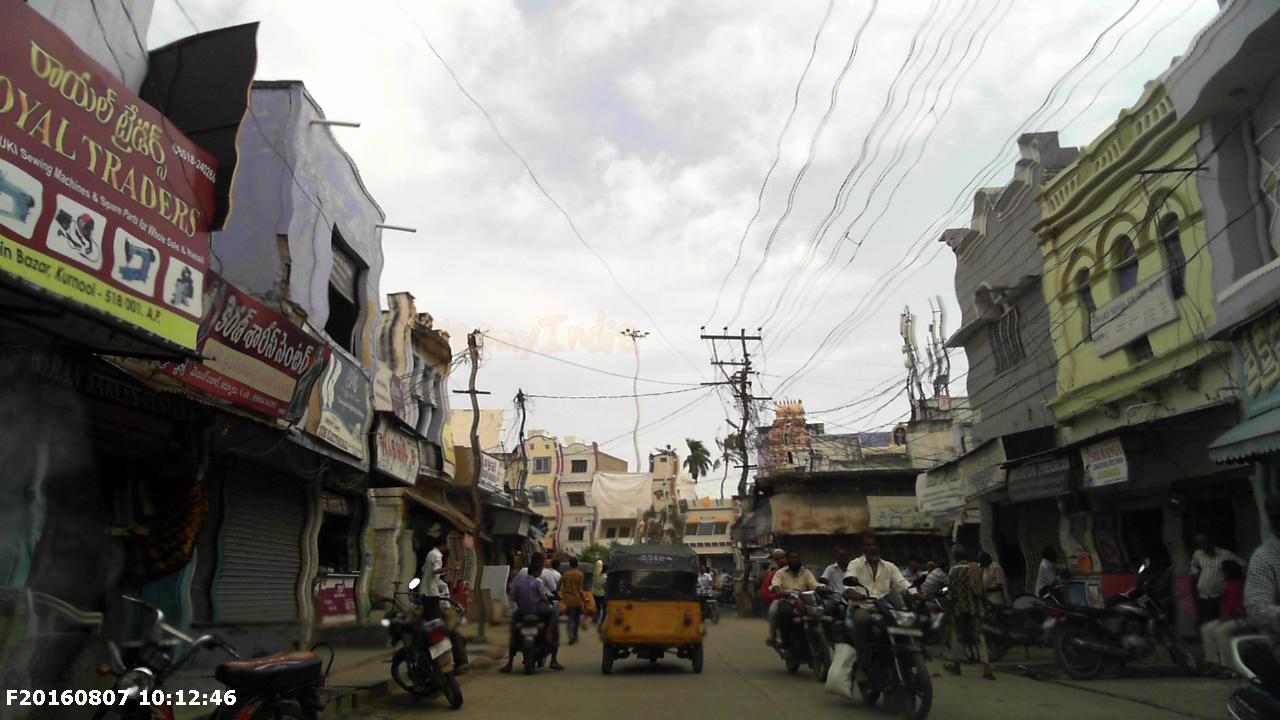

Supplement: Supplementary file 2 — Supplementary Material 2 [file 41598_2026_40742_MOESM2_ESM.zip › sample_data_yolov5/08-07 10.12.46.jpg]

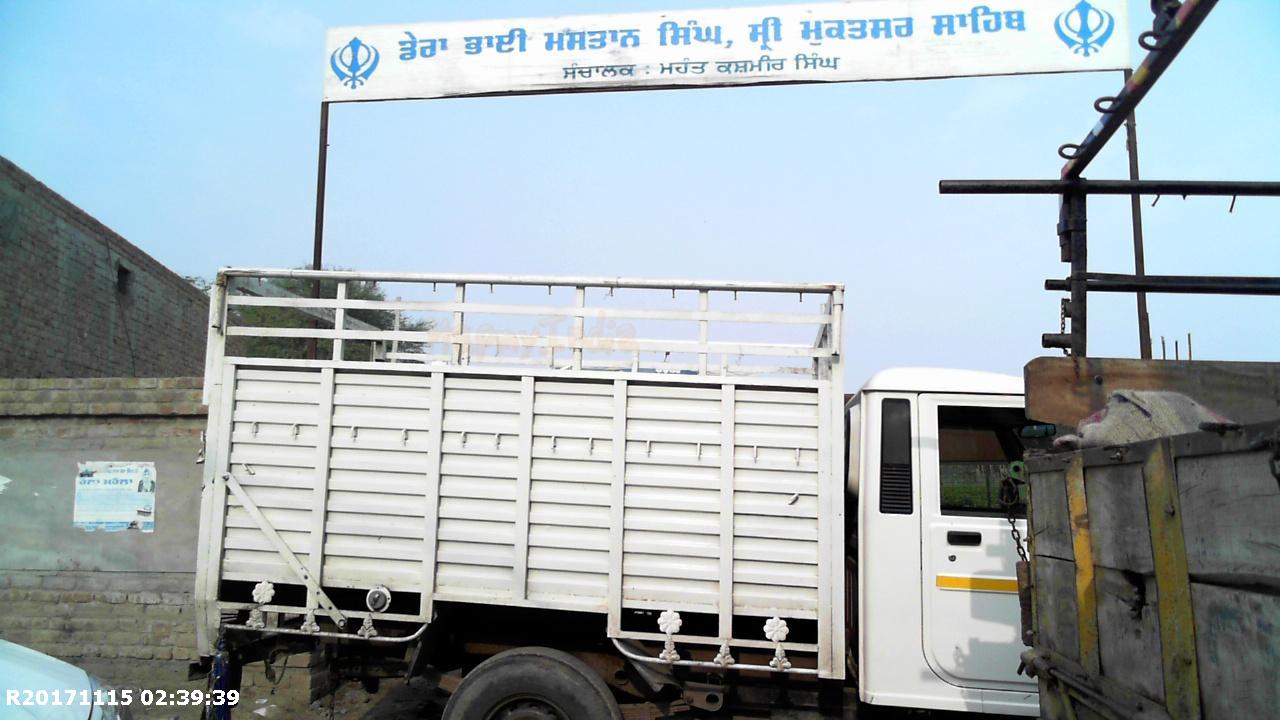

Supplement: Supplementary file 2 — Supplementary Material 2 [file 41598_2026_40742_MOESM2_ESM.zip › sample_data_yolov5/11-15 02.39.39.jpg]

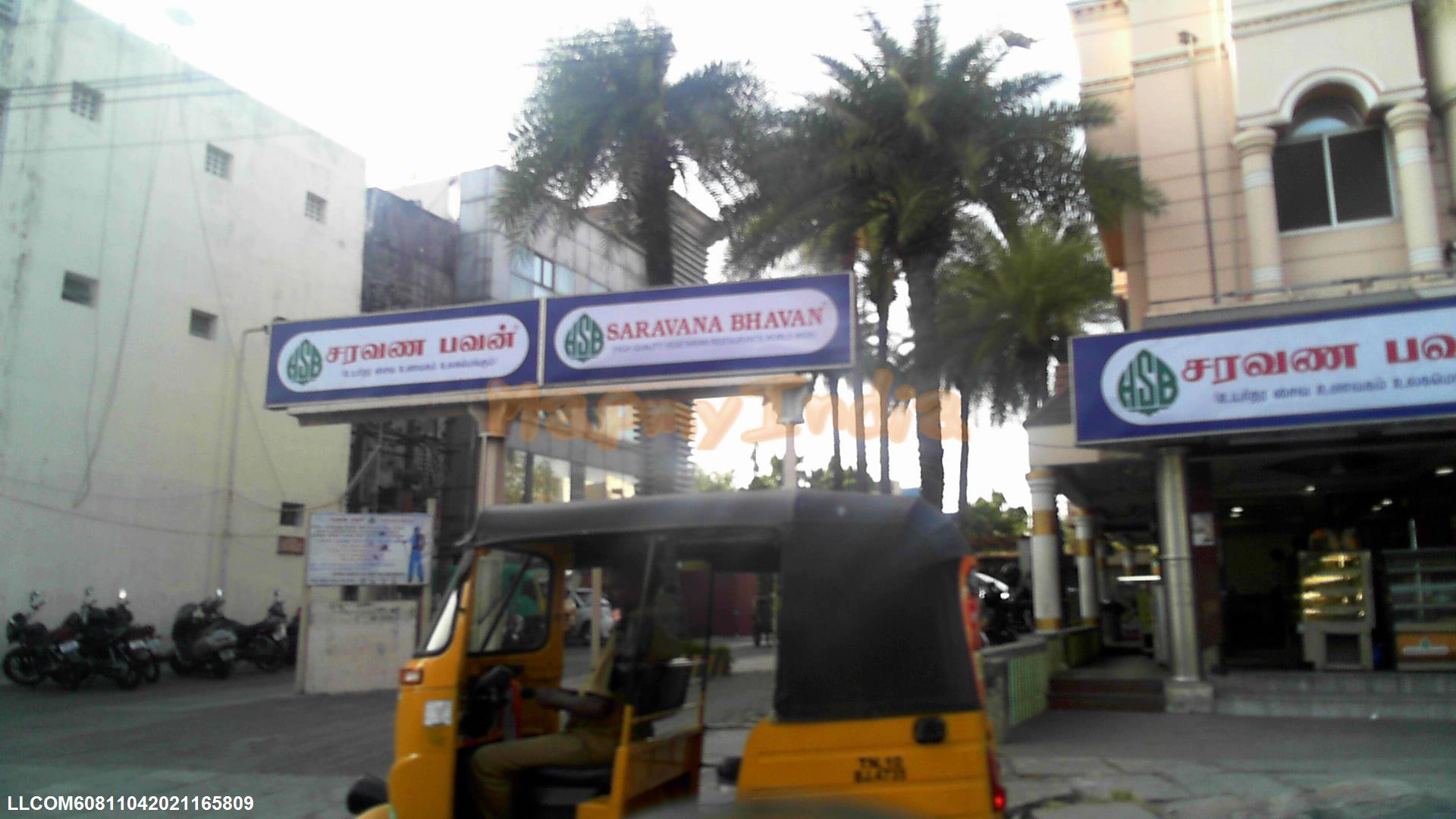

Supplement: Supplementary file 2 — Supplementary Material 2 [file 41598_2026_40742_MOESM2_ESM.zip › sample_data_yolov5/LCOM60811042021165809.jpg]

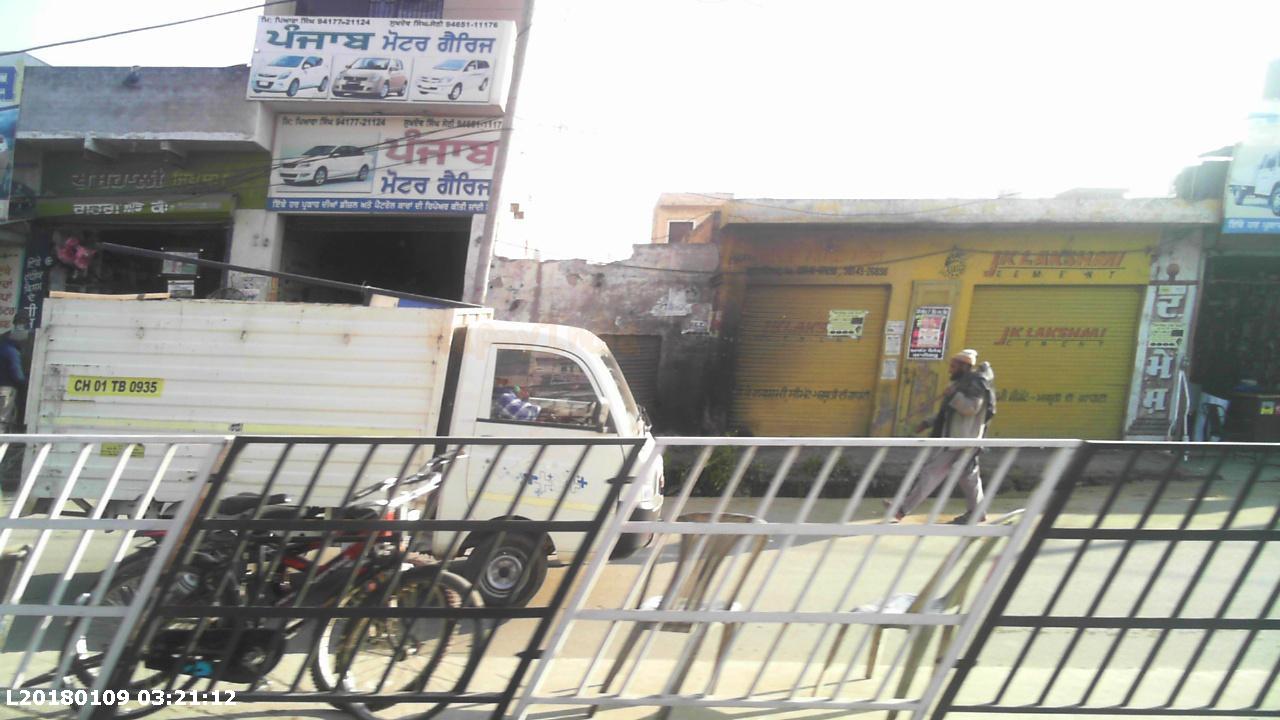

Supplement: Supplementary file 2 — Supplementary Material 2 [file 41598_2026_40742_MOESM2_ESM.zip › sample_data_yolov5/01-09 03.21.12.jpg]

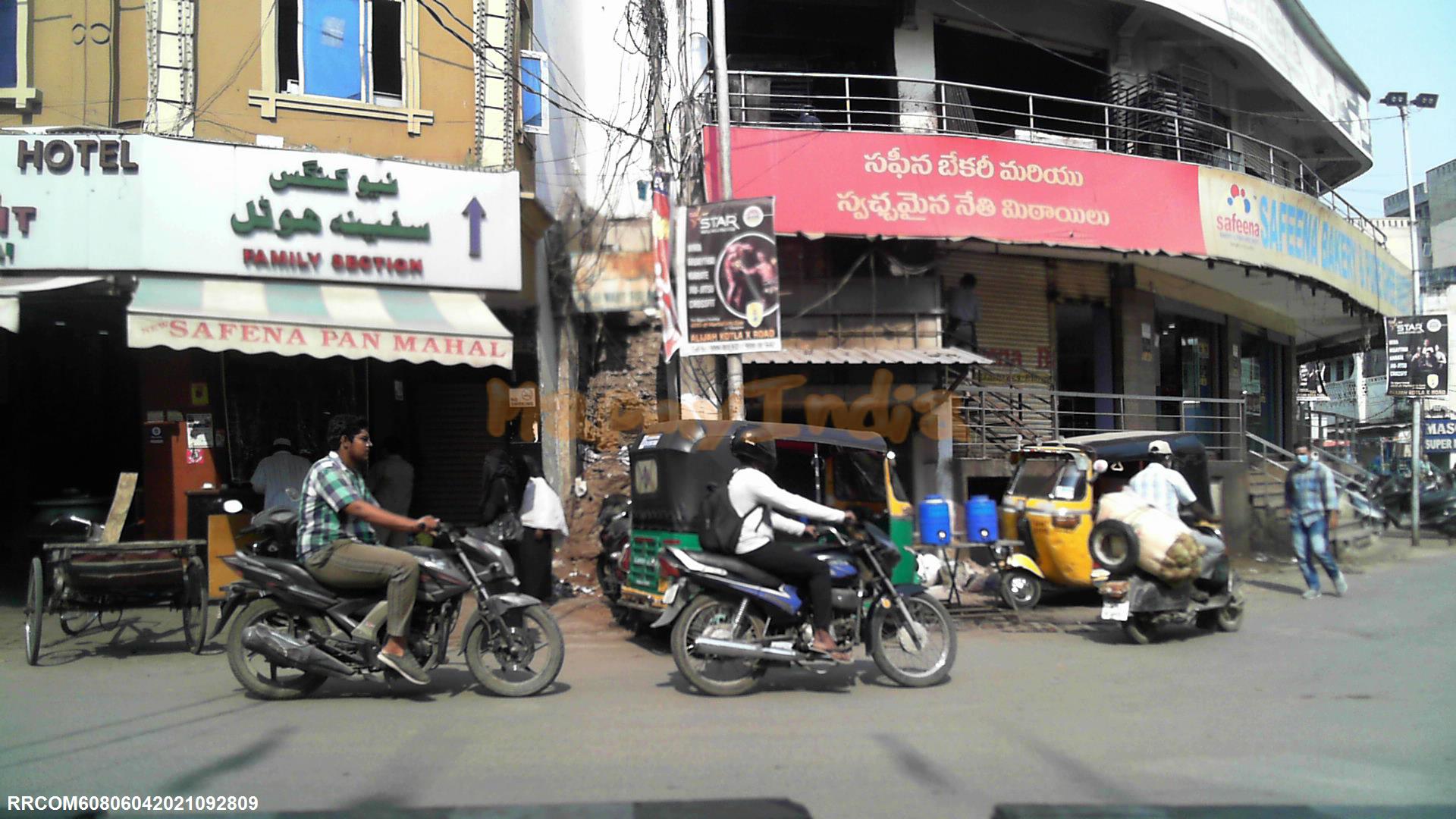

Supplement: Supplementary file 2 — Supplementary Material 2 [file 41598_2026_40742_MOESM2_ESM.zip › sample_data_yolov5/RCOM60806042021092809.jpg]

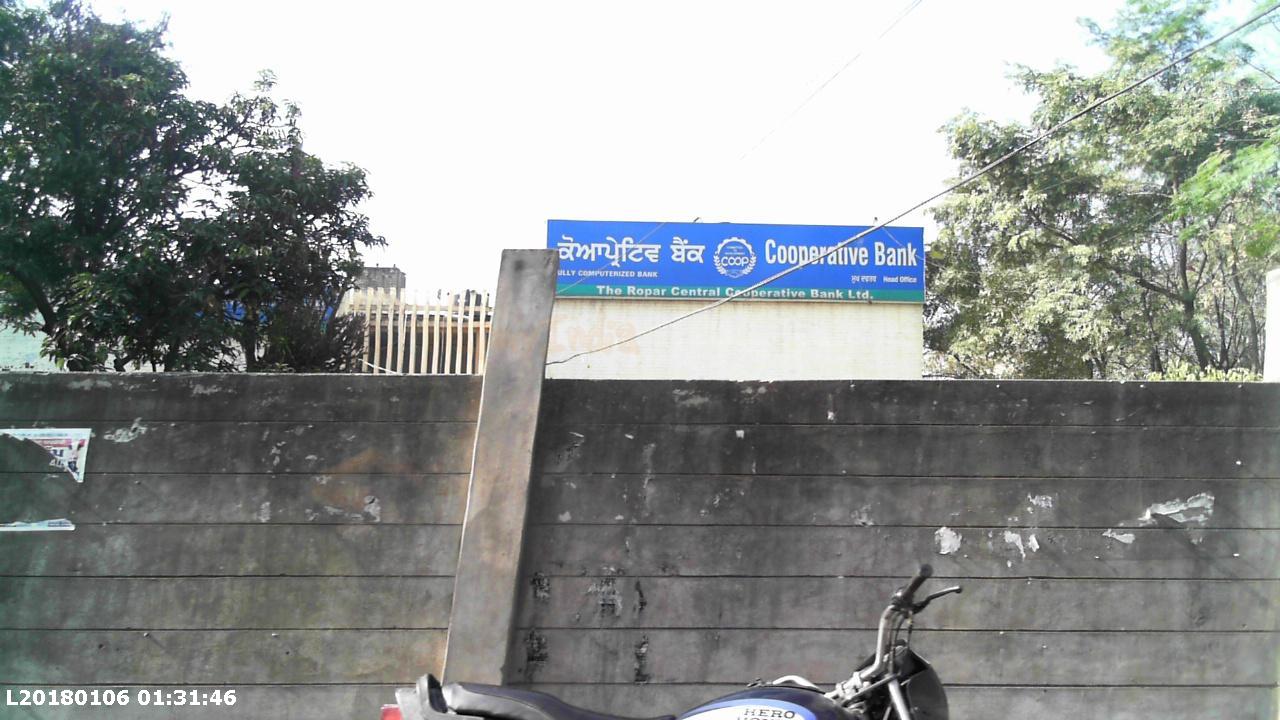

Supplement: Supplementary file 2 — Supplementary Material 2 [file 41598_2026_40742_MOESM2_ESM.zip › sample_data_yolov5/01-06 01.31.46.jpg]

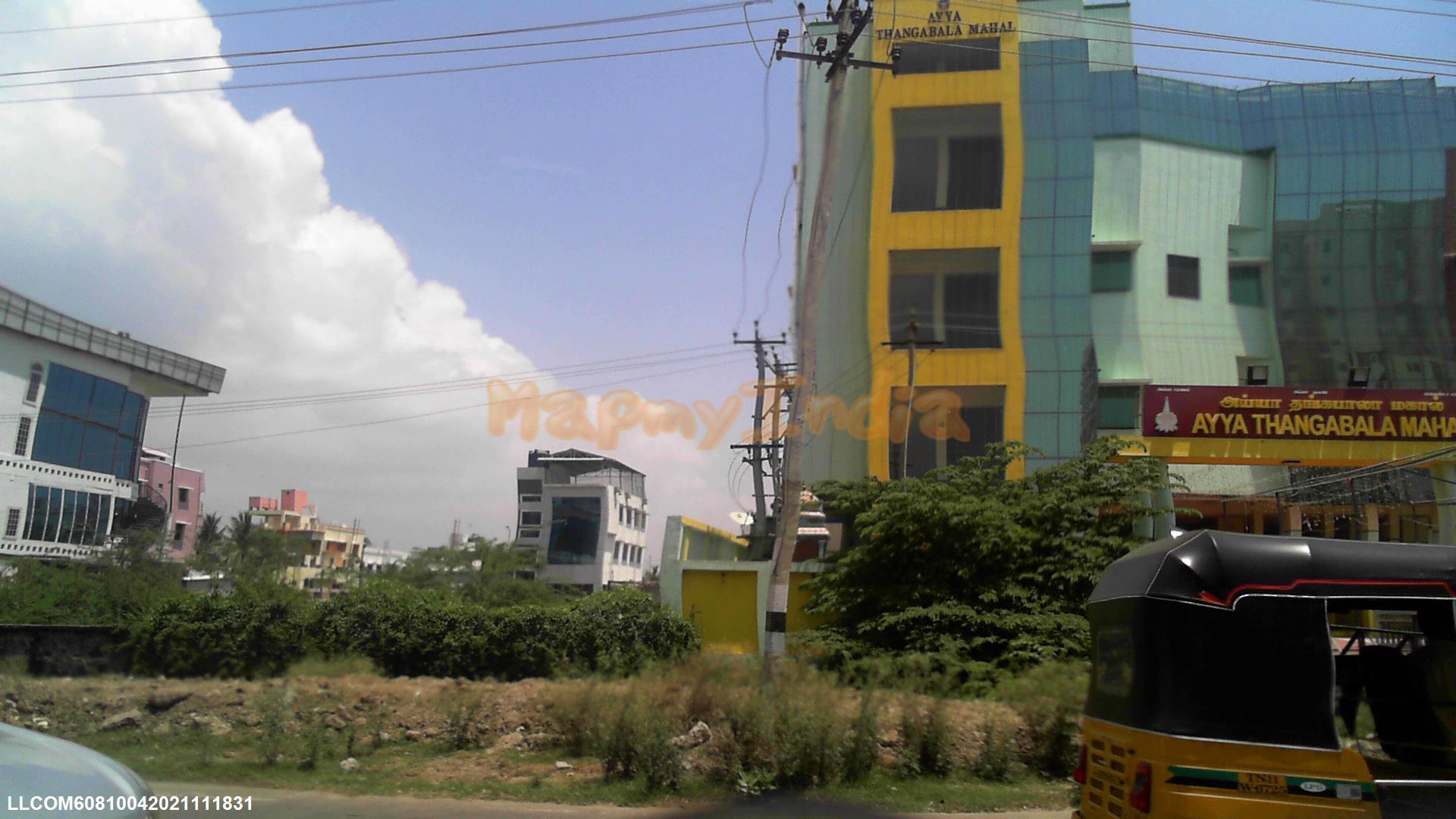

Supplement: Supplementary file 2 — Supplementary Material 2 [file 41598_2026_40742_MOESM2_ESM.zip › sample_data_yolov5/LCOM60810042021111831.jpg]

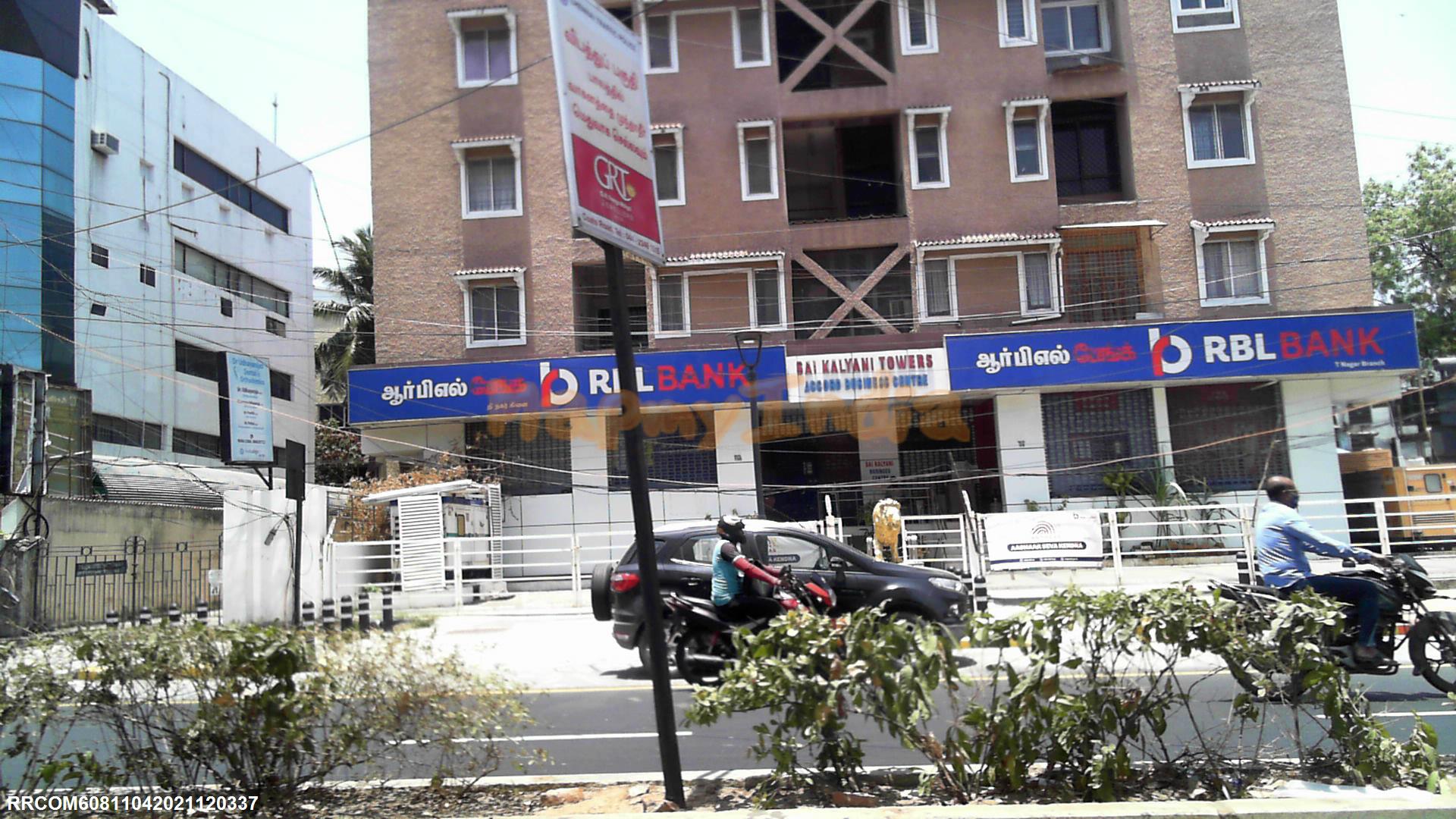

Supplement: Supplementary file 2 — Supplementary Material 2 [file 41598_2026_40742_MOESM2_ESM.zip › sample_data_yolov5/RCOM60811042021120337.jpg]

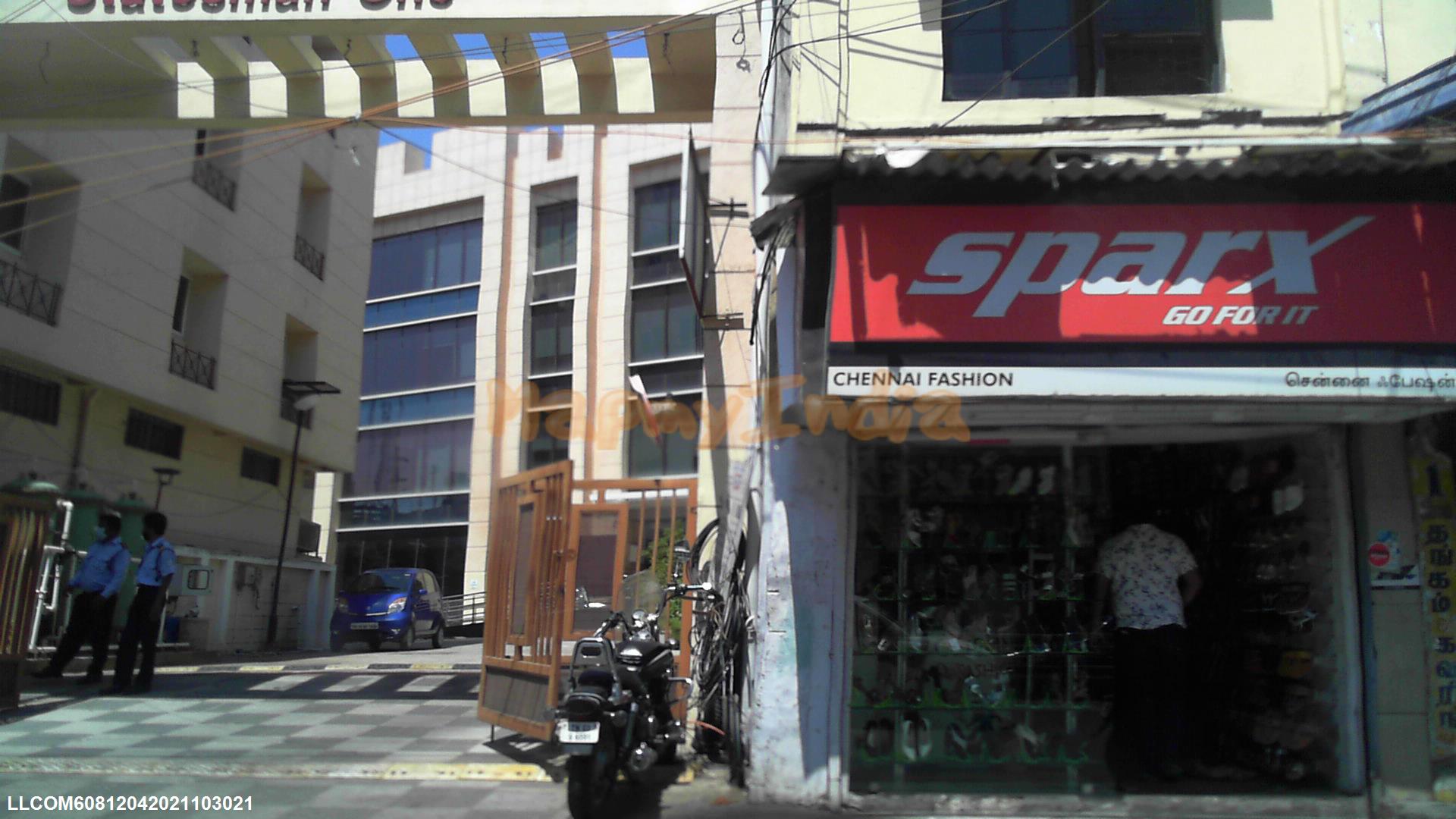

Supplement: Supplementary file 2 — Supplementary Material 2 [file 41598_2026_40742_MOESM2_ESM.zip › sample_data_yolov5/LCOM60812042021103021.jpg]

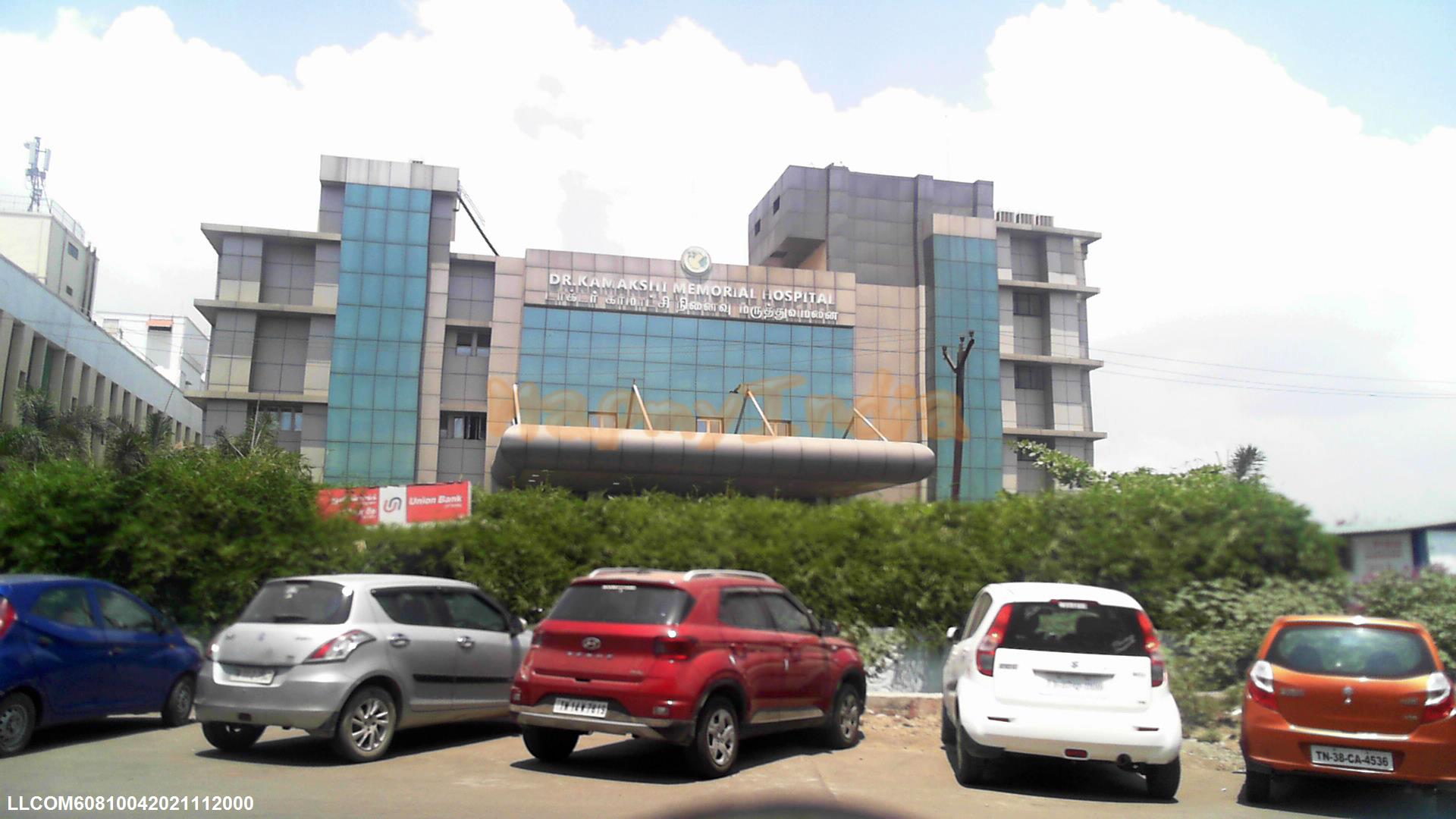

Supplement: Supplementary file 2 — Supplementary Material 2 [file 41598_2026_40742_MOESM2_ESM.zip › sample_data_yolov5/LCOM60810042021112000.jpg]

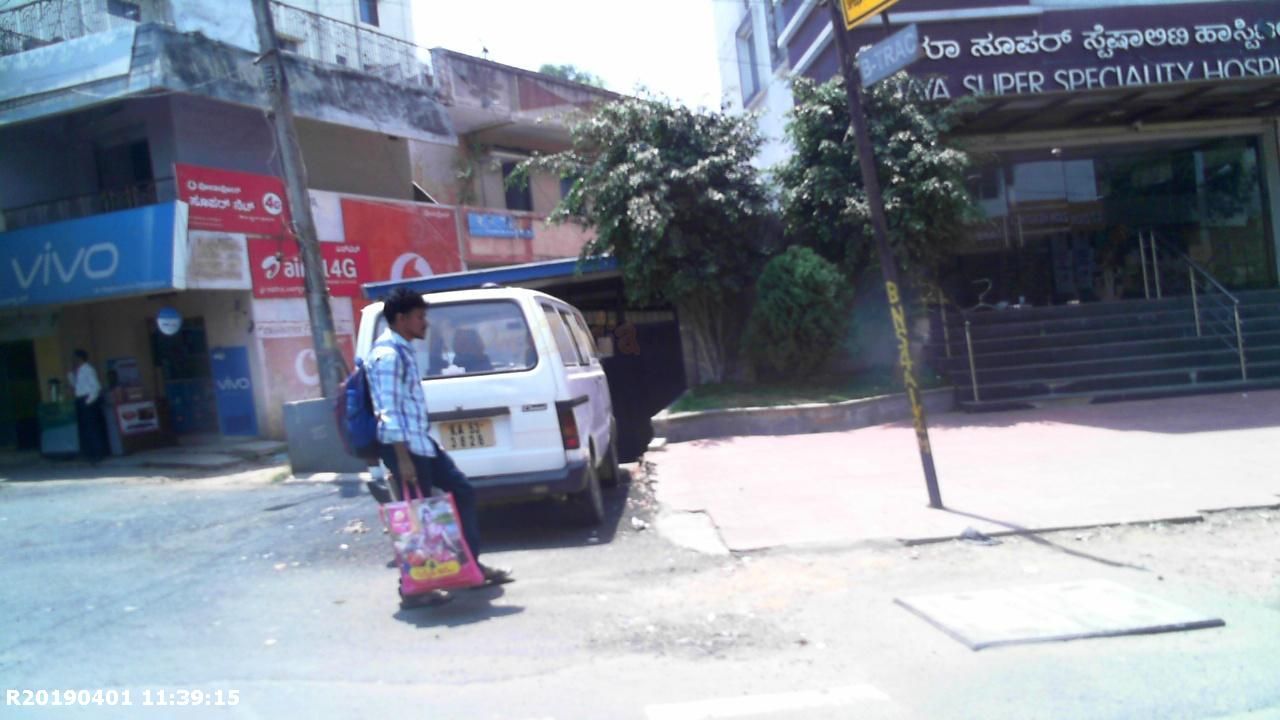

Supplement: Supplementary file 2 — Supplementary Material 2 [file 41598_2026_40742_MOESM2_ESM.zip › sample_data_yolov5/04-01_11.39.15.jpg]

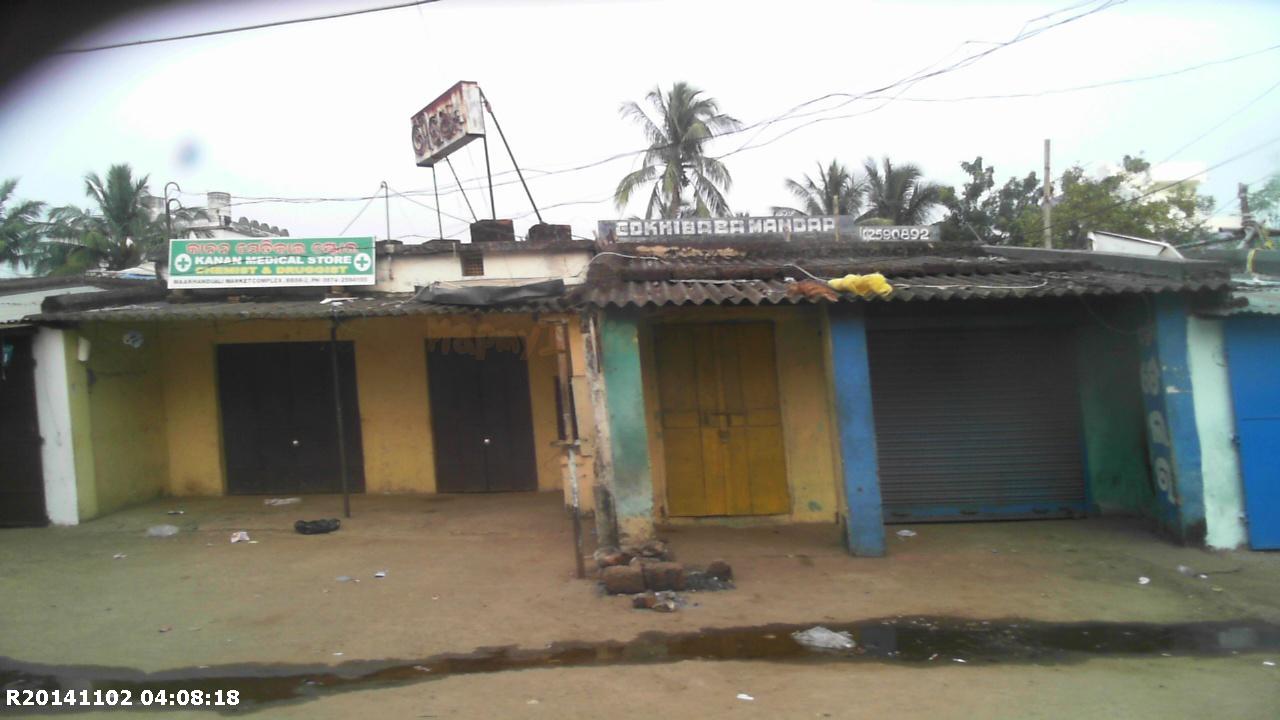

Supplement: Supplementary file 2 — Supplementary Material 2 [file 41598_2026_40742_MOESM2_ESM.zip › sample_data_yolov5/R_11-02_04.08.18.jpg]

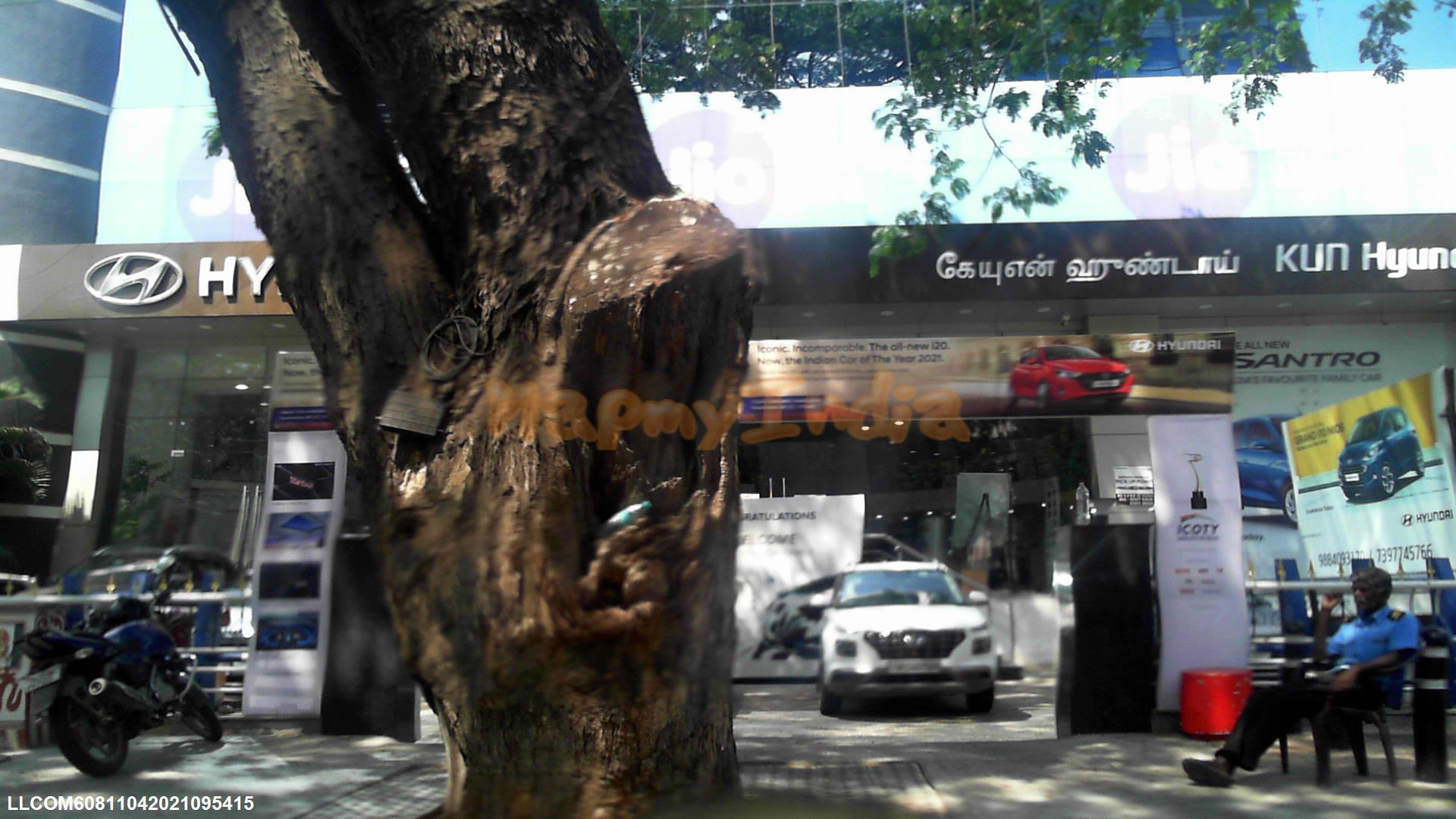

Supplement: Supplementary file 2 — Supplementary Material 2 [file 41598_2026_40742_MOESM2_ESM.zip › sample_data_yolov5/LCOM60811042021095415.jpg]

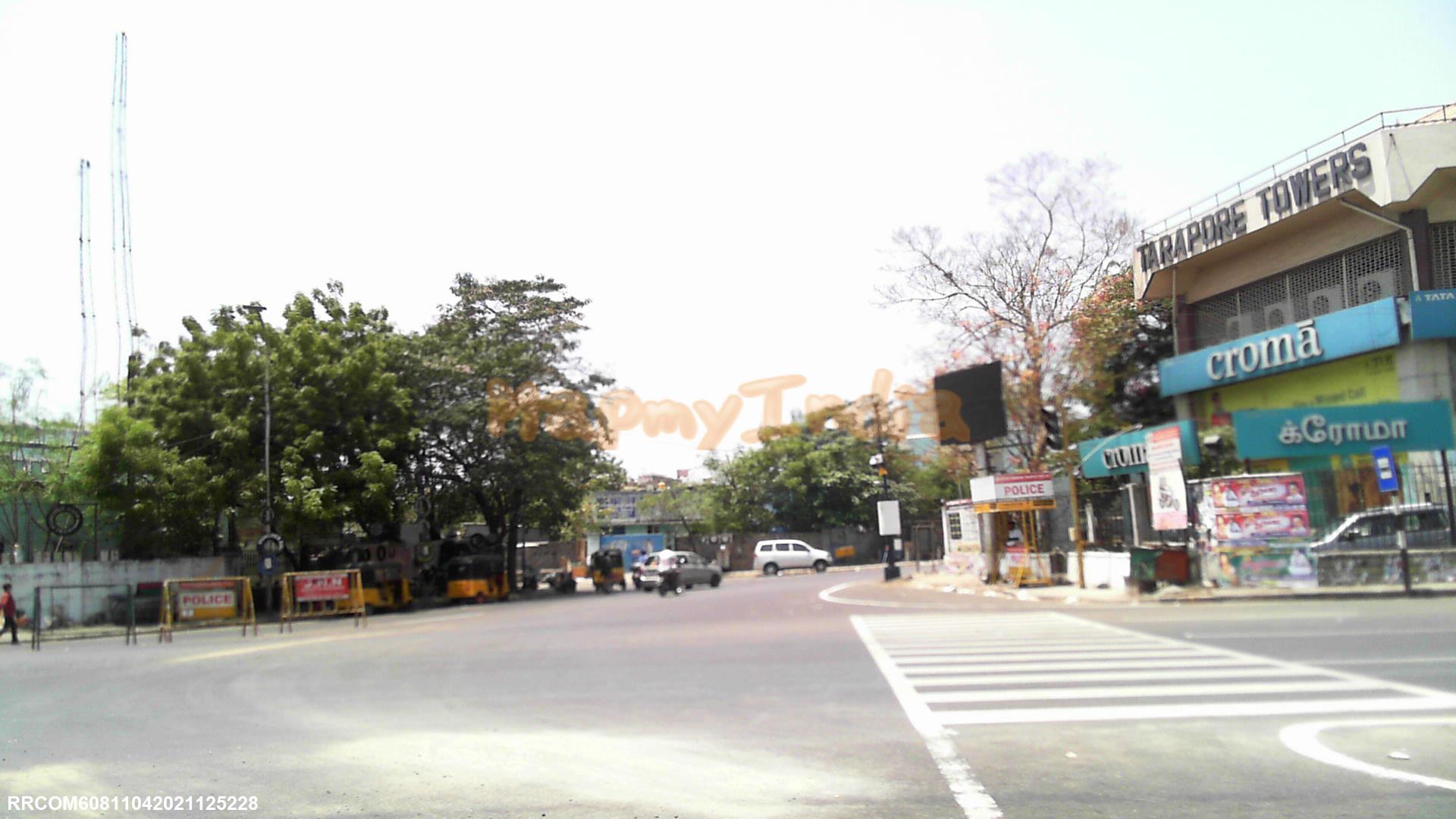

Supplement: Supplementary file 2 — Supplementary Material 2 [file 41598_2026_40742_MOESM2_ESM.zip › sample_data_yolov5/RCOM60811042021125228.jpg]

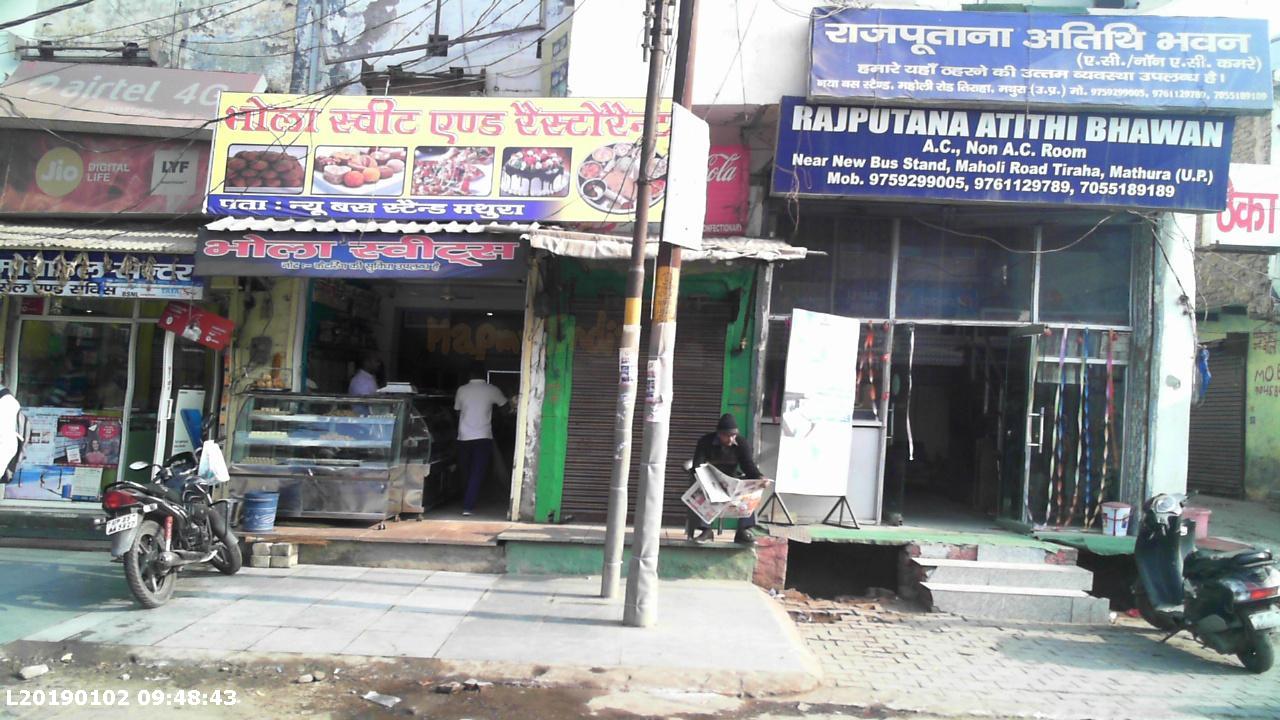

Supplement: Supplementary file 2 — Supplementary Material 2 [file 41598_2026_40742_MOESM2_ESM.zip › sample_data_yolov5/L_01-02_09.48.43.jpg]

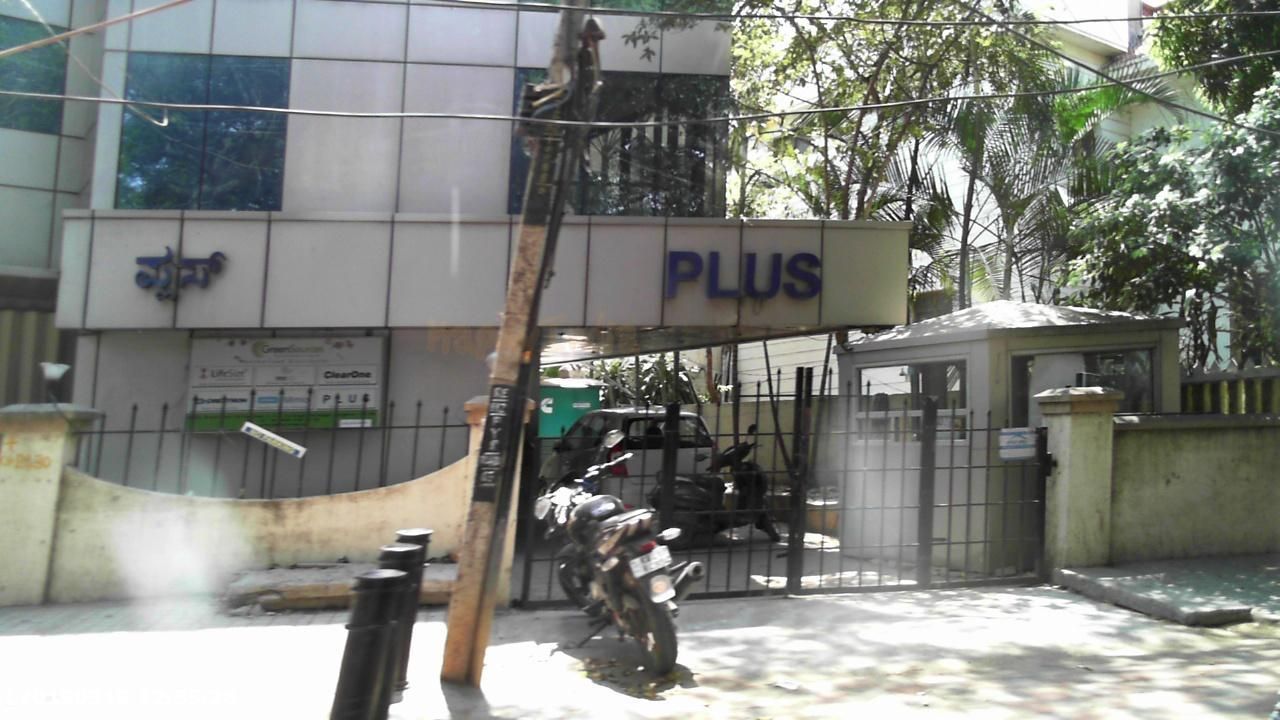

Supplement: Supplementary file 2 — Supplementary Material 2 [file 41598_2026_40742_MOESM2_ESM.zip › sample_data_yolov5/03-18_12.55.26.jpg]

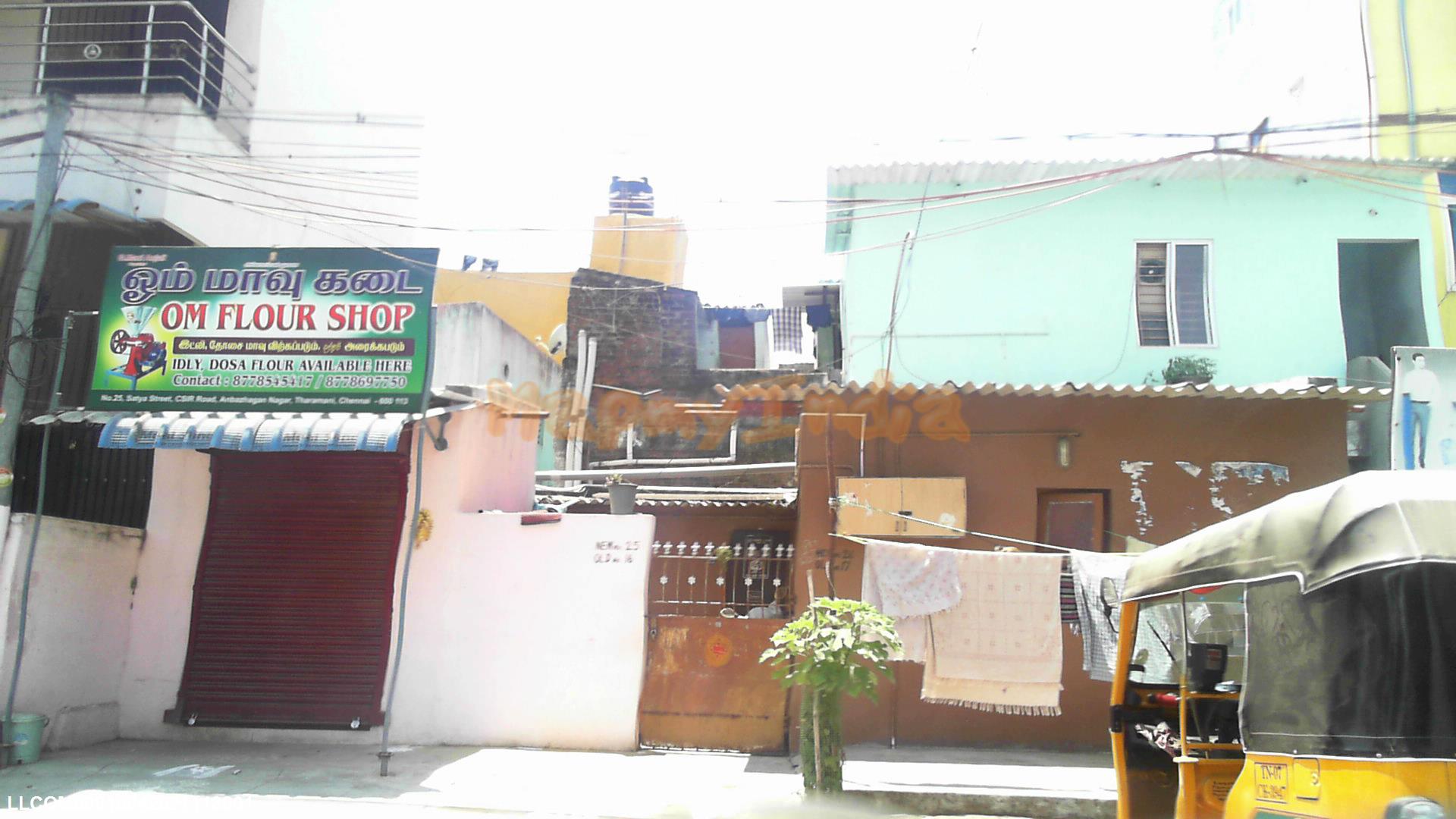

Supplement: Supplementary file 2 — Supplementary Material 2 [file 41598_2026_40742_MOESM2_ESM.zip › sample_data_yolov5/LCOM60810042021113801.jpg]

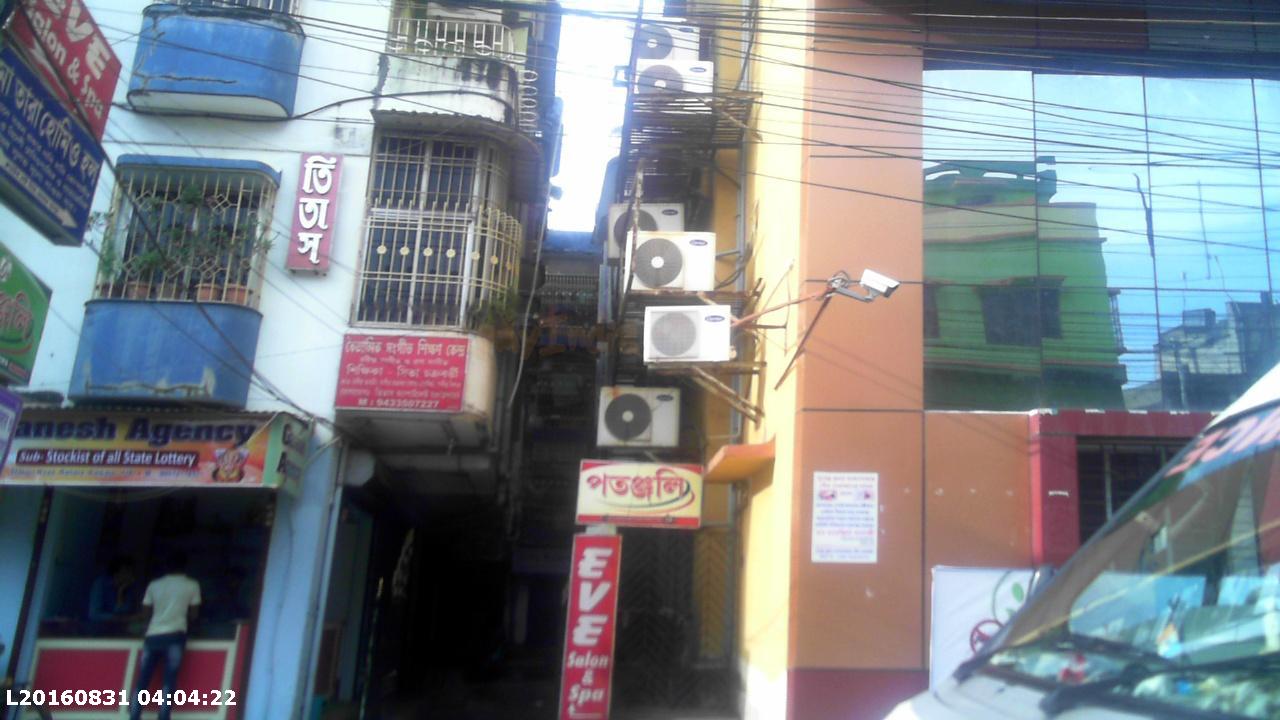

Supplement: Supplementary file 2 — Supplementary Material 2 [file 41598_2026_40742_MOESM2_ESM.zip › sample_data_yolov5/L_08-31_04.04.22.jpg]

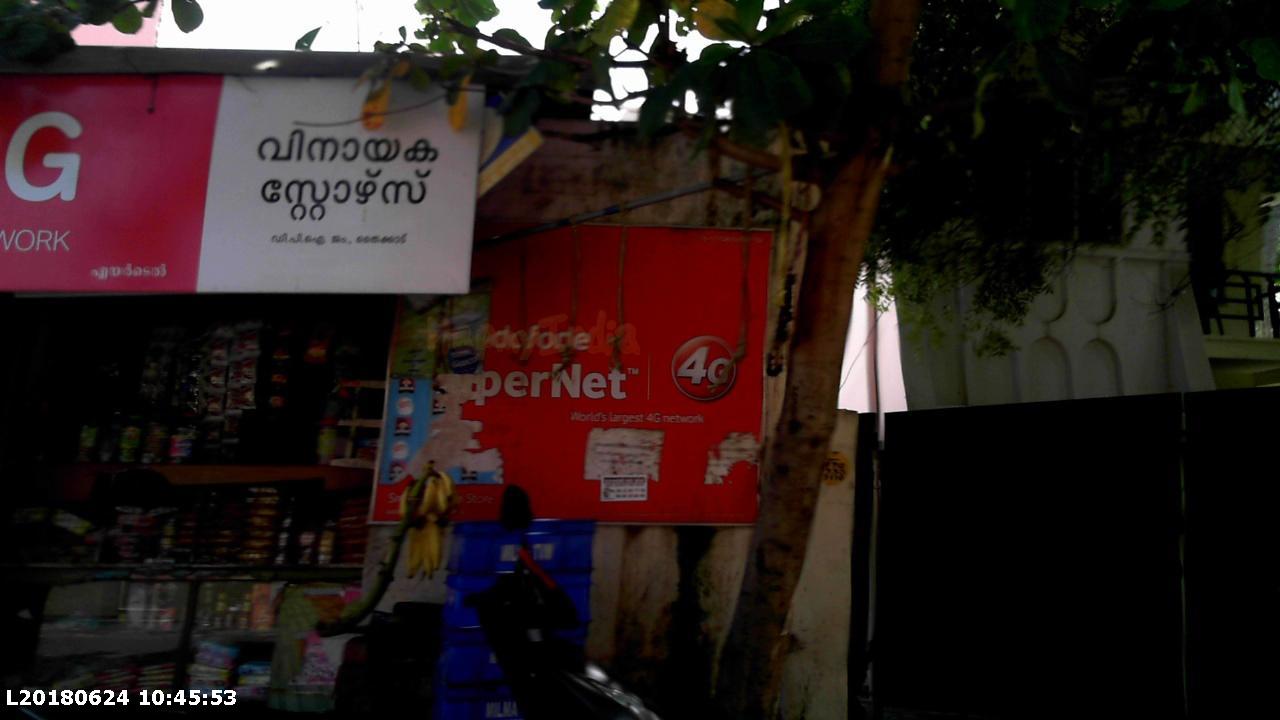

Supplement: Supplementary file 2 — Supplementary Material 2 [file 41598_2026_40742_MOESM2_ESM.zip › sample_data_yolov5/L_06-24_10.45.53.jpg]

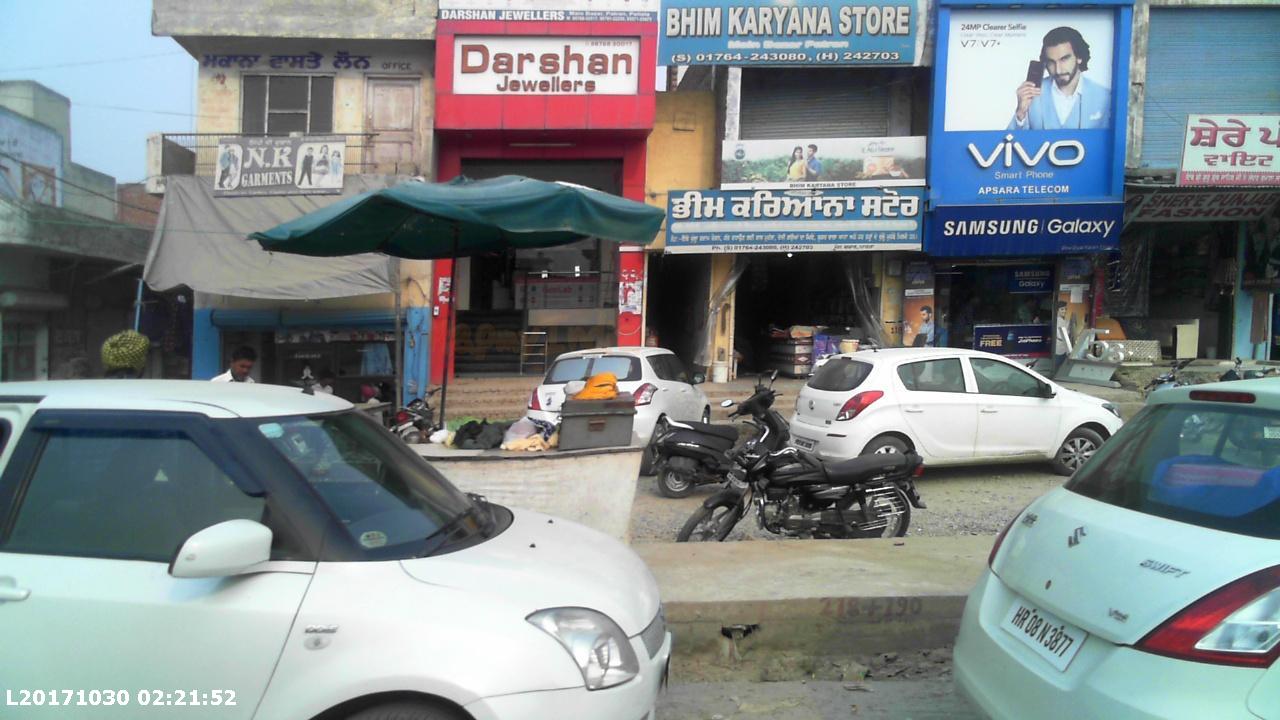

Supplement: Supplementary file 2 — Supplementary Material 2 [file 41598_2026_40742_MOESM2_ESM.zip › sample_data_yolov5/10-30 02.21.52.jpg]

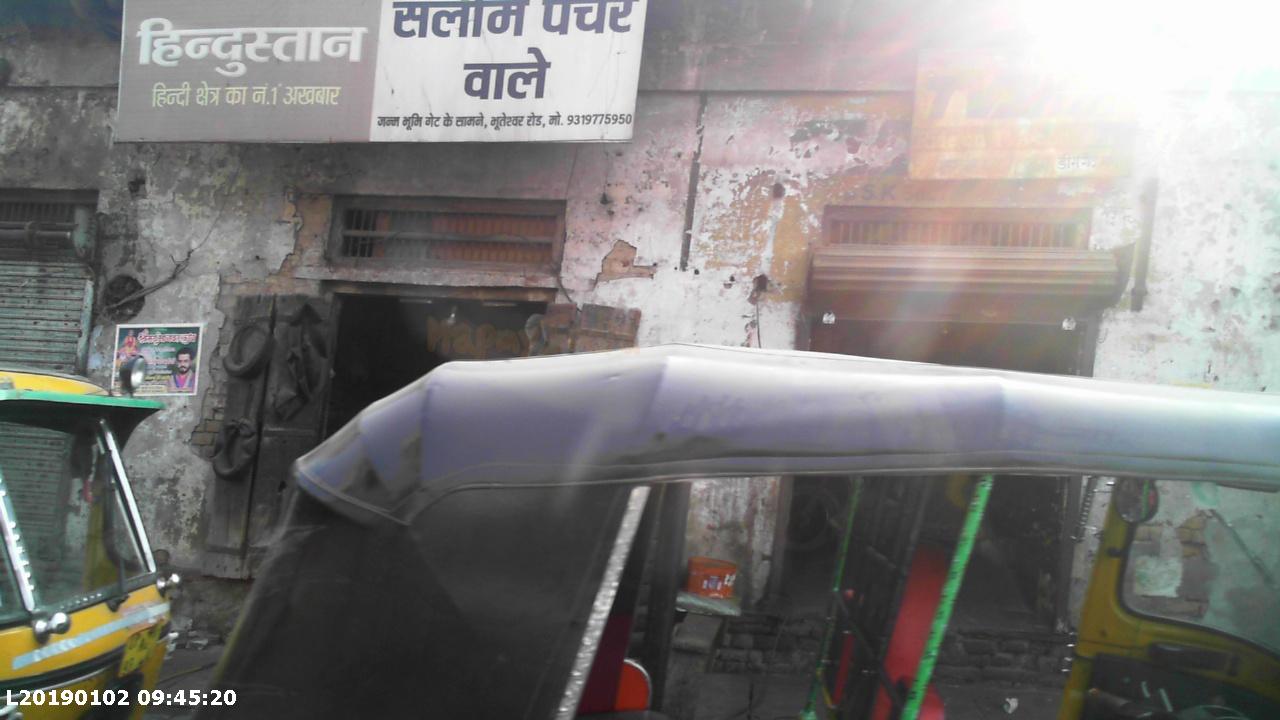

Supplement: Supplementary file 2 — Supplementary Material 2 [file 41598_2026_40742_MOESM2_ESM.zip › sample_data_yolov5/L_01-02_09.45.20.jpg]

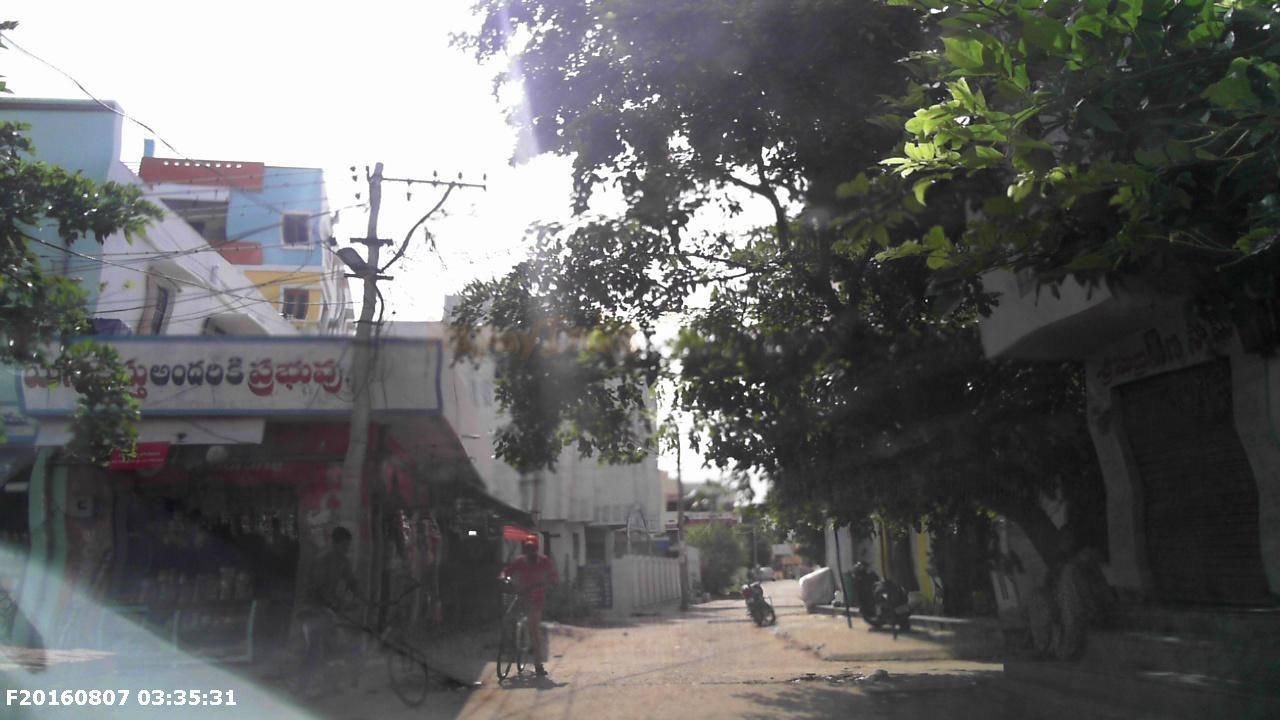

Supplement: Supplementary file 2 — Supplementary Material 2 [file 41598_2026_40742_MOESM2_ESM.zip › sample_data_yolov5/08-07 03.35.31.jpg]

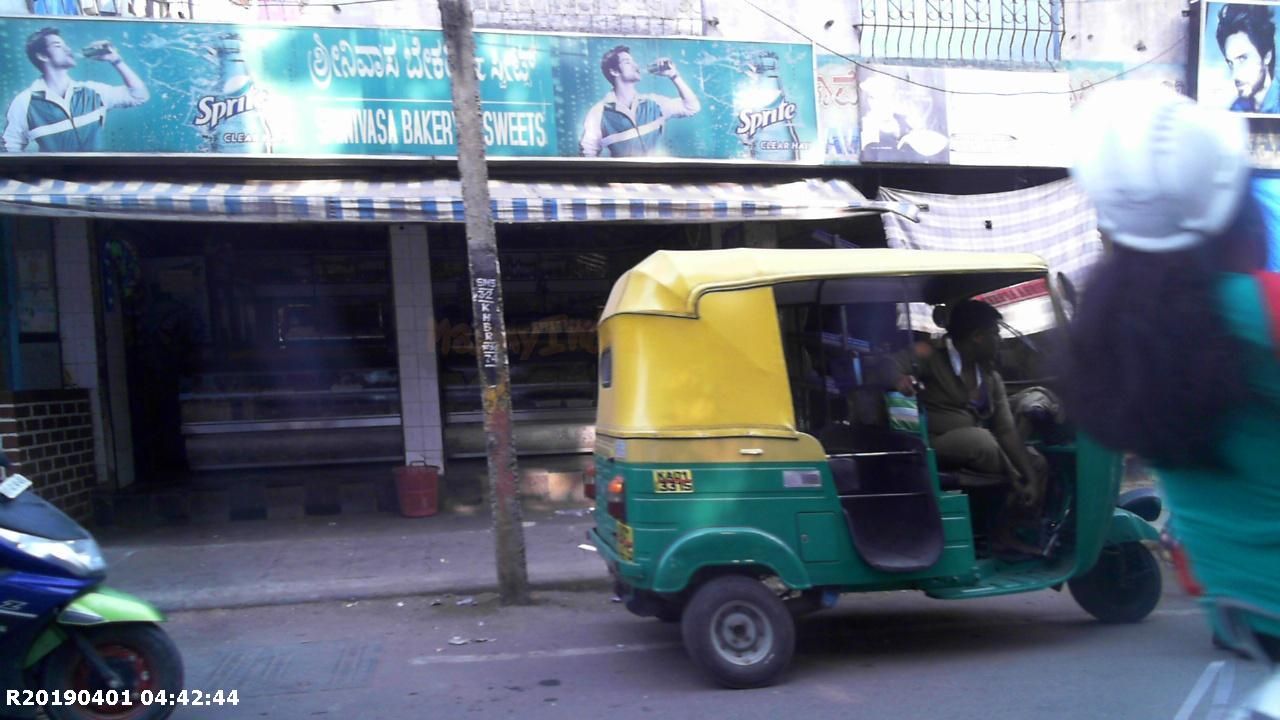

Supplement: Supplementary file 2 — Supplementary Material 2 [file 41598_2026_40742_MOESM2_ESM.zip › sample_data_yolov5/04-01_04.42.44.jpg]

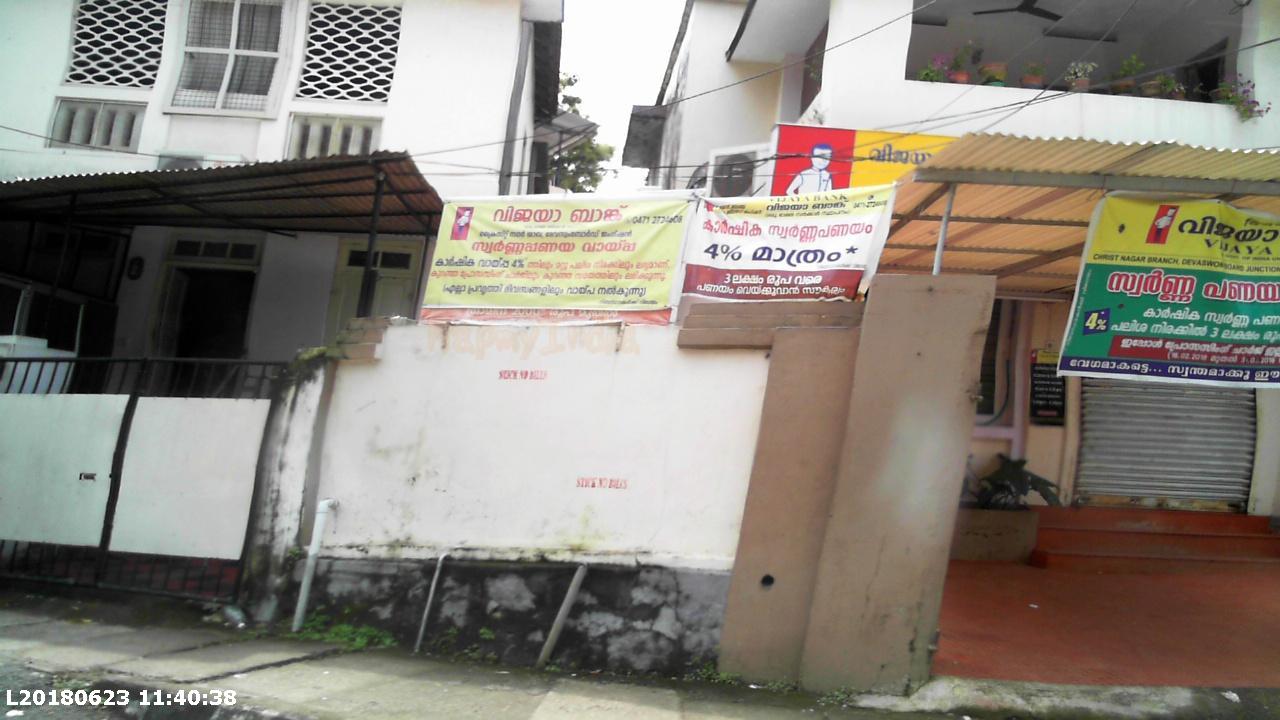

Supplement: Supplementary file 2 — Supplementary Material 2 [file 41598_2026_40742_MOESM2_ESM.zip › sample_data_yolov5/L_06-23_11.40.38.jpg]

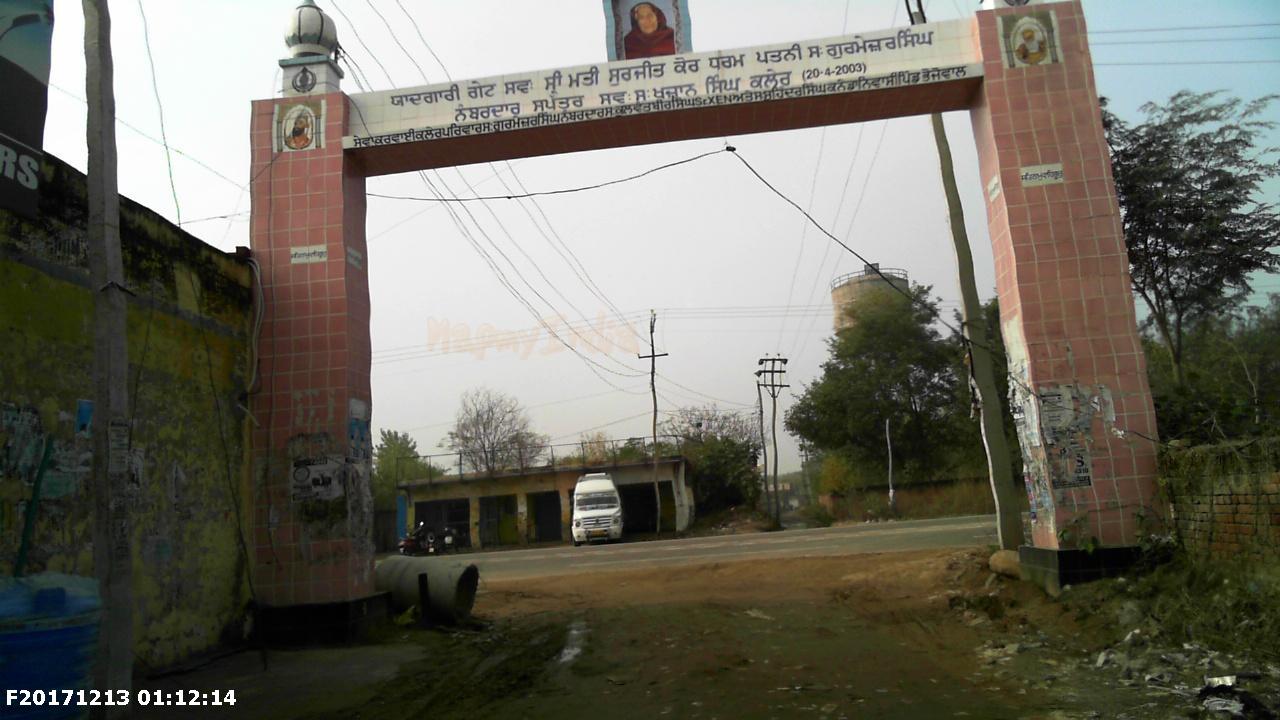

Supplement: Supplementary file 2 — Supplementary Material 2 [file 41598_2026_40742_MOESM2_ESM.zip › sample_data_yolov5/12-13 01.12.14.jpg]

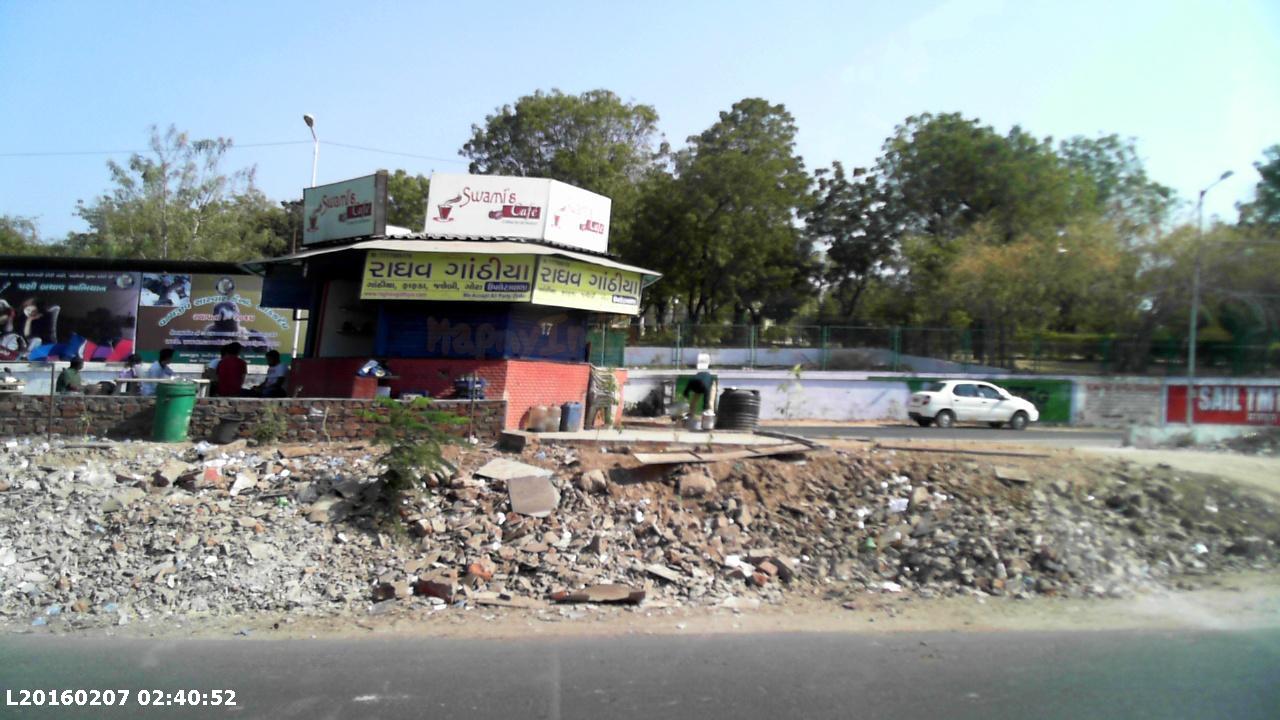

Supplement: Supplementary file 2 — Supplementary Material 2 [file 41598_2026_40742_MOESM2_ESM.zip › sample_data_yolov5/02-07 02.40.52.jpg]

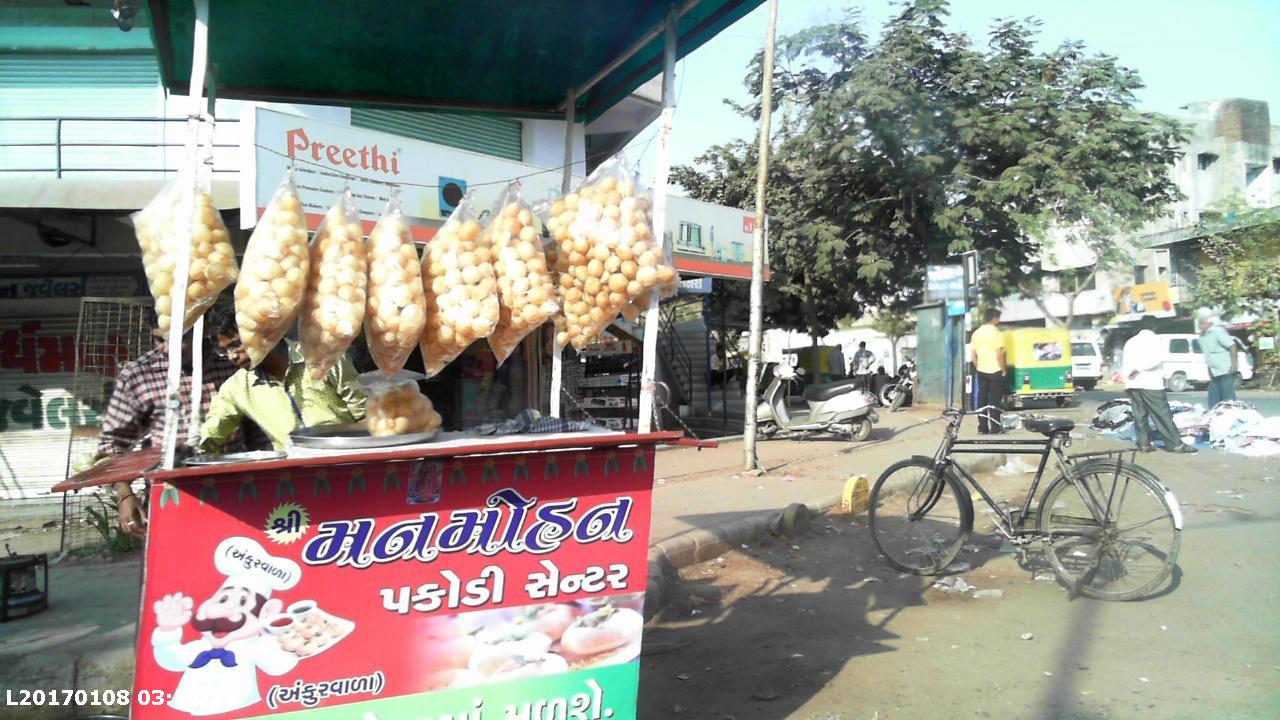

Supplement: Supplementary file 2 — Supplementary Material 2 [file 41598_2026_40742_MOESM2_ESM.zip › sample_data_yolov5/01-08 03.51.21.jpg]

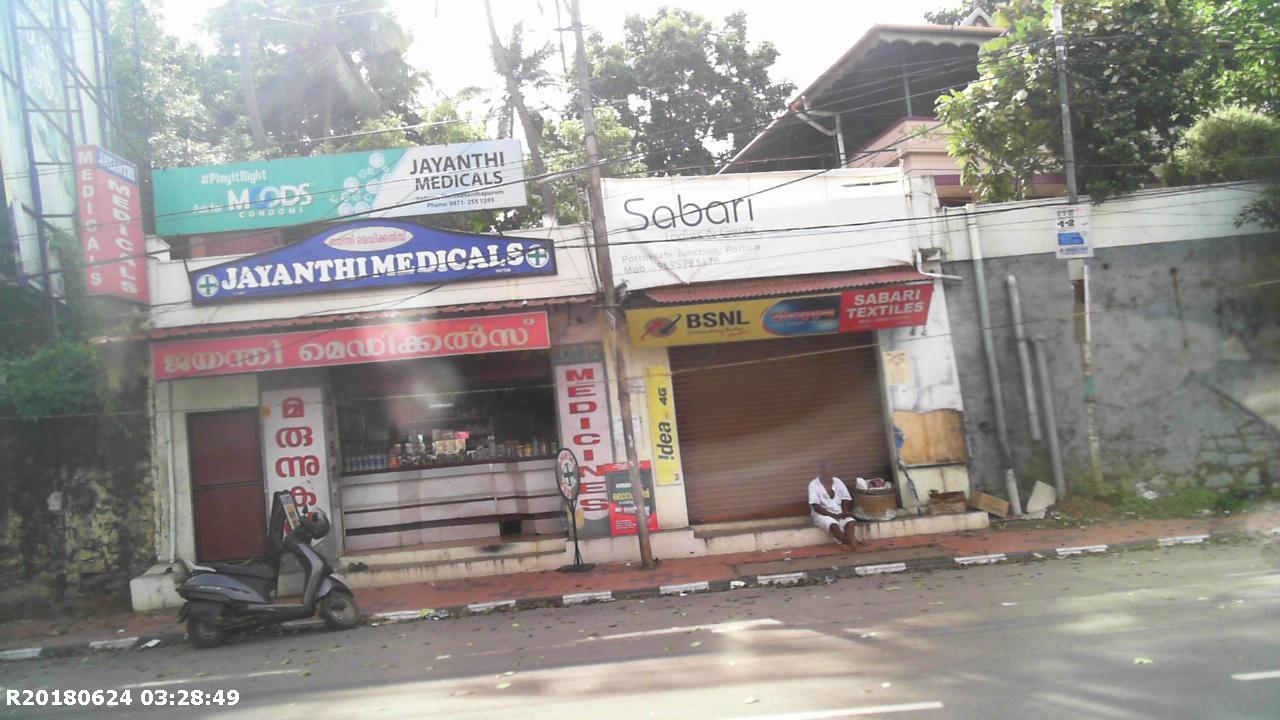

Supplement: Supplementary file 2 — Supplementary Material 2 [file 41598_2026_40742_MOESM2_ESM.zip › sample_data_yolov5/R_06-24_03.28.49.jpg]

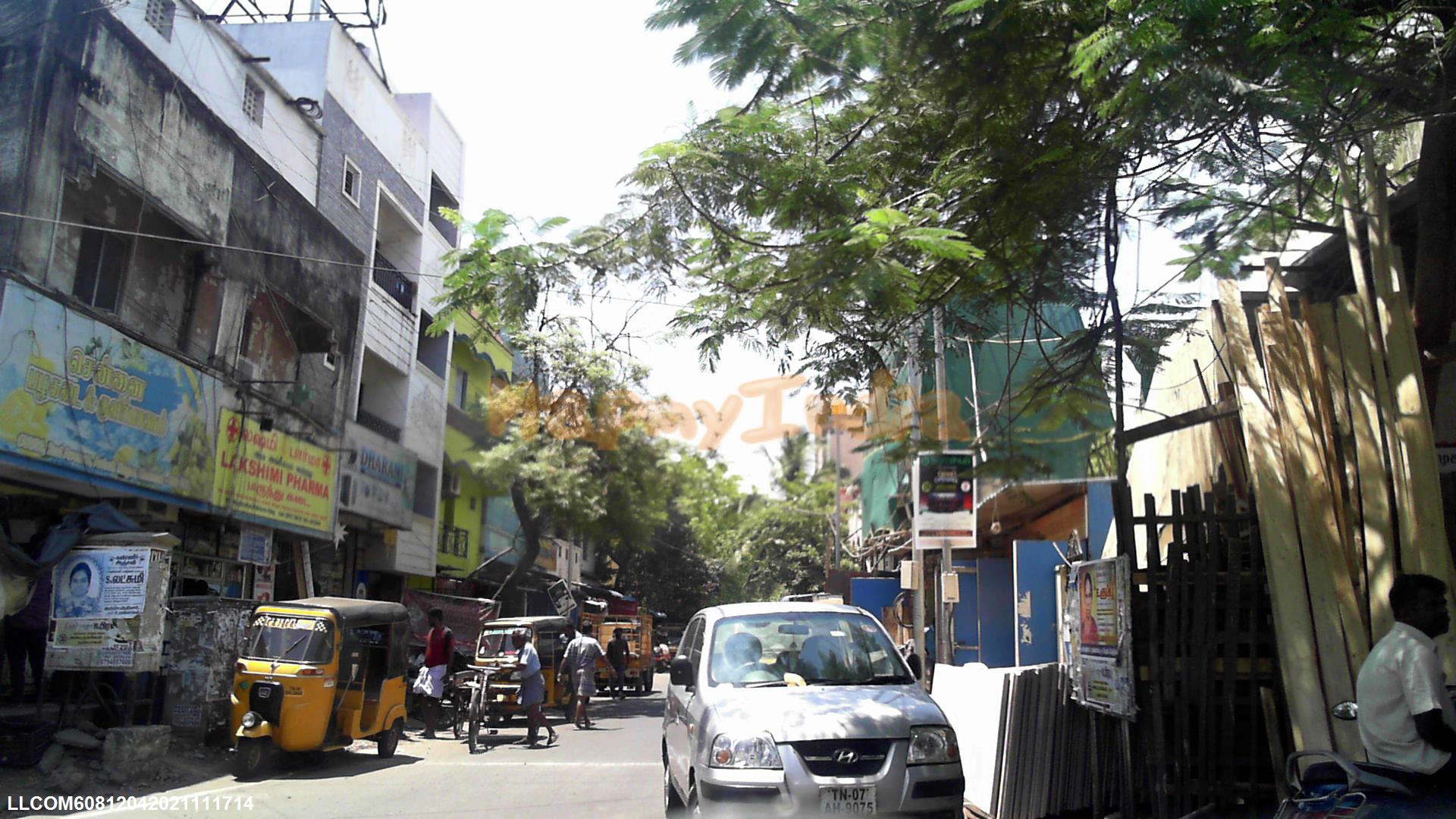

Supplement: Supplementary file 2 — Supplementary Material 2 [file 41598_2026_40742_MOESM2_ESM.zip › sample_data_yolov5/LCOM60812042021111714.jpg]

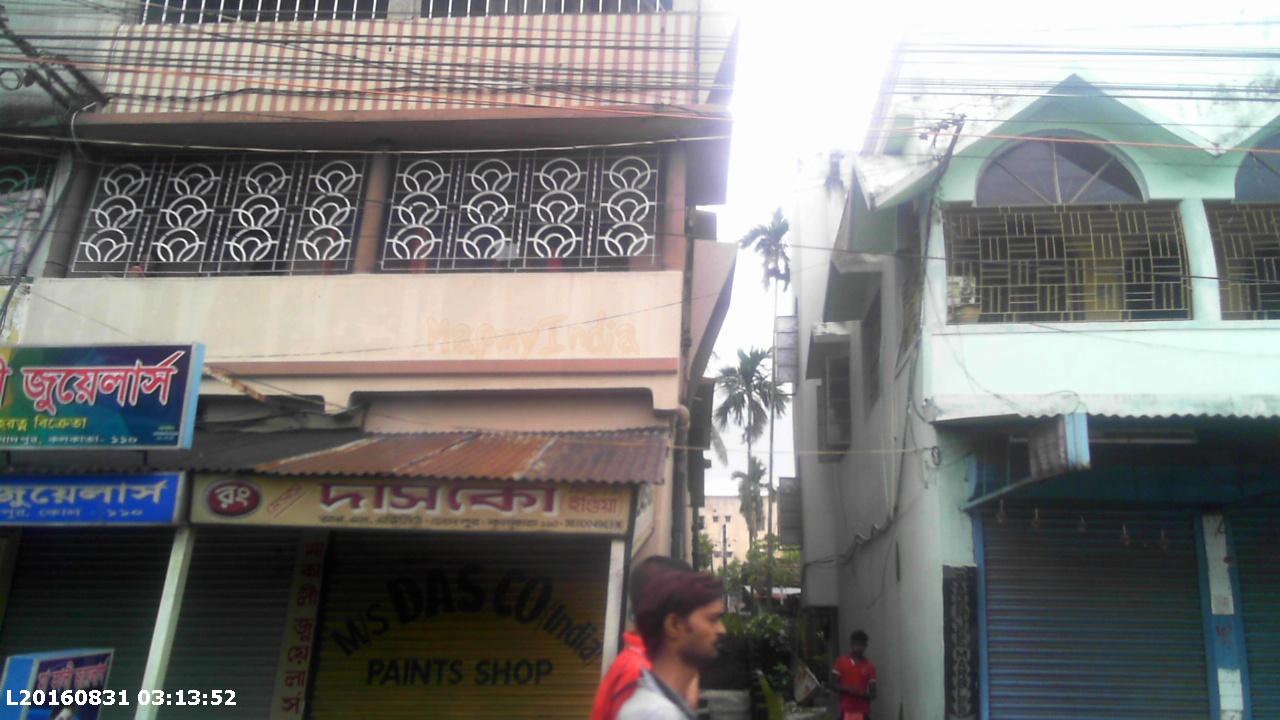

Supplement: Supplementary file 2 — Supplementary Material 2 [file 41598_2026_40742_MOESM2_ESM.zip › sample_data_yolov5/L_08-31_03.13.52.jpg]

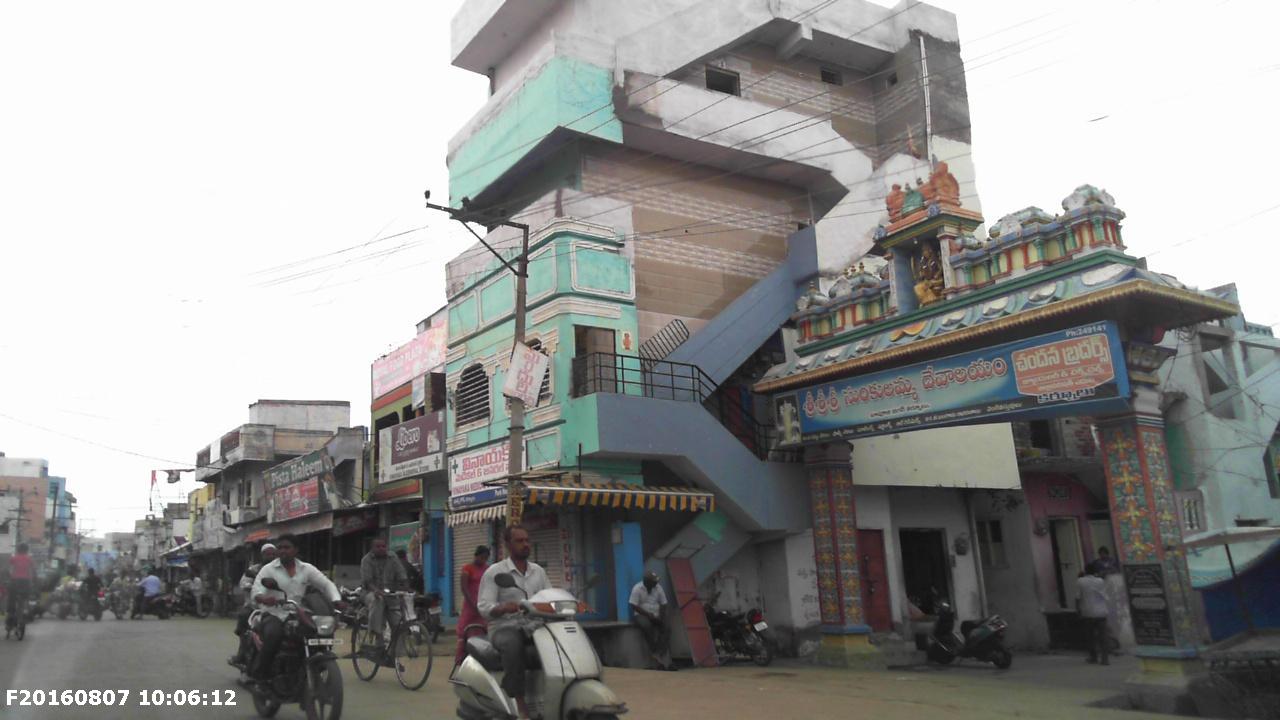

Supplement: Supplementary file 2 — Supplementary Material 2 [file 41598_2026_40742_MOESM2_ESM.zip › sample_data_yolov5/08-07 10.06.12.jpg]

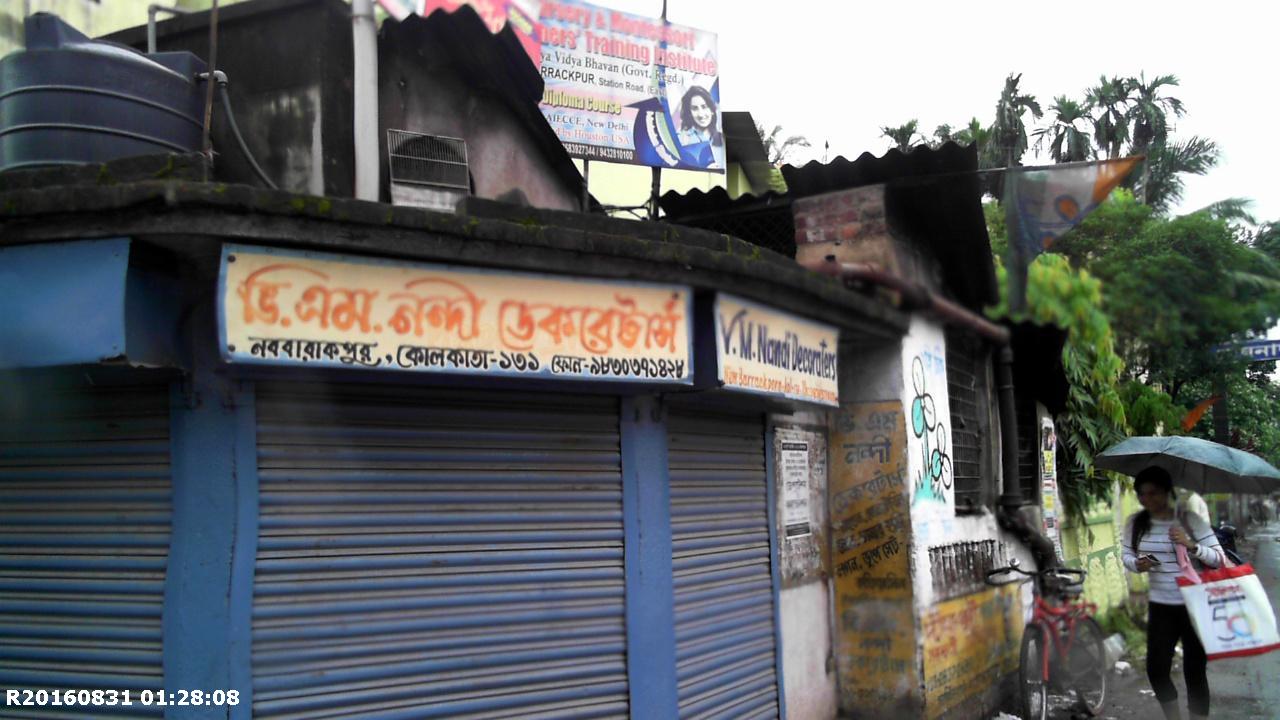

Supplement: Supplementary file 2 — Supplementary Material 2 [file 41598_2026_40742_MOESM2_ESM.zip › sample_data_yolov5/R_08-31_01.28.08.jpg]

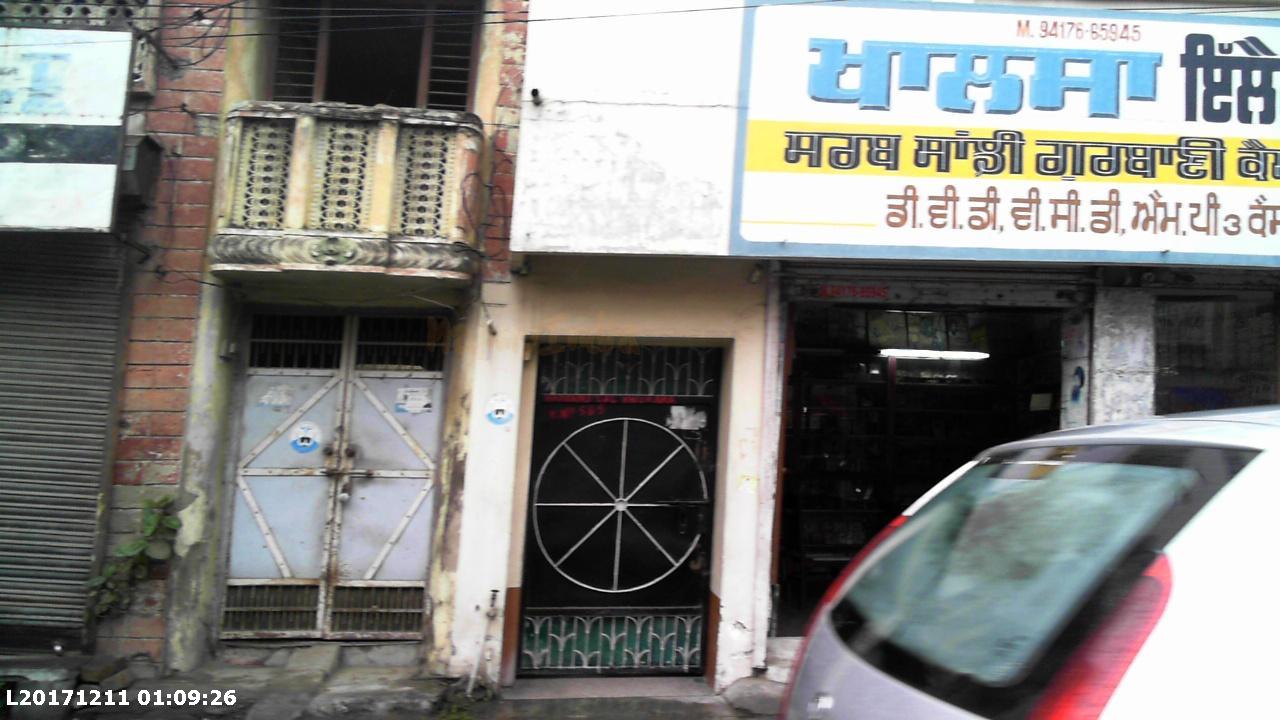

Supplement: Supplementary file 2 — Supplementary Material 2 [file 41598_2026_40742_MOESM2_ESM.zip › sample_data_yolov5/12-11 01.09.26.jpg]

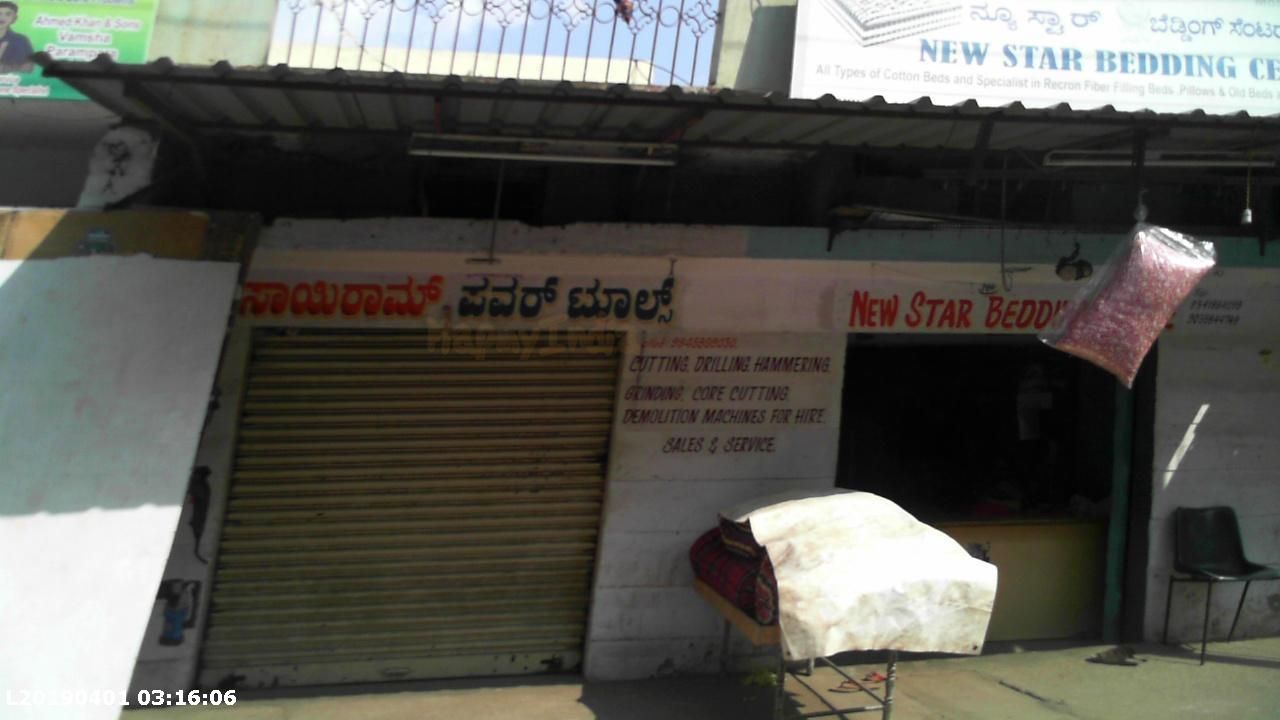

Supplement: Supplementary file 2 — Supplementary Material 2 [file 41598_2026_40742_MOESM2_ESM.zip › sample_data_yolov5/04-01_03.16.06.jpg]

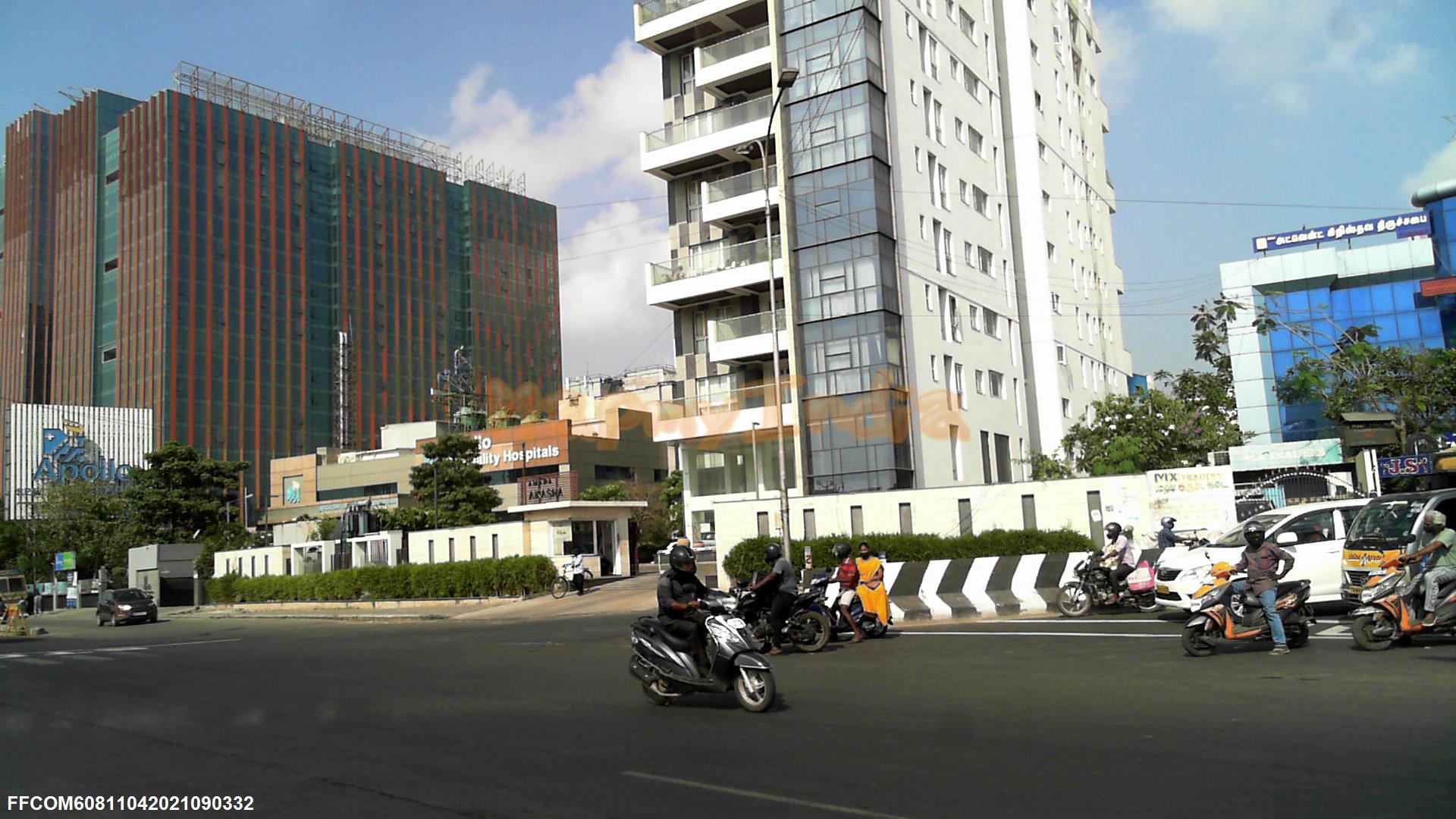

Supplement: Supplementary file 2 — Supplementary Material 2 [file 41598_2026_40742_MOESM2_ESM.zip › sample_data_yolov5/FCOM60811042021090332.jpg]

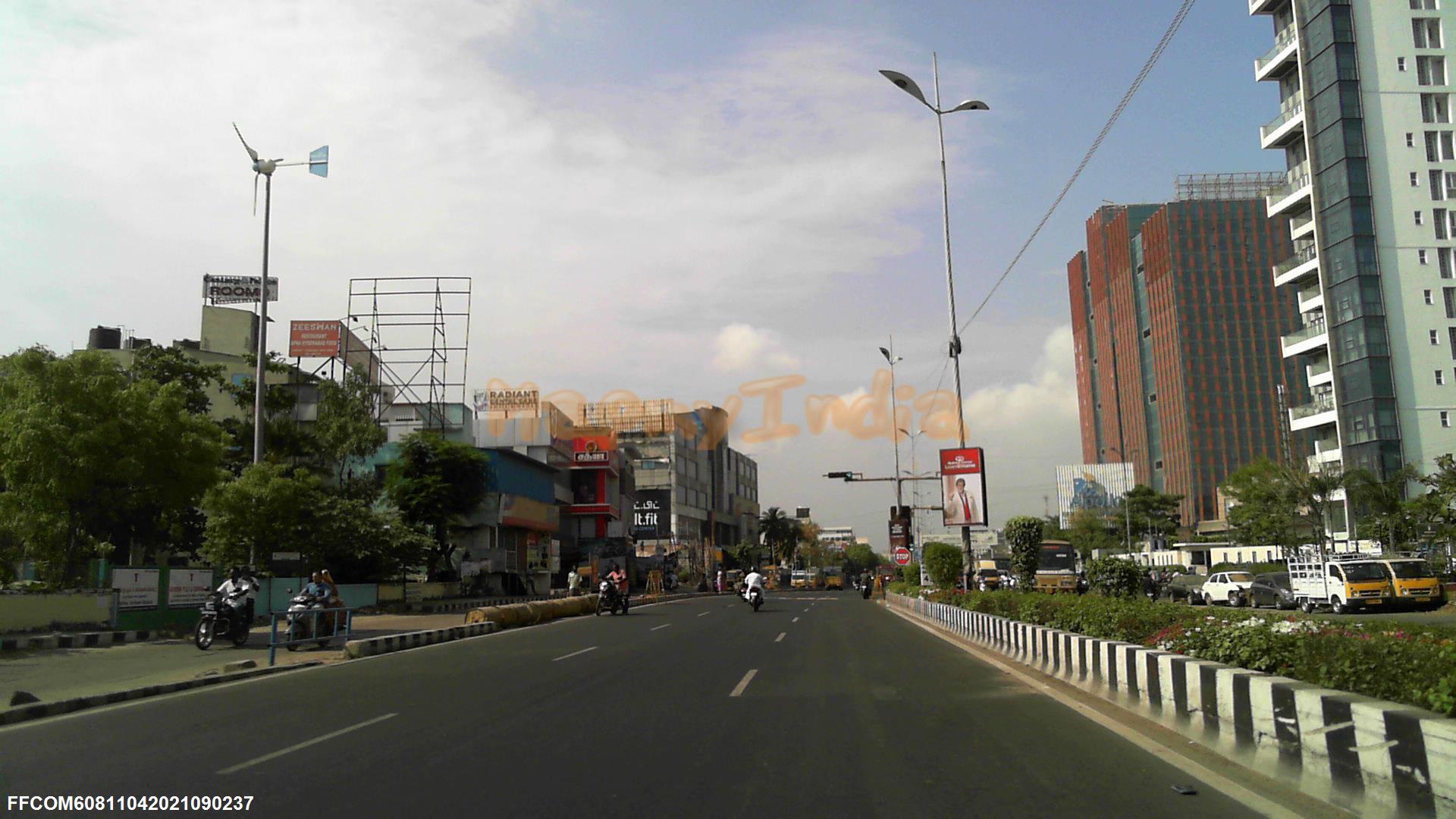

Supplement: Supplementary file 2 — Supplementary Material 2 [file 41598_2026_40742_MOESM2_ESM.zip › sample_data_yolov5/FCOM60811042021090237.jpg]

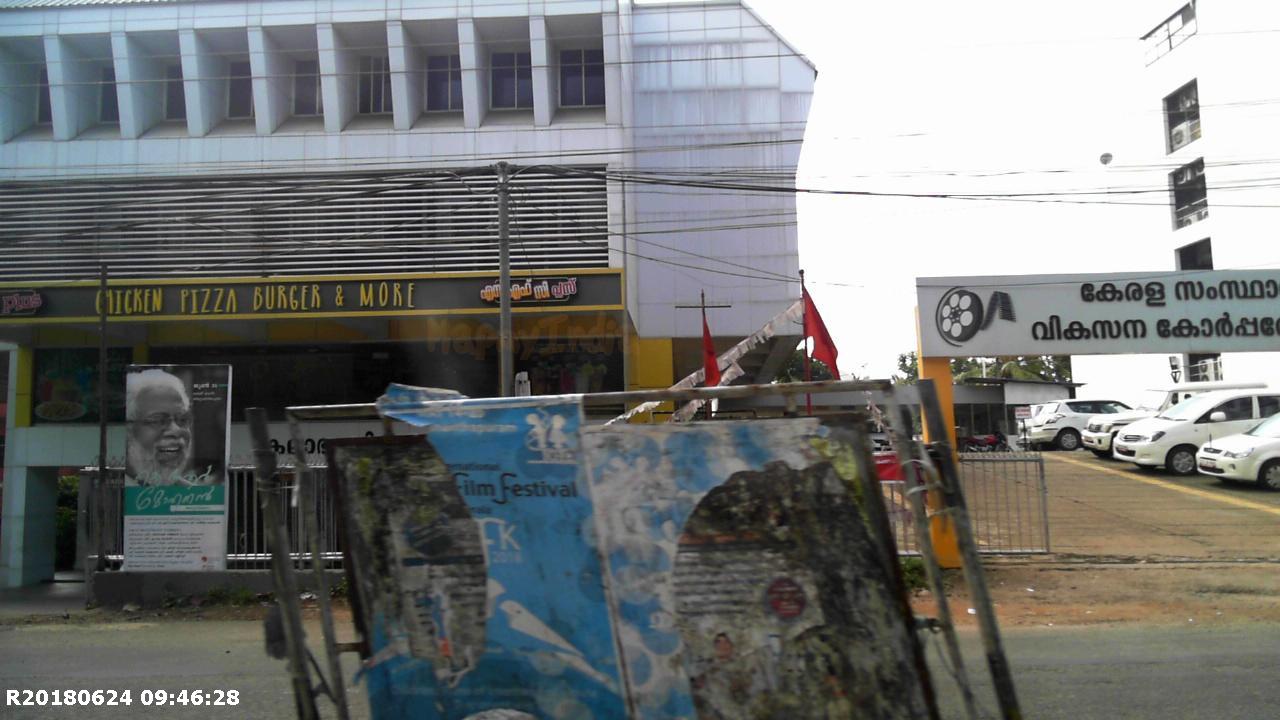

Supplement: Supplementary file 2 — Supplementary Material 2 [file 41598_2026_40742_MOESM2_ESM.zip › sample_data_yolov5/R_06-24_09.46.28.jpg]

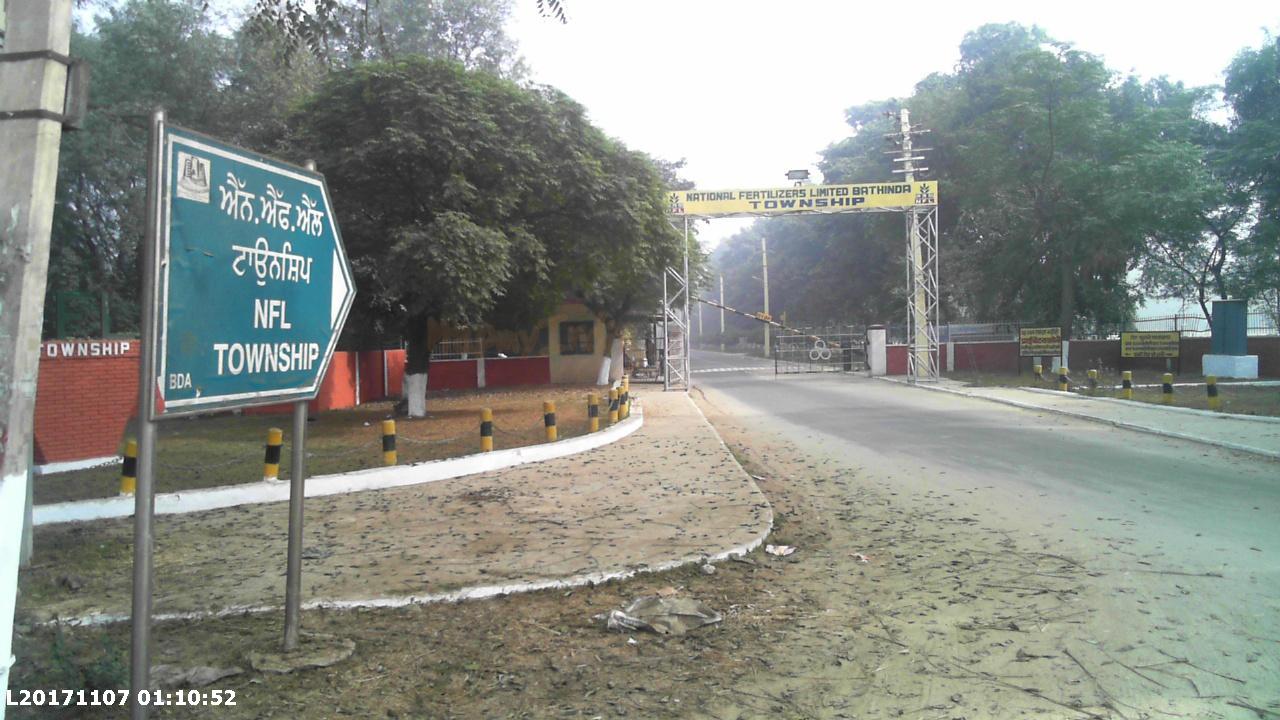

Supplement: Supplementary file 2 — Supplementary Material 2 [file 41598_2026_40742_MOESM2_ESM.zip › sample_data_yolov5/11-07 01.10.52.jpg]

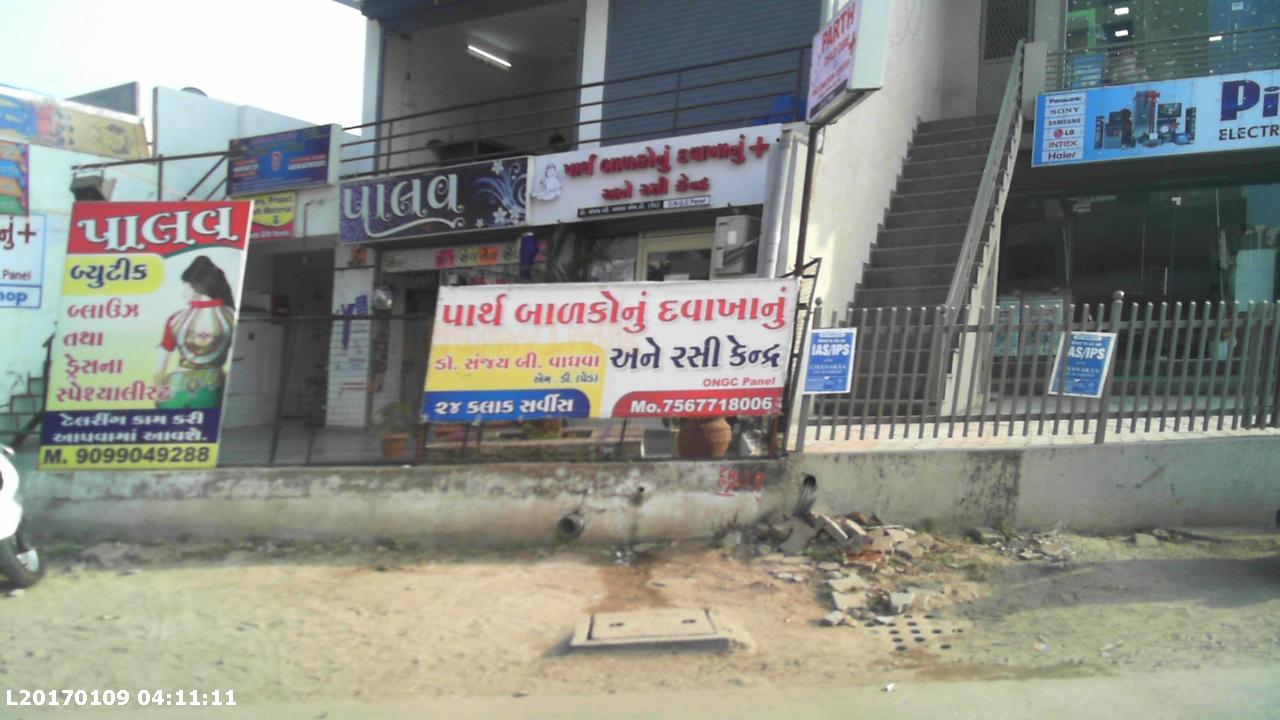

Supplement: Supplementary file 2 — Supplementary Material 2 [file 41598_2026_40742_MOESM2_ESM.zip › sample_data_yolov5/01-09 04.11.11.jpg]

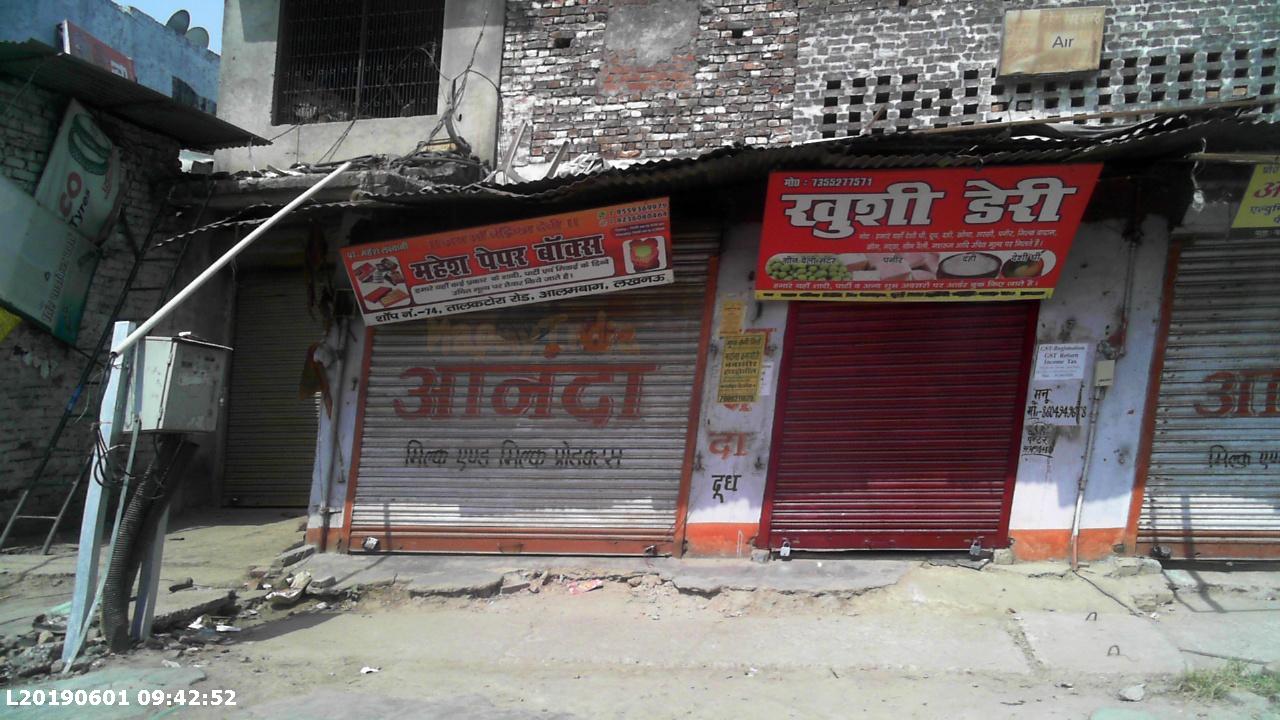

Supplement: Supplementary file 2 — Supplementary Material 2 [file 41598_2026_40742_MOESM2_ESM.zip › sample_data_yolov5/L_06-01_09.42.52.jpg]

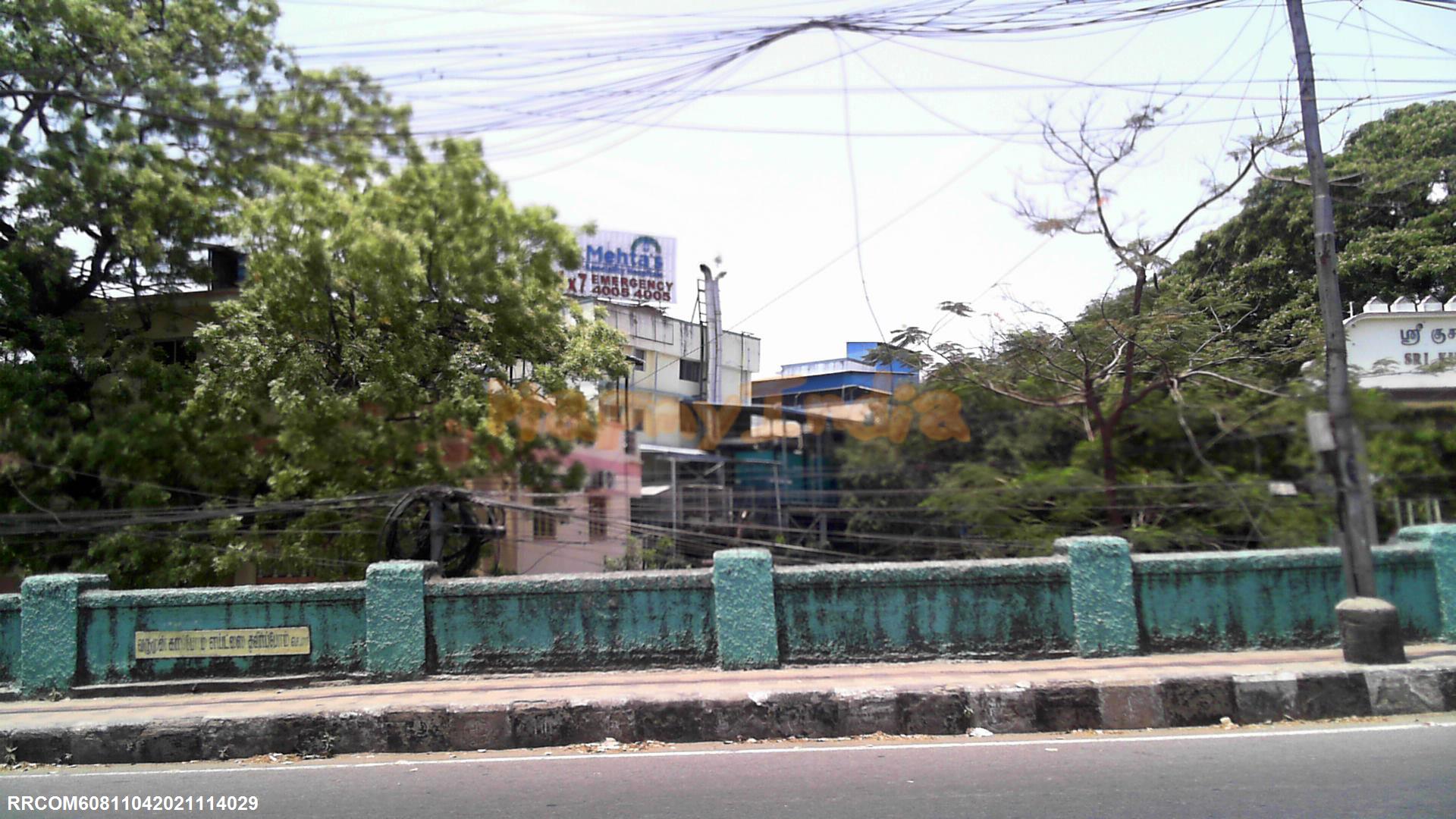

Supplement: Supplementary file 2 — Supplementary Material 2 [file 41598_2026_40742_MOESM2_ESM.zip › sample_data_yolov5/RCOM60811042021114029.jpg]

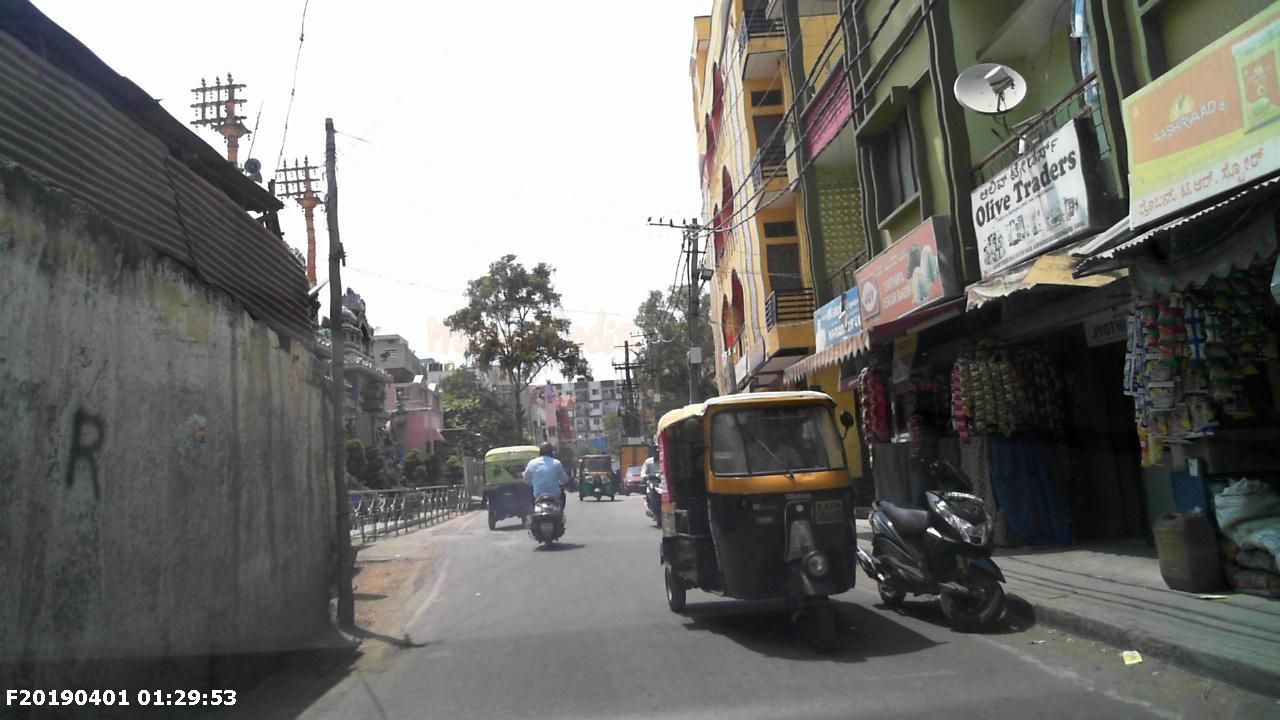

Supplement: Supplementary file 2 — Supplementary Material 2 [file 41598_2026_40742_MOESM2_ESM.zip › sample_data_yolov5/04-01_01.29.53.jpg]

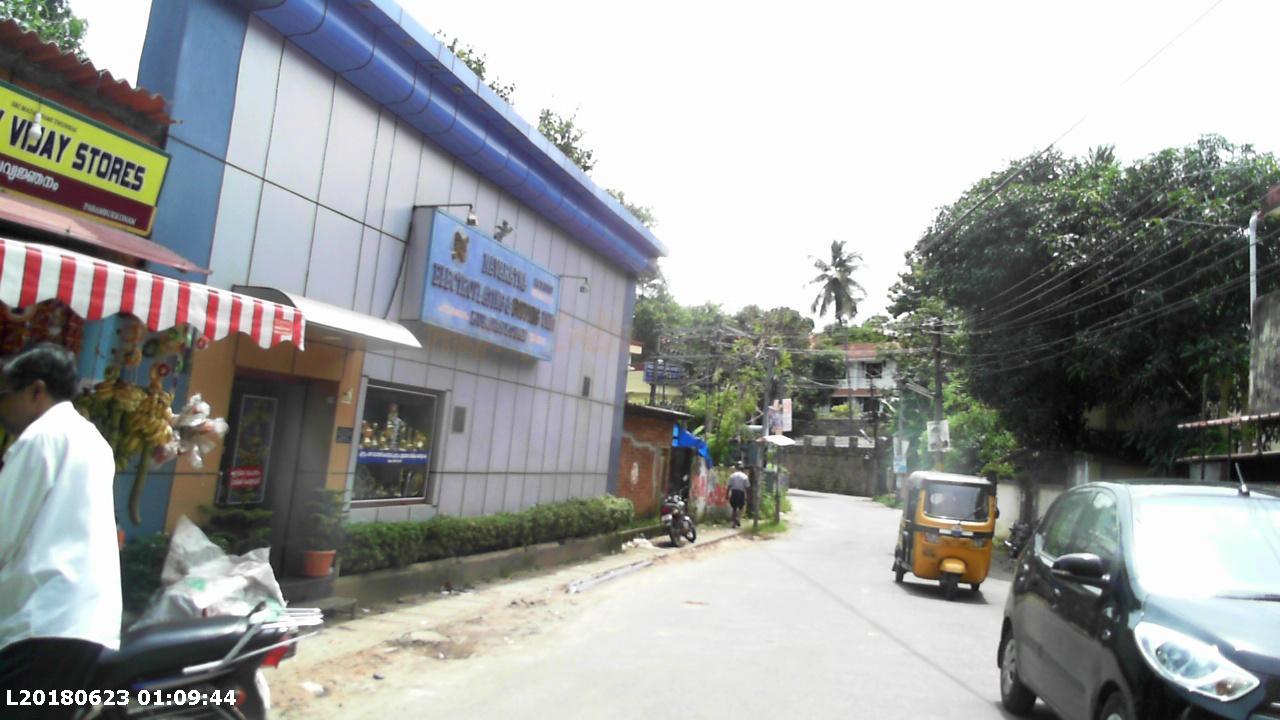

Supplement: Supplementary file 2 — Supplementary Material 2 [file 41598_2026_40742_MOESM2_ESM.zip › sample_data_yolov5/L_06-23_01.09.44.jpg]

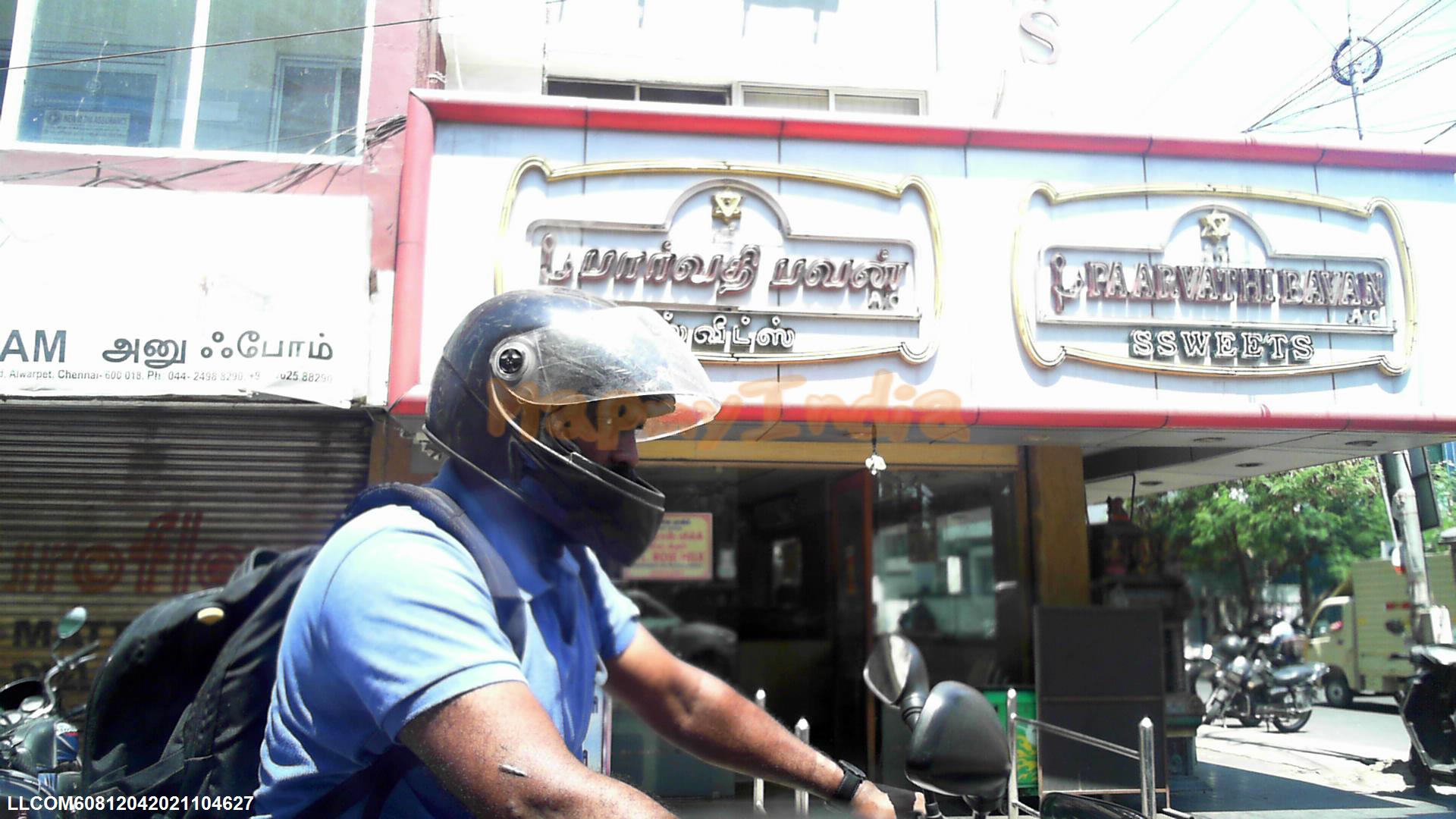

Supplement: Supplementary file 2 — Supplementary Material 2 [file 41598_2026_40742_MOESM2_ESM.zip › sample_data_yolov5/LCOM60812042021104627.jpg]

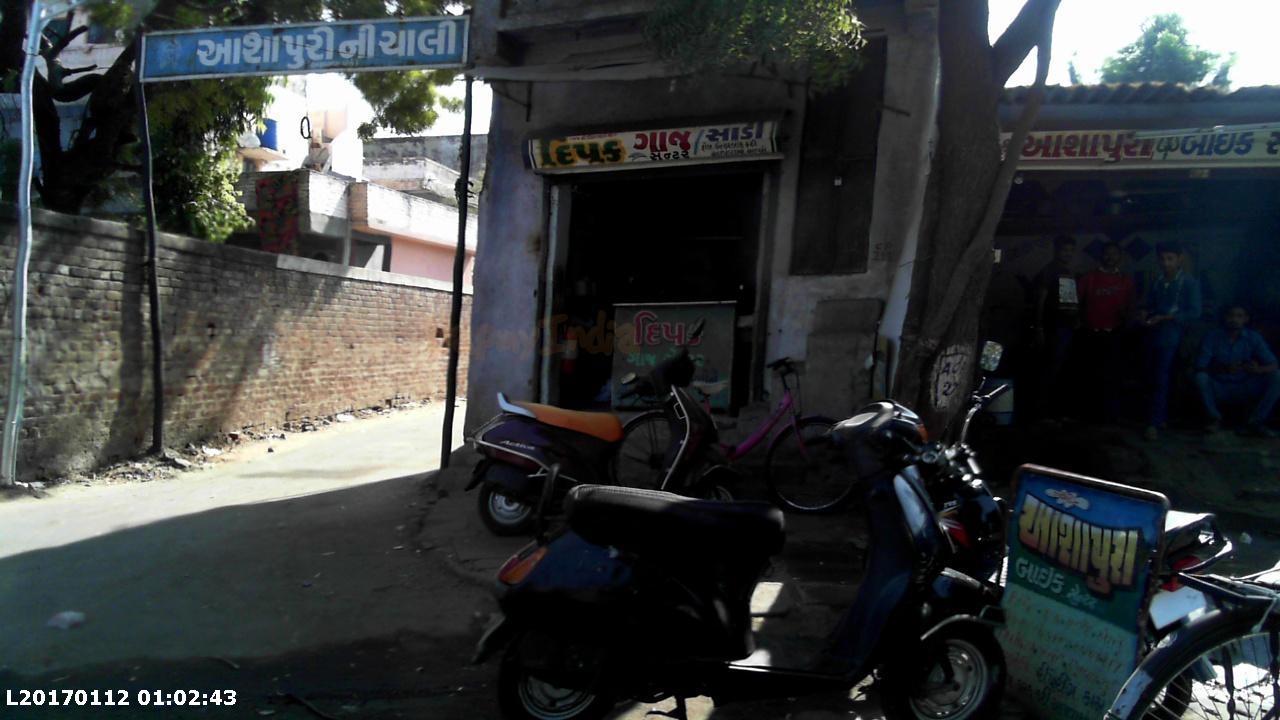

Supplement: Supplementary file 2 — Supplementary Material 2 [file 41598_2026_40742_MOESM2_ESM.zip › sample_data_yolov5/01-12 01.02.43.jpg]

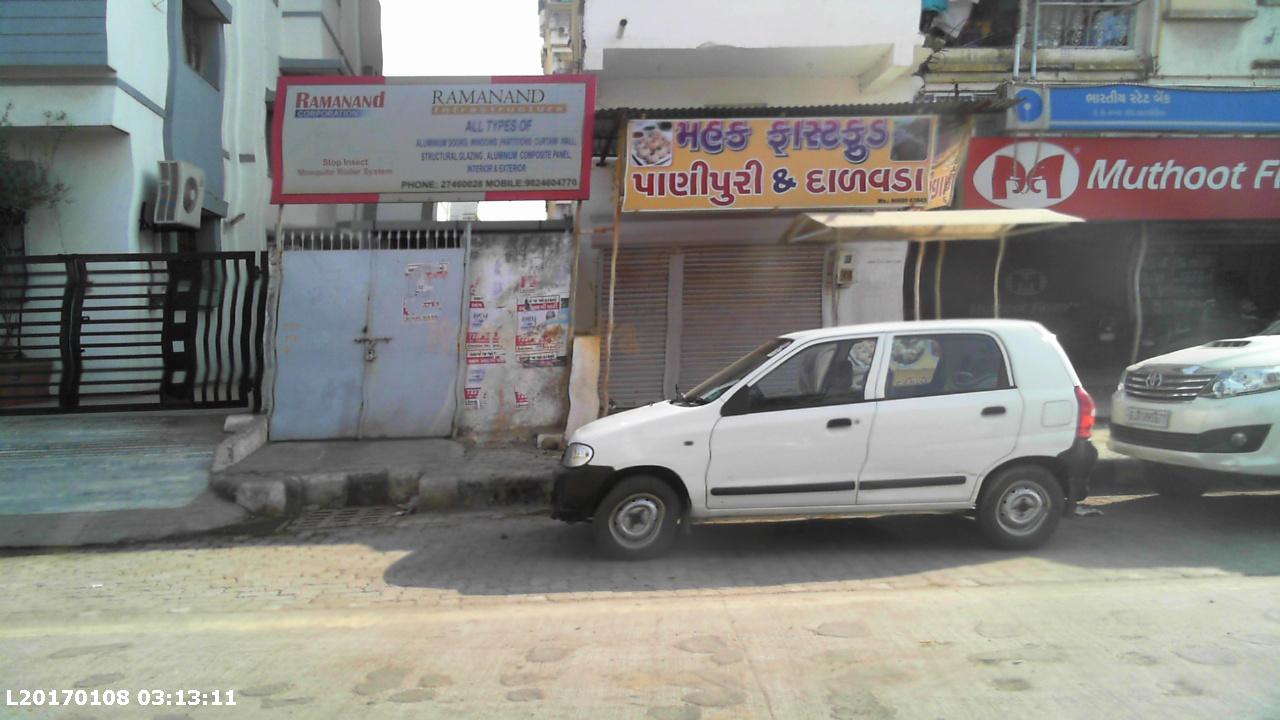

Supplement: Supplementary file 2 — Supplementary Material 2 [file 41598_2026_40742_MOESM2_ESM.zip › sample_data_yolov5/01-08 03.13.11.jpg]

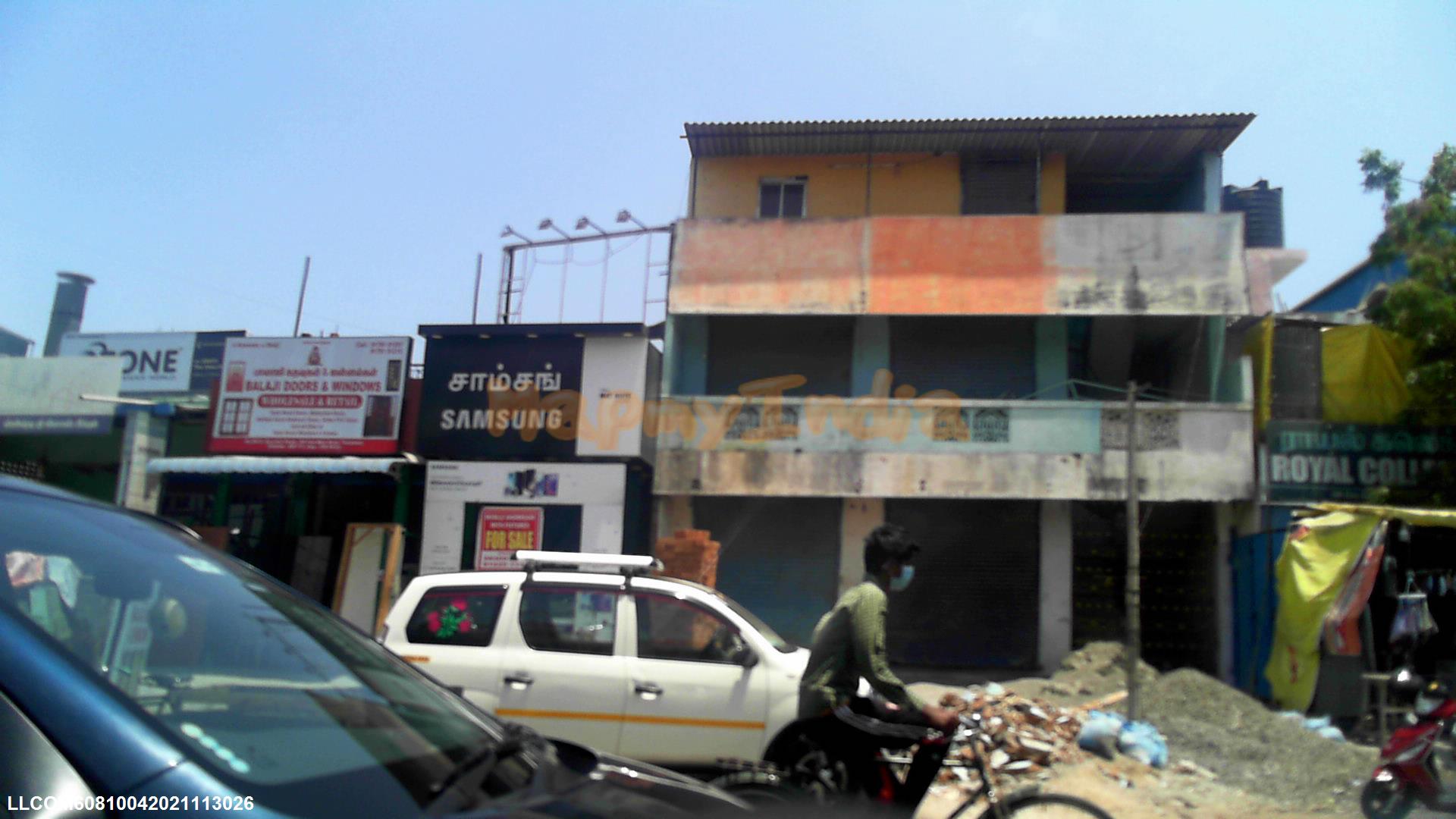

Supplement: Supplementary file 2 — Supplementary Material 2 [file 41598_2026_40742_MOESM2_ESM.zip › sample_data_yolov5/LCOM60810042021113026.jpg]

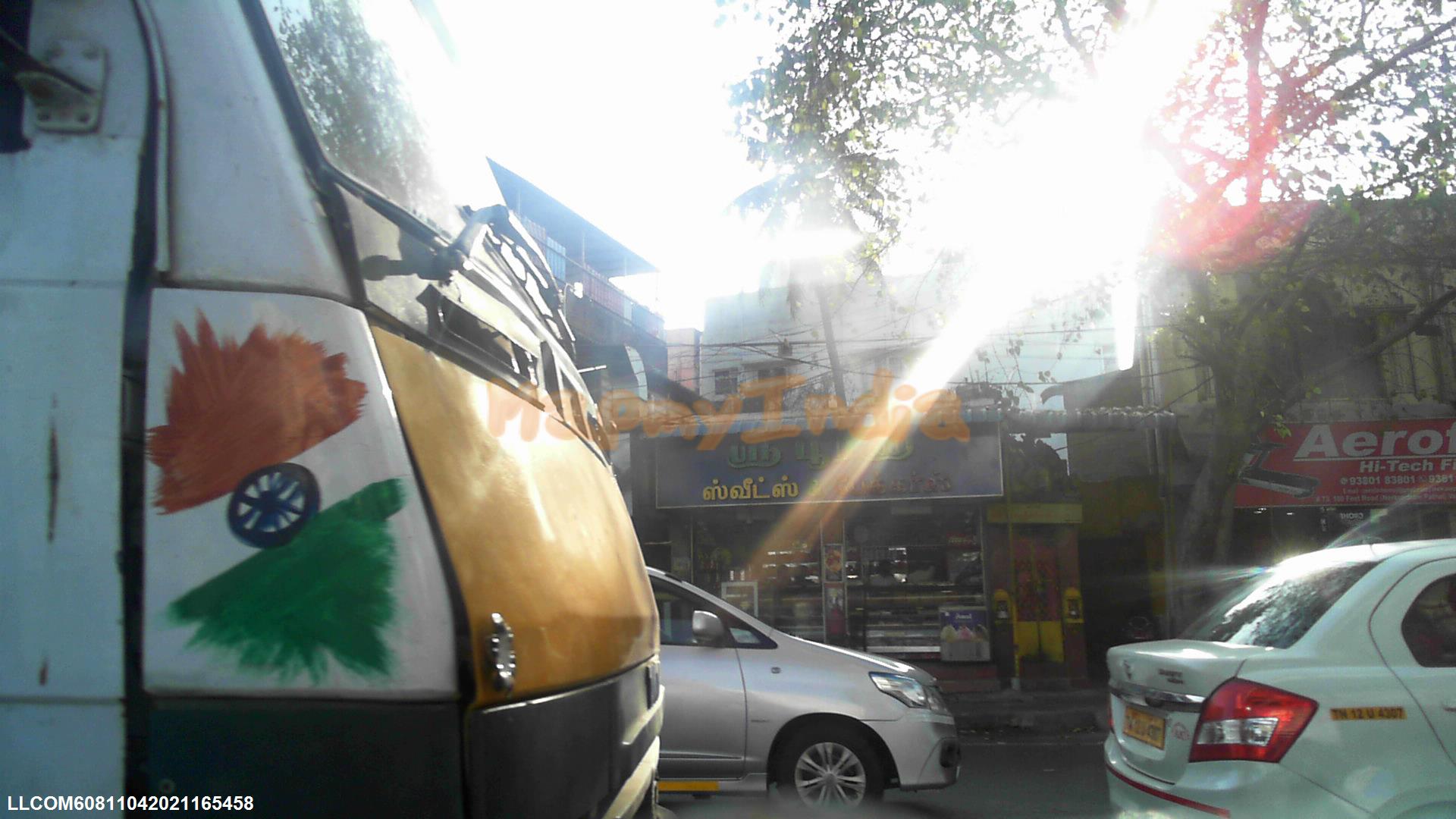

Supplement: Supplementary file 2 — Supplementary Material 2 [file 41598_2026_40742_MOESM2_ESM.zip › sample_data_yolov5/LCOM60811042021165458.jpg]

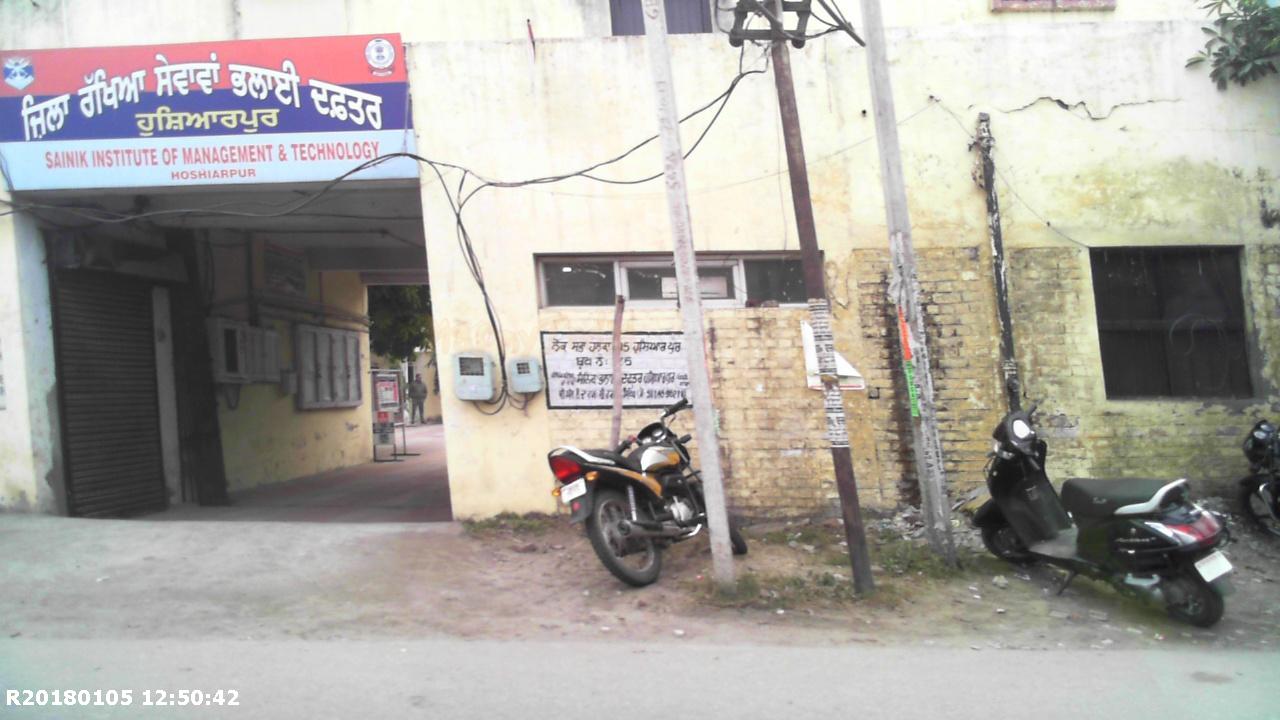

Supplement: Supplementary file 2 — Supplementary Material 2 [file 41598_2026_40742_MOESM2_ESM.zip › sample_data_yolov5/01-05 12.50.42.jpg]

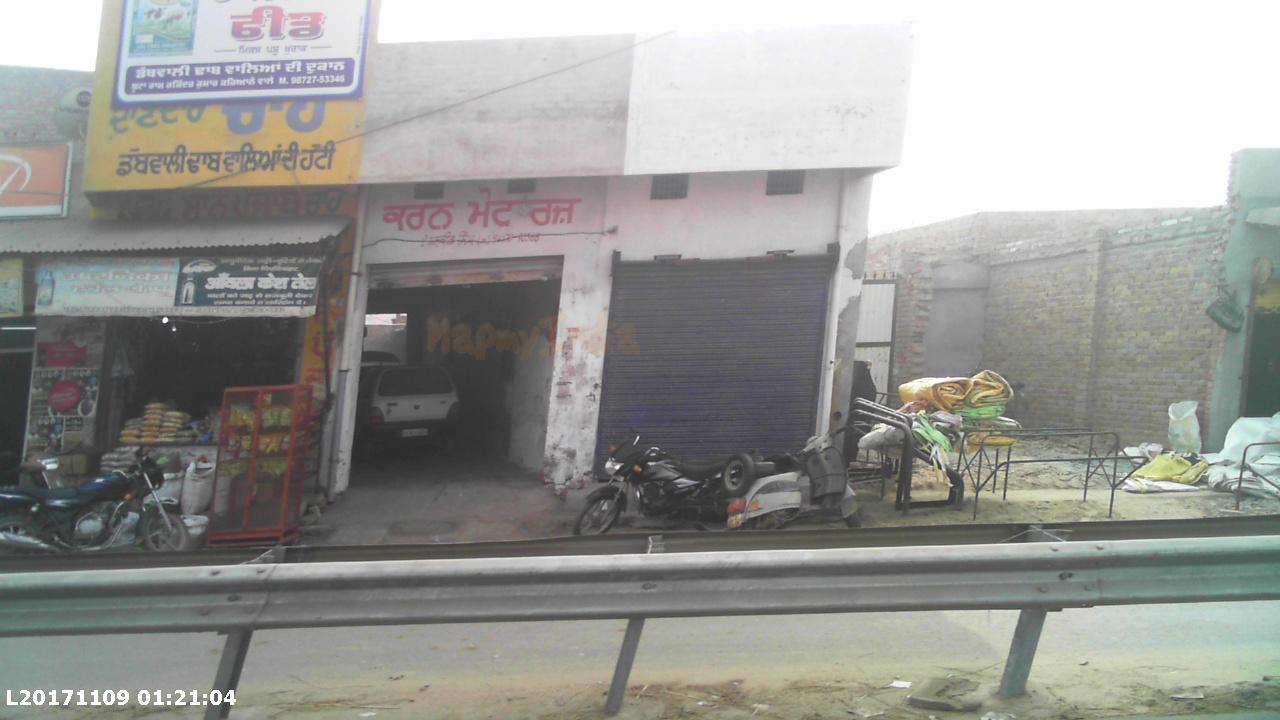

Supplement: Supplementary file 2 — Supplementary Material 2 [file 41598_2026_40742_MOESM2_ESM.zip › sample_data_yolov5/11-09 01.21.04.jpg]
