# Supplementary figures and images for: Integrated framework utilizing scene text detection and recognition techniques for enhancing point of interest extraction from name boards in all Indic languages (part 1 of 2)
Source: Sci Rep. 2026 Mar 10;16:12907. doi: 10.1038/s41598-026-40742-w (PMC13096107; doi:10.1038/s41598-026-40742-w)

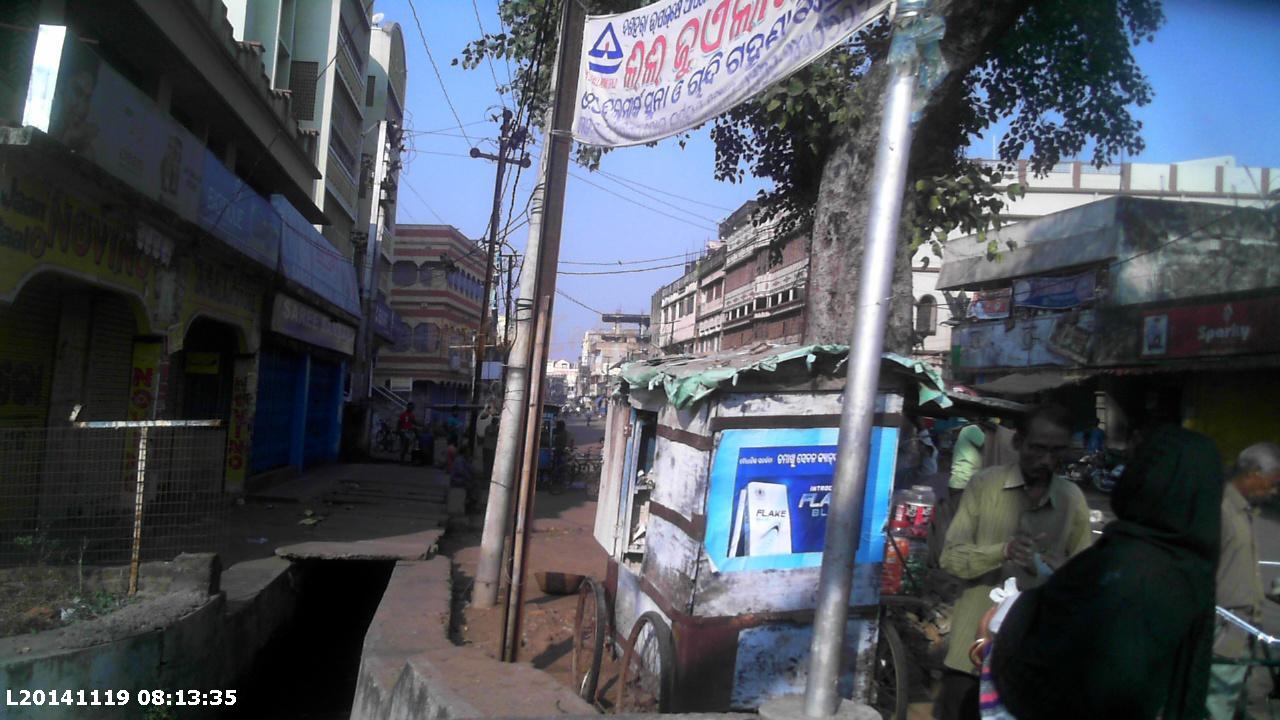

Supplement: Supplementary file 2 — Supplementary Material 2 [file 41598_2026_40742_MOESM2_ESM.zip › sample_data_yolov5/L_11-19_08.13.35.jpg]

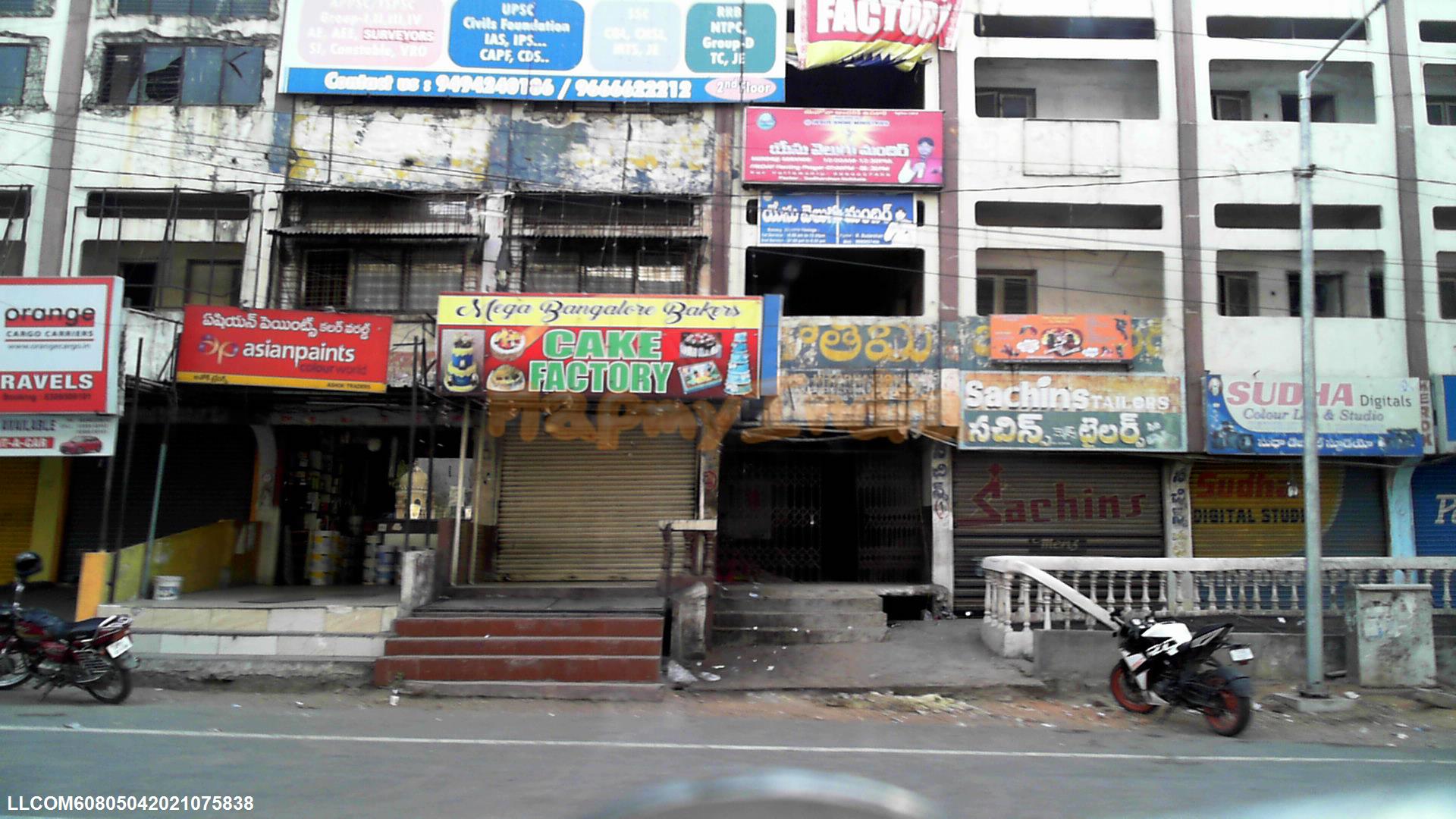

Supplement: Supplementary file 2 — Supplementary Material 2 [file 41598_2026_40742_MOESM2_ESM.zip › sample_data_yolov5/LCOM60805042021075838.jpg]

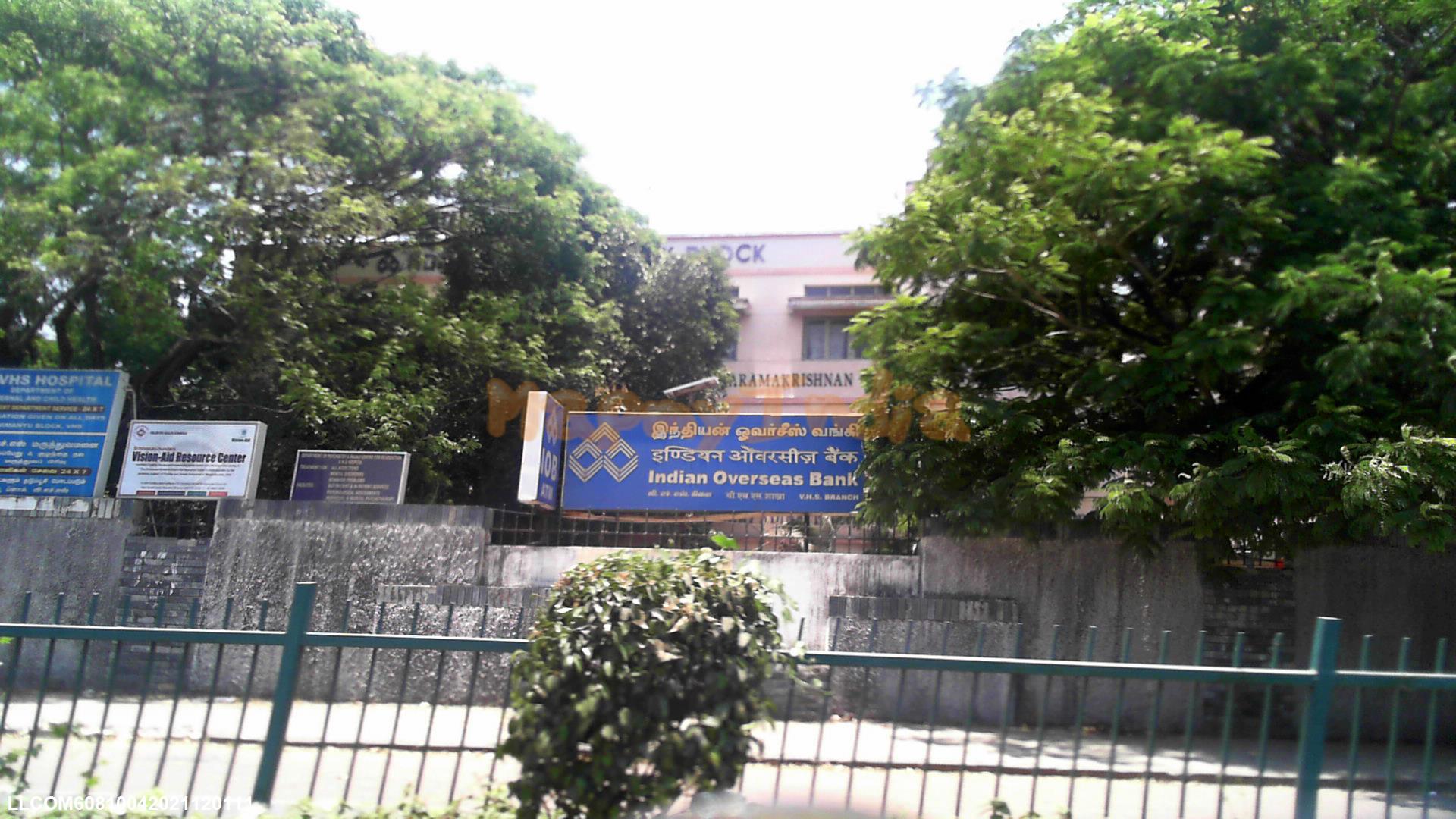

Supplement: Supplementary file 2 — Supplementary Material 2 [file 41598_2026_40742_MOESM2_ESM.zip › sample_data_yolov5/LCOM60810042021120111.jpg]

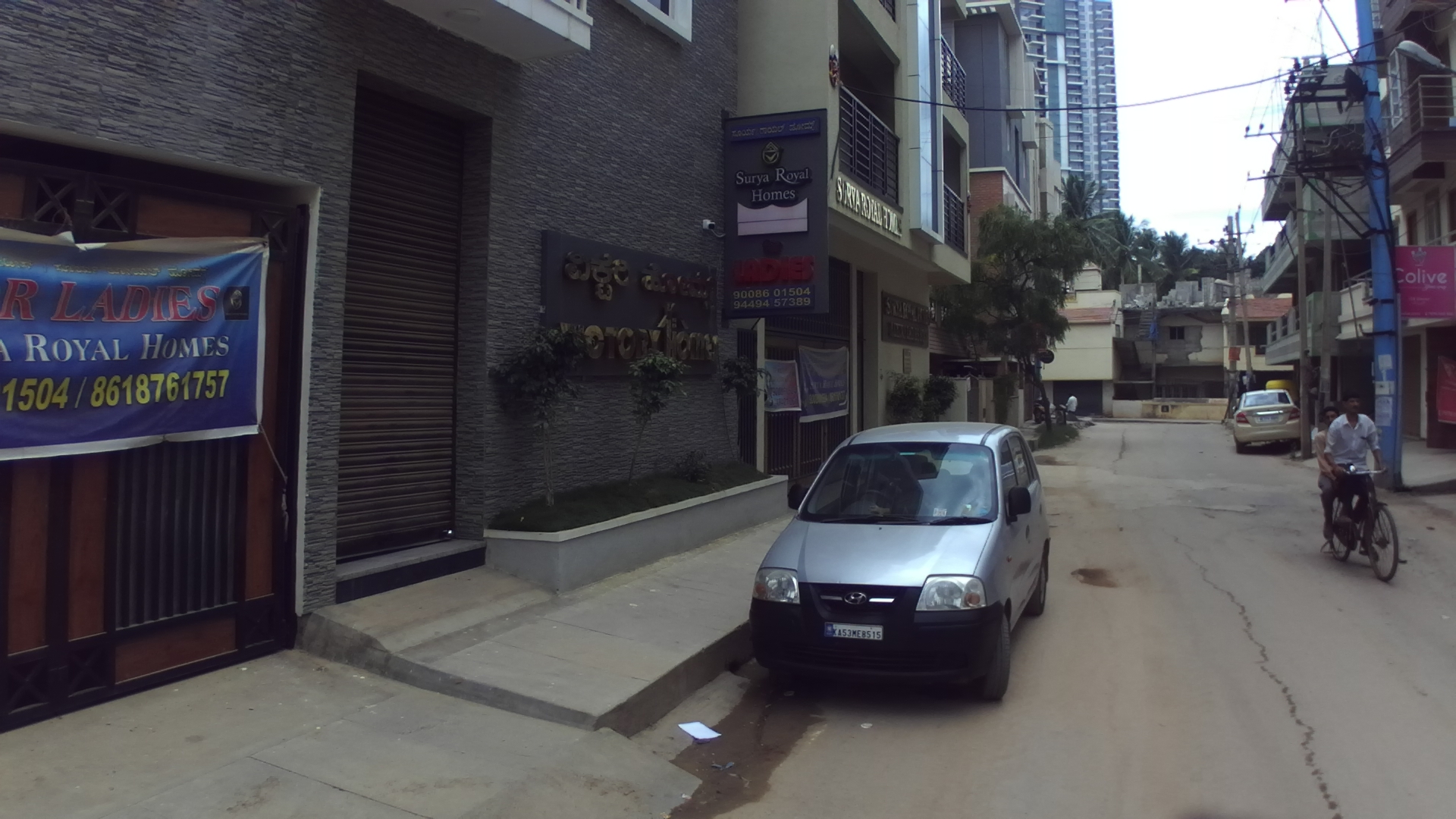

Supplement: Supplementary file 2 — Supplementary Material 2 [file 41598_2026_40742_MOESM2_ESM.zip › sample_data_yolov5/T1_051020_123506_24377_zed_l_347.jpg]

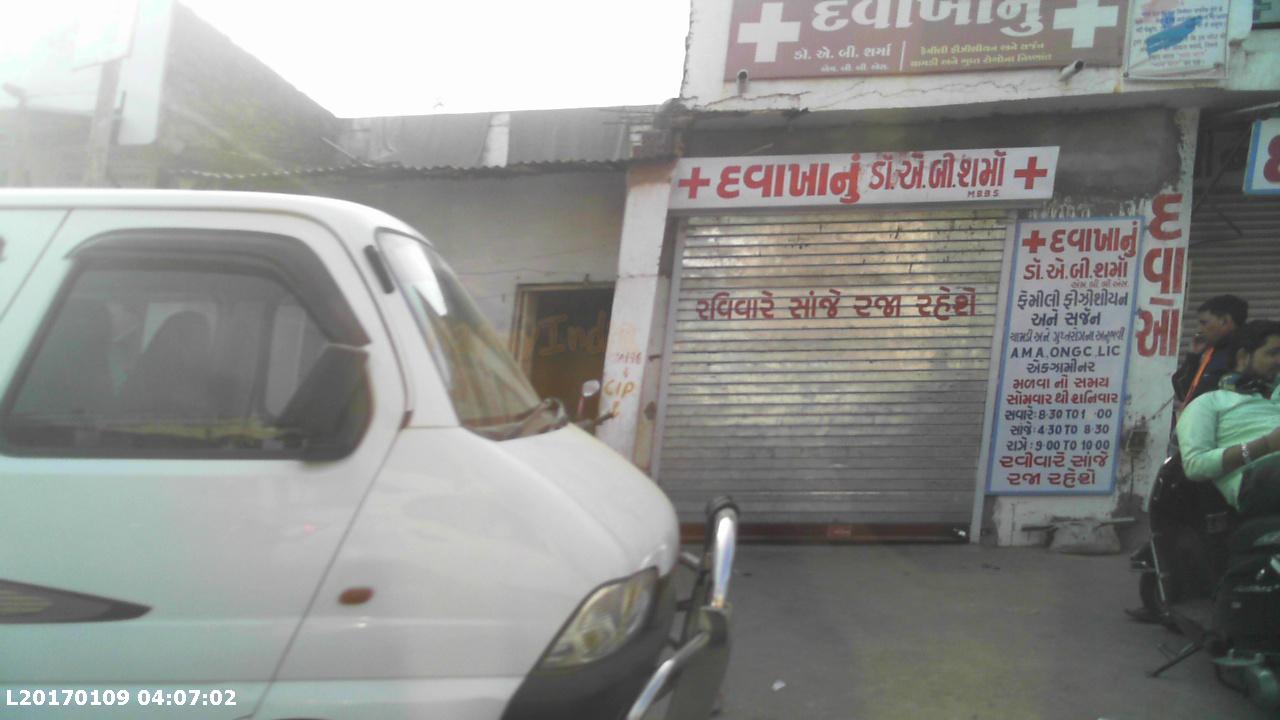

Supplement: Supplementary file 2 — Supplementary Material 2 [file 41598_2026_40742_MOESM2_ESM.zip › sample_data_yolov5/01-09 04.07.02.jpg]

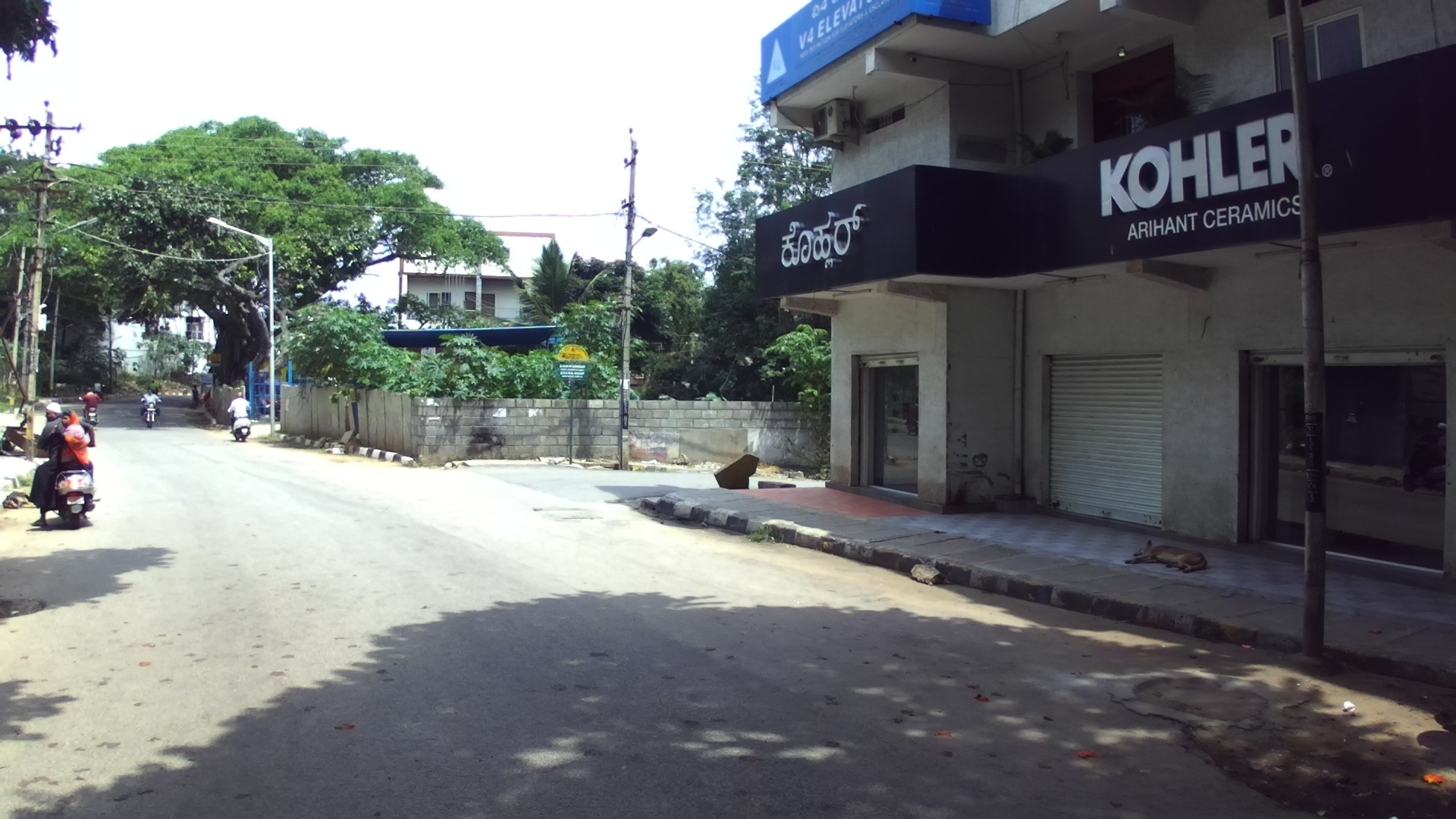

Supplement: Supplementary file 2 — Supplementary Material 2 [file 41598_2026_40742_MOESM2_ESM.zip › sample_data_yolov5/T1_051020_114321_24376_zed_l_618.jpg]

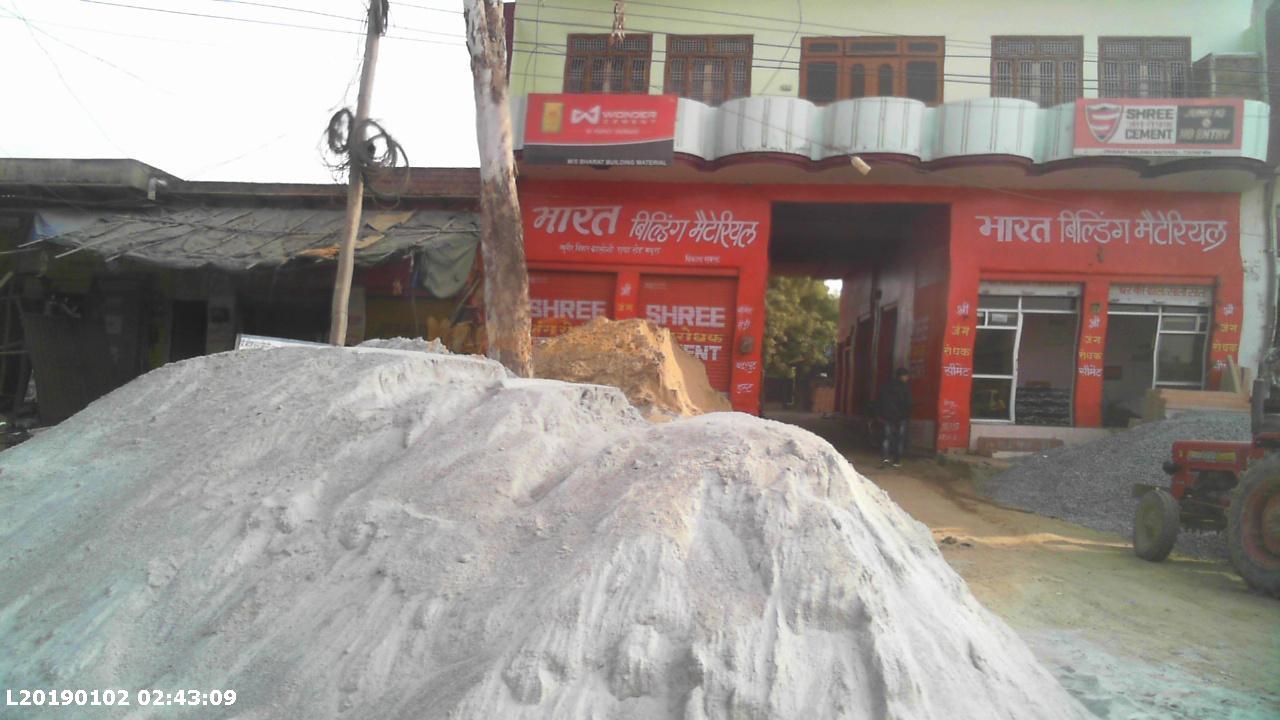

Supplement: Supplementary file 2 — Supplementary Material 2 [file 41598_2026_40742_MOESM2_ESM.zip › sample_data_yolov5/L_01-02_02.43.09.jpg]

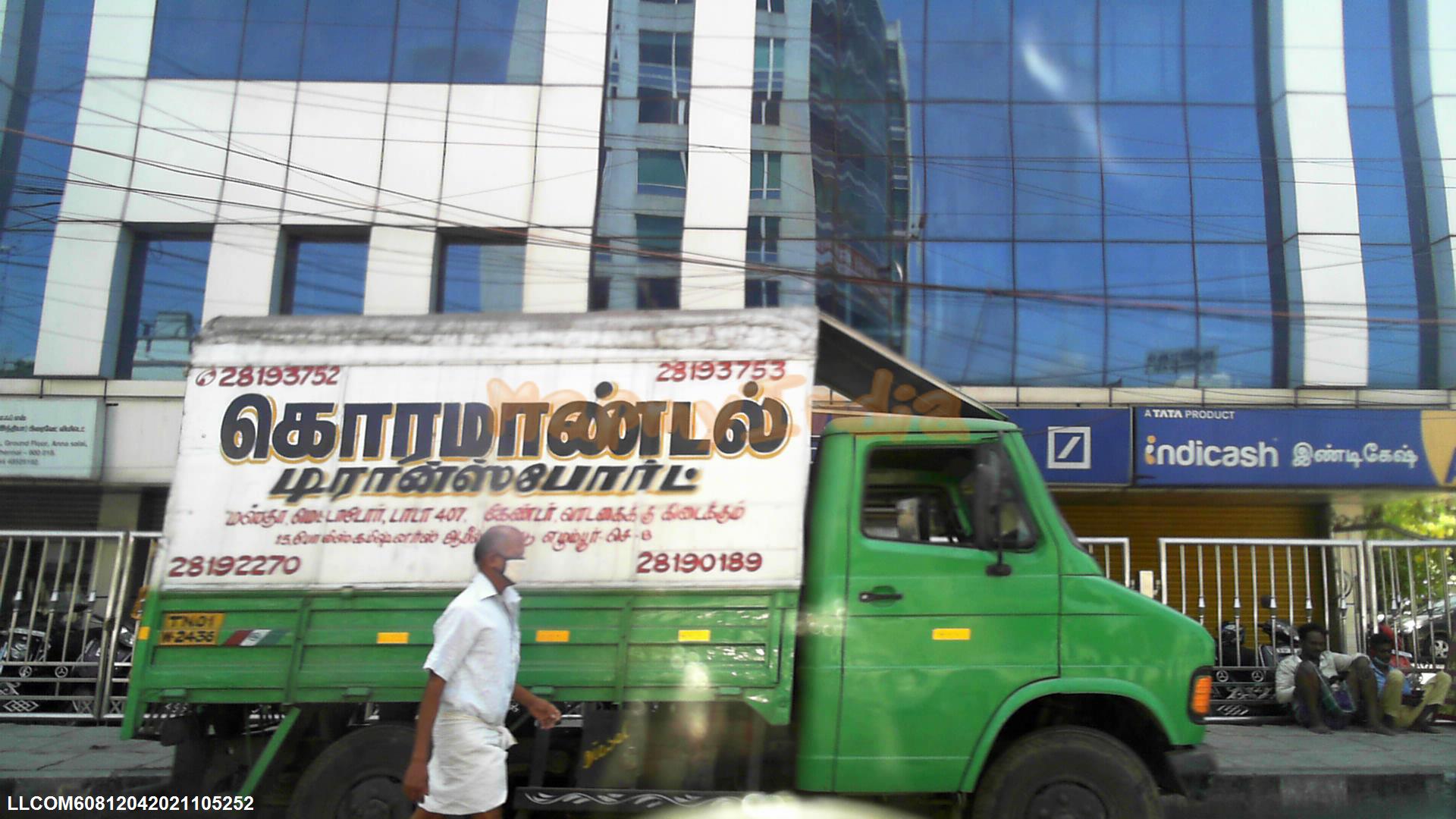

Supplement: Supplementary file 2 — Supplementary Material 2 [file 41598_2026_40742_MOESM2_ESM.zip › sample_data_yolov5/LCOM60812042021105252.jpg]

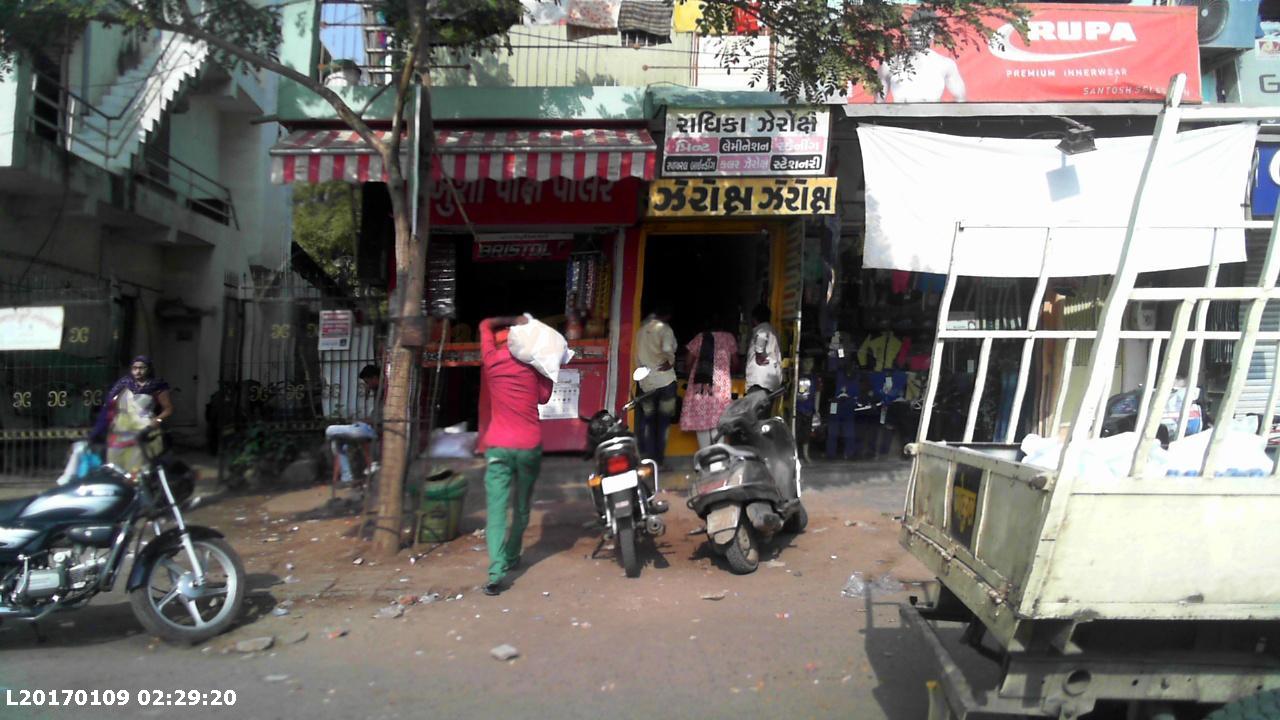

Supplement: Supplementary file 2 — Supplementary Material 2 [file 41598_2026_40742_MOESM2_ESM.zip › sample_data_yolov5/01-09 02.29.20.jpg]

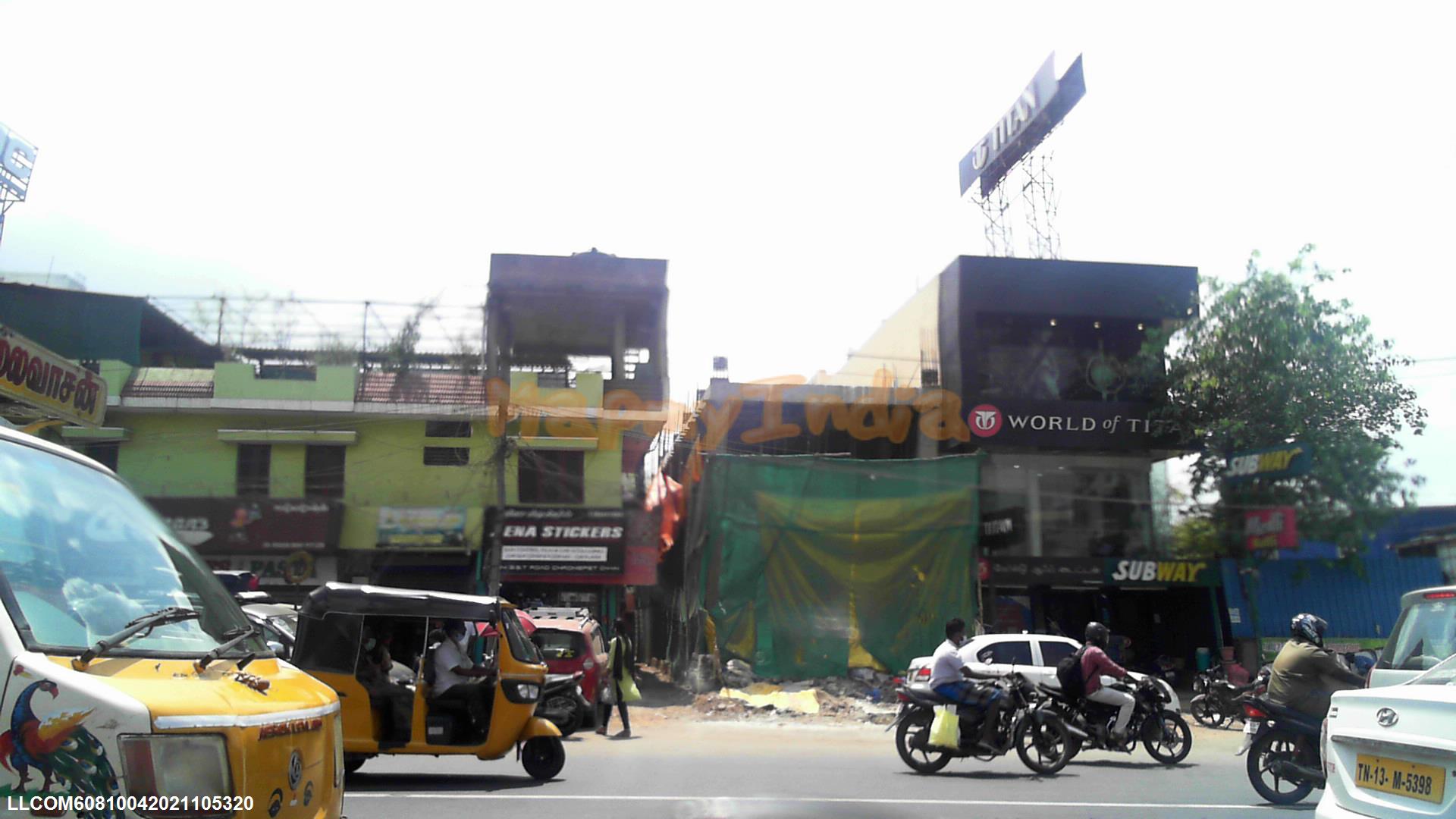

Supplement: Supplementary file 2 — Supplementary Material 2 [file 41598_2026_40742_MOESM2_ESM.zip › sample_data_yolov5/LCOM60810042021105320.jpg]

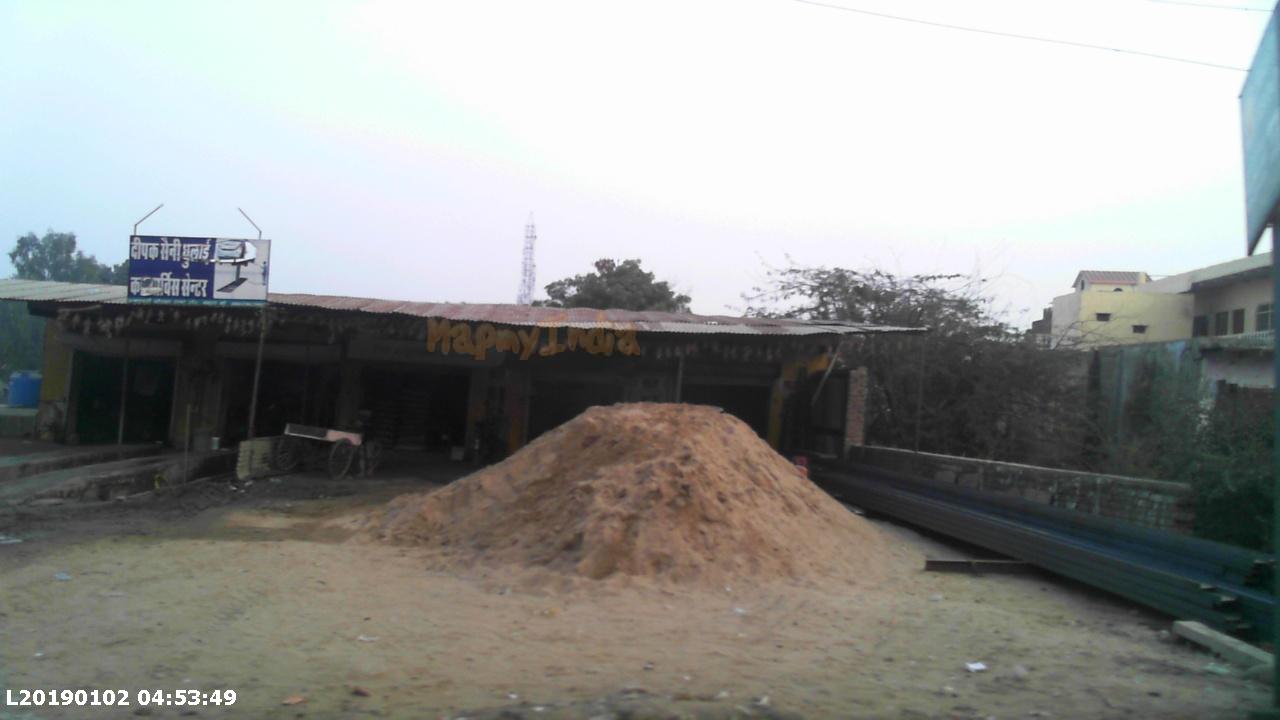

Supplement: Supplementary file 2 — Supplementary Material 2 [file 41598_2026_40742_MOESM2_ESM.zip › sample_data_yolov5/L_01-02_04.53.49.jpg]

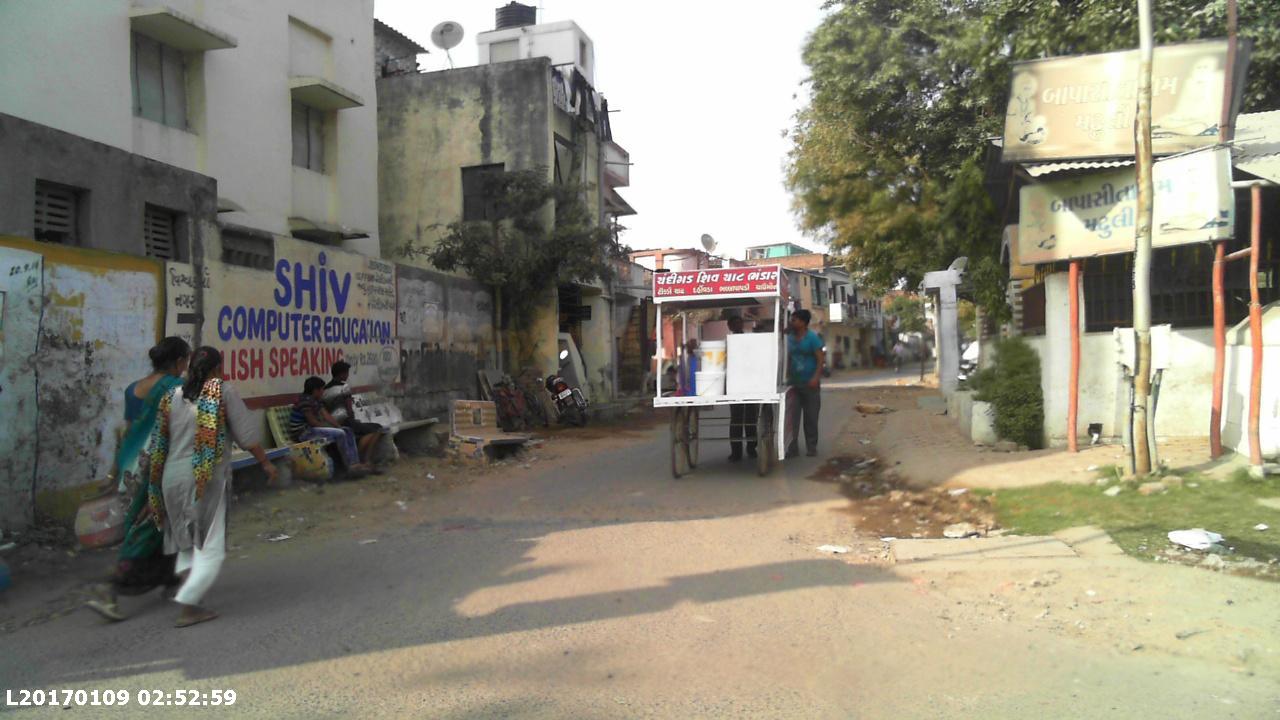

Supplement: Supplementary file 2 — Supplementary Material 2 [file 41598_2026_40742_MOESM2_ESM.zip › sample_data_yolov5/01-09 02.52.59.jpg]

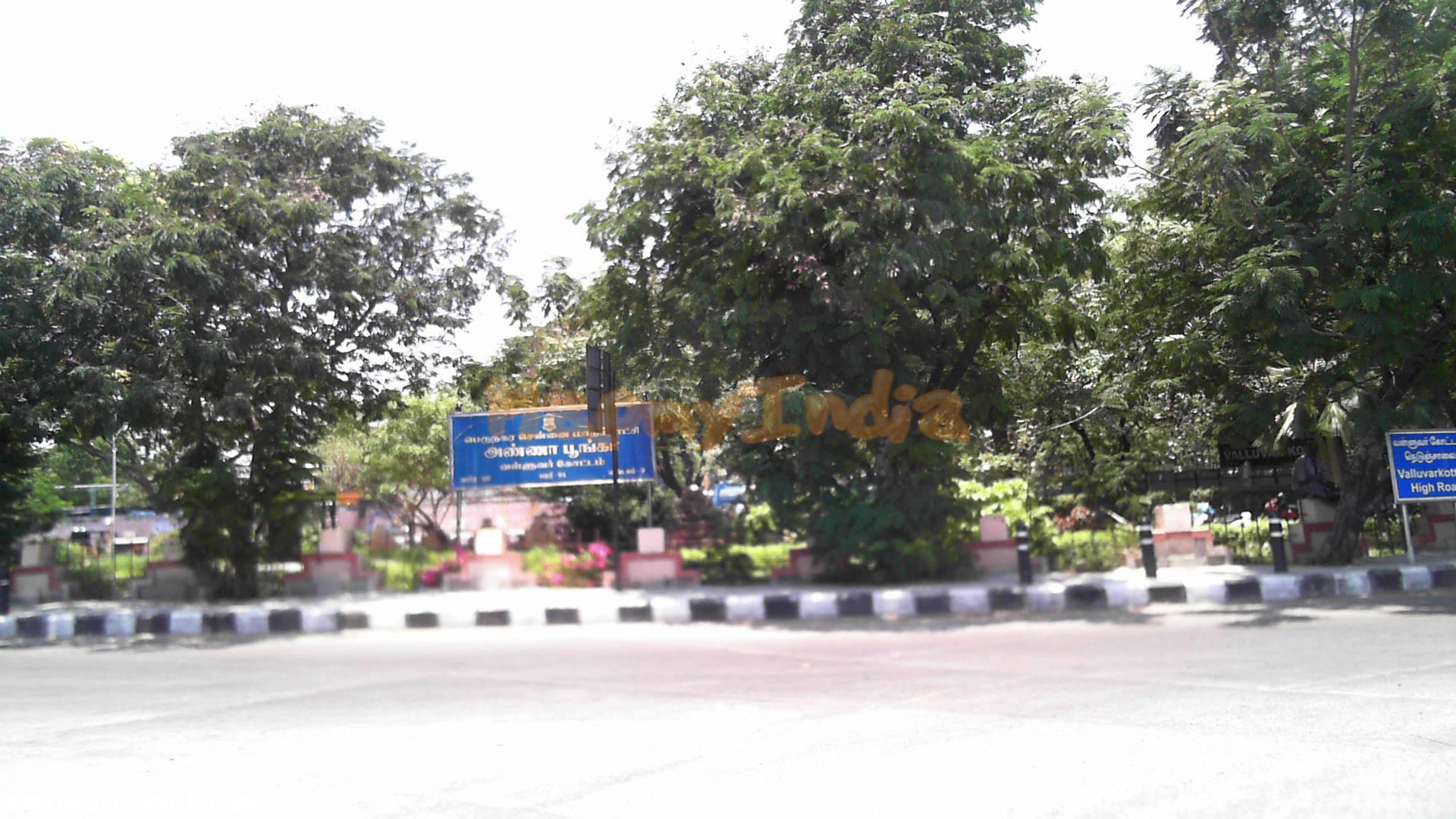

Supplement: Supplementary file 2 — Supplementary Material 2 [file 41598_2026_40742_MOESM2_ESM.zip › sample_data_yolov5/RCOM60811042021115405.jpg]

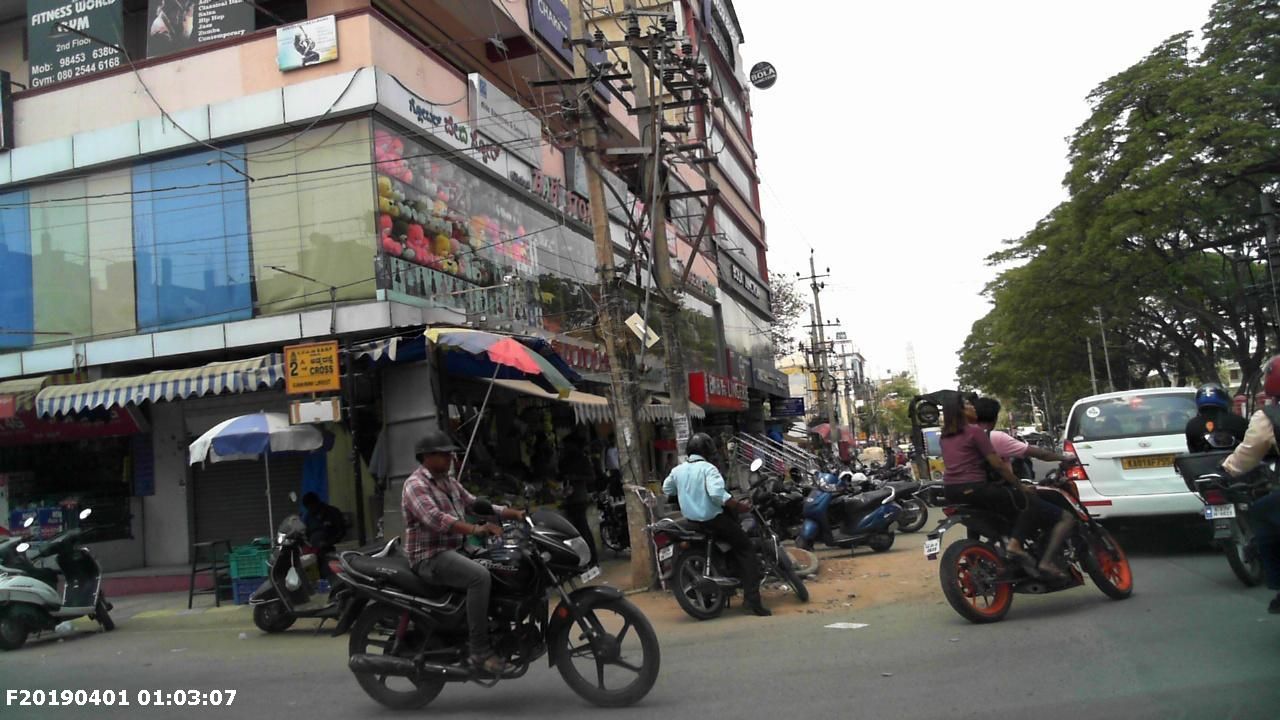

Supplement: Supplementary file 2 — Supplementary Material 2 [file 41598_2026_40742_MOESM2_ESM.zip › sample_data_yolov5/04-01_01.03.07.jpg]

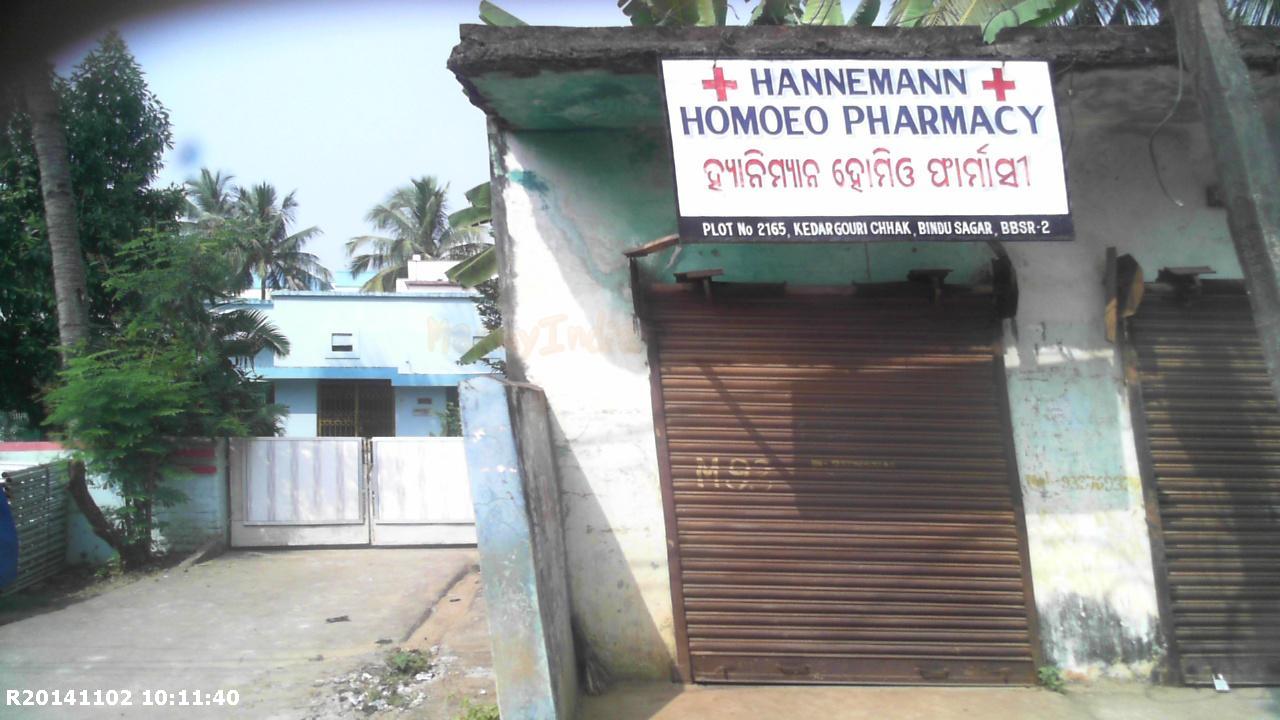

Supplement: Supplementary file 2 — Supplementary Material 2 [file 41598_2026_40742_MOESM2_ESM.zip › sample_data_yolov5/R_11-02_10.11.40.jpg]

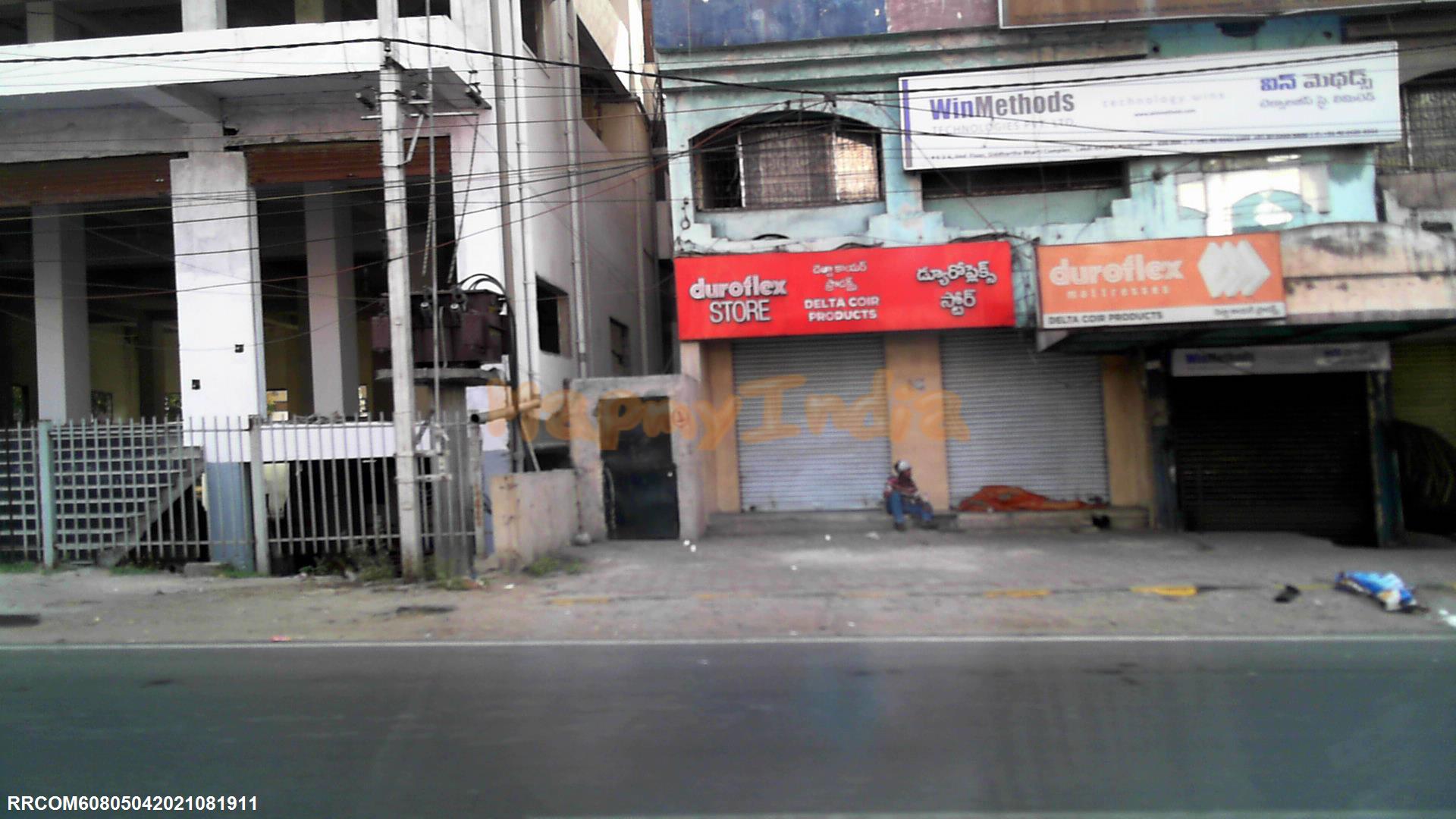

Supplement: Supplementary file 2 — Supplementary Material 2 [file 41598_2026_40742_MOESM2_ESM.zip › sample_data_yolov5/RCOM60805042021081911.jpg]

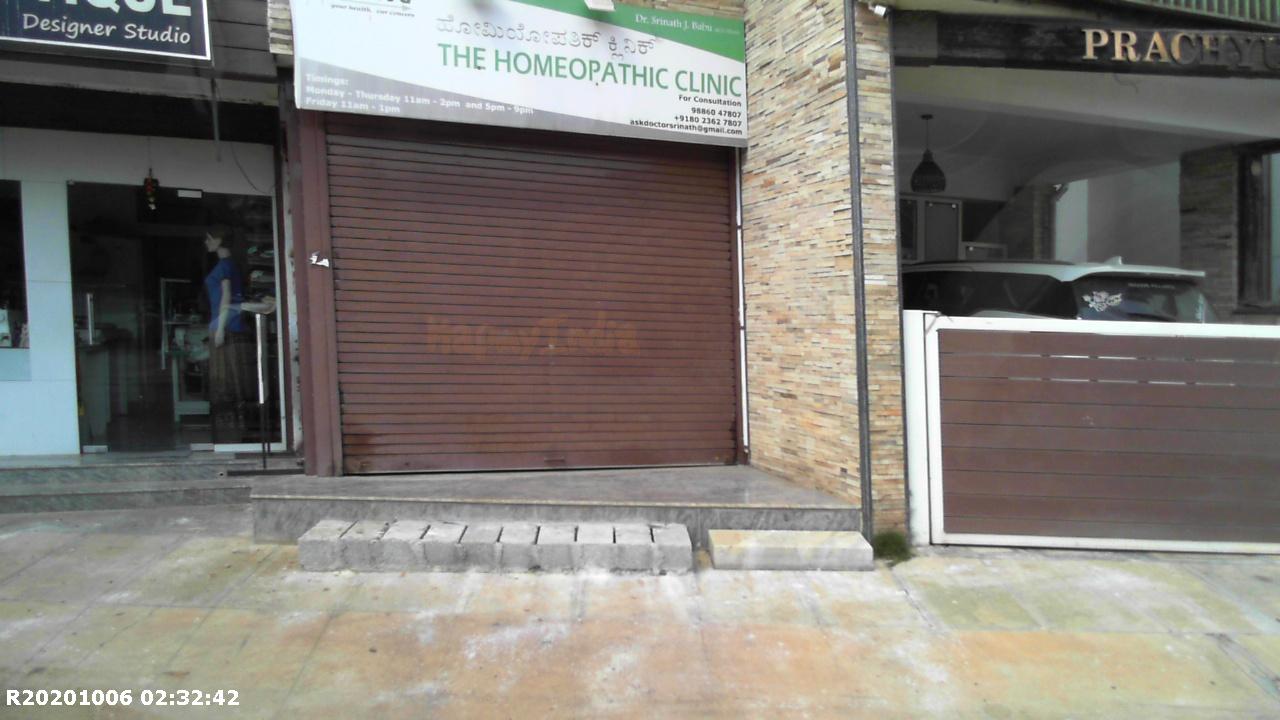

Supplement: Supplementary file 2 — Supplementary Material 2 [file 41598_2026_40742_MOESM2_ESM.zip › sample_data_yolov5/T99_061020_143242_99991_acs_r_001.jpg]

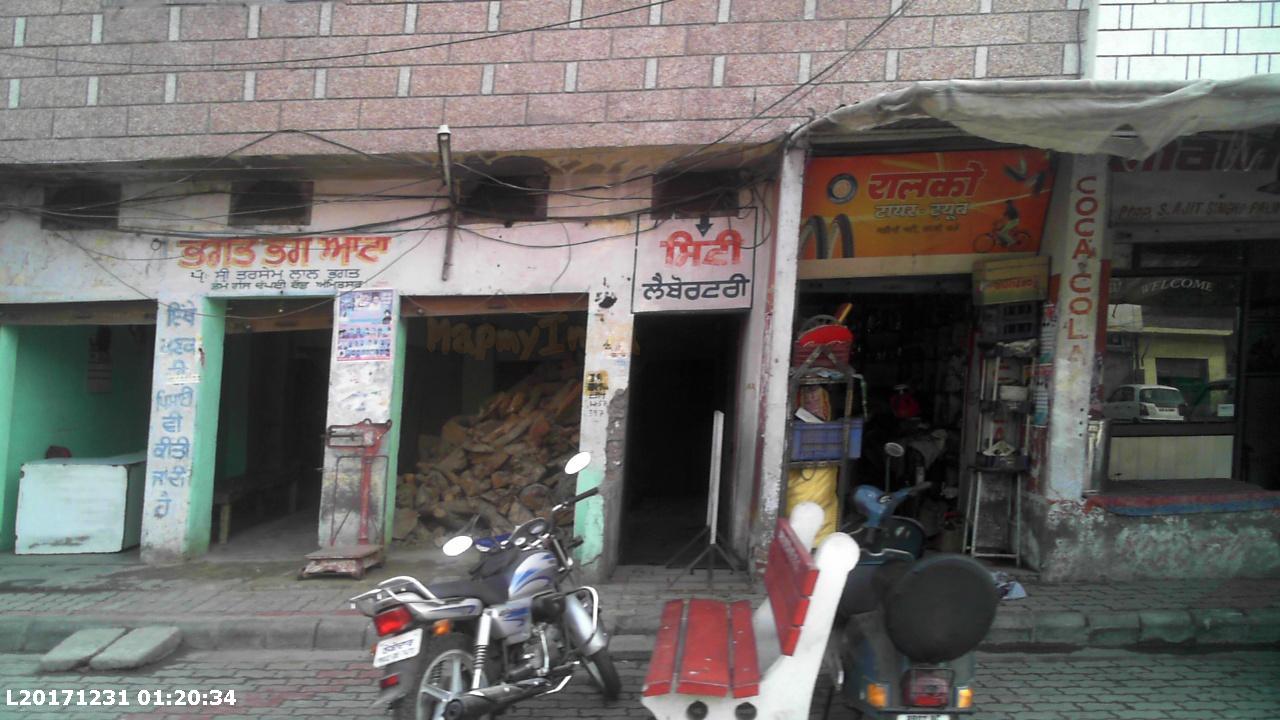

Supplement: Supplementary file 2 — Supplementary Material 2 [file 41598_2026_40742_MOESM2_ESM.zip › sample_data_yolov5/12-31 01.20.34.jpg]

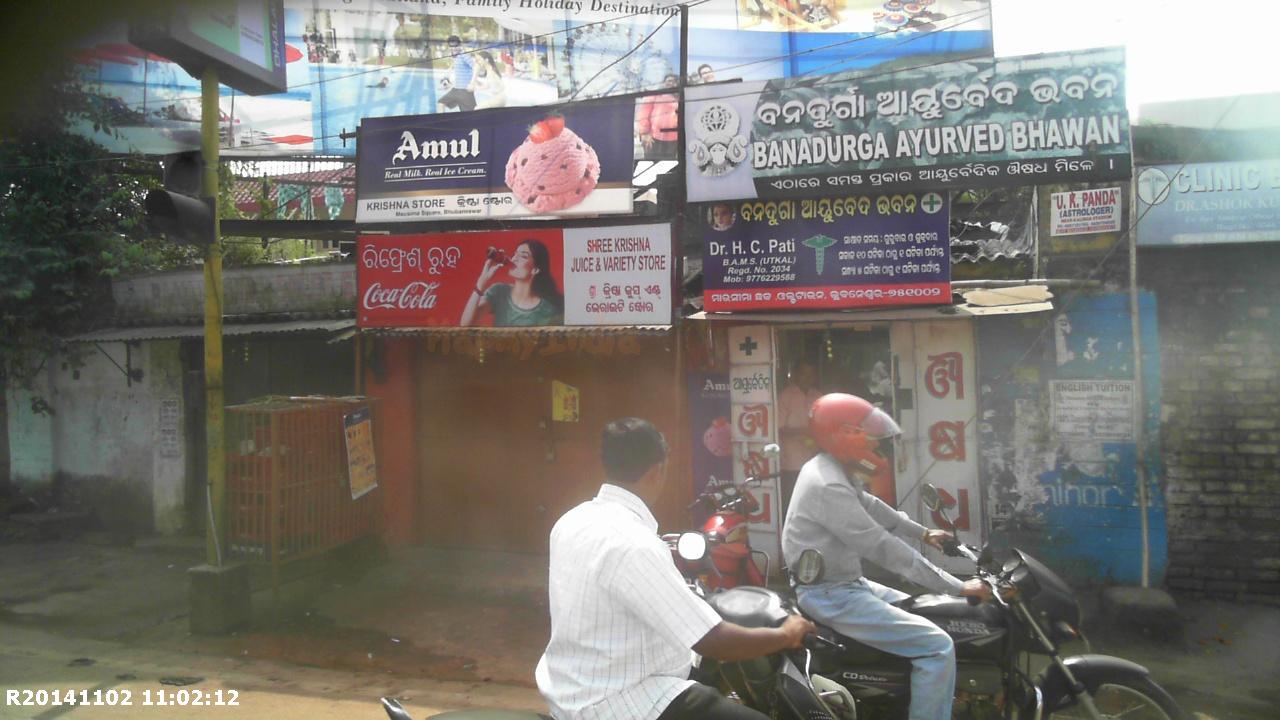

Supplement: Supplementary file 2 — Supplementary Material 2 [file 41598_2026_40742_MOESM2_ESM.zip › sample_data_yolov5/R_11-02_11.02.12.jpg]

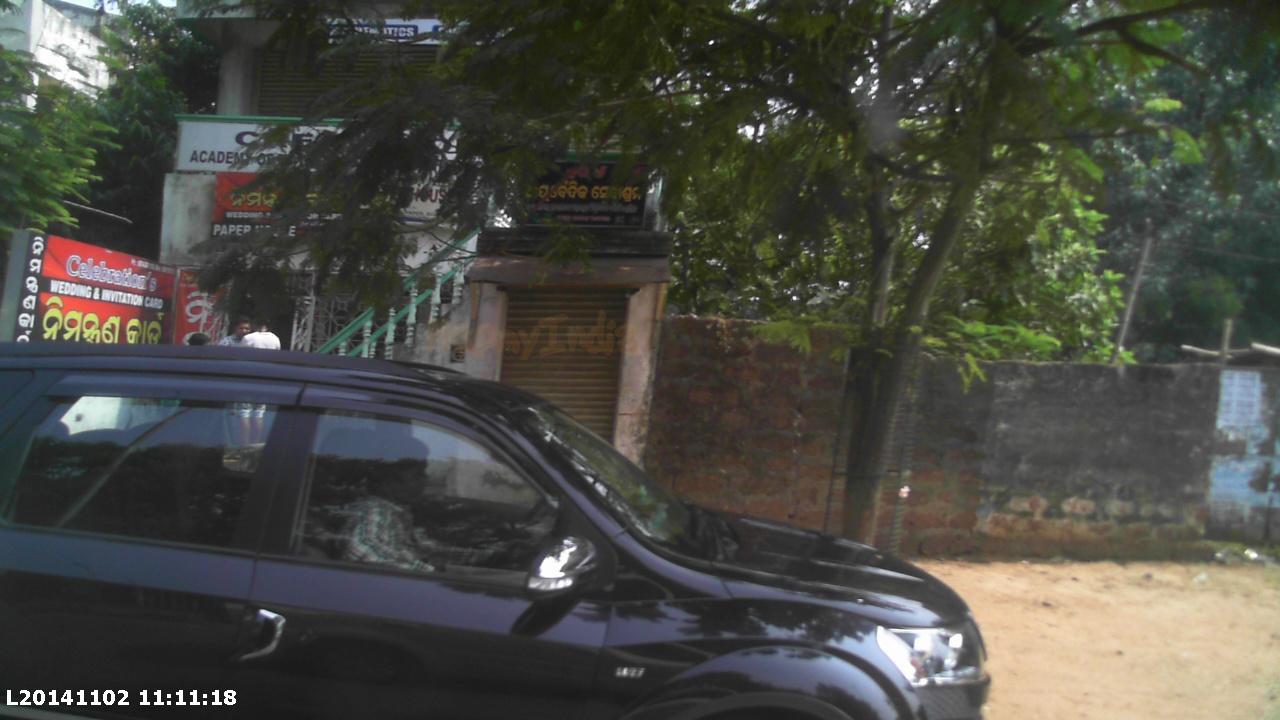

Supplement: Supplementary file 2 — Supplementary Material 2 [file 41598_2026_40742_MOESM2_ESM.zip › sample_data_yolov5/L_11-02_11.11.18.jpg]

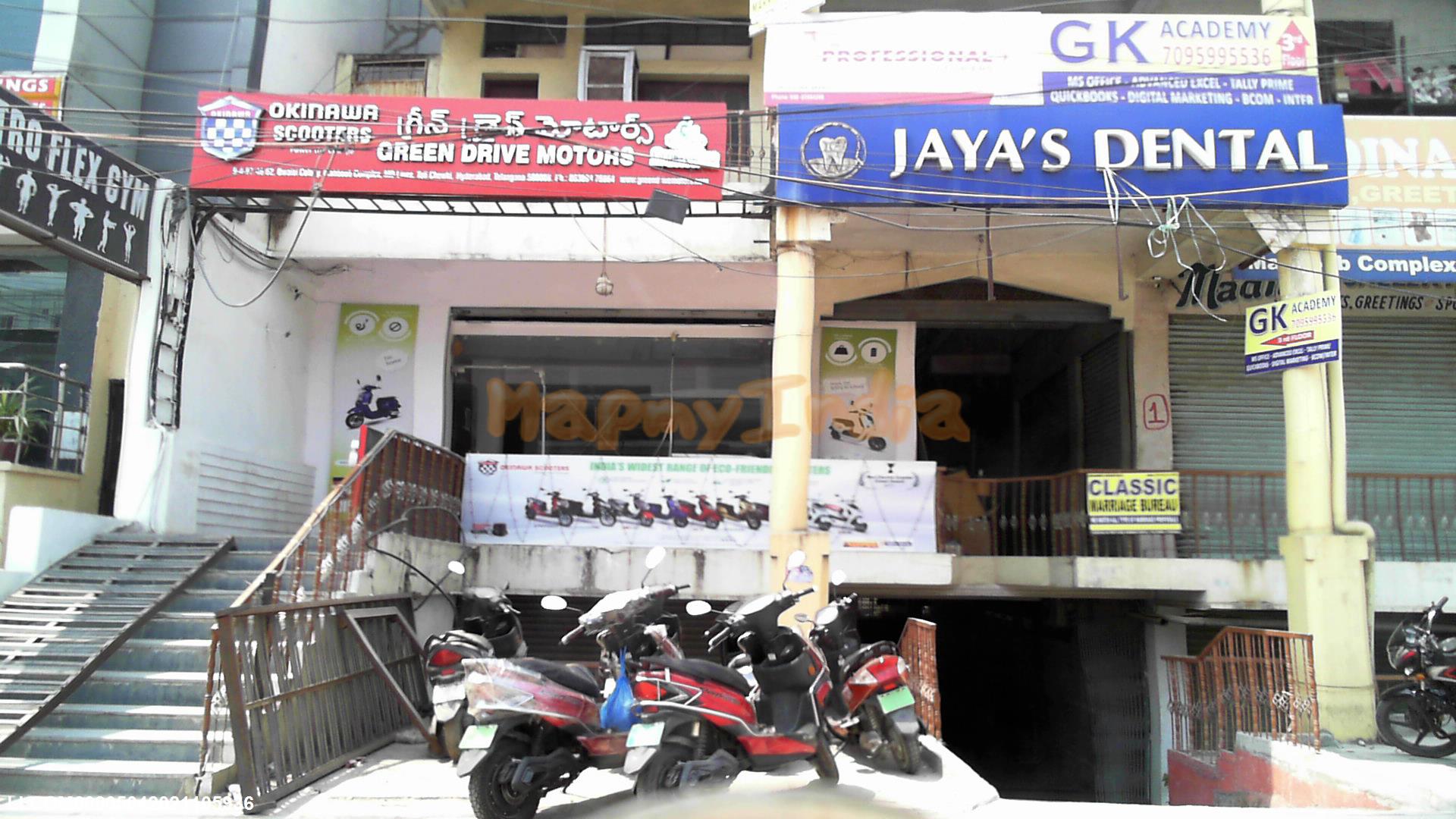

Supplement: Supplementary file 2 — Supplementary Material 2 [file 41598_2026_40742_MOESM2_ESM.zip › sample_data_yolov5/LCOM60805042021105936.jpg]

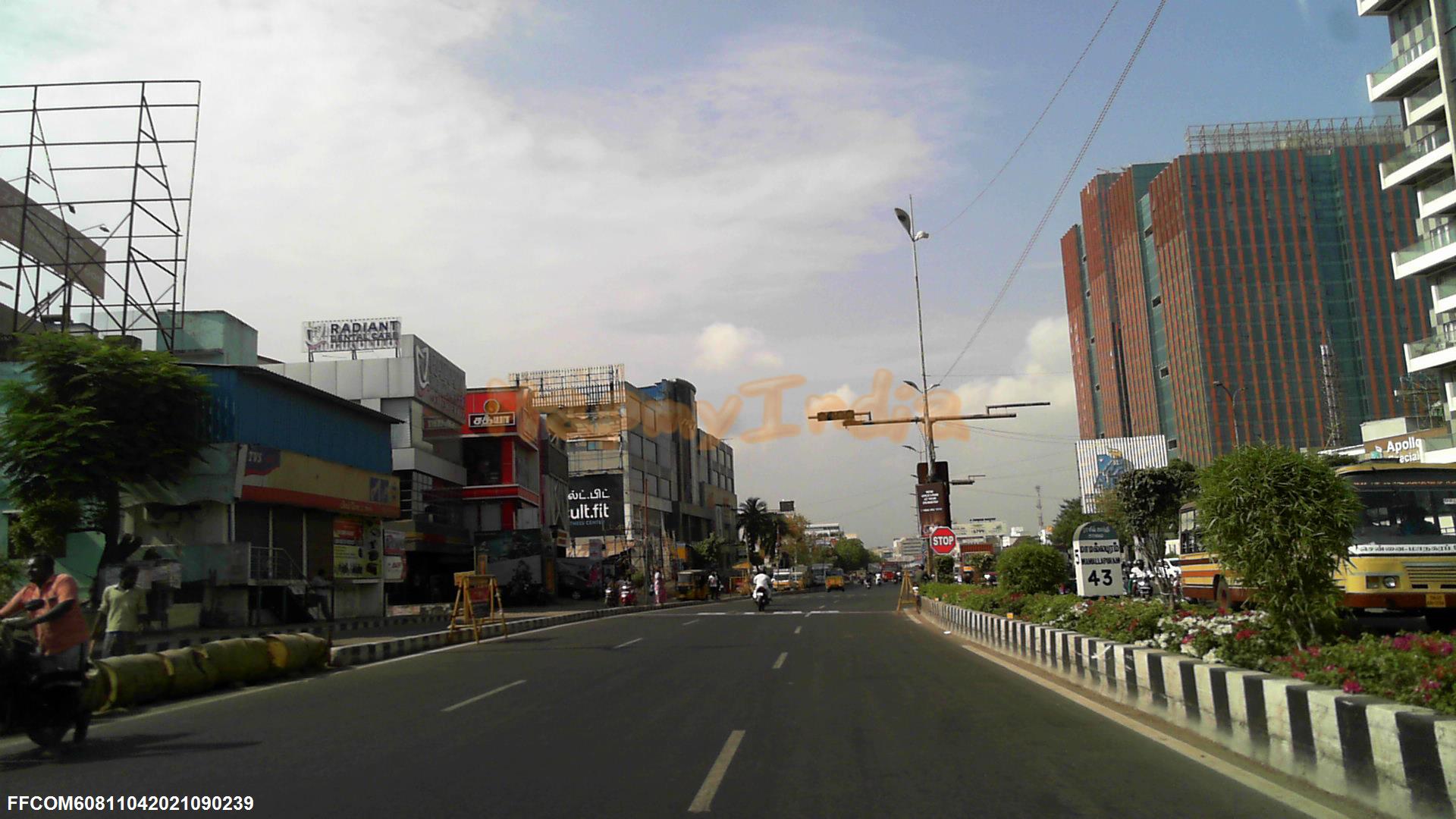

Supplement: Supplementary file 2 — Supplementary Material 2 [file 41598_2026_40742_MOESM2_ESM.zip › sample_data_yolov5/FCOM60811042021090239.jpg]

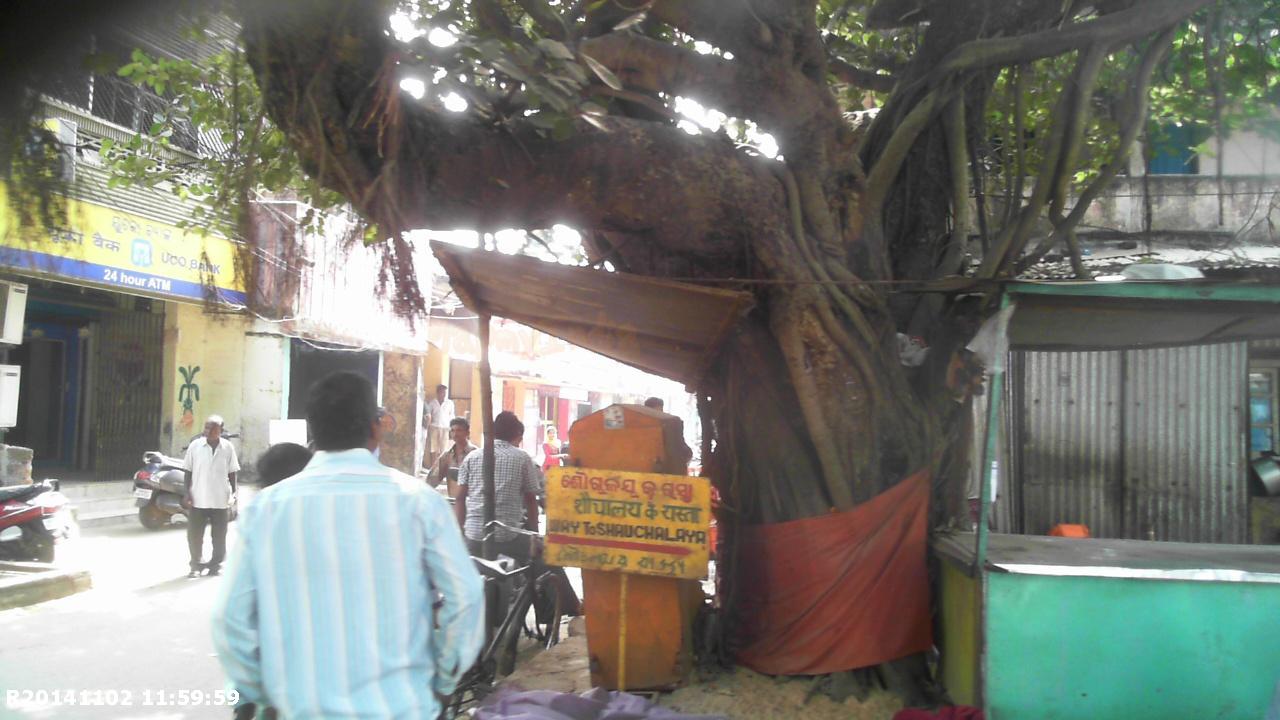

Supplement: Supplementary file 2 — Supplementary Material 2 [file 41598_2026_40742_MOESM2_ESM.zip › sample_data_yolov5/R_11-02_11.59.59.jpg]

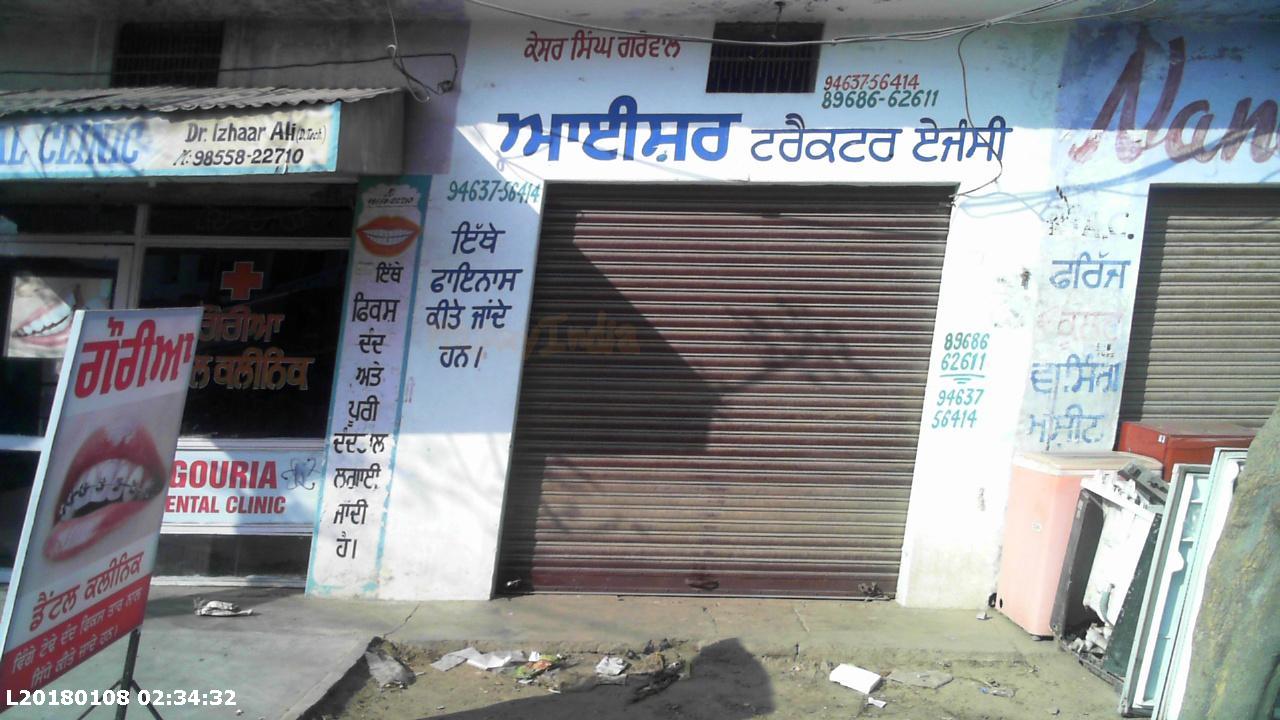

Supplement: Supplementary file 2 — Supplementary Material 2 [file 41598_2026_40742_MOESM2_ESM.zip › sample_data_yolov5/01-08 02.34.32.jpg]

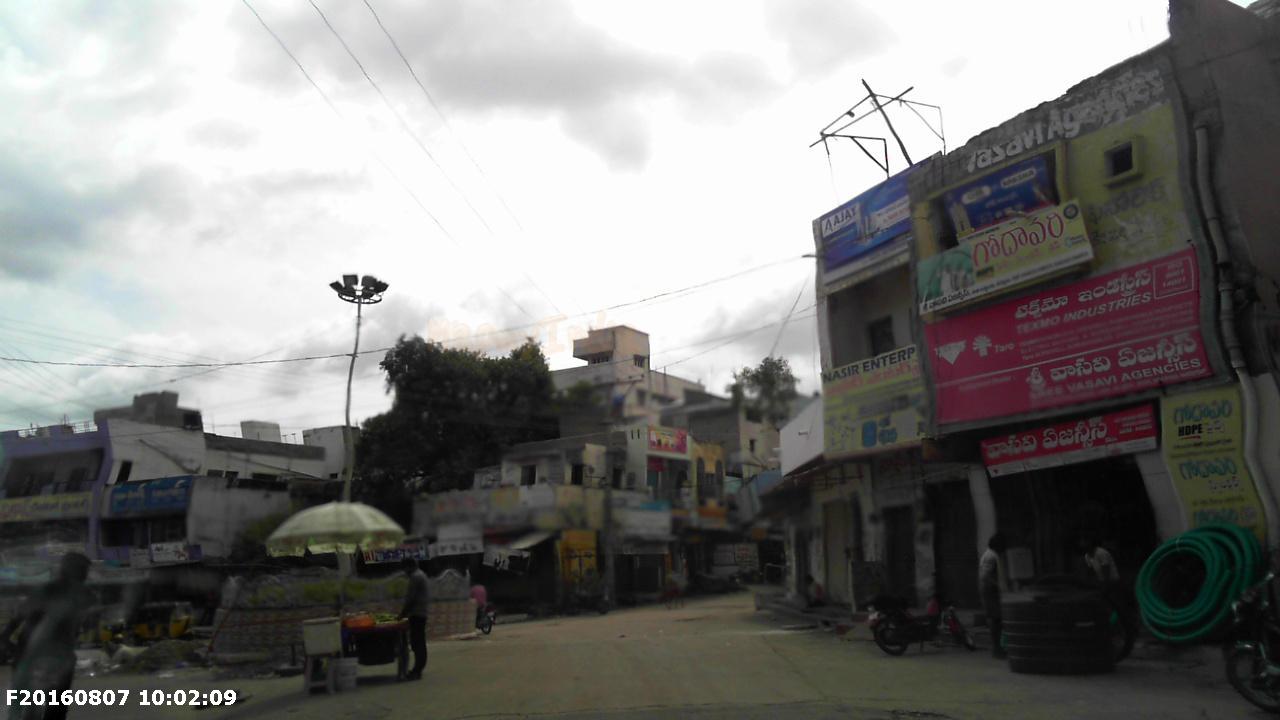

Supplement: Supplementary file 2 — Supplementary Material 2 [file 41598_2026_40742_MOESM2_ESM.zip › sample_data_yolov5/08-07 10.02.09.jpg]

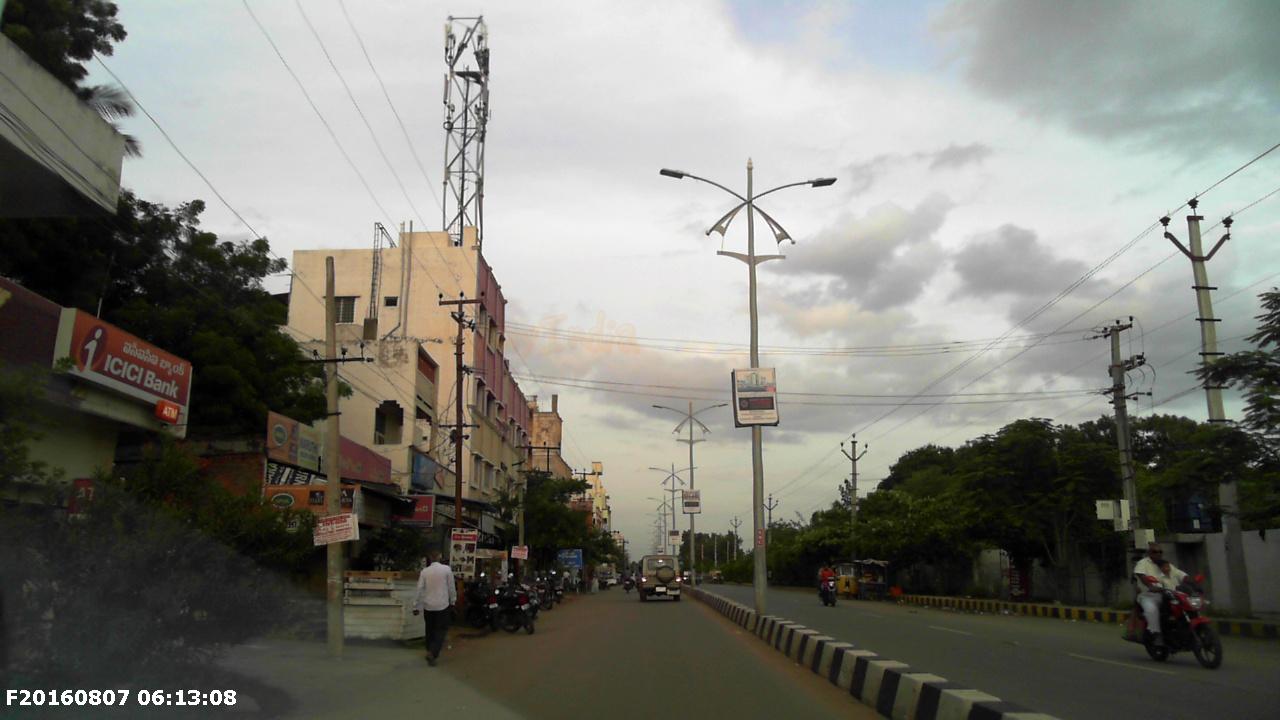

Supplement: Supplementary file 2 — Supplementary Material 2 [file 41598_2026_40742_MOESM2_ESM.zip › sample_data_yolov5/08-07 06.13.08.jpg]

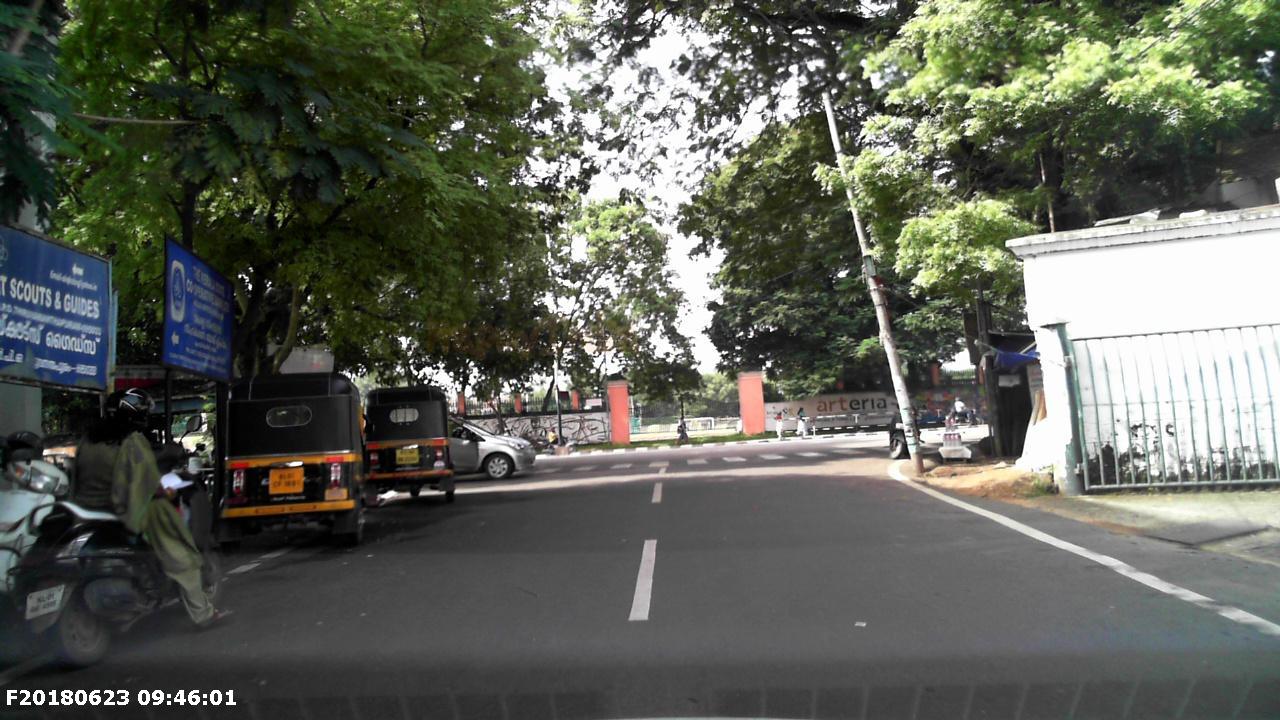

Supplement: Supplementary file 2 — Supplementary Material 2 [file 41598_2026_40742_MOESM2_ESM.zip › sample_data_yolov5/F_06-23_09.46.01.jpg]

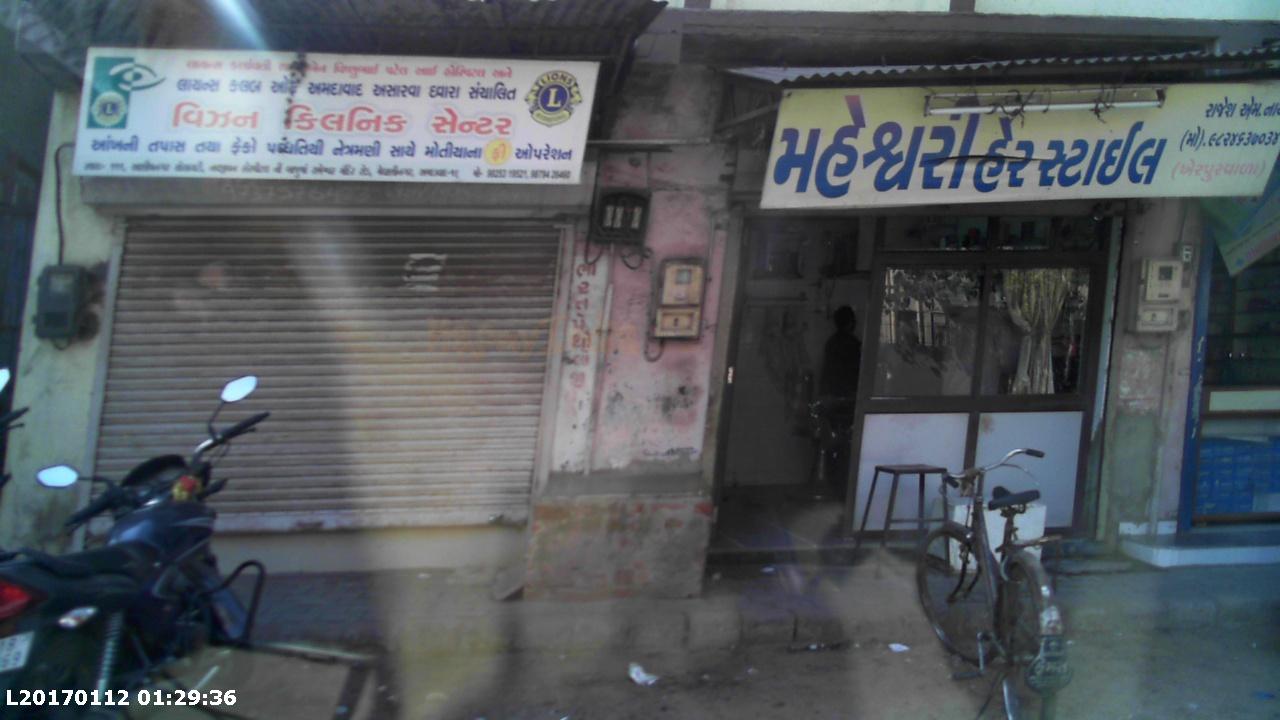

Supplement: Supplementary file 2 — Supplementary Material 2 [file 41598_2026_40742_MOESM2_ESM.zip › sample_data_yolov5/01-12 01.29.36.jpg]

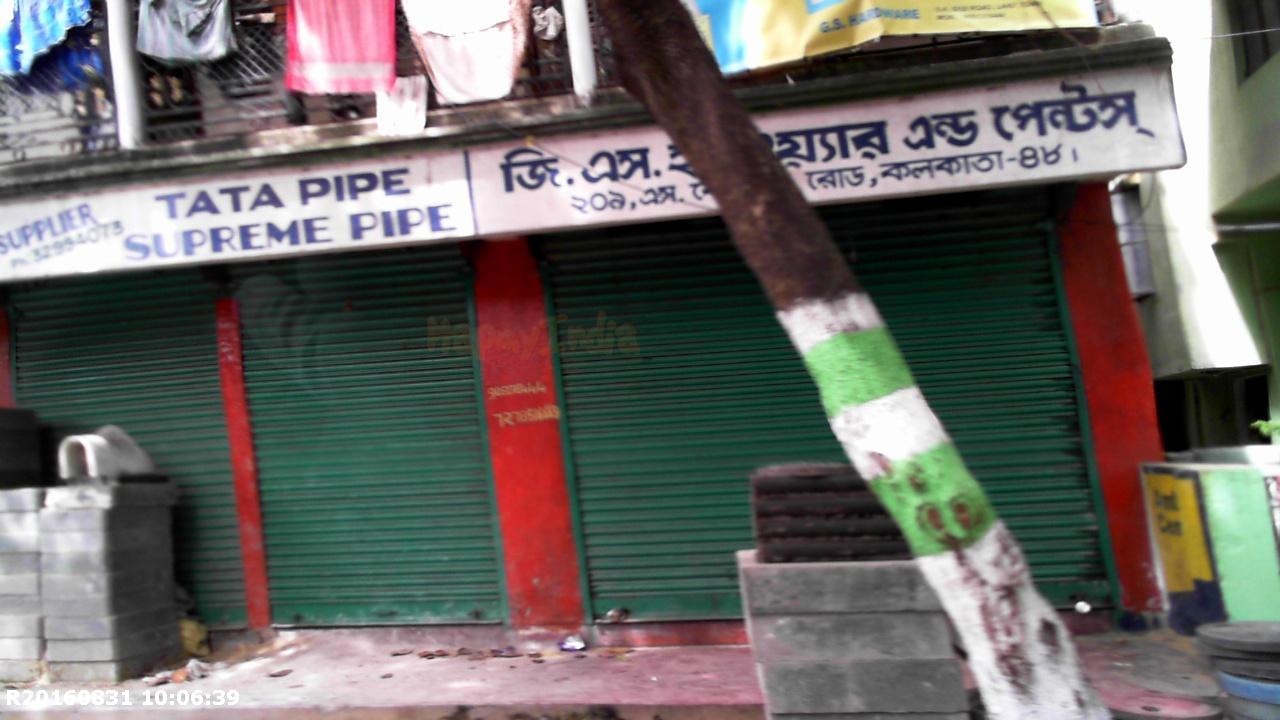

Supplement: Supplementary file 2 — Supplementary Material 2 [file 41598_2026_40742_MOESM2_ESM.zip › sample_data_yolov5/R_08-31_10.06.39.jpg]

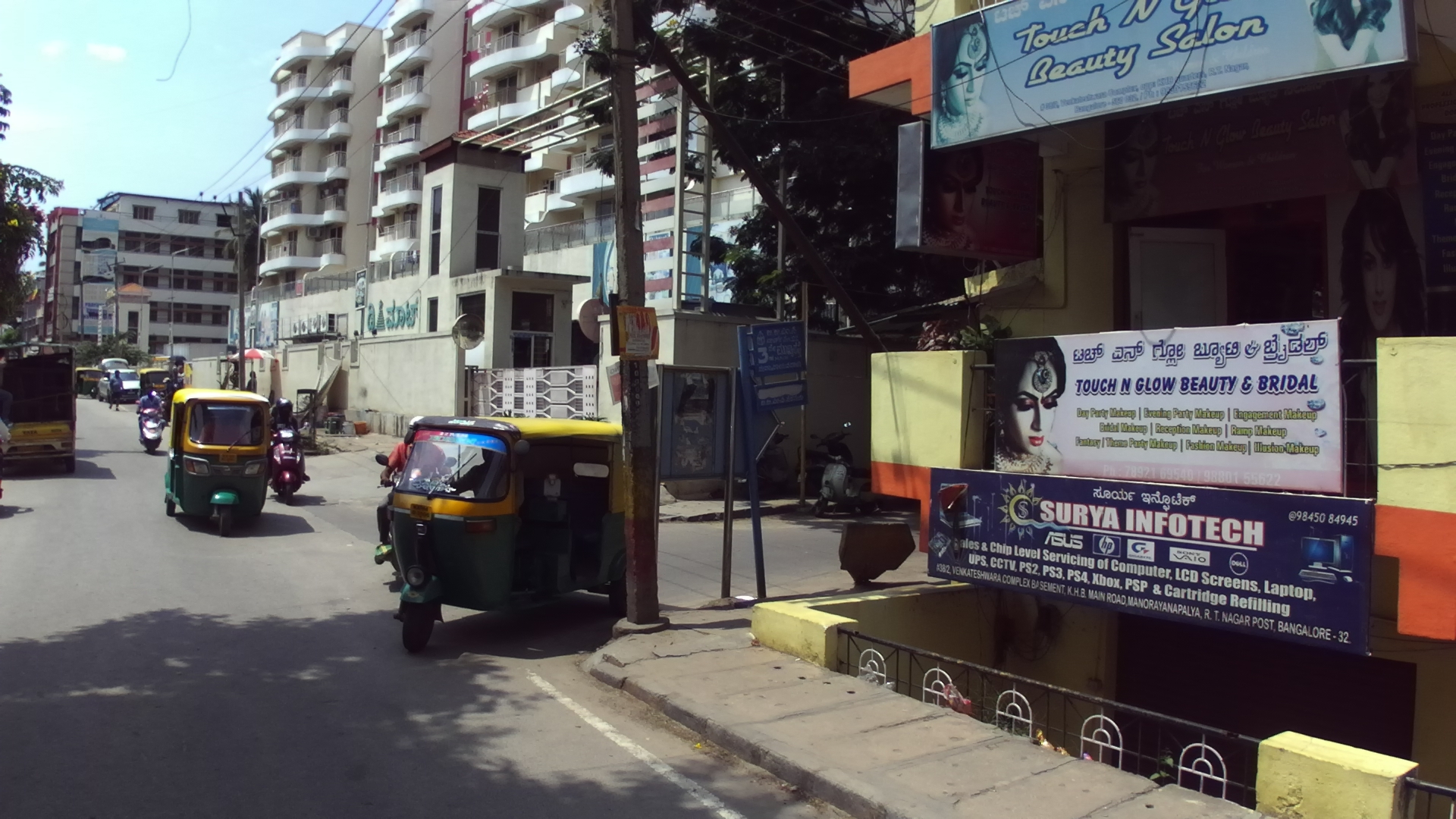

Supplement: Supplementary file 2 — Supplementary Material 2 [file 41598_2026_40742_MOESM2_ESM.zip › sample_data_yolov5/T1_051020_112403_24376_zed_l_118.jpg]

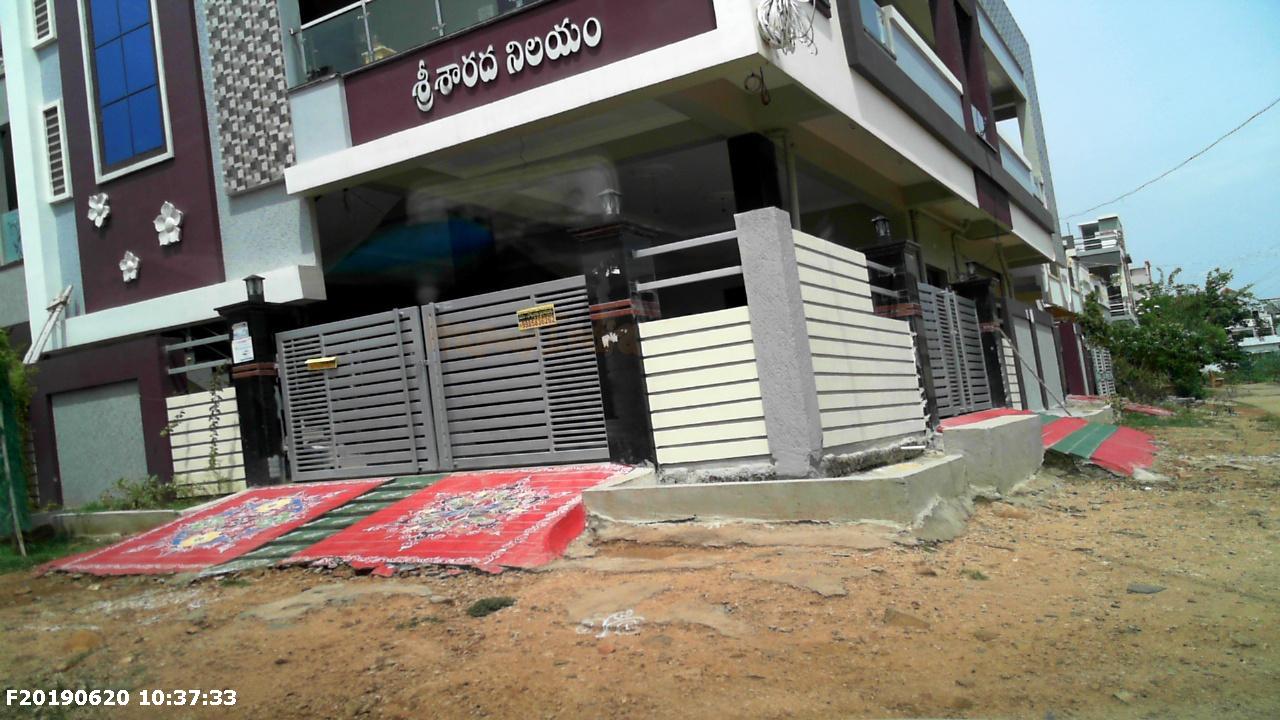

Supplement: Supplementary file 2 — Supplementary Material 2 [file 41598_2026_40742_MOESM2_ESM.zip › sample_data_yolov5/06-20 10.37.33.jpg]

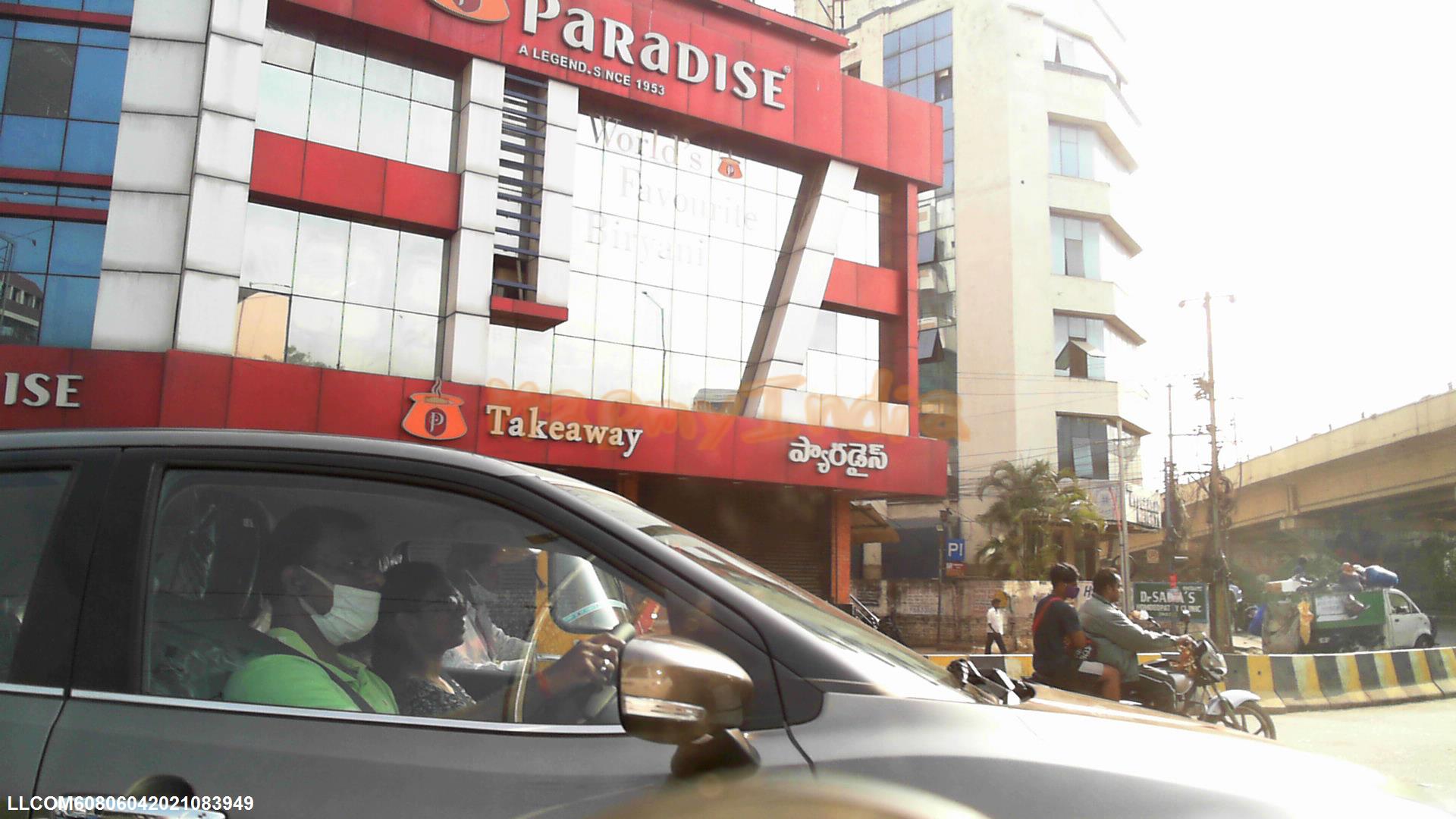

Supplement: Supplementary file 2 — Supplementary Material 2 [file 41598_2026_40742_MOESM2_ESM.zip › sample_data_yolov5/LCOM60806042021083949.jpg]

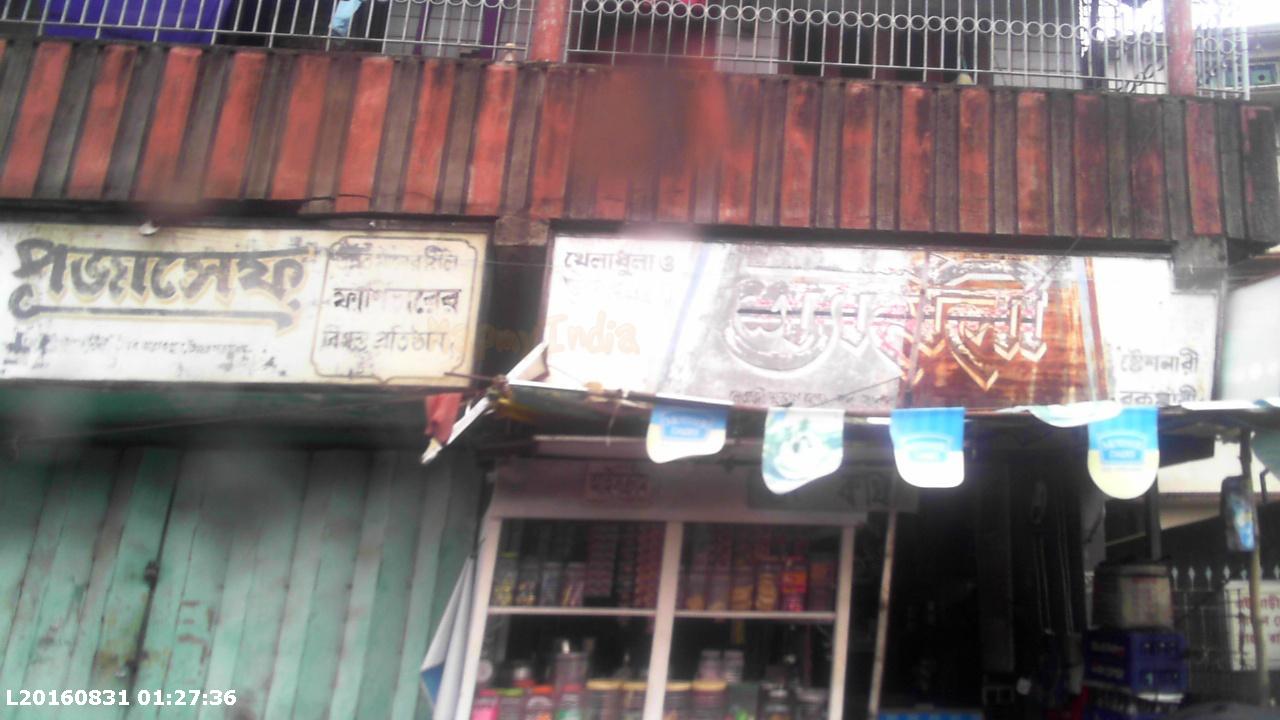

Supplement: Supplementary file 2 — Supplementary Material 2 [file 41598_2026_40742_MOESM2_ESM.zip › sample_data_yolov5/L_08-31_01.27.36.jpg]

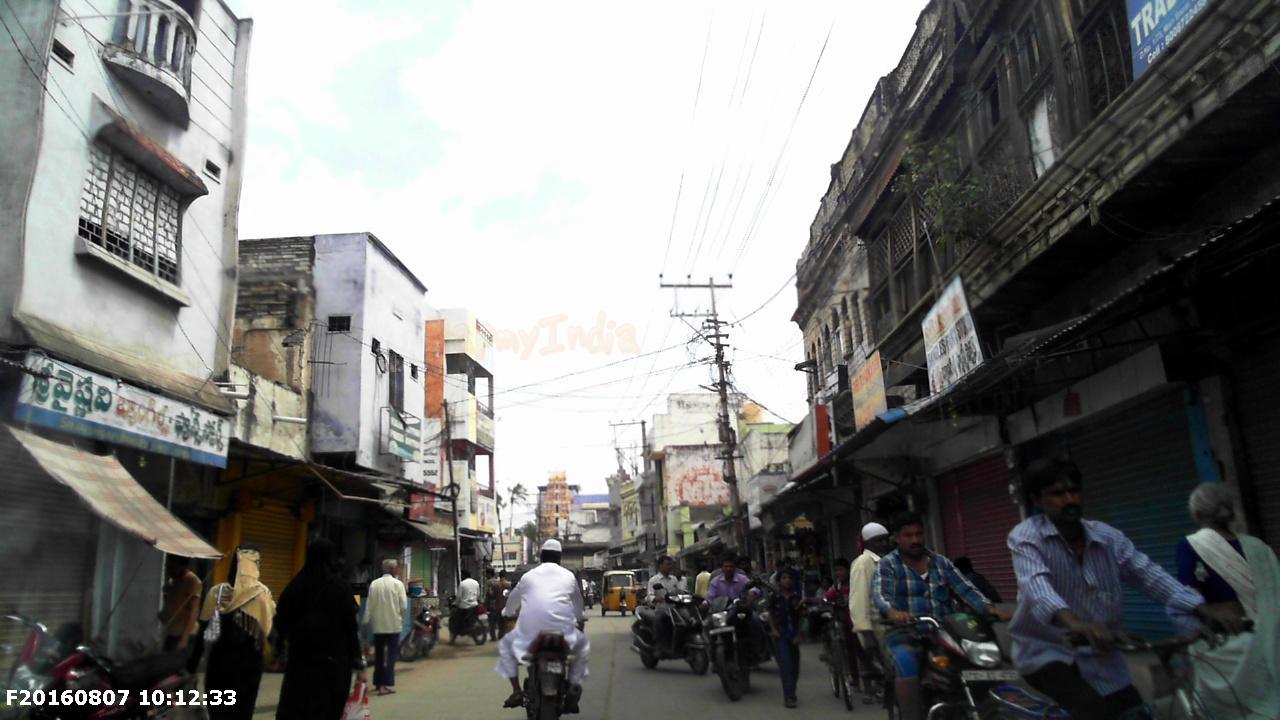

Supplement: Supplementary file 2 — Supplementary Material 2 [file 41598_2026_40742_MOESM2_ESM.zip › sample_data_yolov5/08-07 10.12.33.jpg]

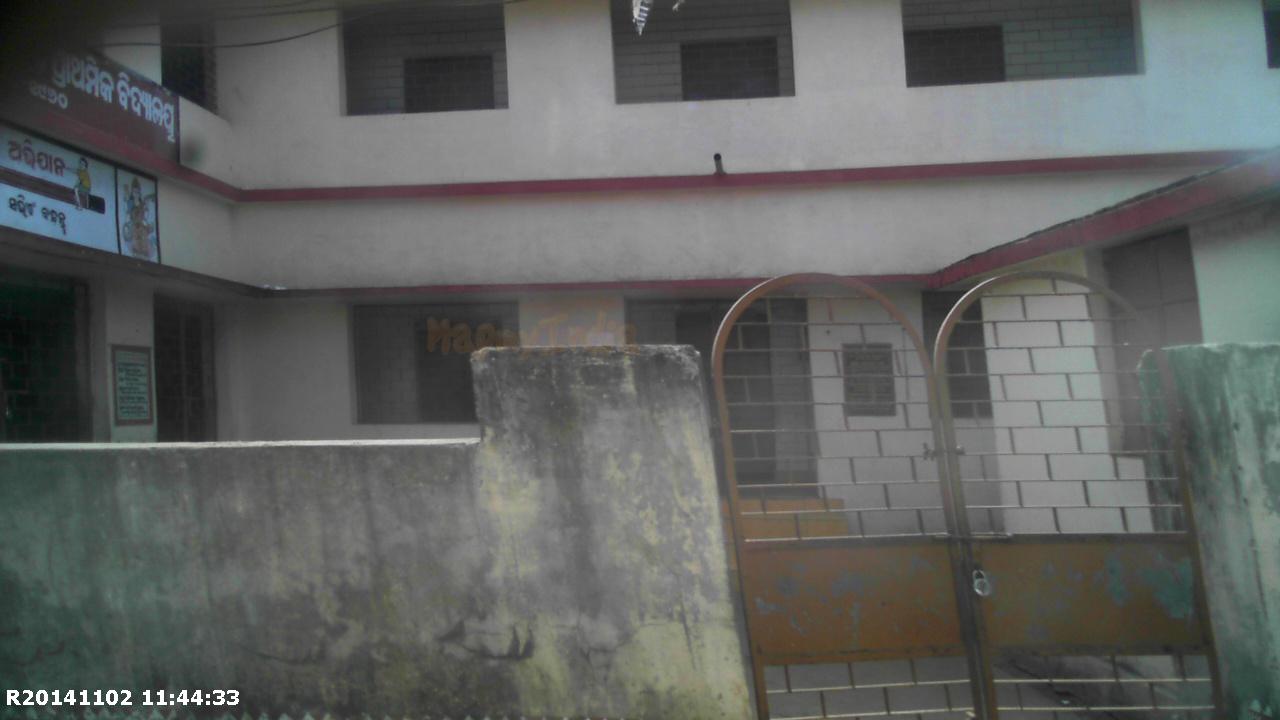

Supplement: Supplementary file 2 — Supplementary Material 2 [file 41598_2026_40742_MOESM2_ESM.zip › sample_data_yolov5/R_11-02_11.44.33.jpg]

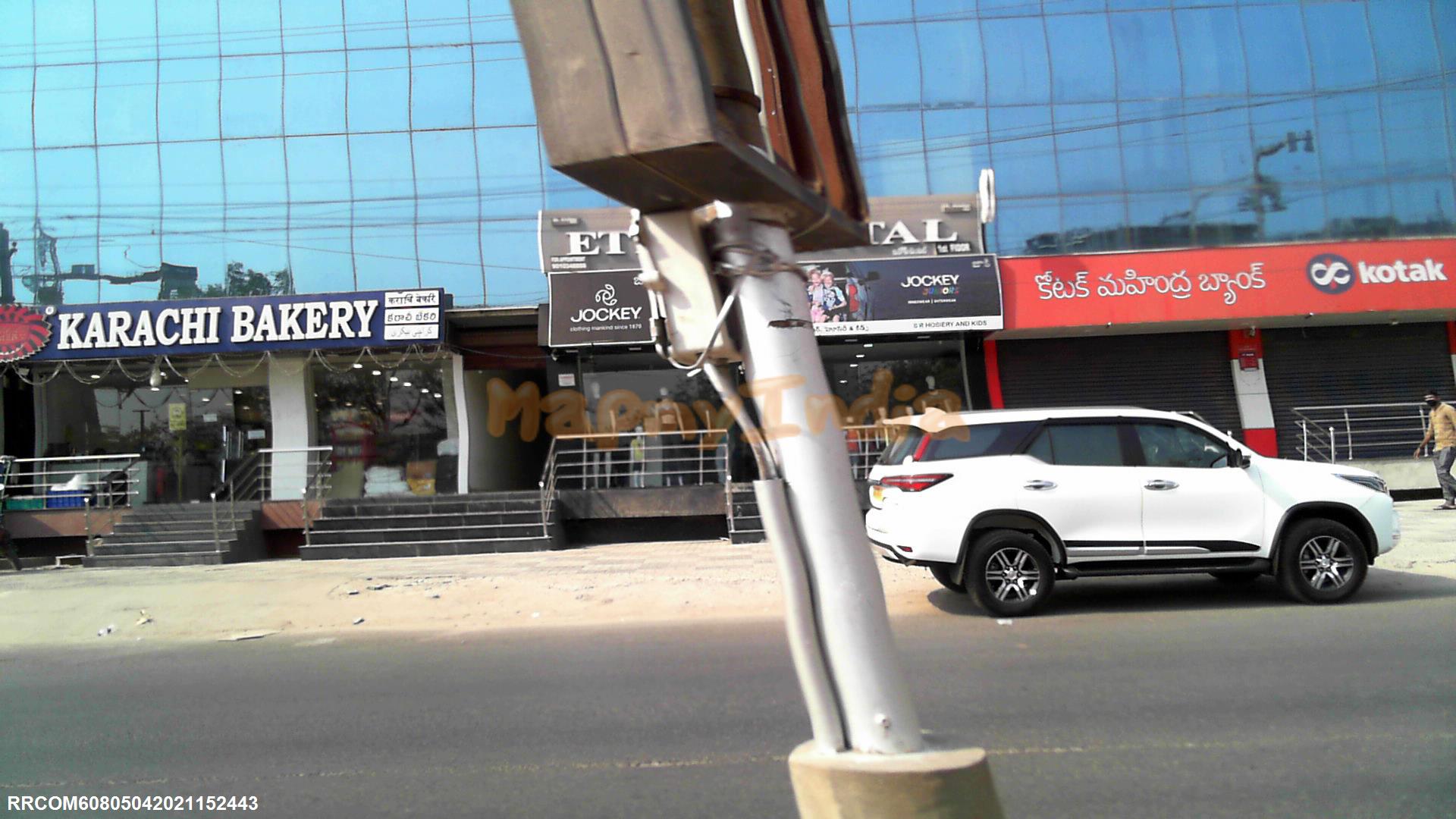

Supplement: Supplementary file 2 — Supplementary Material 2 [file 41598_2026_40742_MOESM2_ESM.zip › sample_data_yolov5/RCOM60805042021152443.jpg]

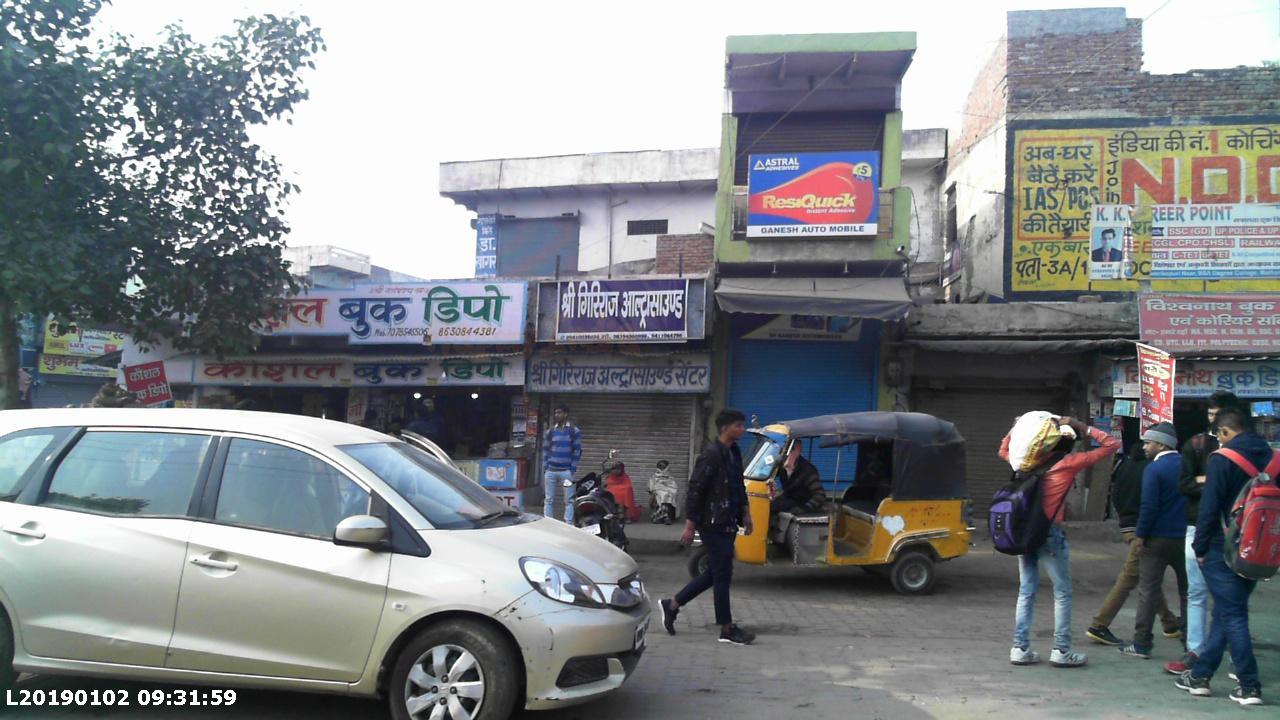

Supplement: Supplementary file 2 — Supplementary Material 2 [file 41598_2026_40742_MOESM2_ESM.zip › sample_data_yolov5/L_01-02_09.31.59.jpg]

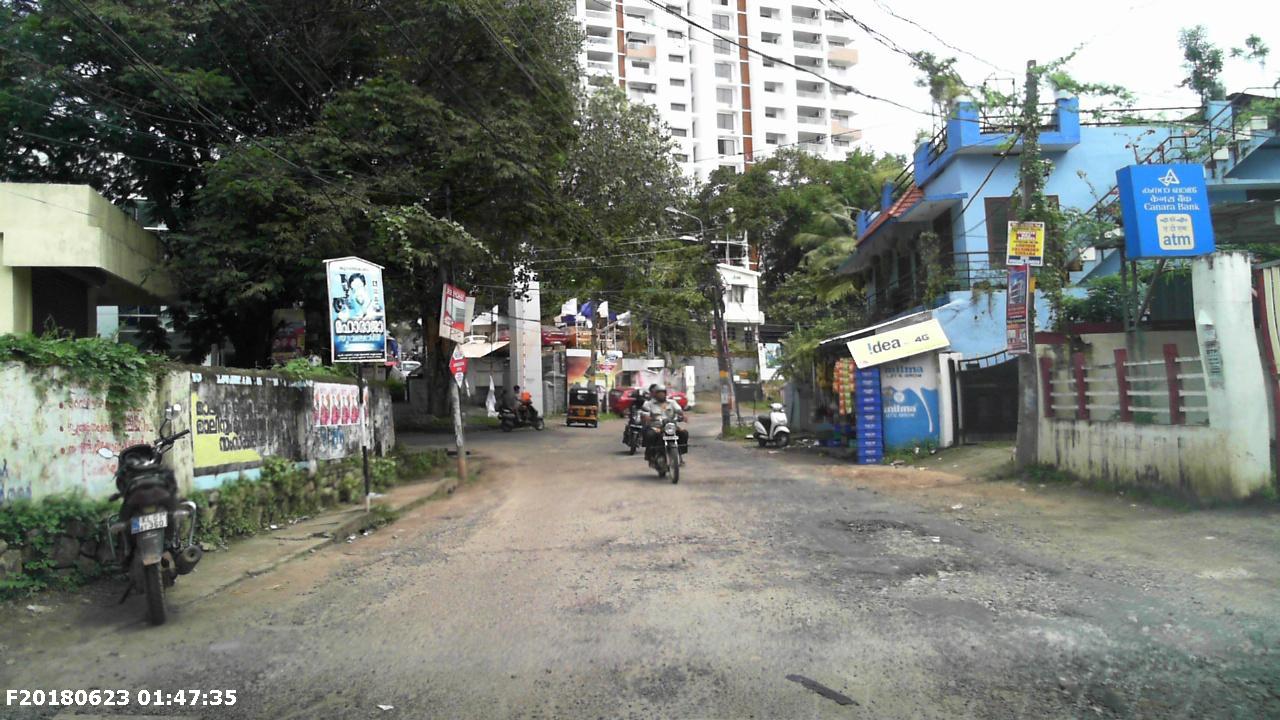

Supplement: Supplementary file 2 — Supplementary Material 2 [file 41598_2026_40742_MOESM2_ESM.zip › sample_data_yolov5/F_06-23_01.47.35.jpg]

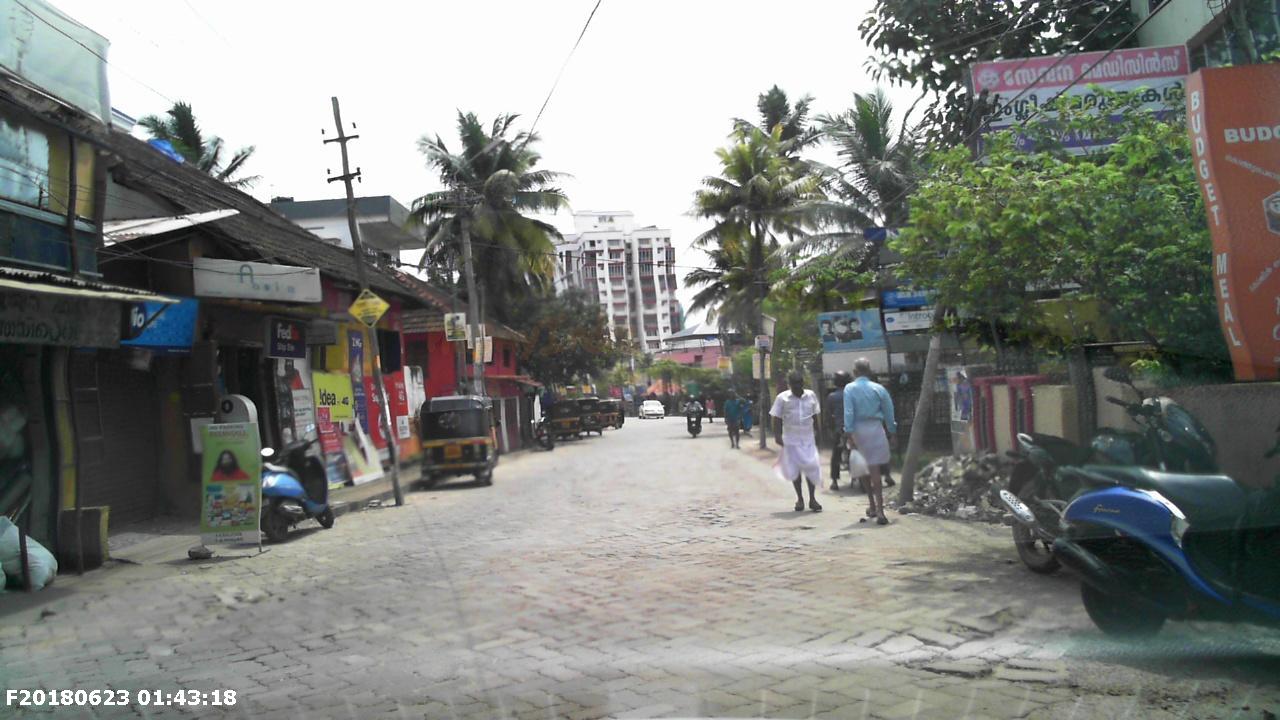

Supplement: Supplementary file 2 — Supplementary Material 2 [file 41598_2026_40742_MOESM2_ESM.zip › sample_data_yolov5/F_06-23_01.43.18.jpg]

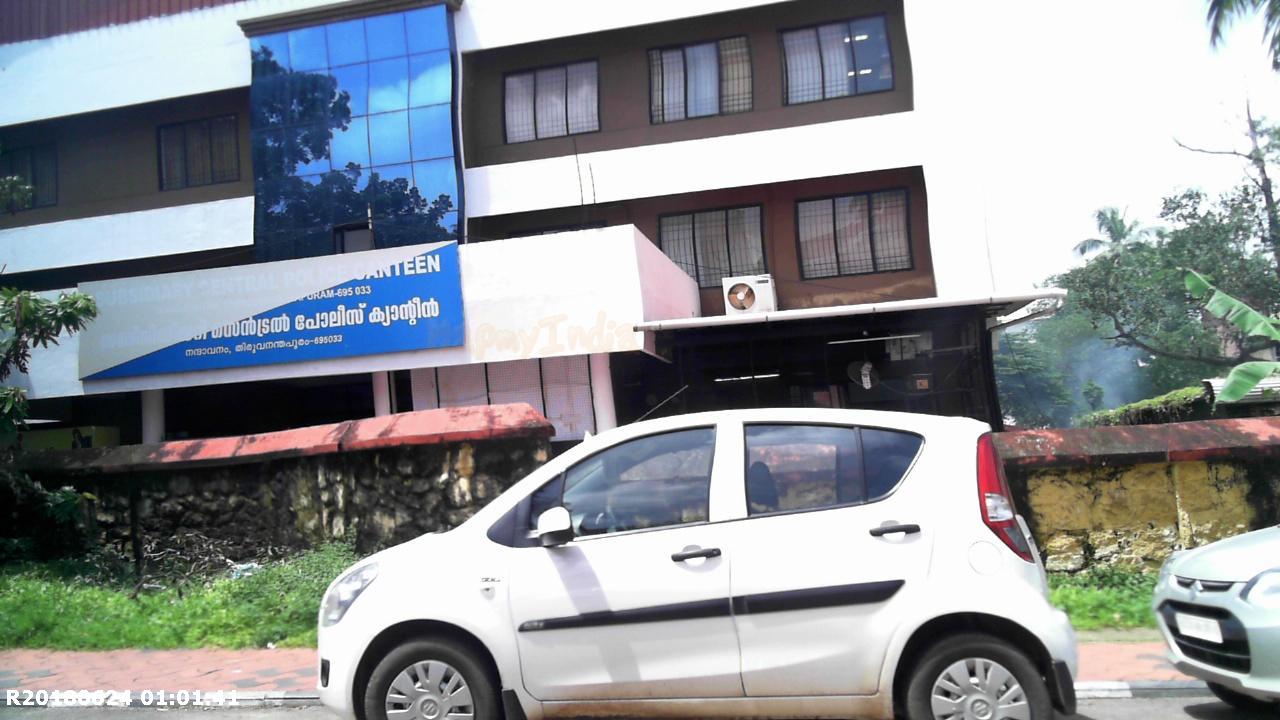

Supplement: Supplementary file 2 — Supplementary Material 2 [file 41598_2026_40742_MOESM2_ESM.zip › sample_data_yolov5/R_06-24_01.01.41.jpg]

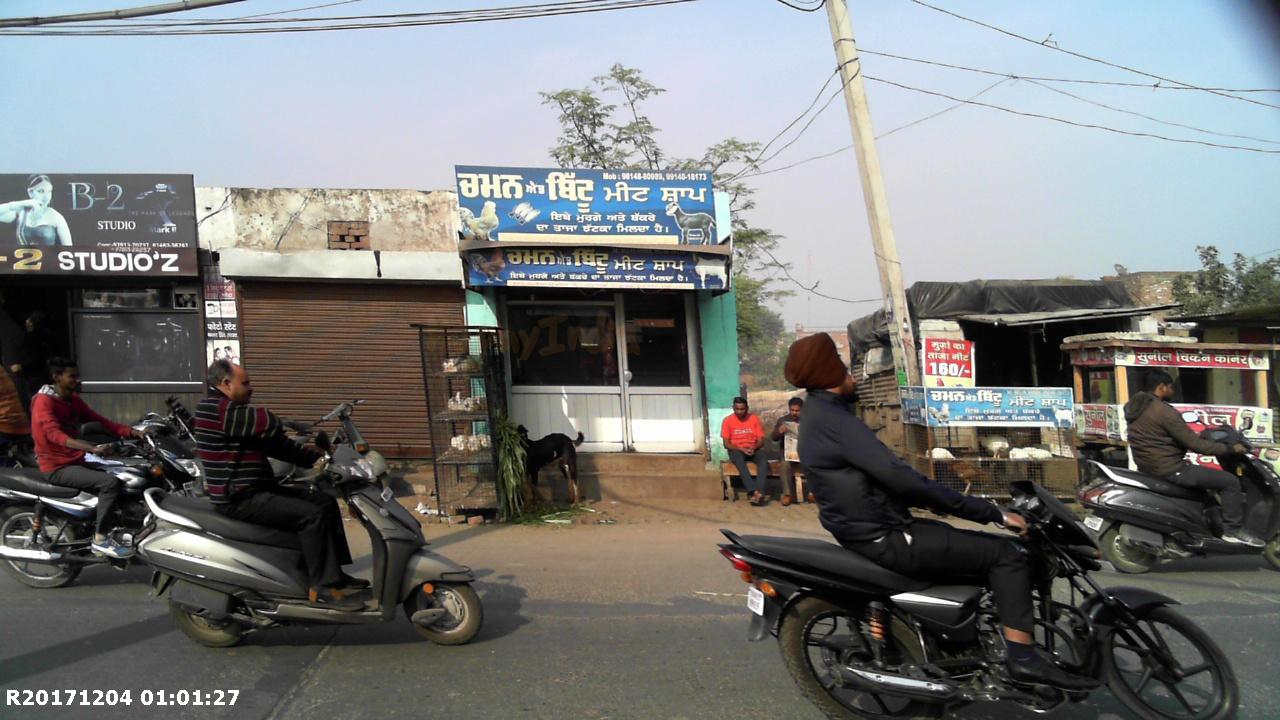

Supplement: Supplementary file 2 — Supplementary Material 2 [file 41598_2026_40742_MOESM2_ESM.zip › sample_data_yolov5/12-04 01.01.27.jpg]

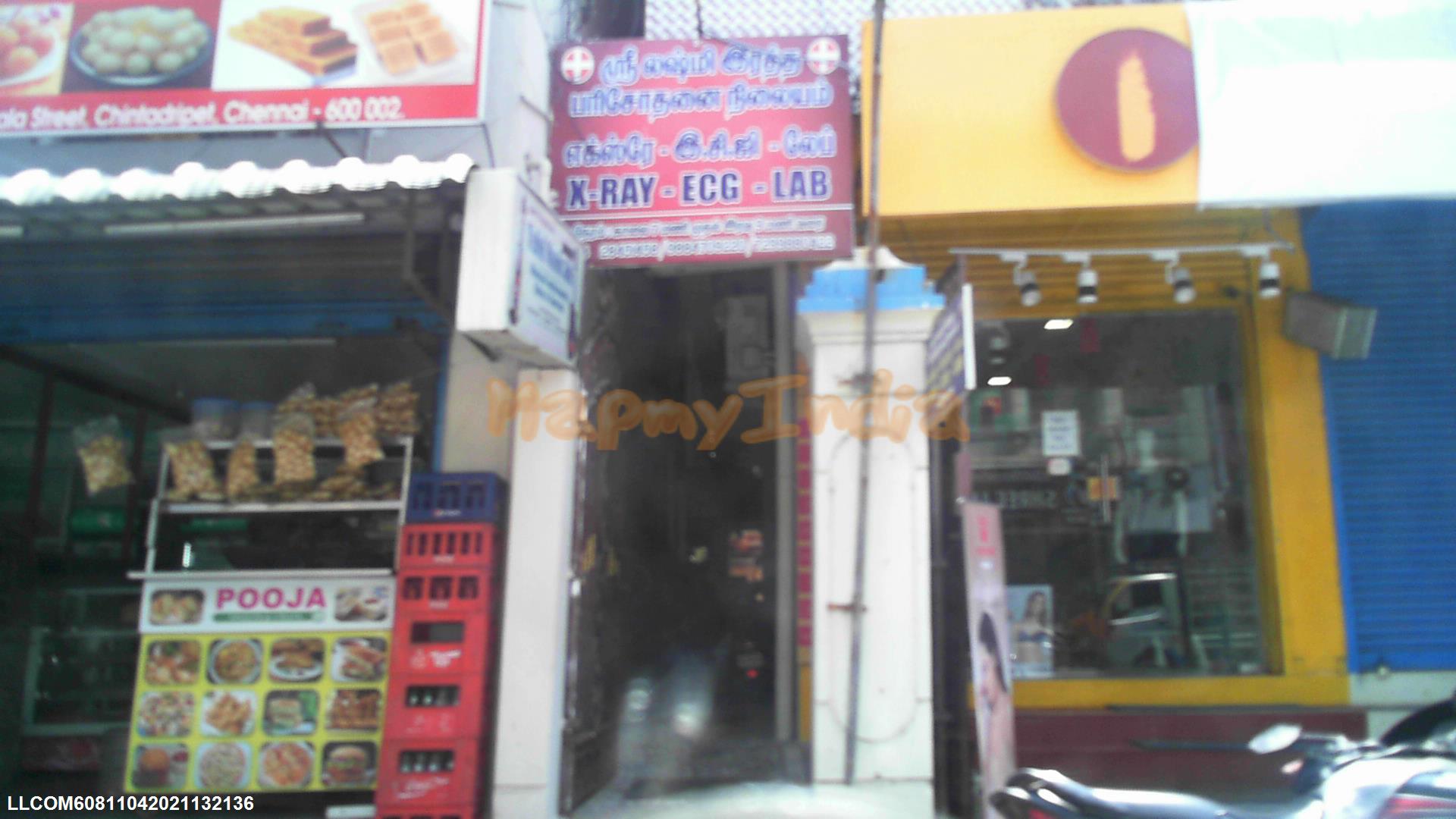

Supplement: Supplementary file 2 — Supplementary Material 2 [file 41598_2026_40742_MOESM2_ESM.zip › sample_data_yolov5/LCOM60811042021132136.jpg]

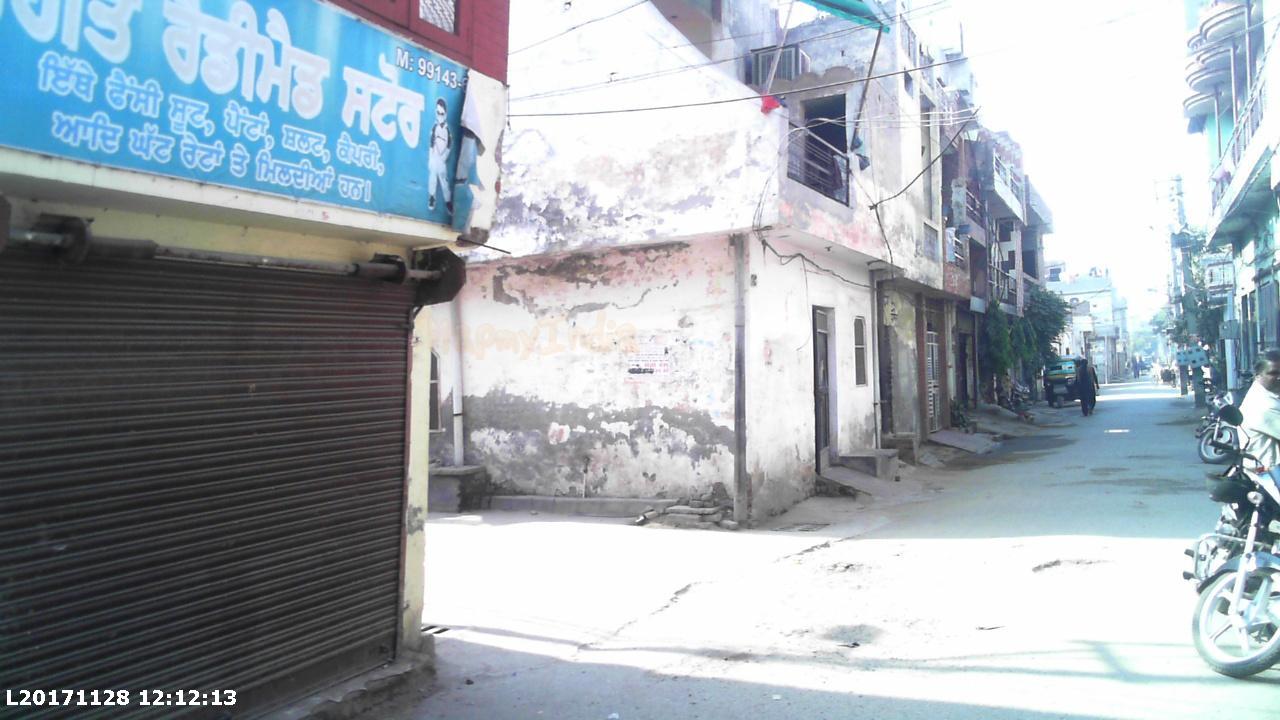

Supplement: Supplementary file 2 — Supplementary Material 2 [file 41598_2026_40742_MOESM2_ESM.zip › sample_data_yolov5/11-28 12.12.13.jpg]

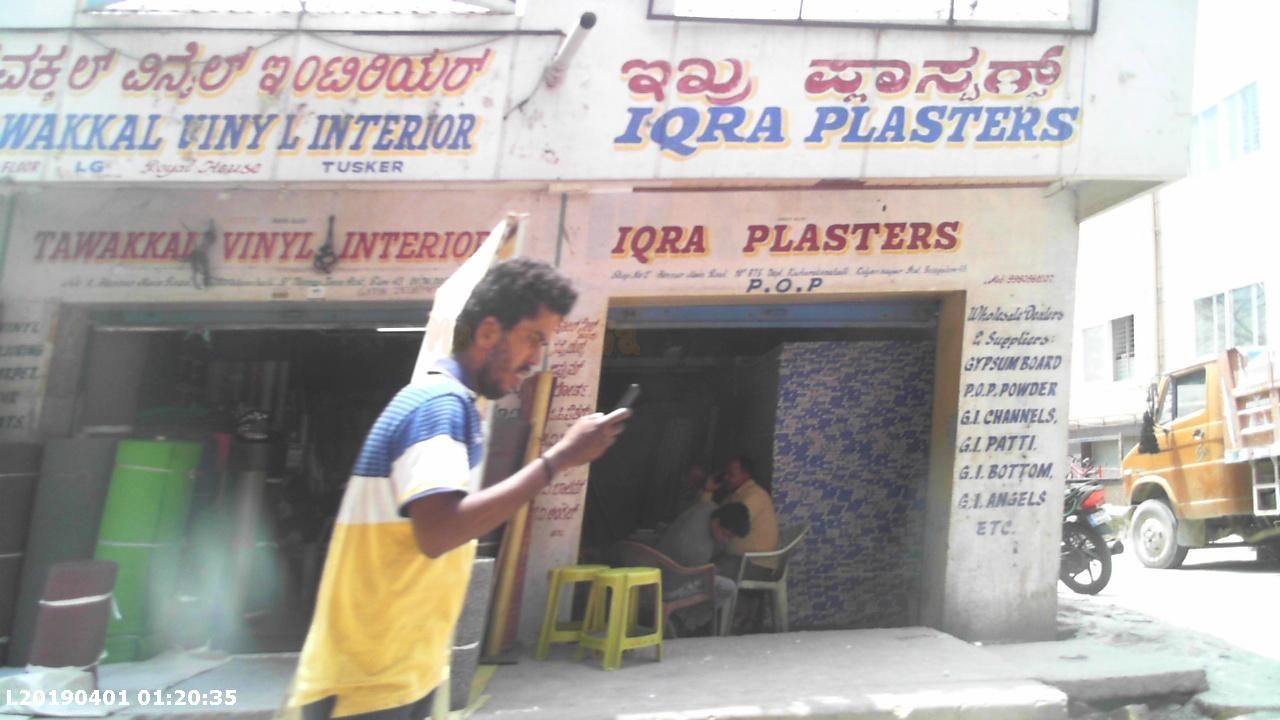

Supplement: Supplementary file 2 — Supplementary Material 2 [file 41598_2026_40742_MOESM2_ESM.zip › sample_data_yolov5/04-01_01.20.35.jpg]

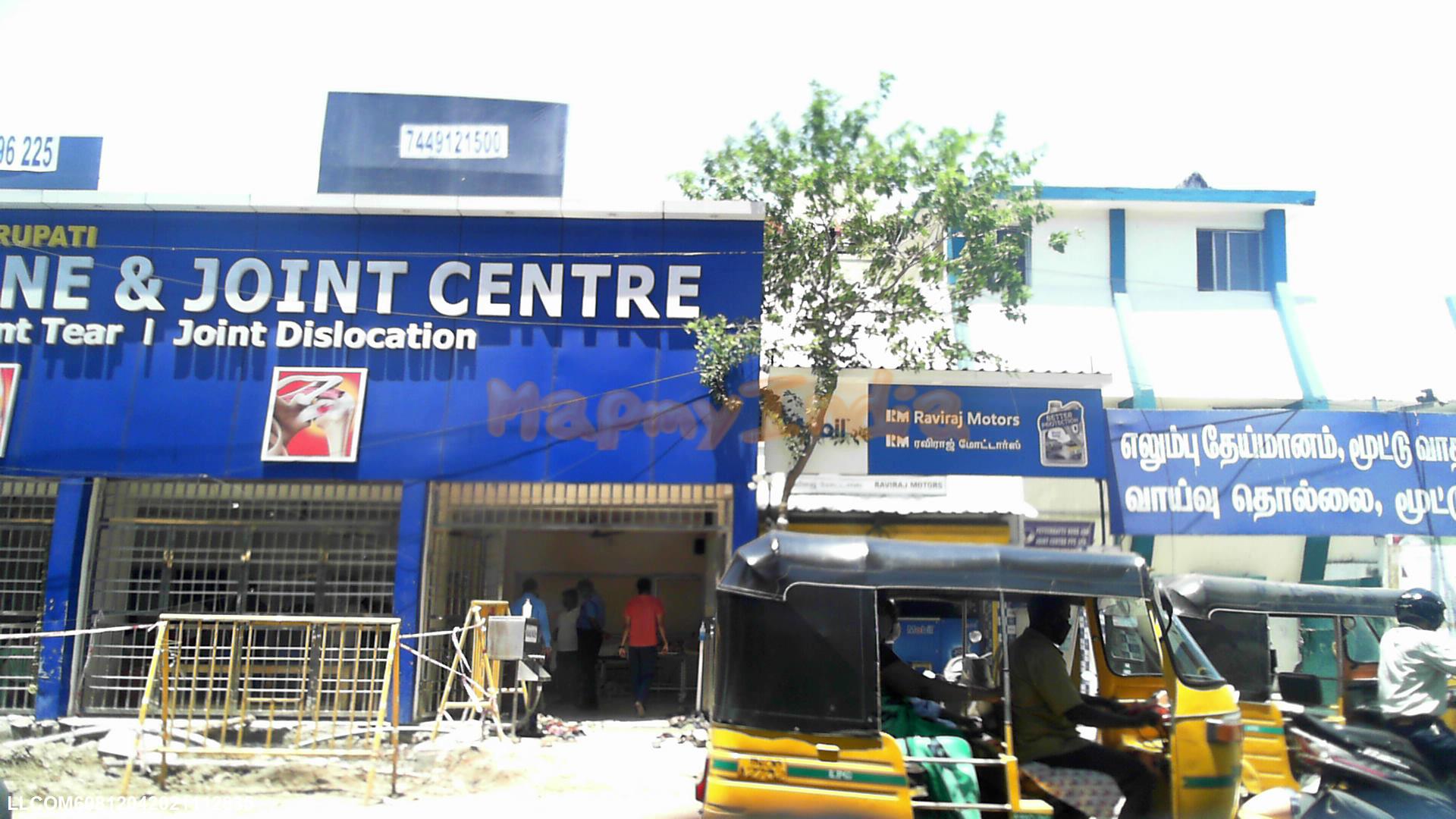

Supplement: Supplementary file 2 — Supplementary Material 2 [file 41598_2026_40742_MOESM2_ESM.zip › sample_data_yolov5/LCOM60812042021112835.jpg]

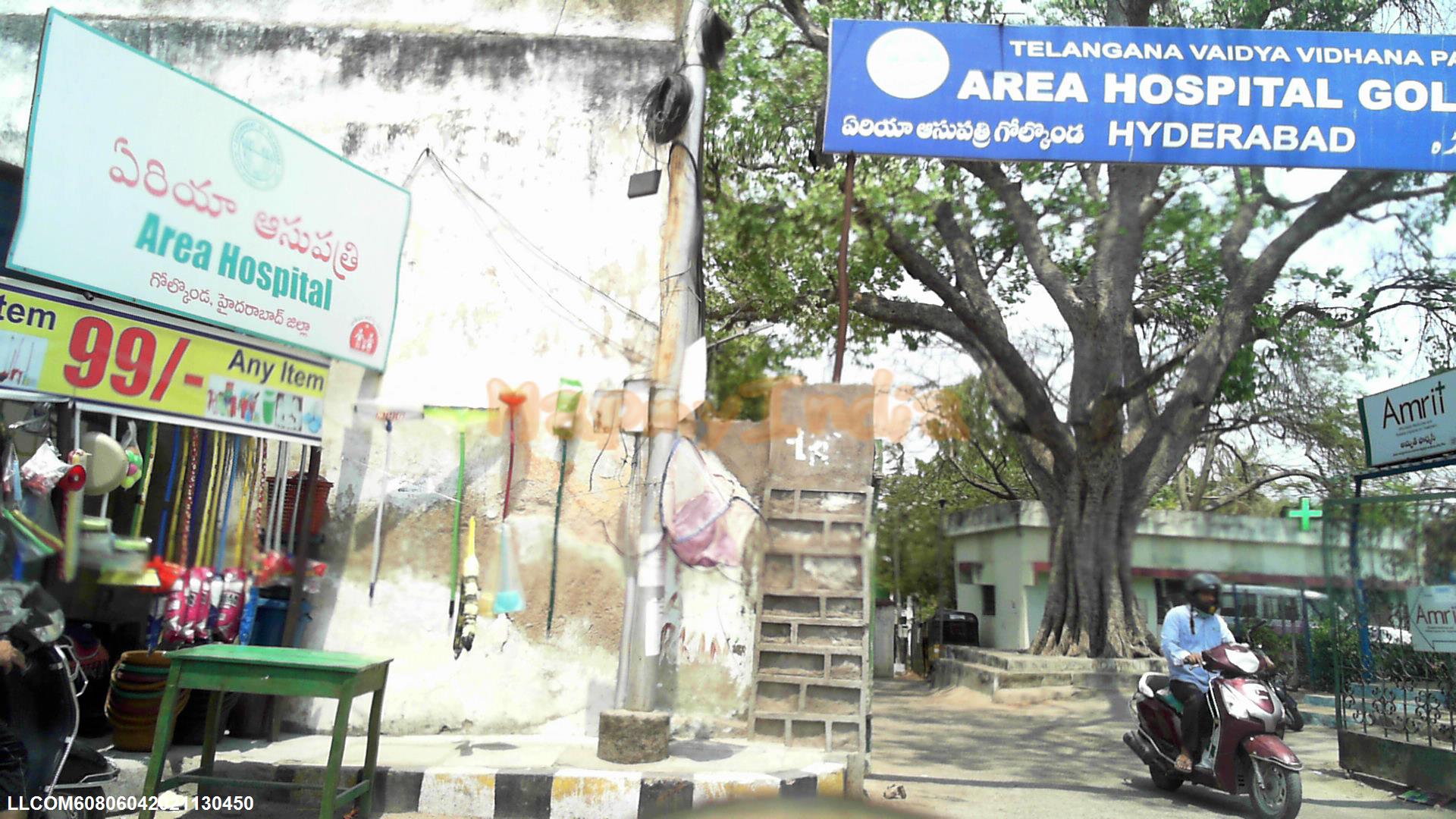

Supplement: Supplementary file 2 — Supplementary Material 2 [file 41598_2026_40742_MOESM2_ESM.zip › sample_data_yolov5/LCOM60806042021130450.jpg]

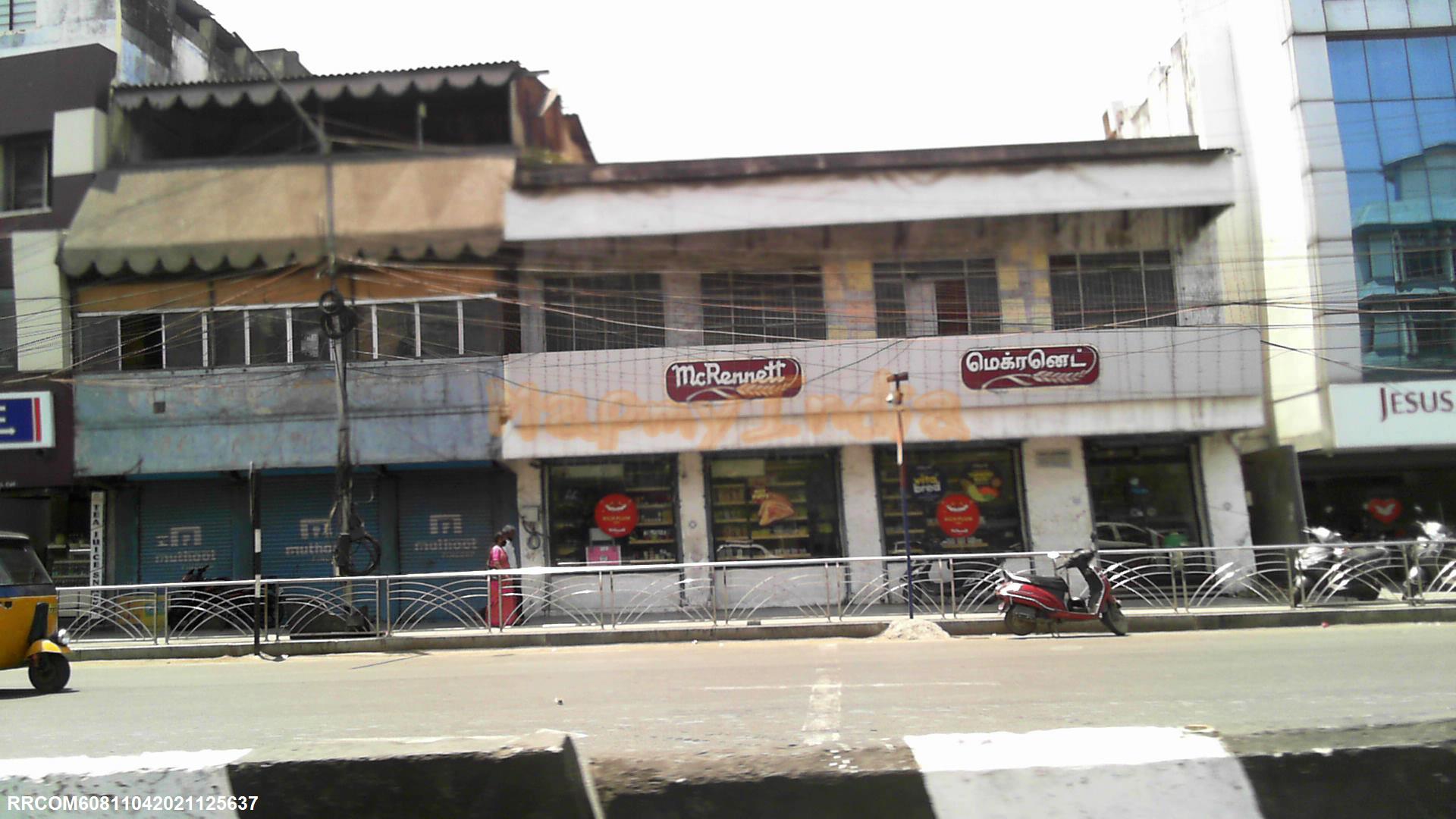

Supplement: Supplementary file 2 — Supplementary Material 2 [file 41598_2026_40742_MOESM2_ESM.zip › sample_data_yolov5/RCOM60811042021125637.jpg]

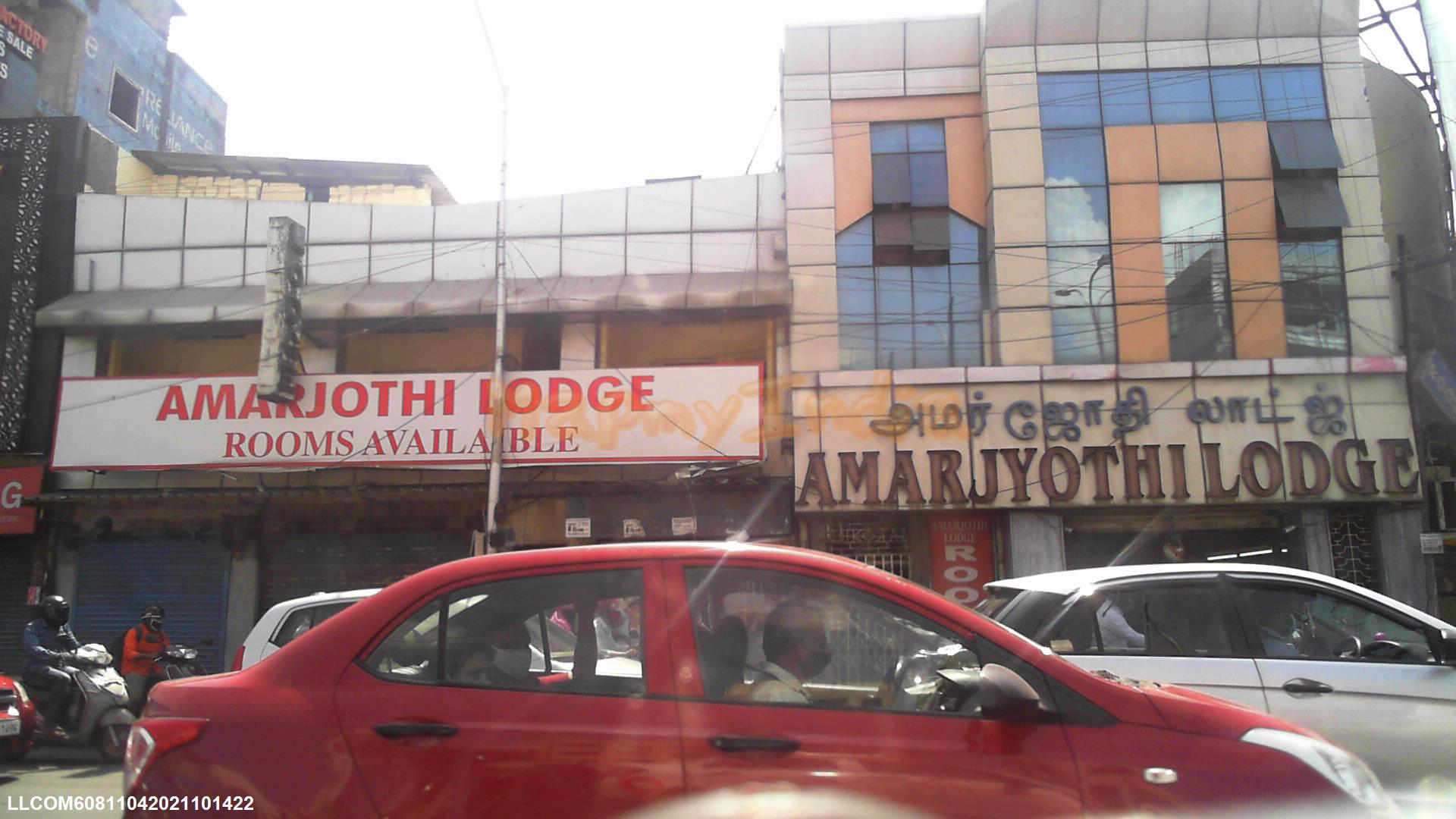

Supplement: Supplementary file 2 — Supplementary Material 2 [file 41598_2026_40742_MOESM2_ESM.zip › sample_data_yolov5/LCOM60811042021101422.jpg]

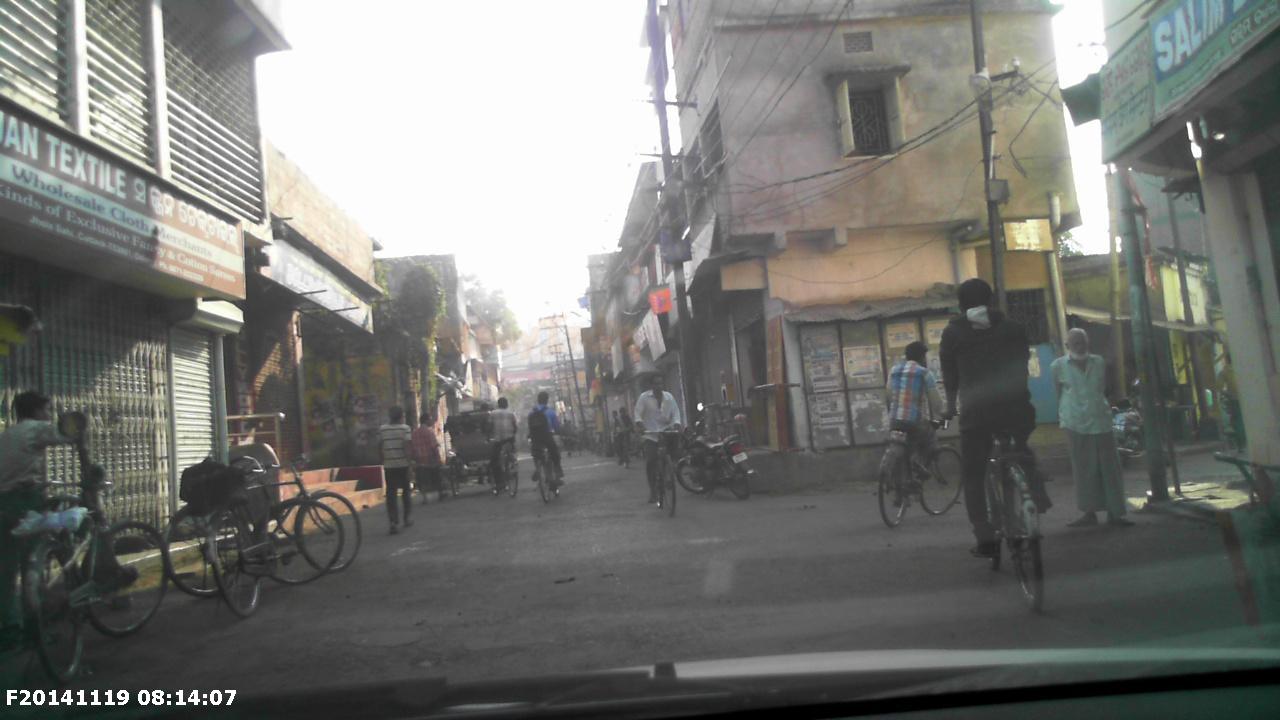

Supplement: Supplementary file 2 — Supplementary Material 2 [file 41598_2026_40742_MOESM2_ESM.zip › sample_data_yolov5/F_11-19_08.14.07.jpg]

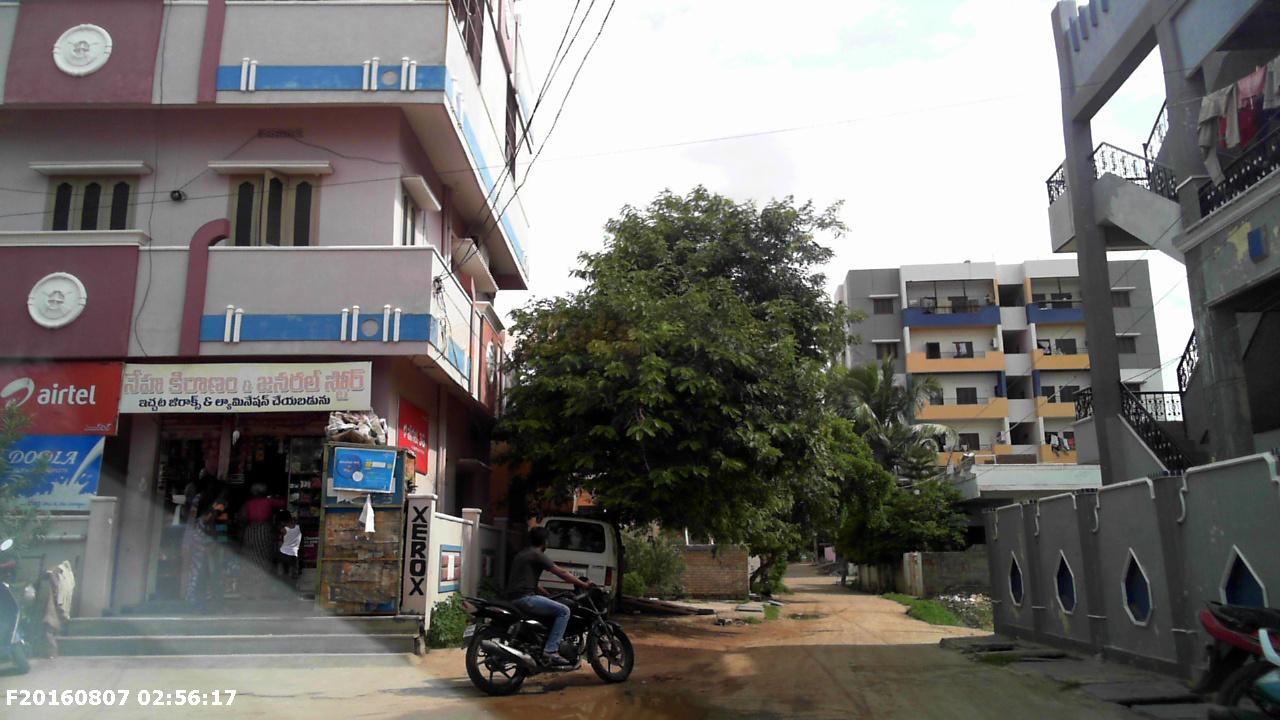

Supplement: Supplementary file 2 — Supplementary Material 2 [file 41598_2026_40742_MOESM2_ESM.zip › sample_data_yolov5/08-07 02.56.17.jpg]

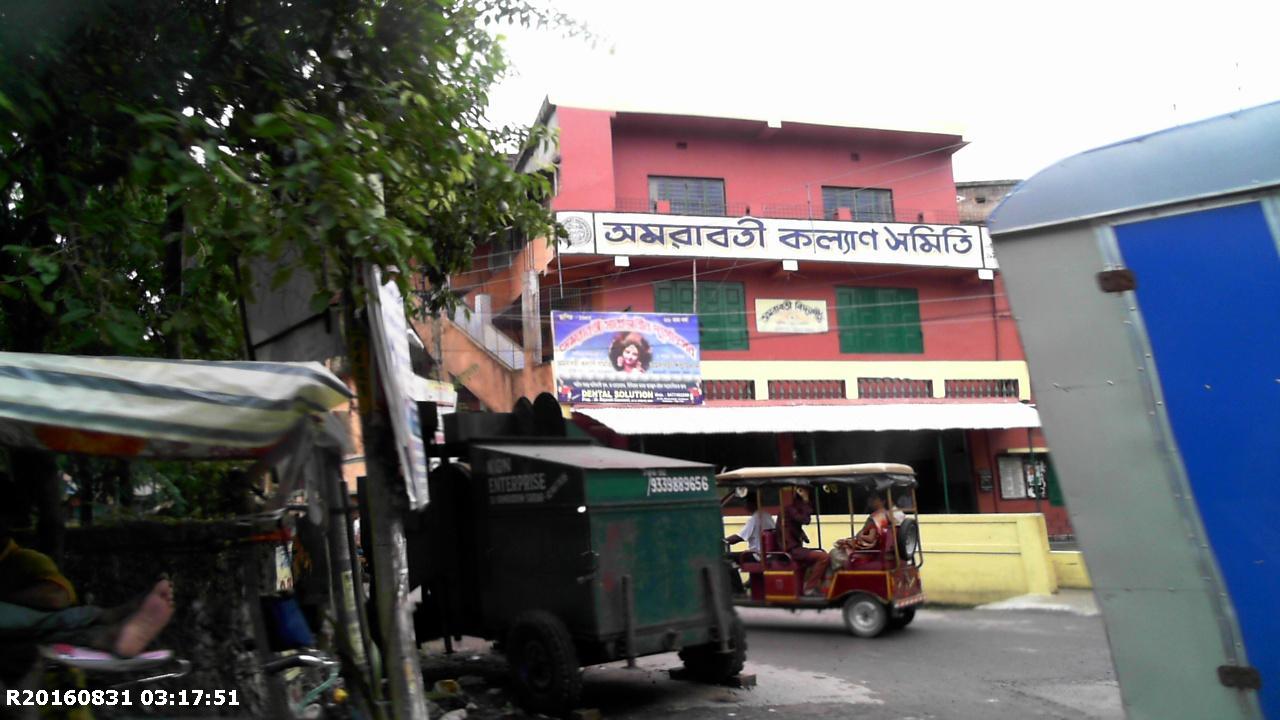

Supplement: Supplementary file 2 — Supplementary Material 2 [file 41598_2026_40742_MOESM2_ESM.zip › sample_data_yolov5/R_08-31_03.17.51.jpg]

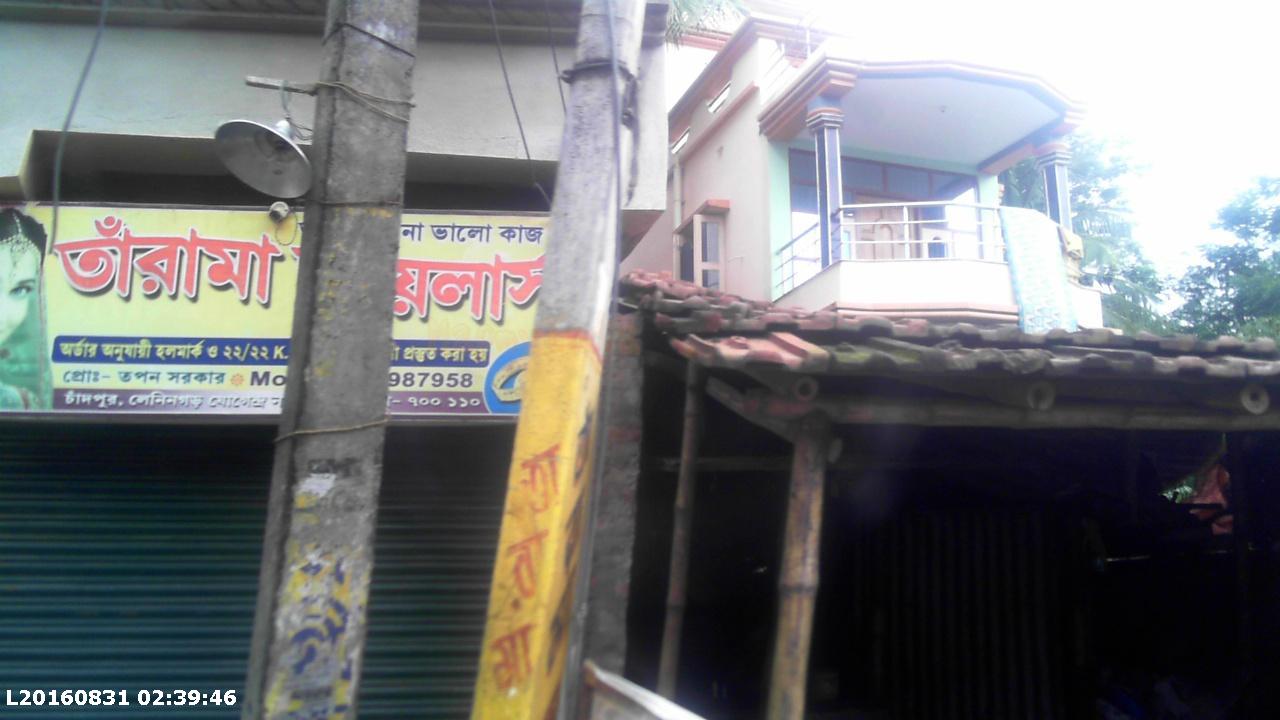

Supplement: Supplementary file 2 — Supplementary Material 2 [file 41598_2026_40742_MOESM2_ESM.zip › sample_data_yolov5/L_08-31_02.39.46.jpg]

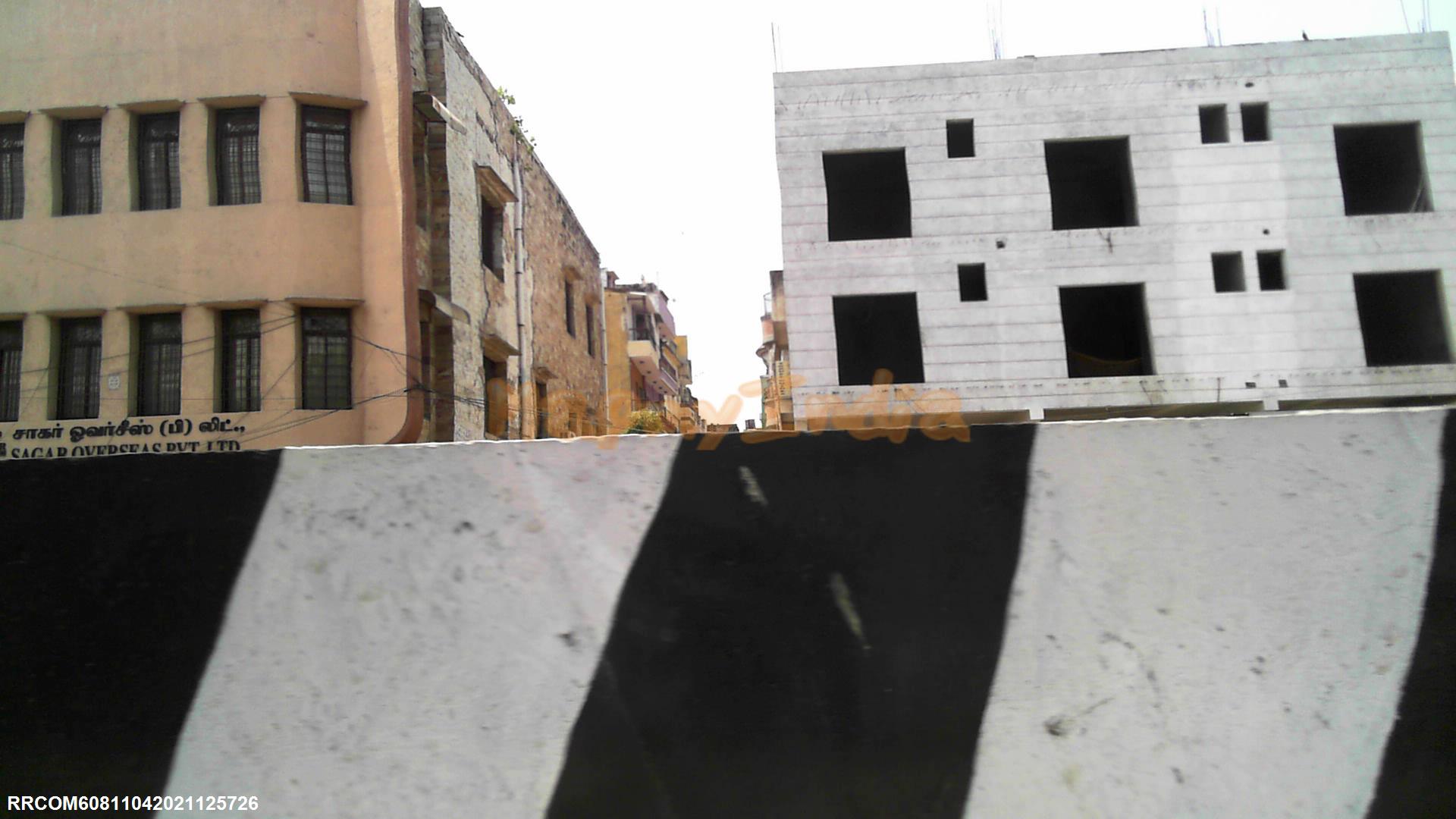

Supplement: Supplementary file 2 — Supplementary Material 2 [file 41598_2026_40742_MOESM2_ESM.zip › sample_data_yolov5/RCOM60811042021125726.jpg]

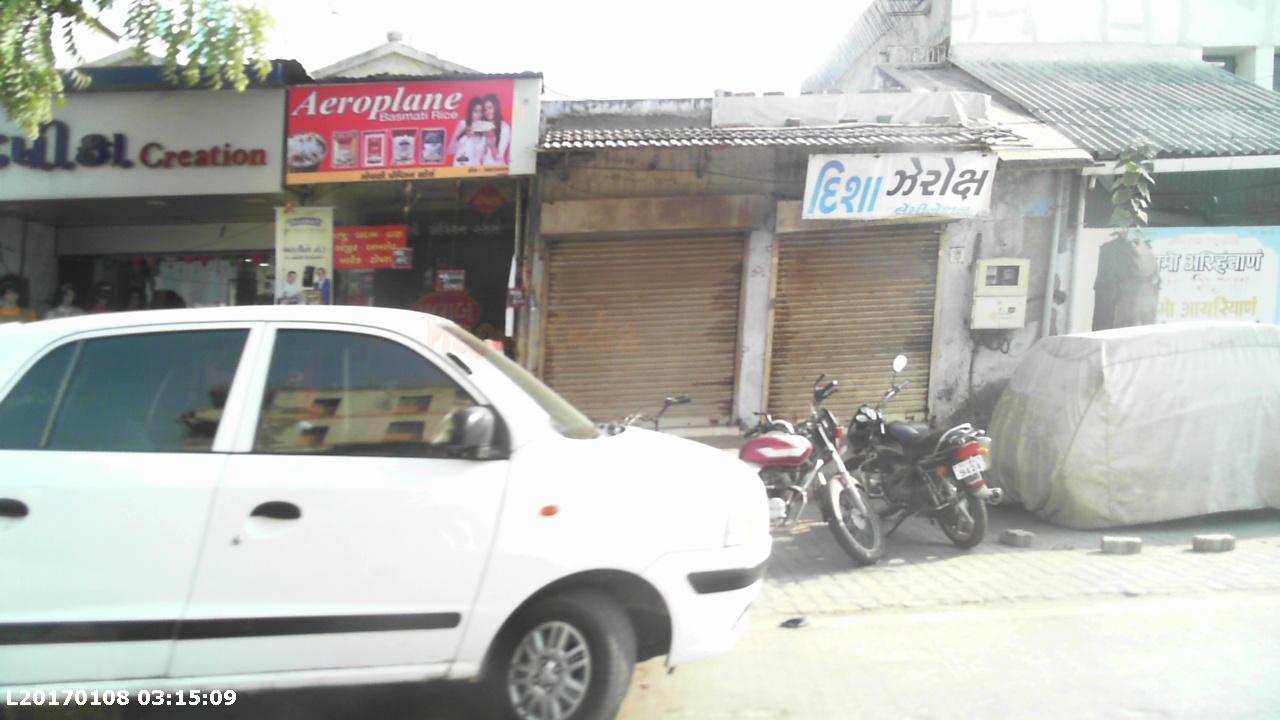

Supplement: Supplementary file 2 — Supplementary Material 2 [file 41598_2026_40742_MOESM2_ESM.zip › sample_data_yolov5/01-08 03.15.09.jpg]

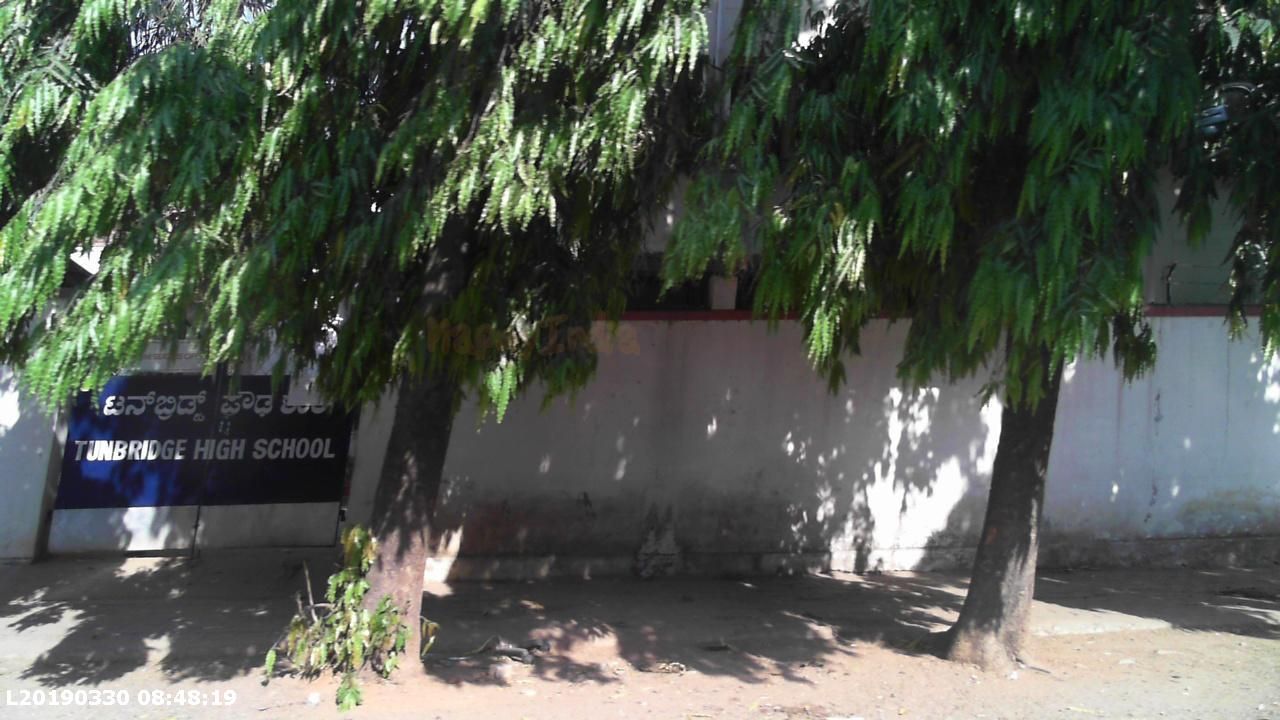

Supplement: Supplementary file 2 — Supplementary Material 2 [file 41598_2026_40742_MOESM2_ESM.zip › sample_data_yolov5/03-30_08.48.19.jpg]

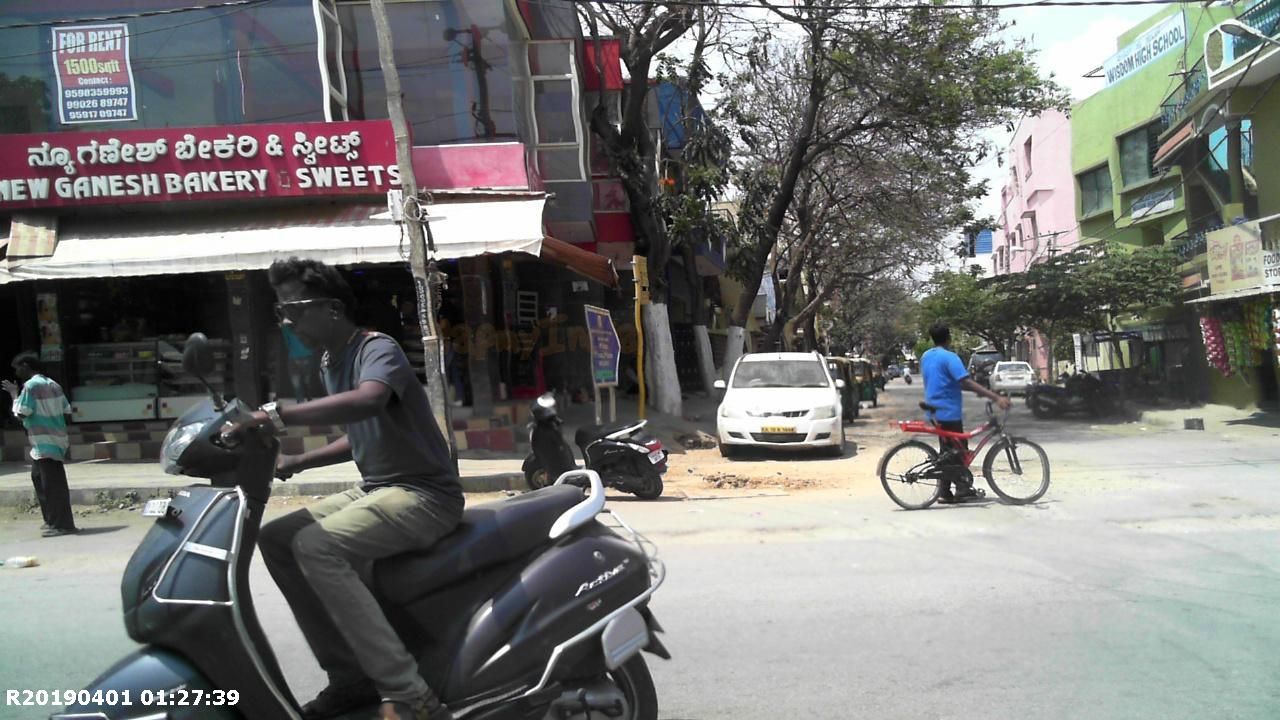

Supplement: Supplementary file 2 — Supplementary Material 2 [file 41598_2026_40742_MOESM2_ESM.zip › sample_data_yolov5/04-01_01.27.39.jpg]

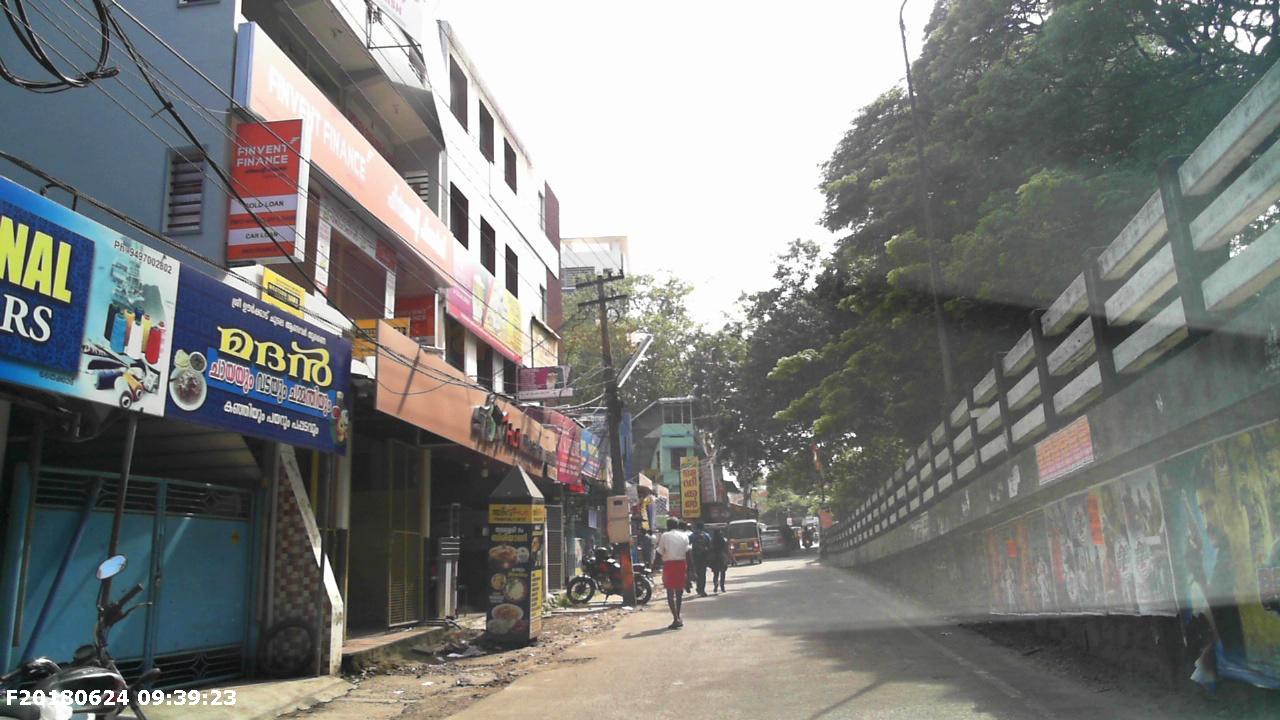

Supplement: Supplementary file 2 — Supplementary Material 2 [file 41598_2026_40742_MOESM2_ESM.zip › sample_data_yolov5/F_06-24_09.39.23.jpg]

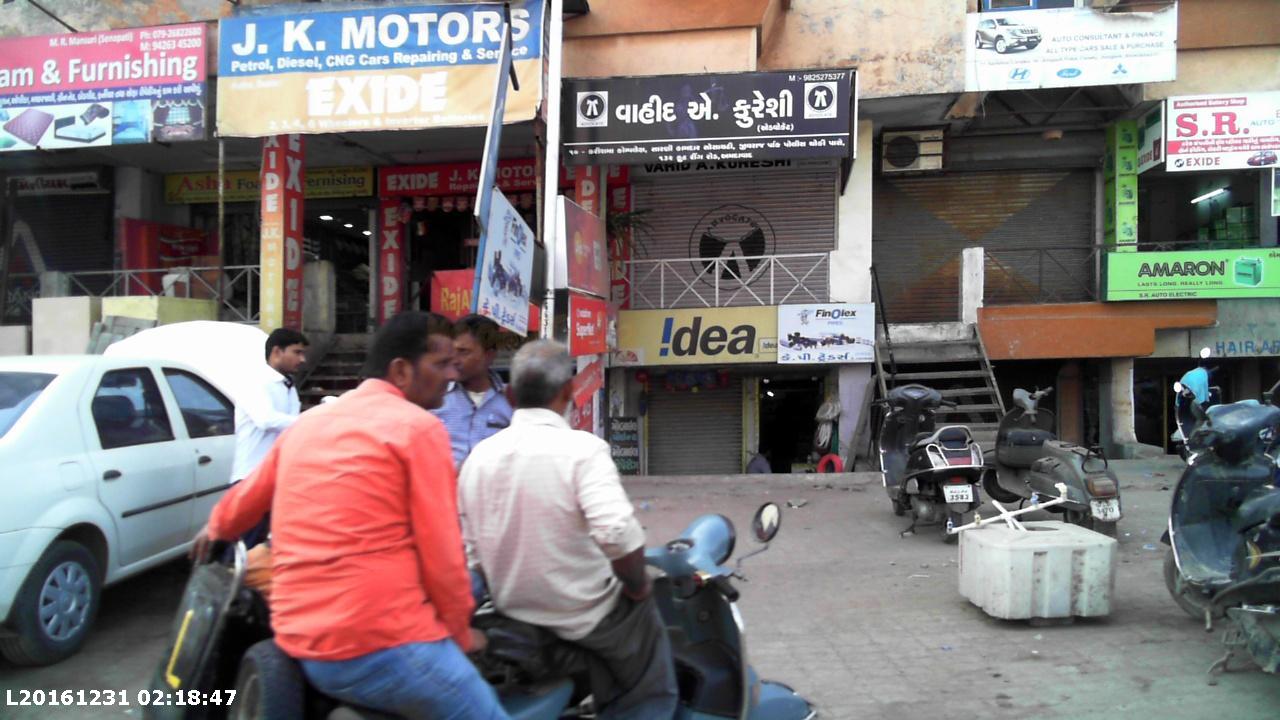

Supplement: Supplementary file 2 — Supplementary Material 2 [file 41598_2026_40742_MOESM2_ESM.zip › sample_data_yolov5/12-31 02.18.47.jpg]

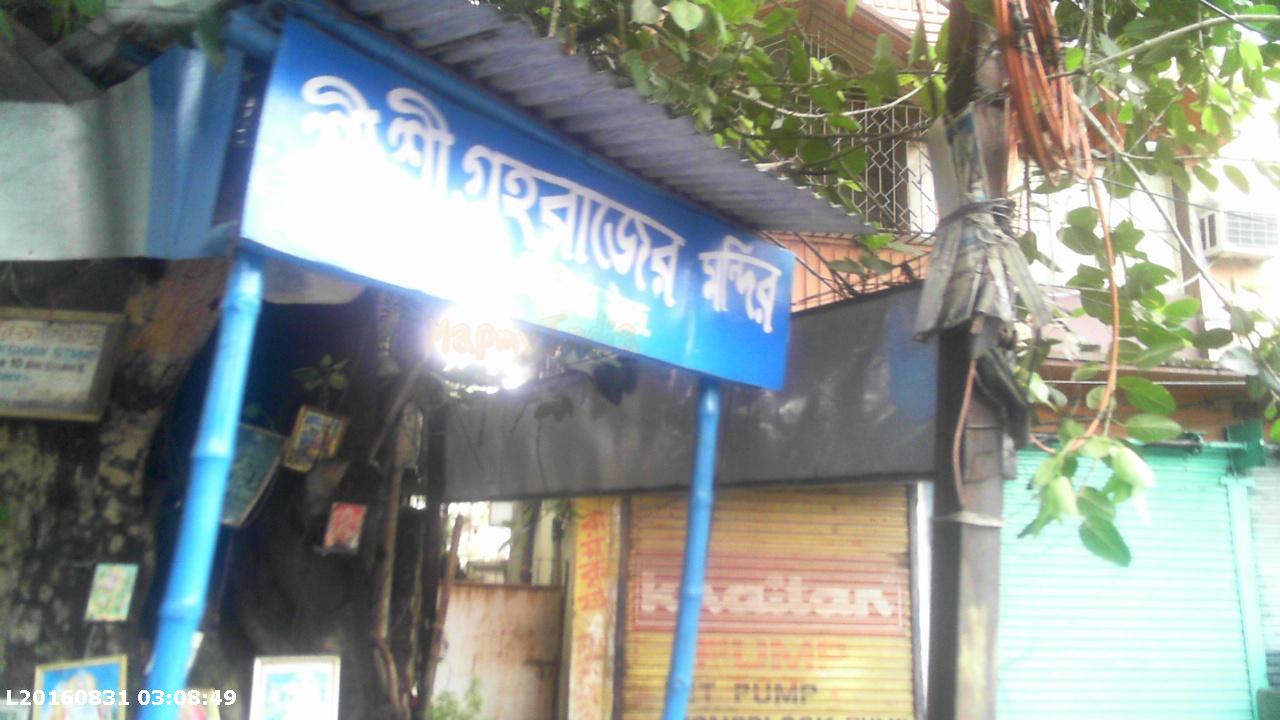

Supplement: Supplementary file 2 — Supplementary Material 2 [file 41598_2026_40742_MOESM2_ESM.zip › sample_data_yolov5/L_08-31_03.08.49.jpg]

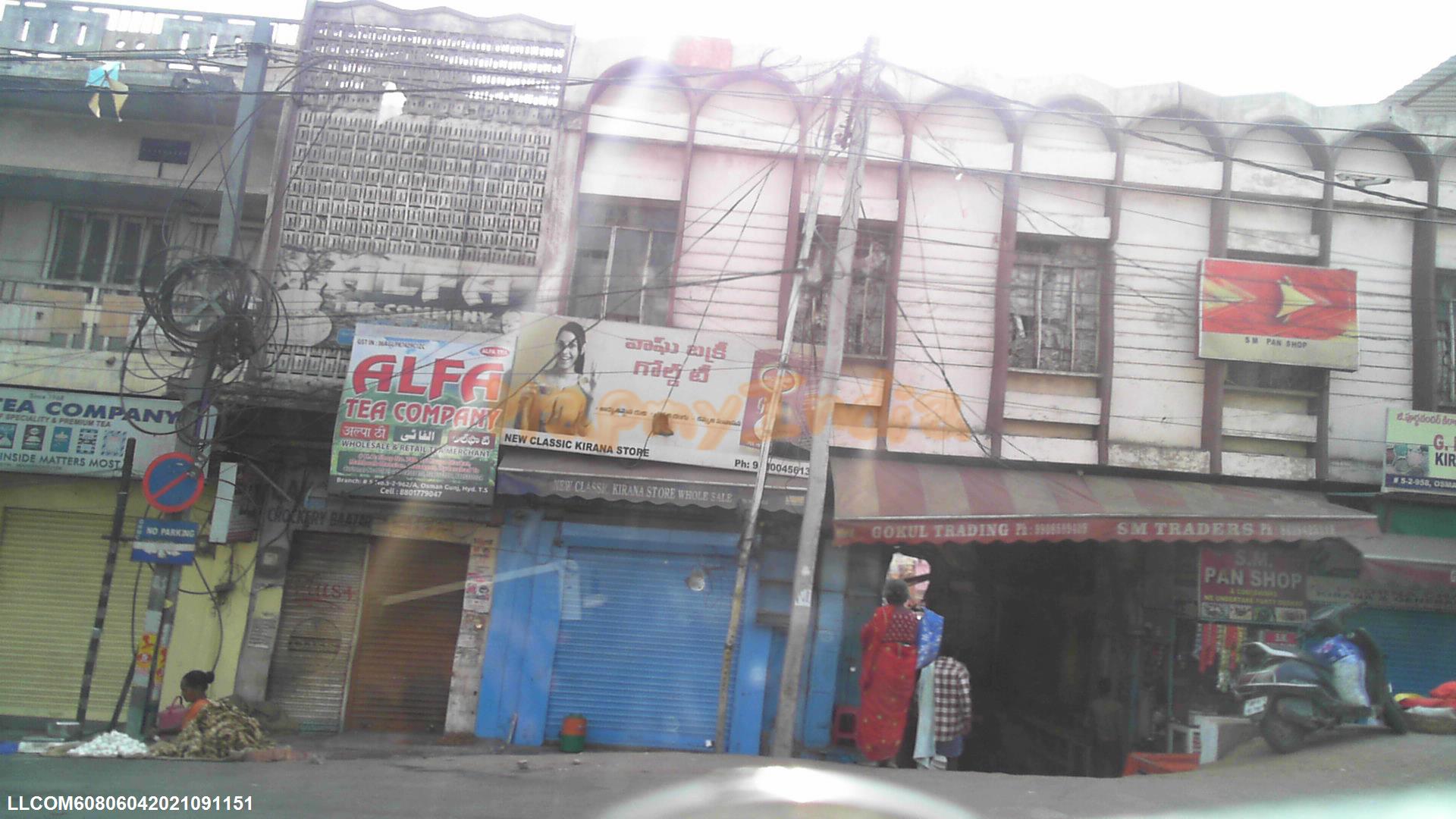

Supplement: Supplementary file 2 — Supplementary Material 2 [file 41598_2026_40742_MOESM2_ESM.zip › sample_data_yolov5/LCOM60806042021091151.jpg]

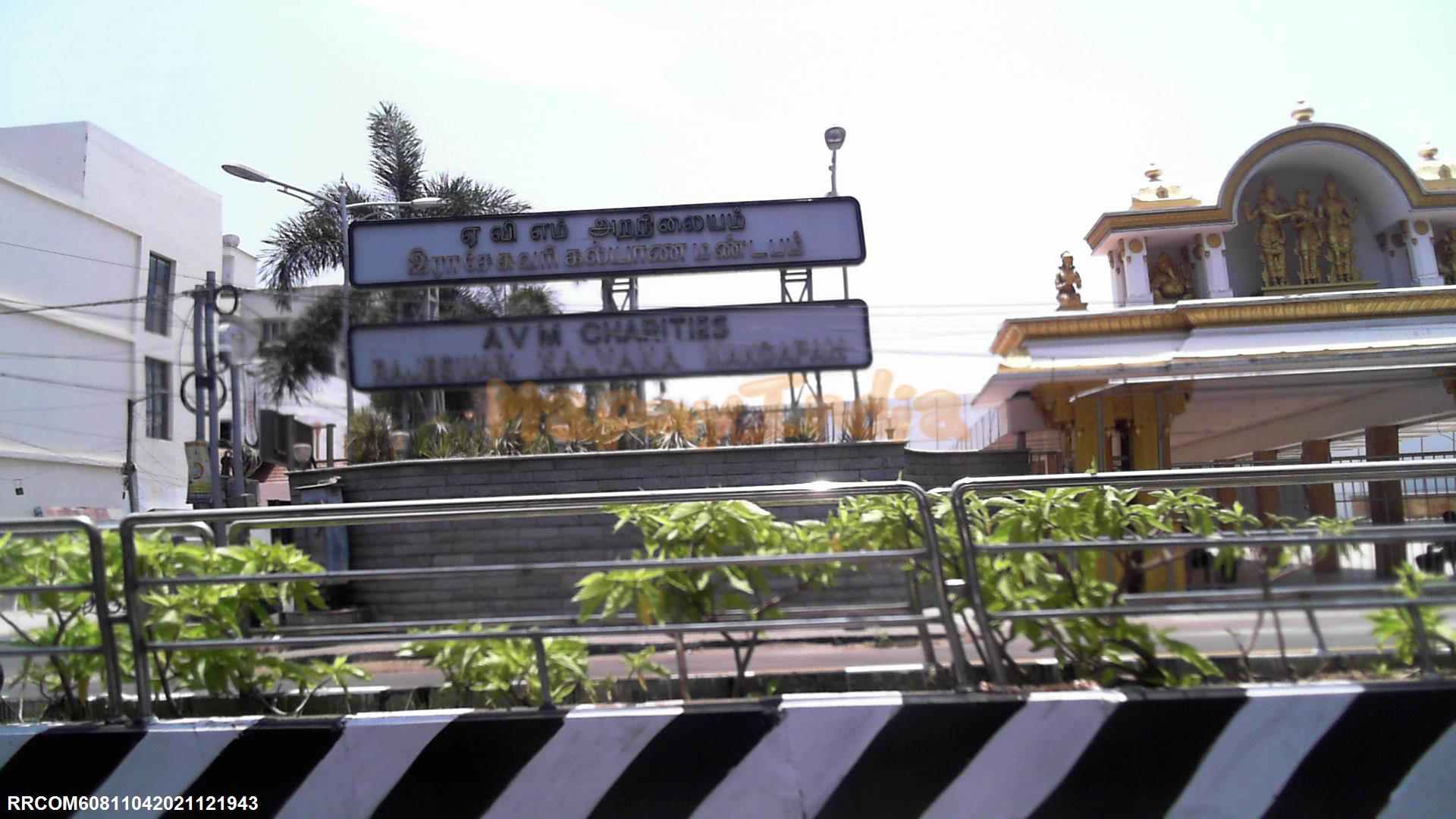

Supplement: Supplementary file 2 — Supplementary Material 2 [file 41598_2026_40742_MOESM2_ESM.zip › sample_data_yolov5/RCOM60811042021121943.jpg]

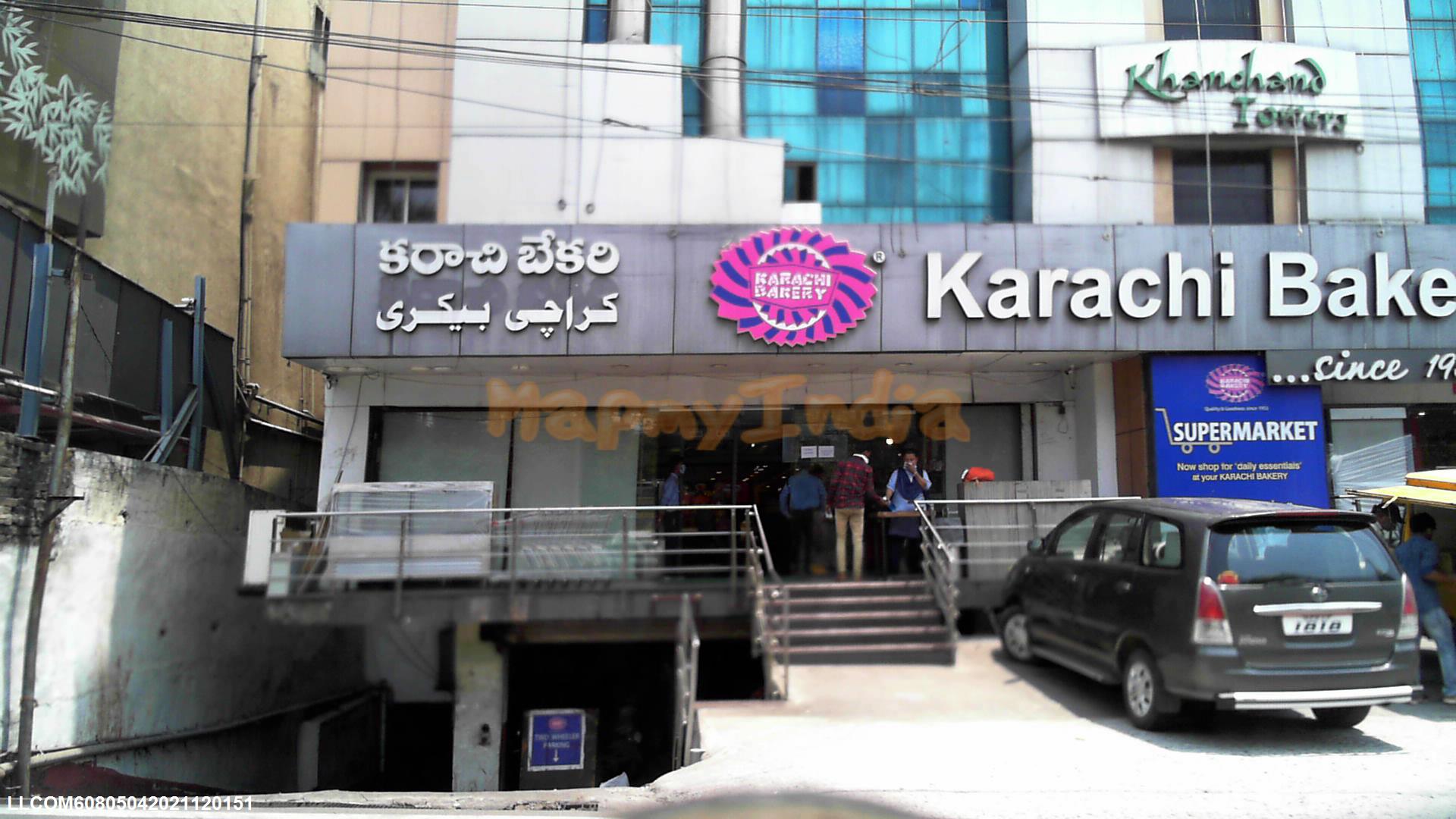

Supplement: Supplementary file 2 — Supplementary Material 2 [file 41598_2026_40742_MOESM2_ESM.zip › sample_data_yolov5/LCOM60805042021120151.jpg]

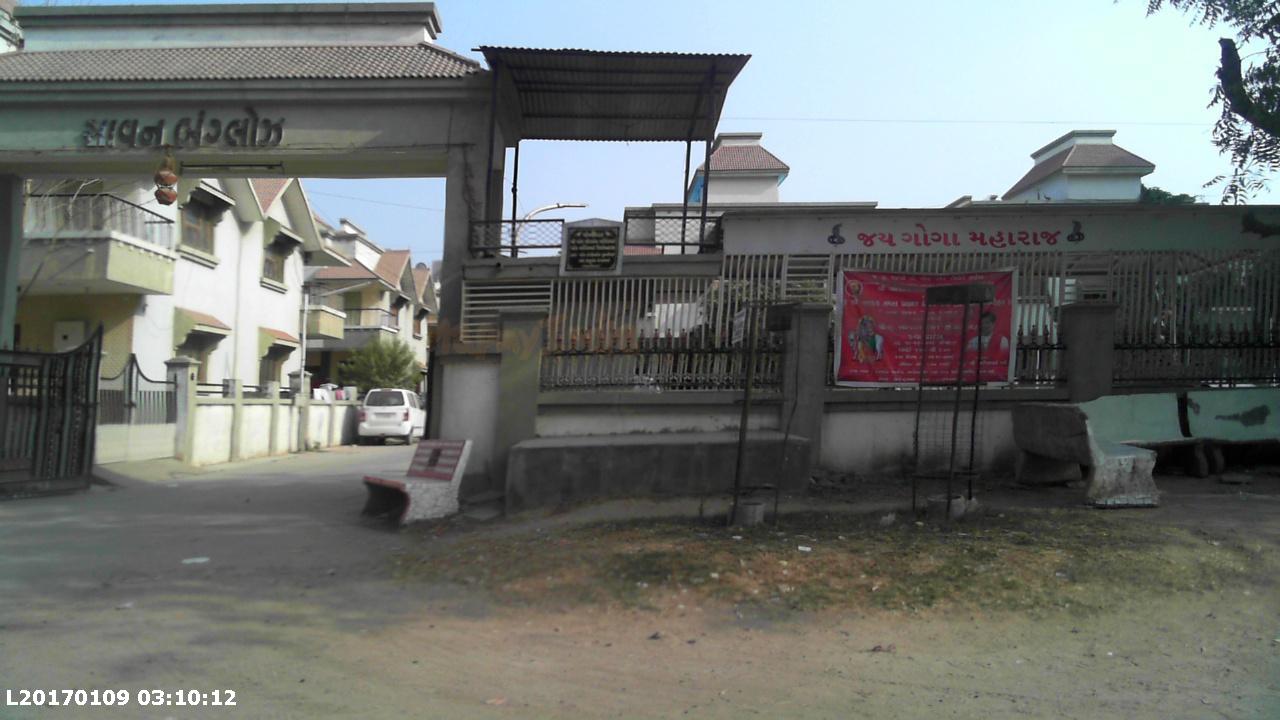

Supplement: Supplementary file 2 — Supplementary Material 2 [file 41598_2026_40742_MOESM2_ESM.zip › sample_data_yolov5/01-09 03.10.12.jpg]

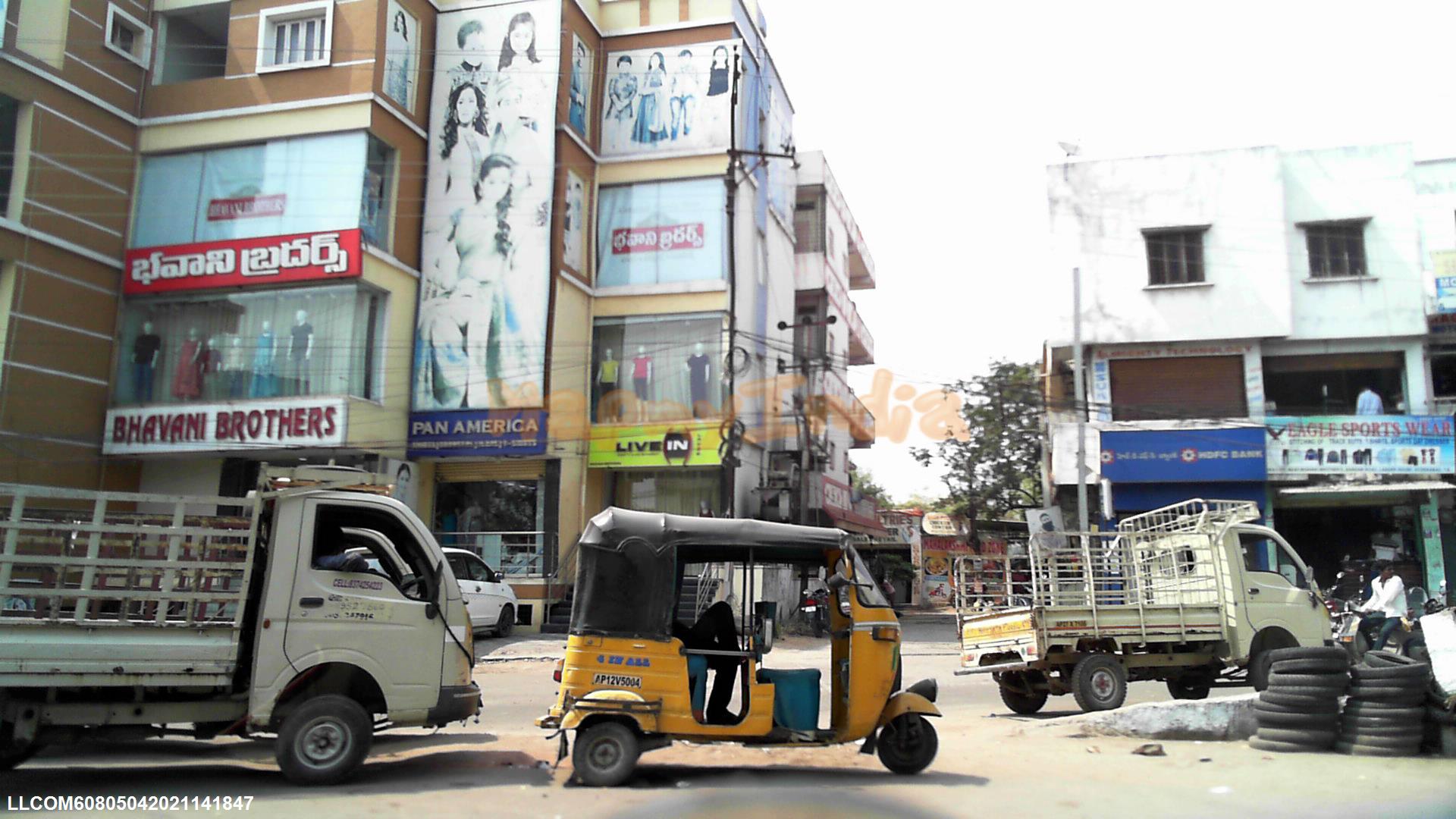

Supplement: Supplementary file 2 — Supplementary Material 2 [file 41598_2026_40742_MOESM2_ESM.zip › sample_data_yolov5/LCOM60805042021141847.jpg]

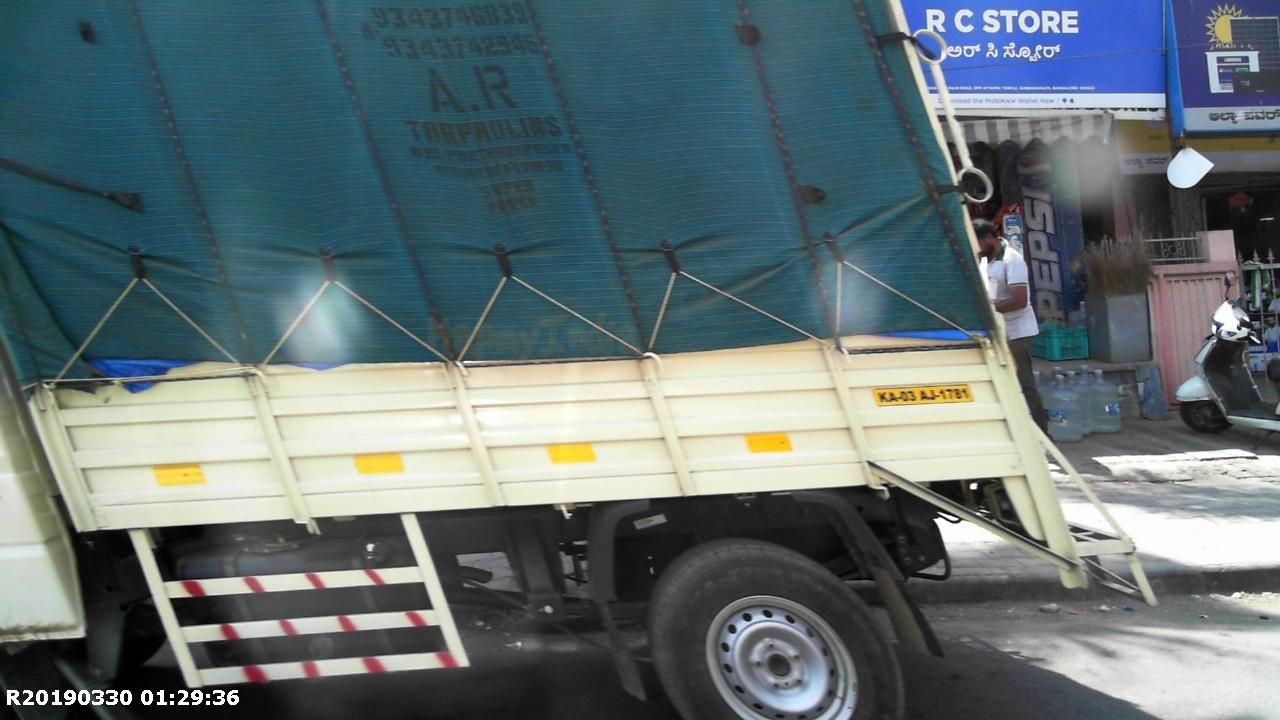

Supplement: Supplementary file 2 — Supplementary Material 2 [file 41598_2026_40742_MOESM2_ESM.zip › sample_data_yolov5/03-30_01.29.36.jpg]

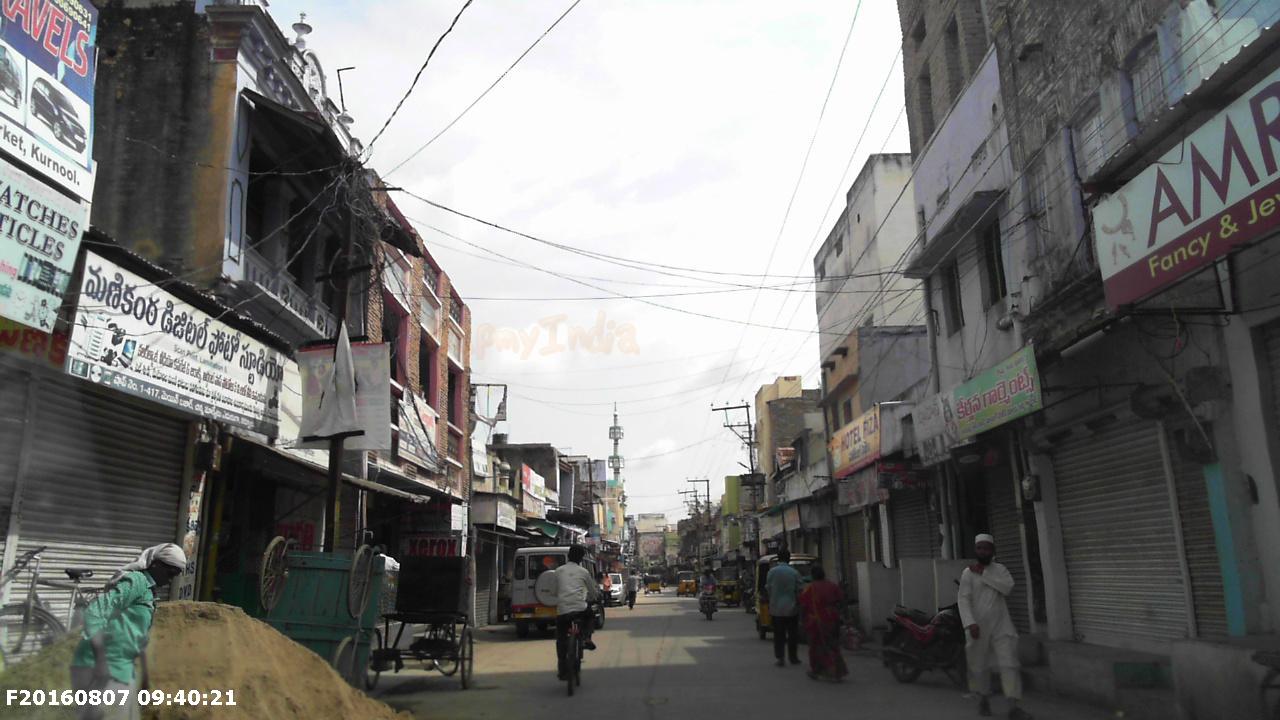

Supplement: Supplementary file 2 — Supplementary Material 2 [file 41598_2026_40742_MOESM2_ESM.zip › sample_data_yolov5/08-07 09.40.21.jpg]

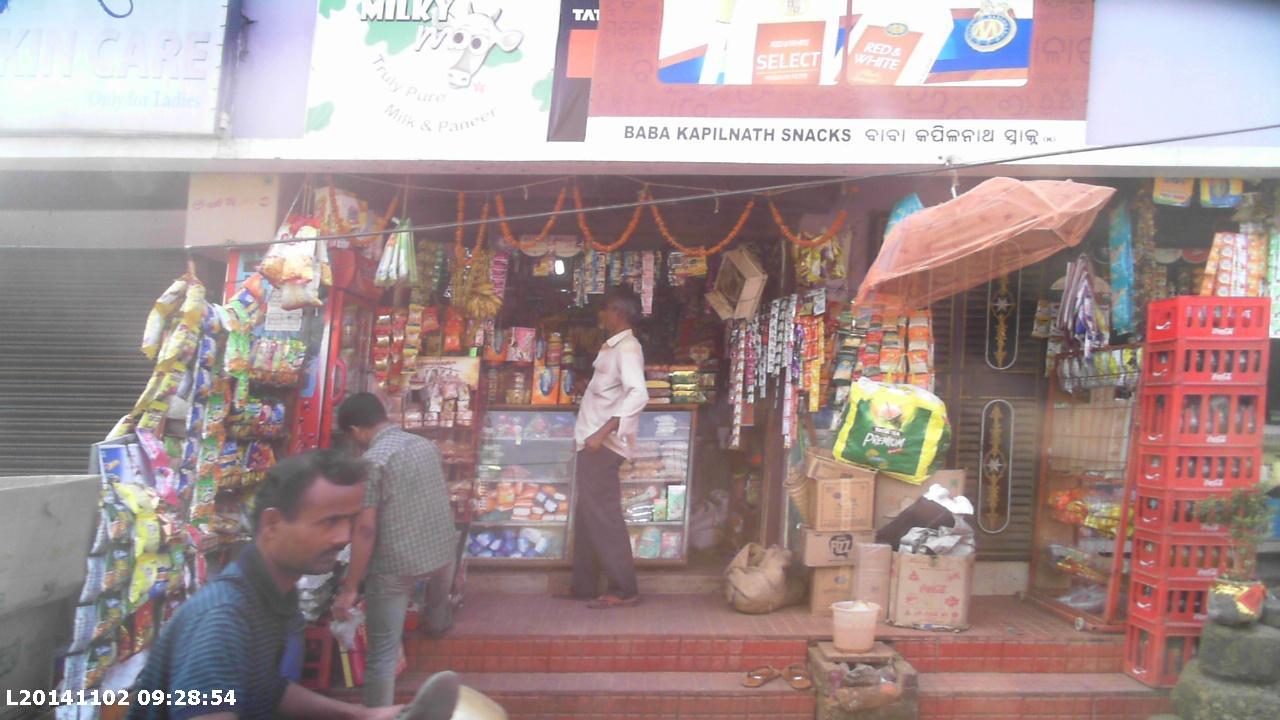

Supplement: Supplementary file 2 — Supplementary Material 2 [file 41598_2026_40742_MOESM2_ESM.zip › sample_data_yolov5/L_11-02_09.28.54.jpg]

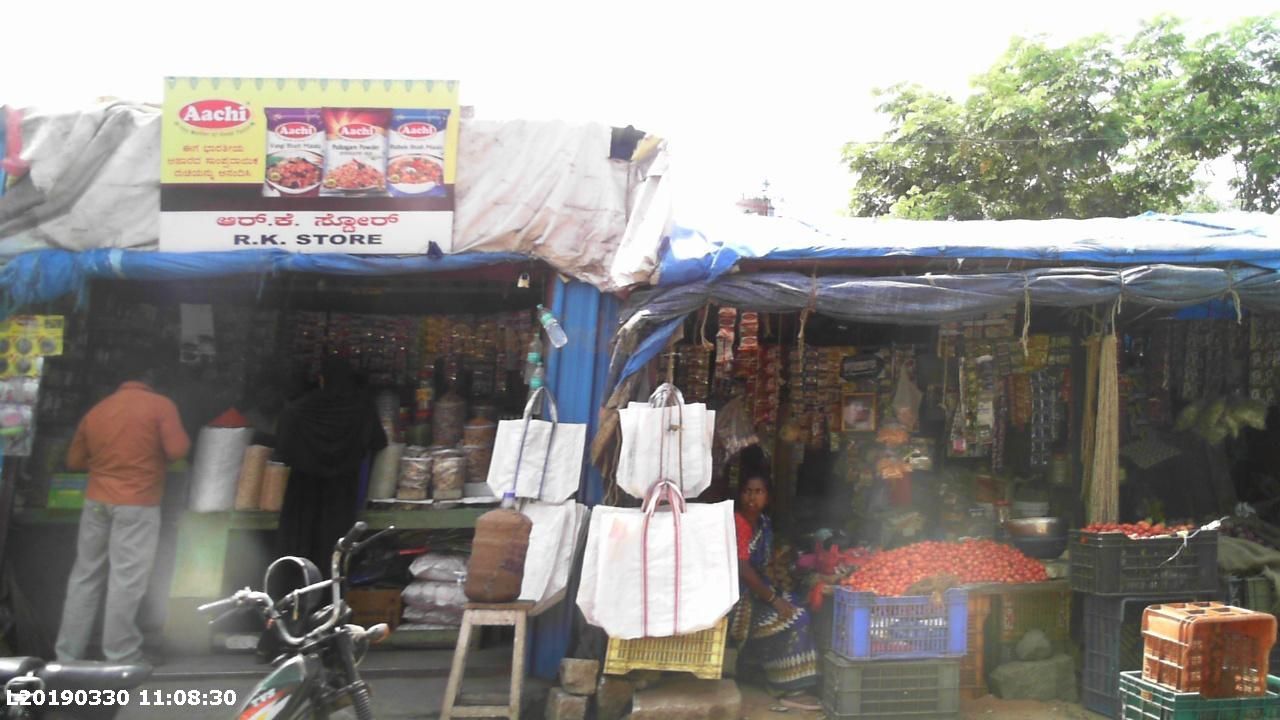

Supplement: Supplementary file 2 — Supplementary Material 2 [file 41598_2026_40742_MOESM2_ESM.zip › sample_data_yolov5/03-30_11.08.30.jpg]

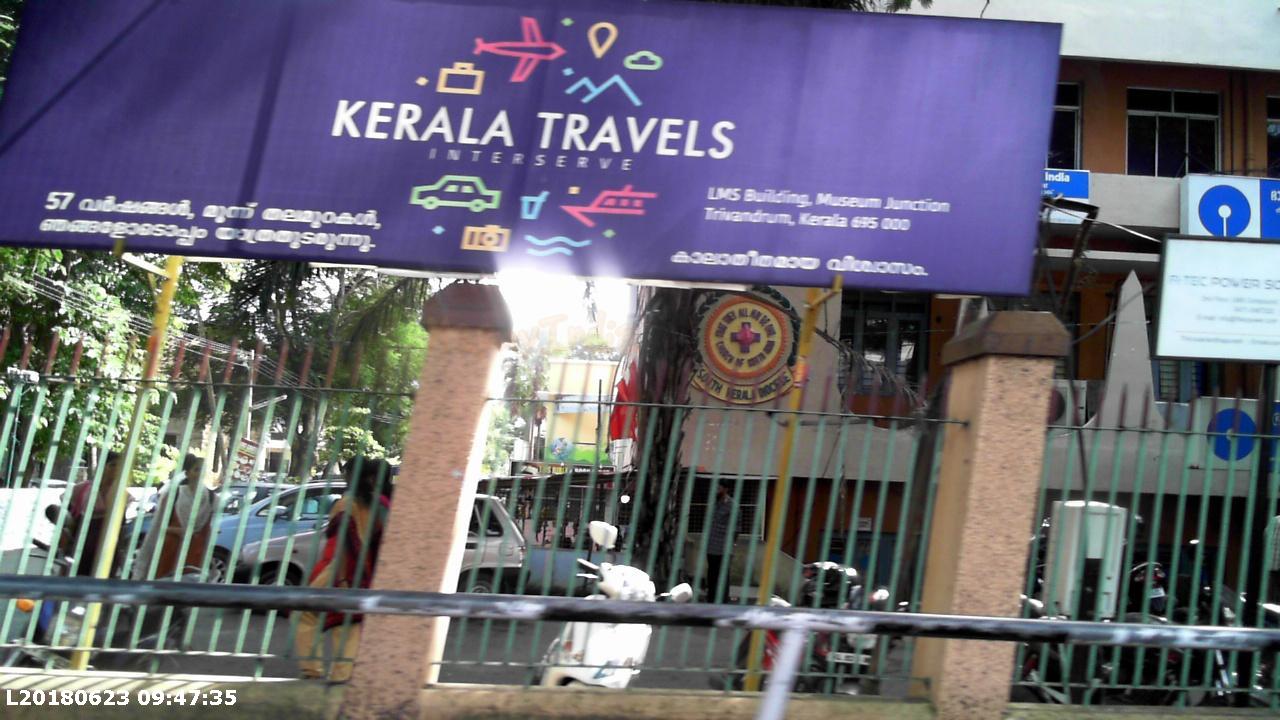

Supplement: Supplementary file 2 — Supplementary Material 2 [file 41598_2026_40742_MOESM2_ESM.zip › sample_data_yolov5/L_06-23_09.47.35.jpg]

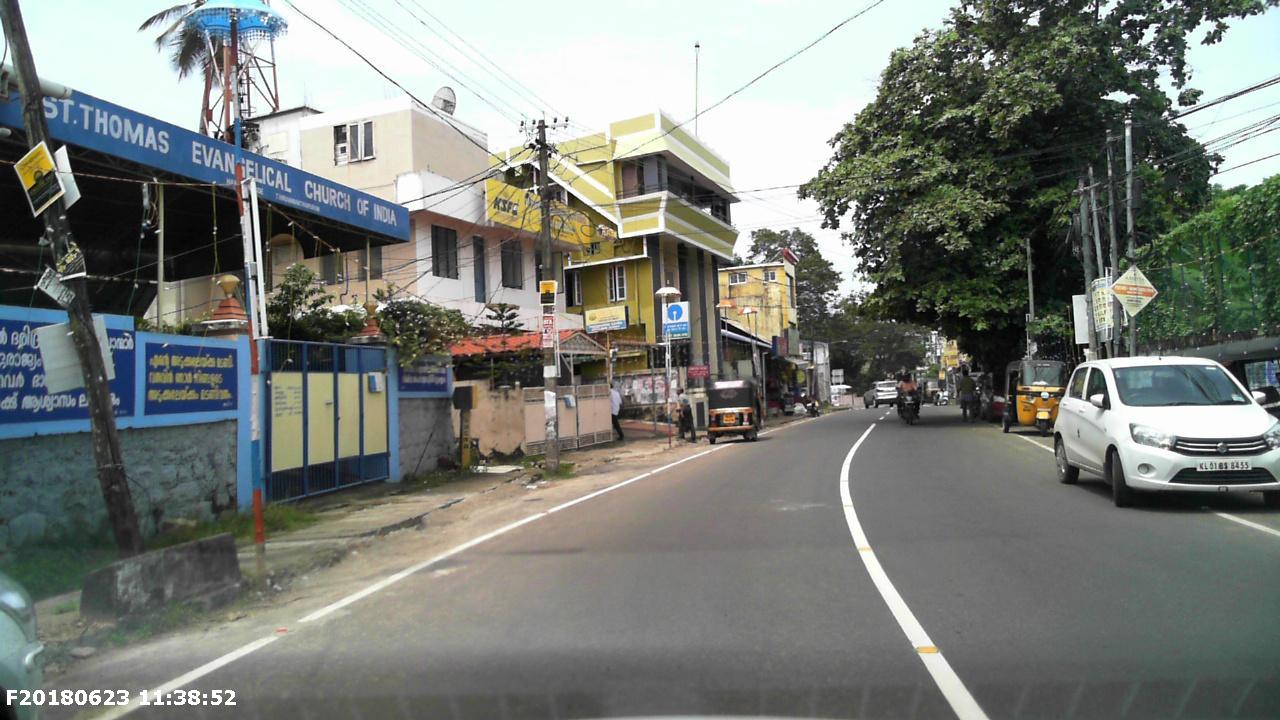

Supplement: Supplementary file 2 — Supplementary Material 2 [file 41598_2026_40742_MOESM2_ESM.zip › sample_data_yolov5/F_06-23_11.38.52.jpg]

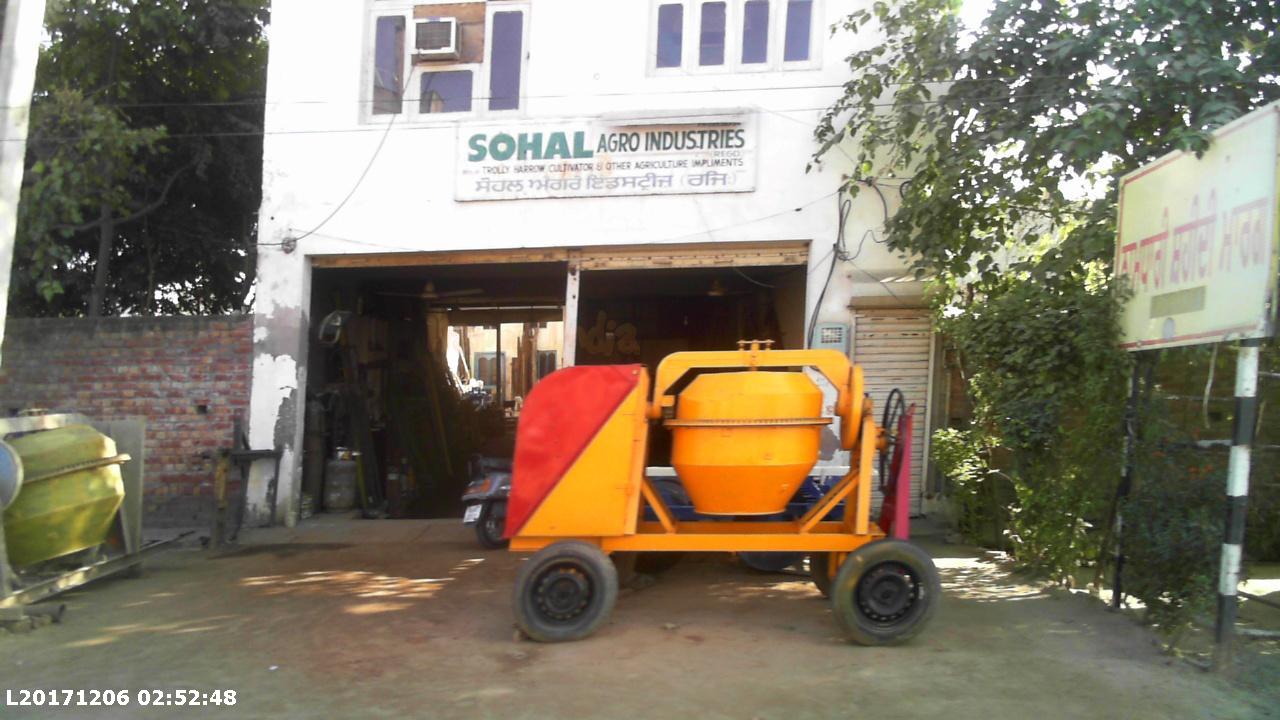

Supplement: Supplementary file 2 — Supplementary Material 2 [file 41598_2026_40742_MOESM2_ESM.zip › sample_data_yolov5/12-06 02.52.48.jpg]

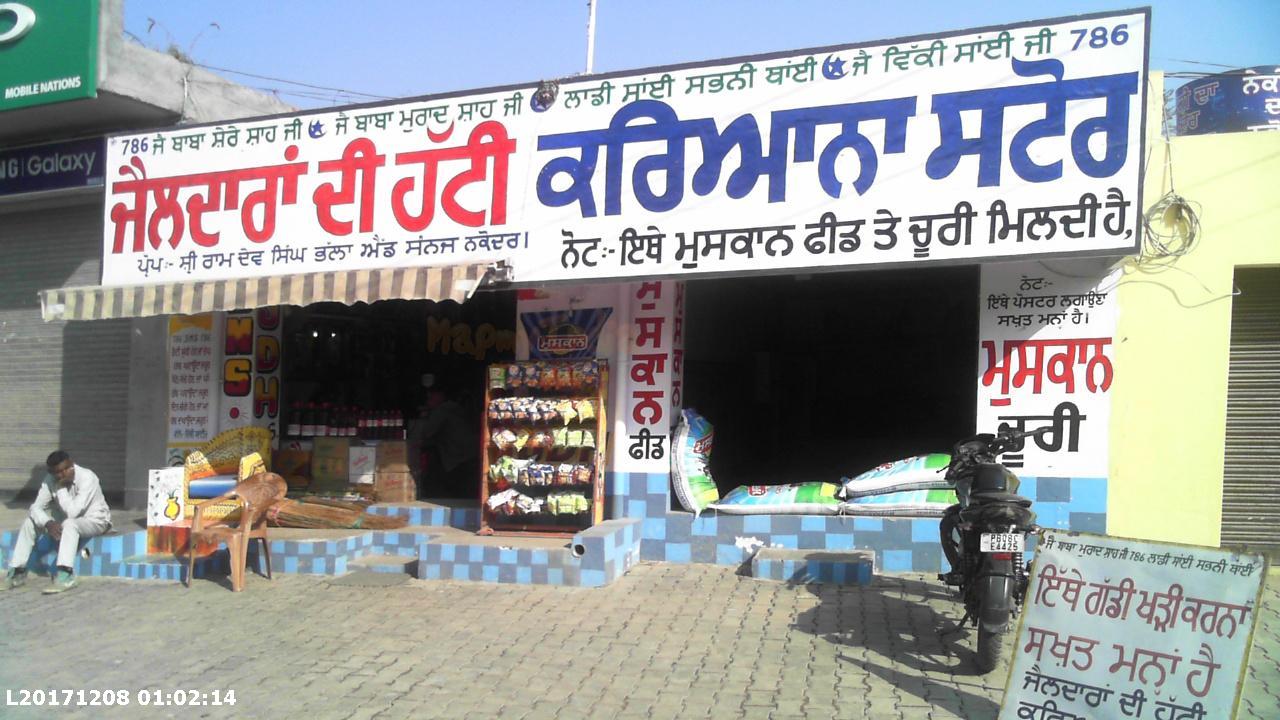

Supplement: Supplementary file 2 — Supplementary Material 2 [file 41598_2026_40742_MOESM2_ESM.zip › sample_data_yolov5/12-08 01.02.14.jpg]

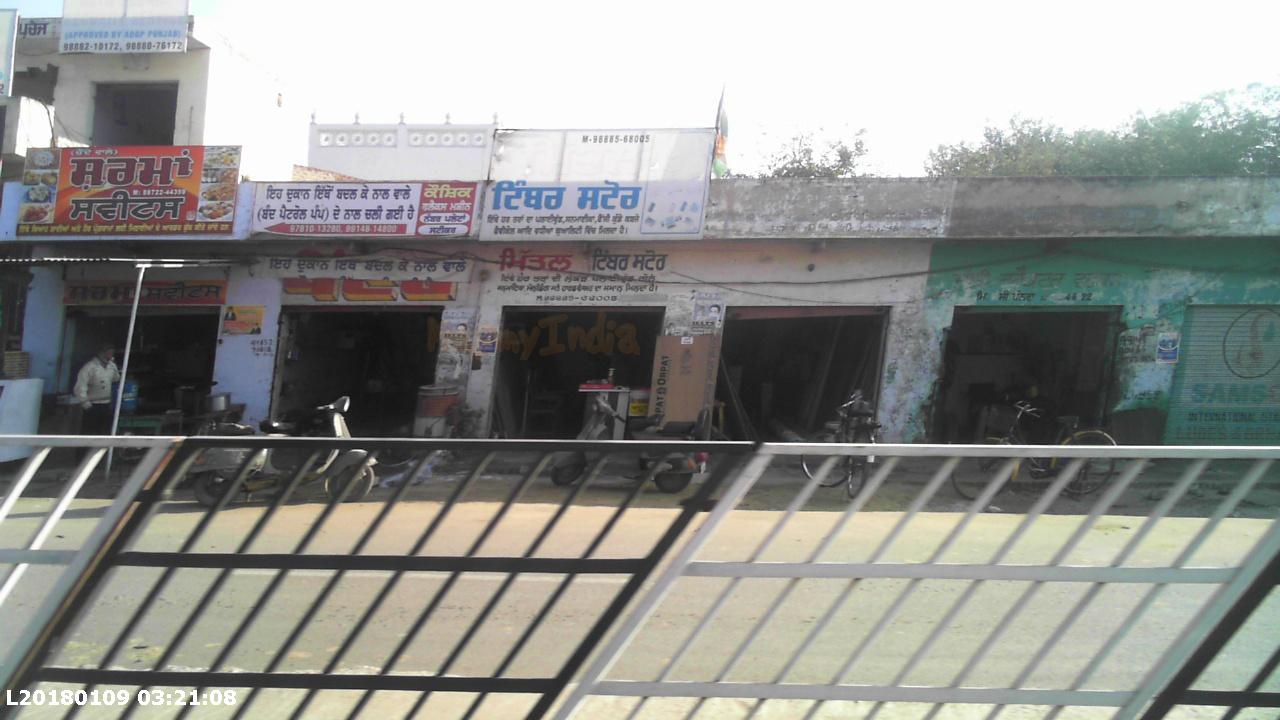

Supplement: Supplementary file 2 — Supplementary Material 2 [file 41598_2026_40742_MOESM2_ESM.zip › sample_data_yolov5/01-09 03.21.08.jpg]

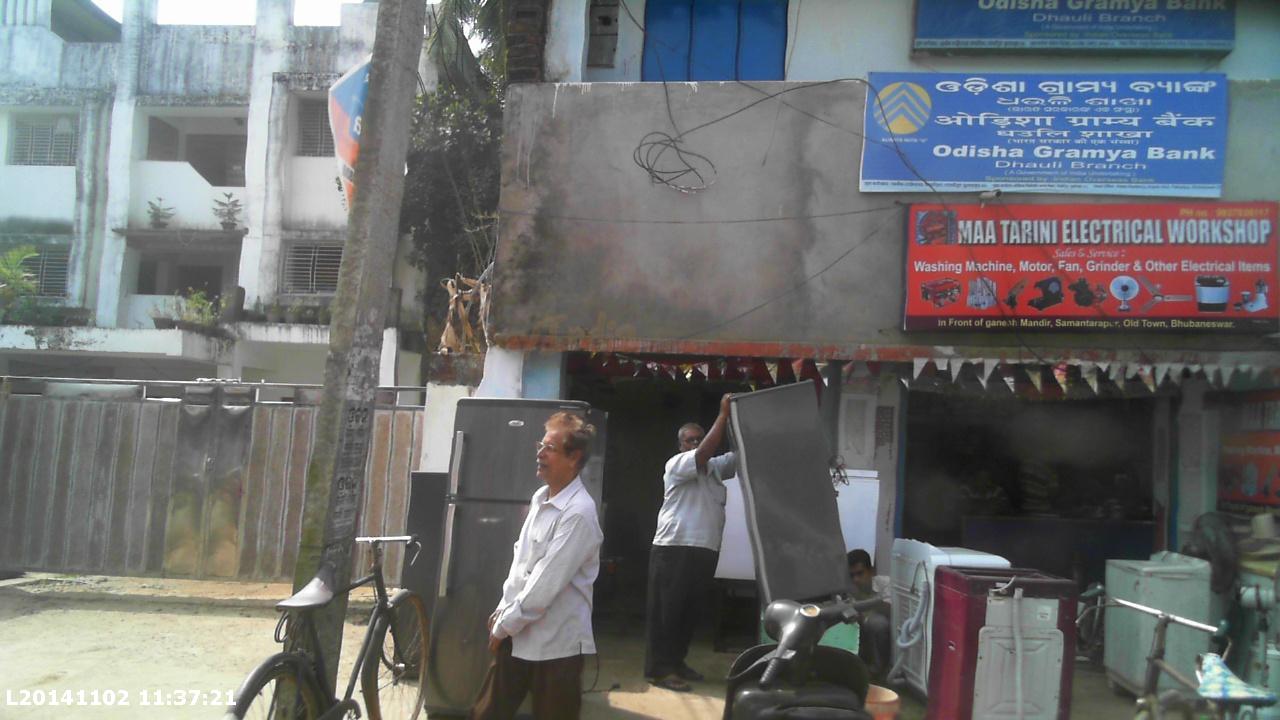

Supplement: Supplementary file 2 — Supplementary Material 2 [file 41598_2026_40742_MOESM2_ESM.zip › sample_data_yolov5/L_11-02_11.37.21.jpg]

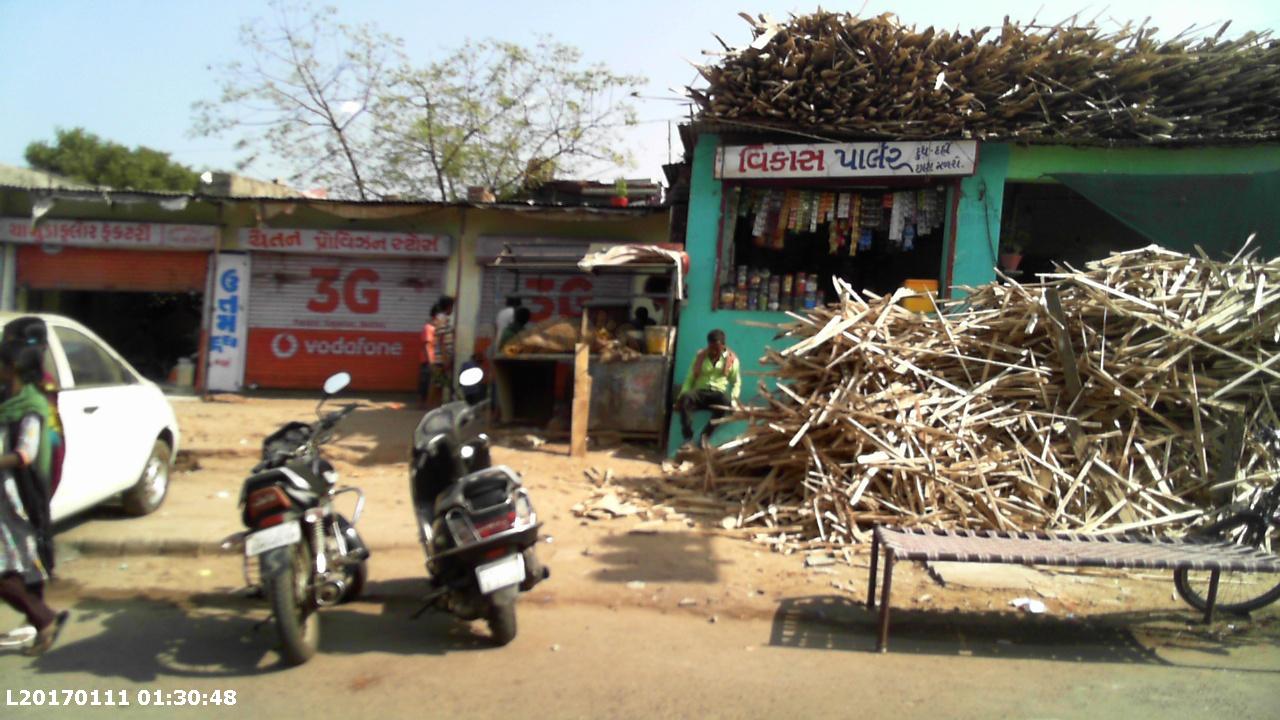

Supplement: Supplementary file 2 — Supplementary Material 2 [file 41598_2026_40742_MOESM2_ESM.zip › sample_data_yolov5/01-11 01.30.48.jpg]

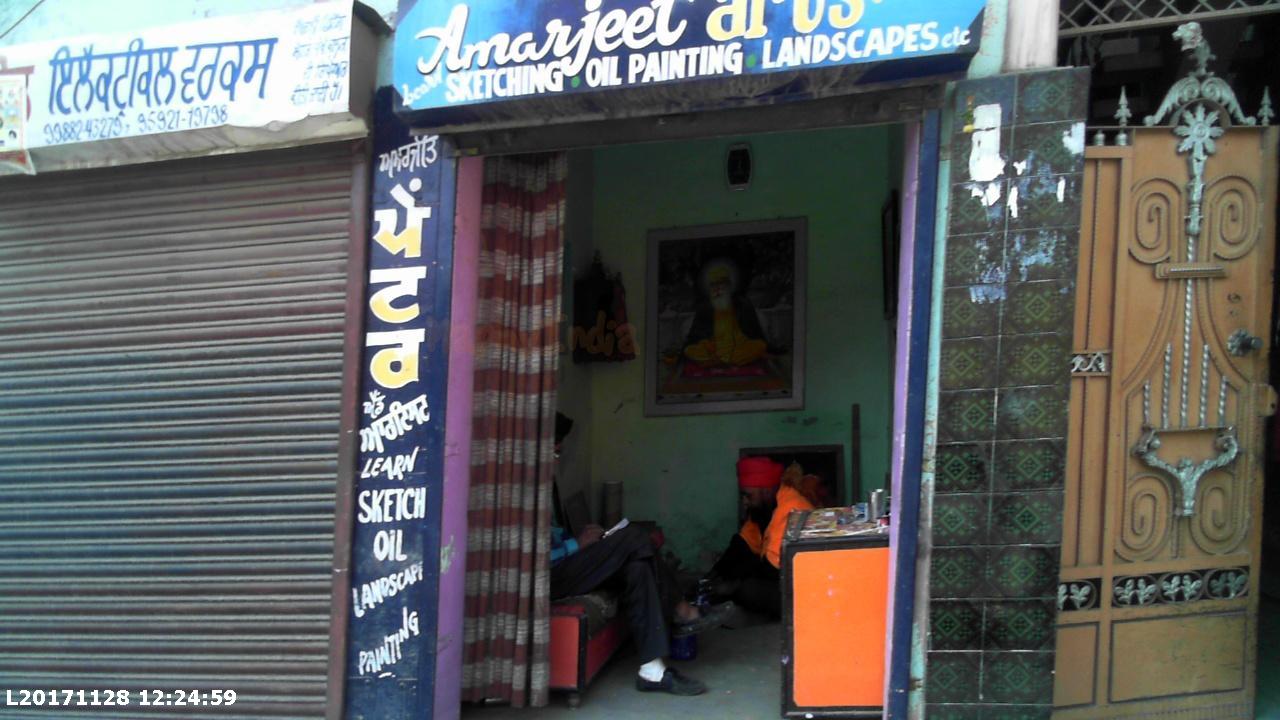

Supplement: Supplementary file 2 — Supplementary Material 2 [file 41598_2026_40742_MOESM2_ESM.zip › sample_data_yolov5/11-28 12.24.59.jpg]

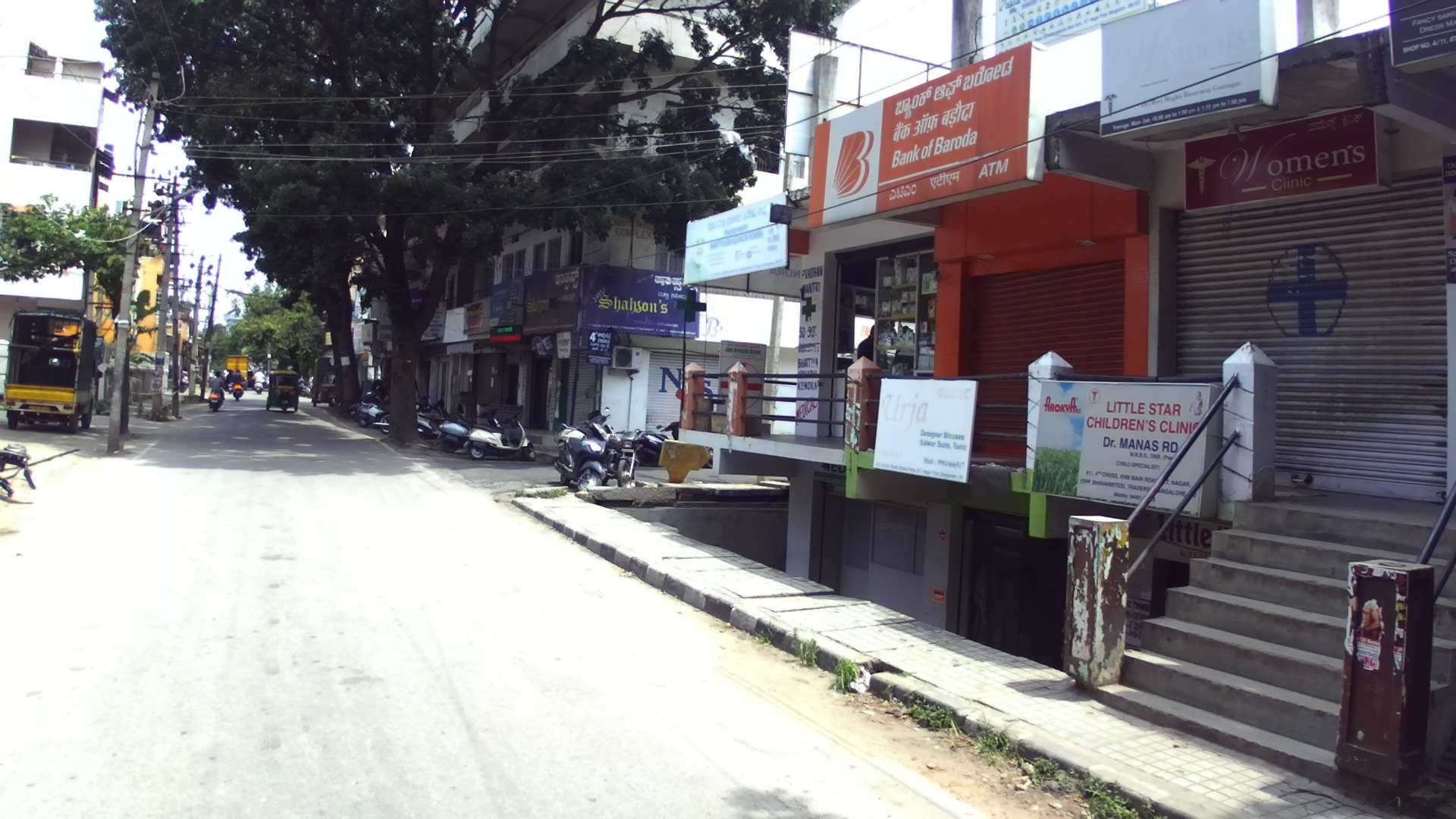

Supplement: Supplementary file 2 — Supplementary Material 2 [file 41598_2026_40742_MOESM2_ESM.zip › sample_data_yolov5/T1_051020_114050_24376_zed_l_299.jpg]

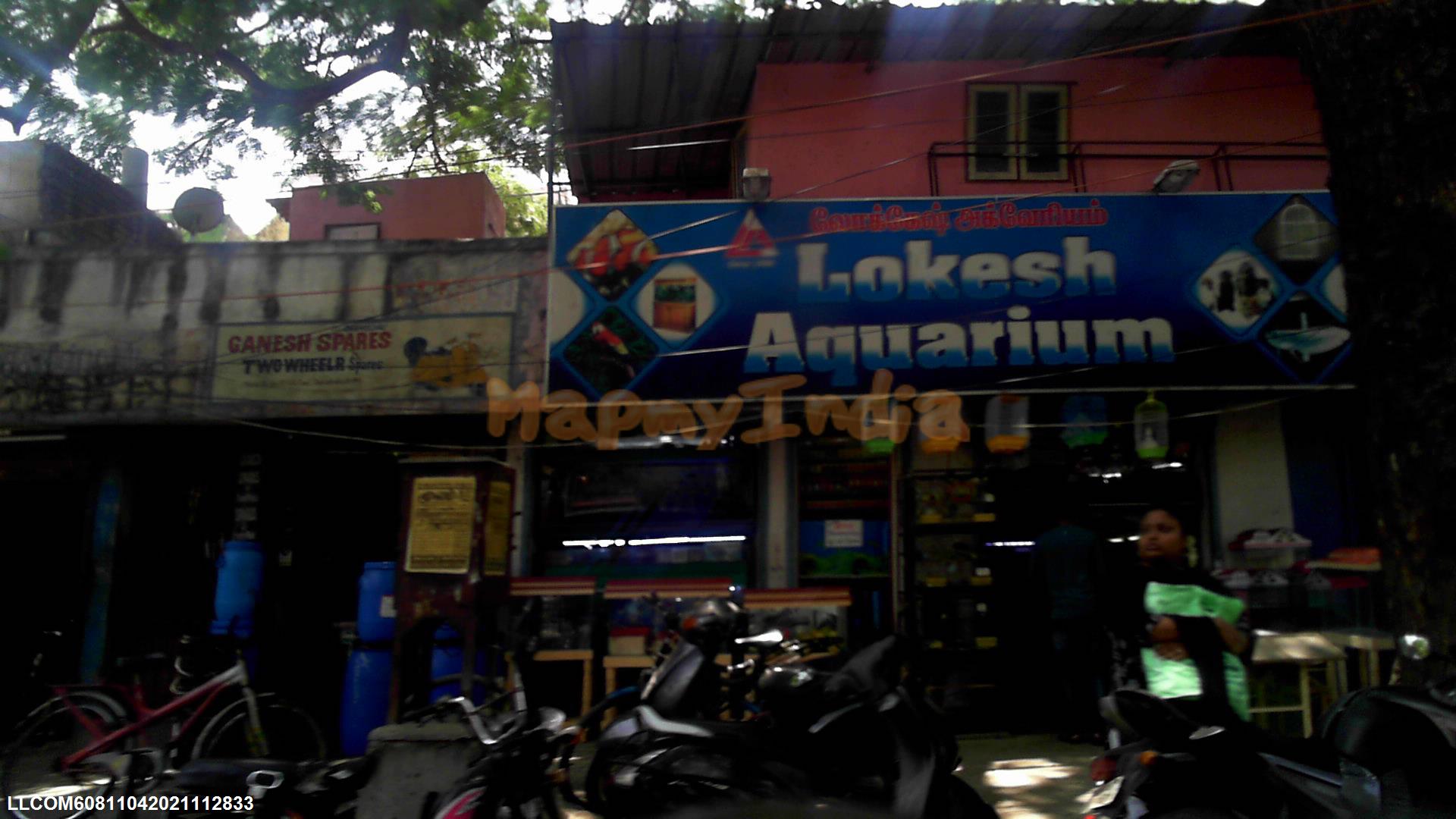

Supplement: Supplementary file 2 — Supplementary Material 2 [file 41598_2026_40742_MOESM2_ESM.zip › sample_data_yolov5/LCOM60811042021112833.jpg]

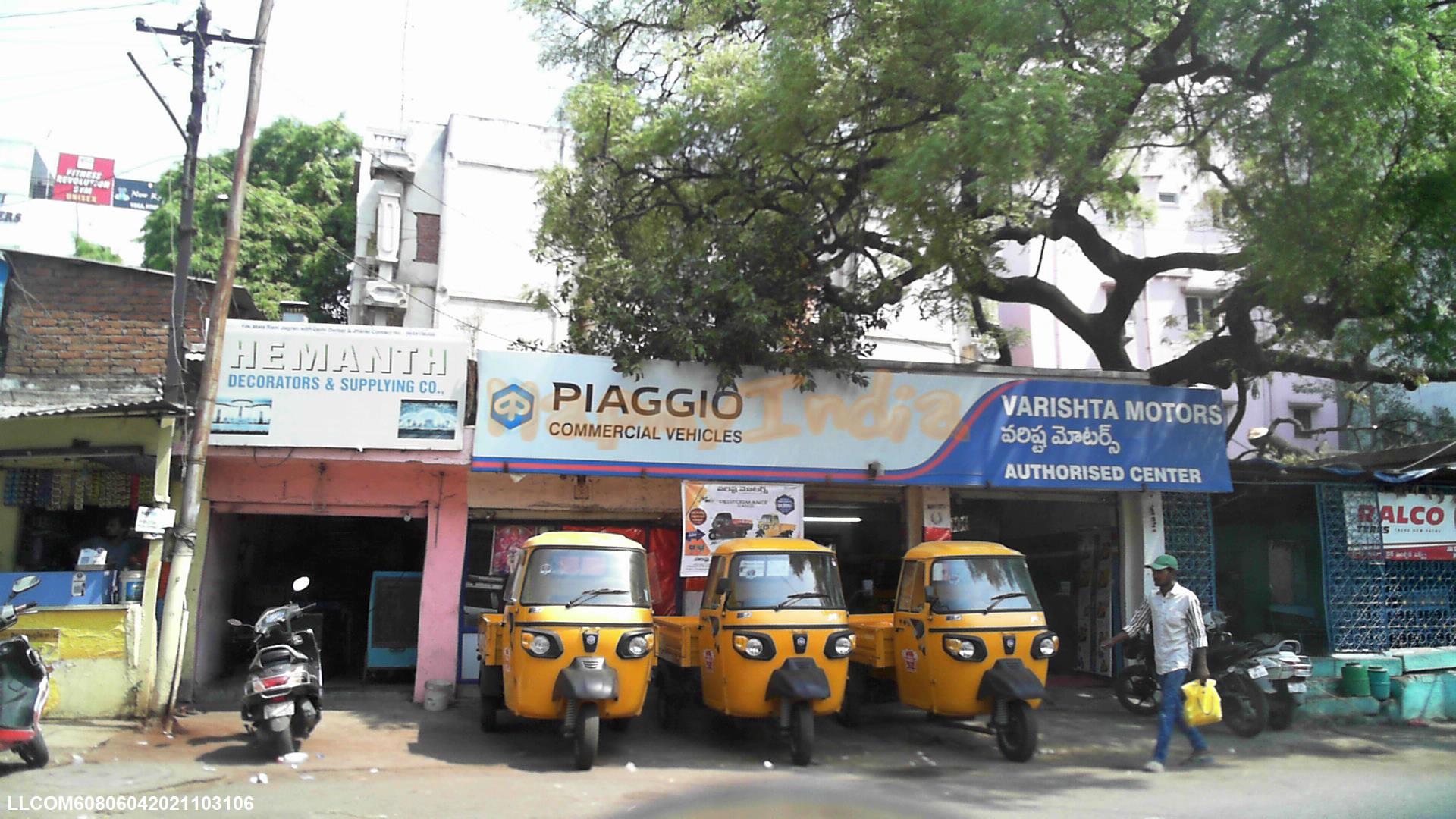

Supplement: Supplementary file 2 — Supplementary Material 2 [file 41598_2026_40742_MOESM2_ESM.zip › sample_data_yolov5/LCOM60806042021103106.jpg]

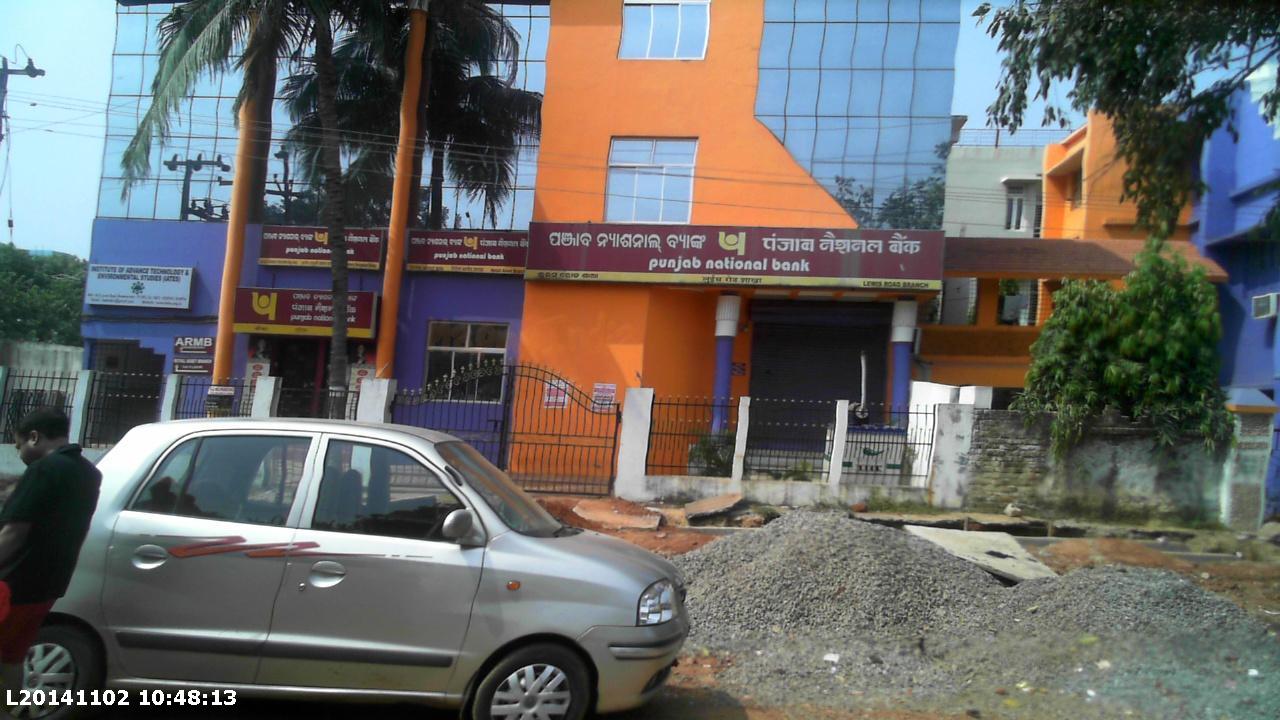

Supplement: Supplementary file 2 — Supplementary Material 2 [file 41598_2026_40742_MOESM2_ESM.zip › sample_data_yolov5/L_11-02_10.48.13.jpg]

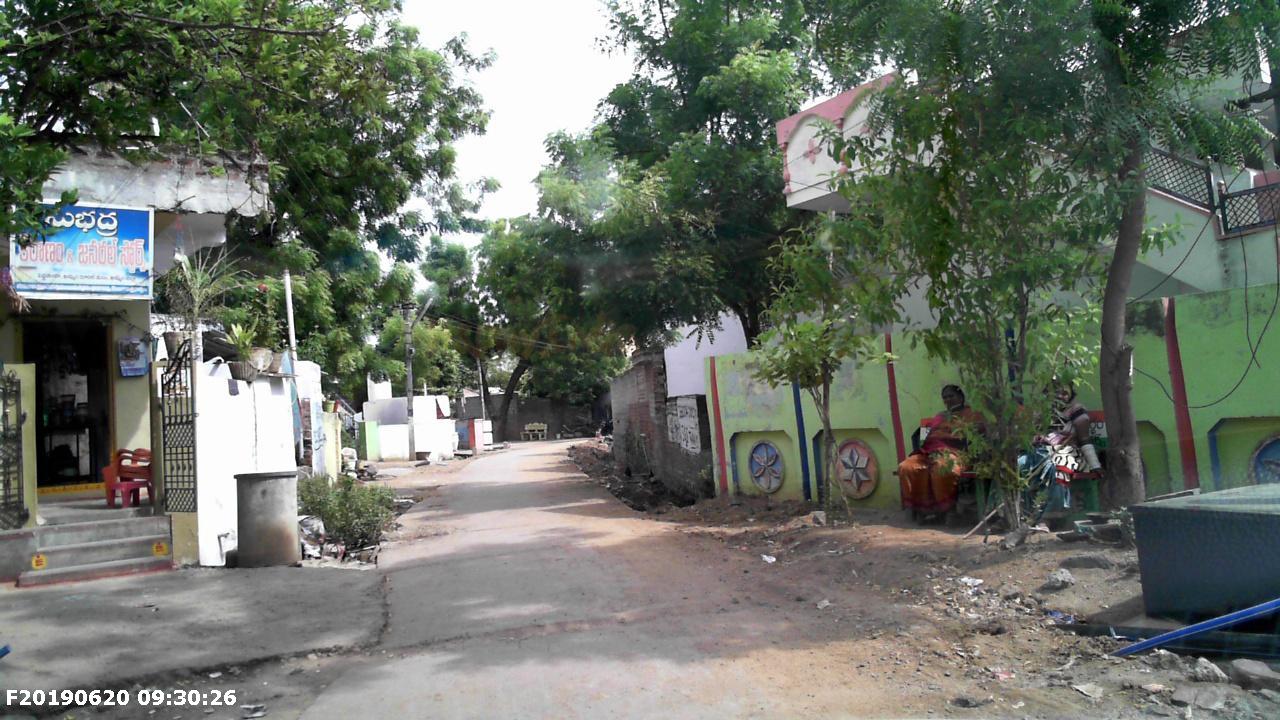

Supplement: Supplementary file 2 — Supplementary Material 2 [file 41598_2026_40742_MOESM2_ESM.zip › sample_data_yolov5/06-20 09.30.26.jpg]

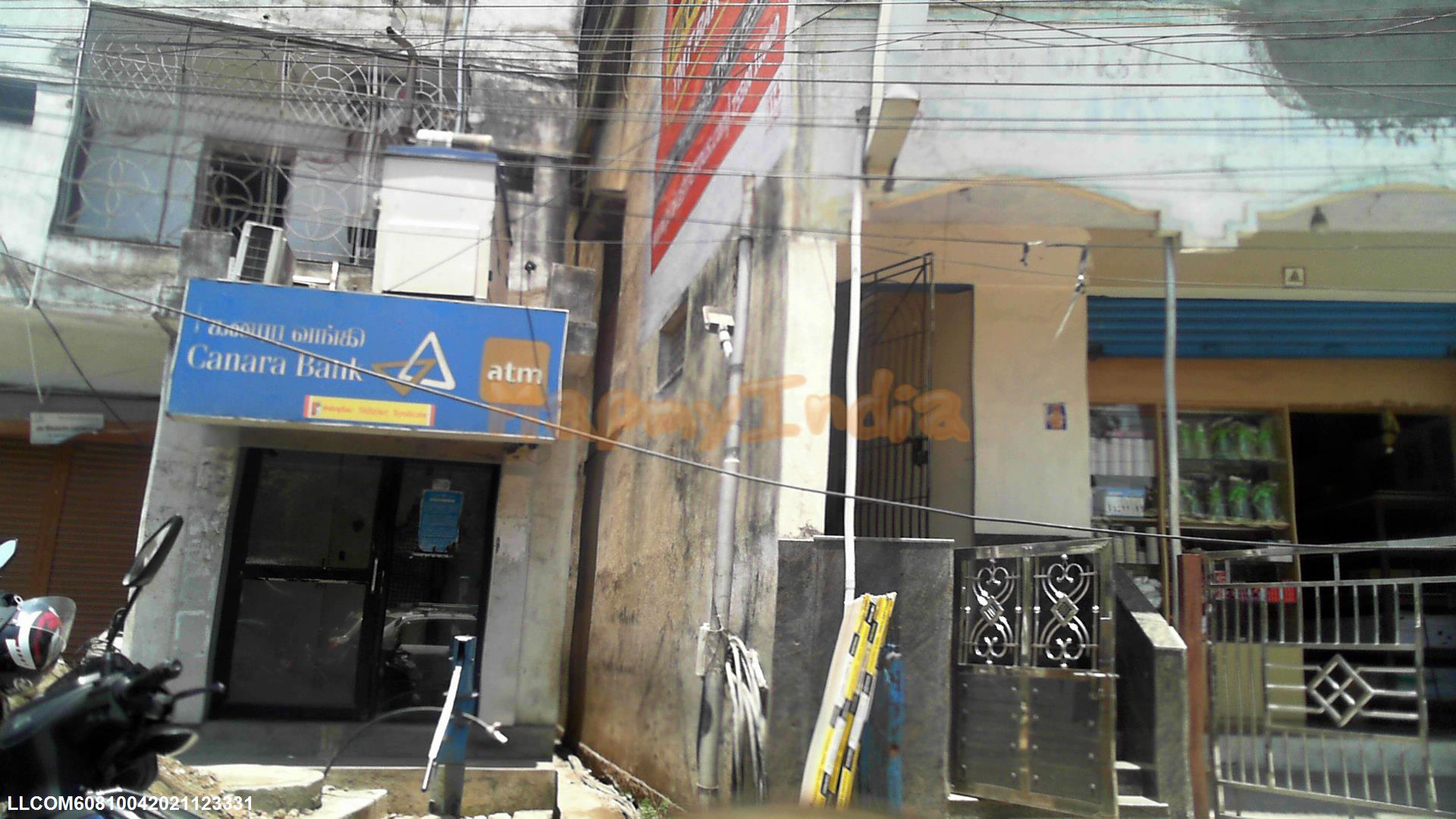

Supplement: Supplementary file 2 — Supplementary Material 2 [file 41598_2026_40742_MOESM2_ESM.zip › sample_data_yolov5/LCOM60810042021123331.jpg]

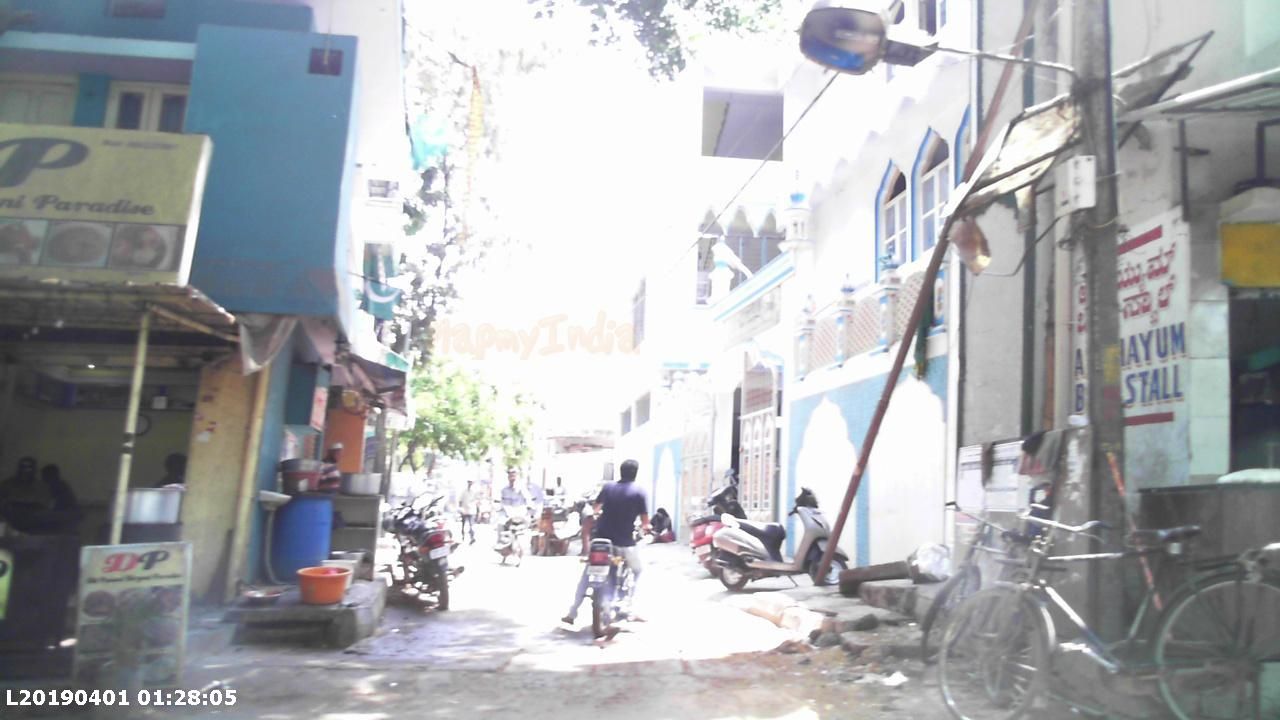

Supplement: Supplementary file 2 — Supplementary Material 2 [file 41598_2026_40742_MOESM2_ESM.zip › sample_data_yolov5/04-01_01.28.05.jpg]

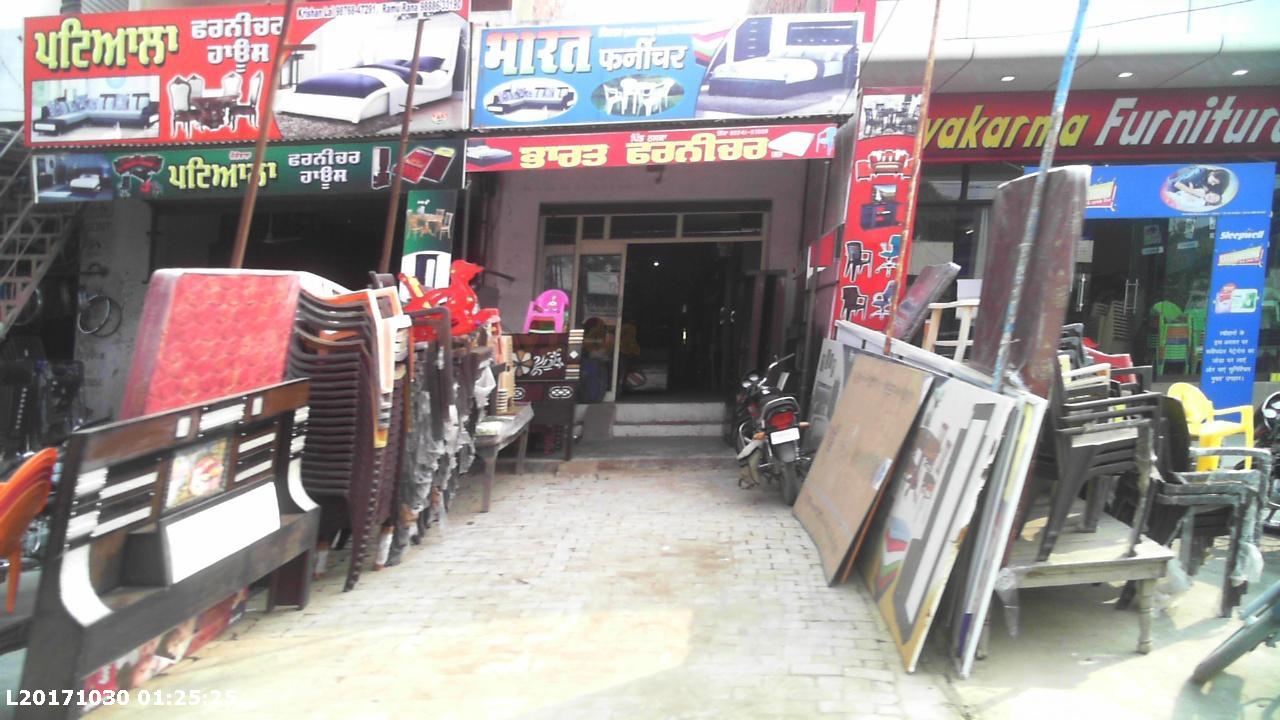

Supplement: Supplementary file 2 — Supplementary Material 2 [file 41598_2026_40742_MOESM2_ESM.zip › sample_data_yolov5/10-30 01.25.25.jpg]

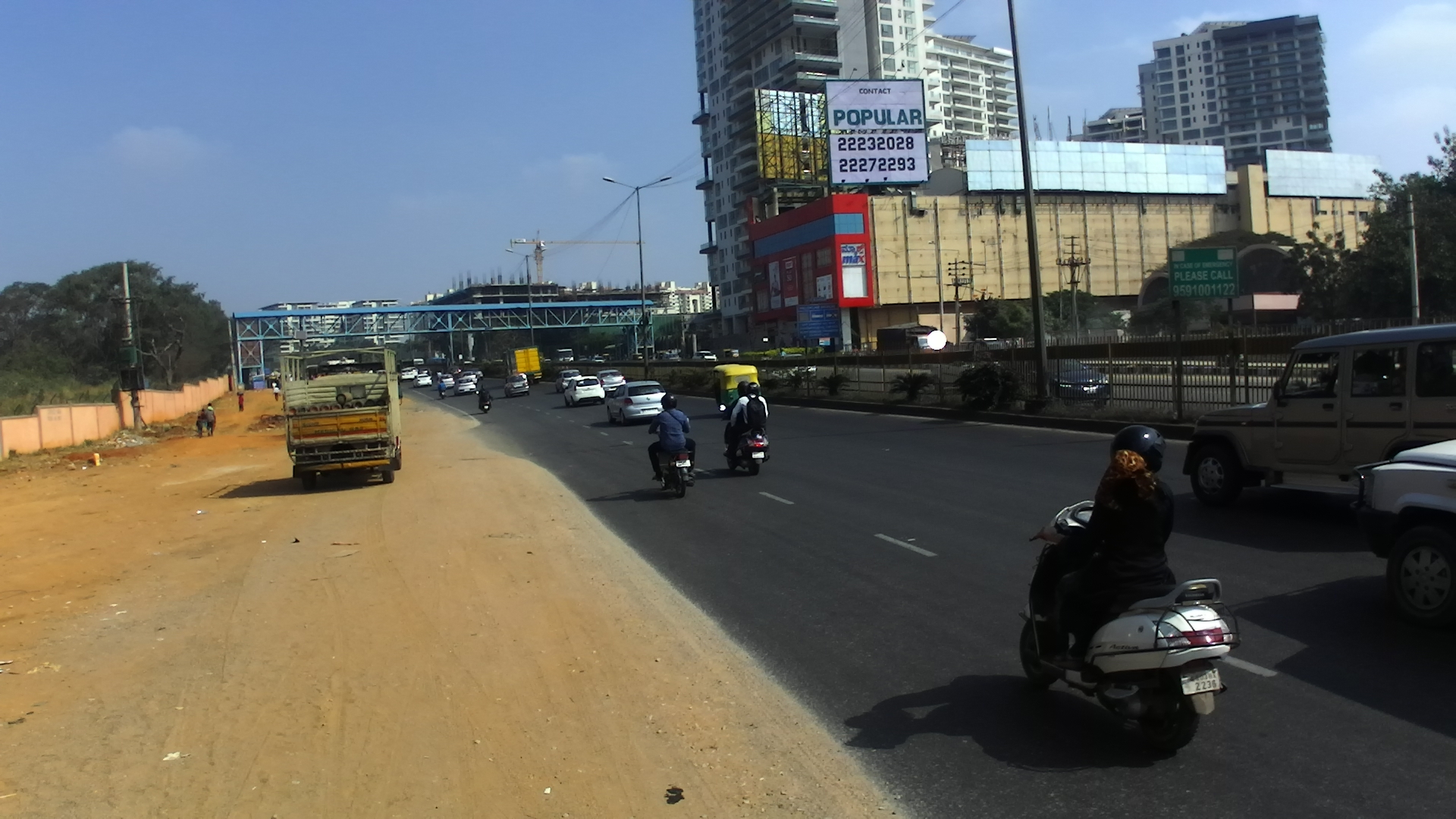

Supplement: Supplementary file 2 — Supplementary Material 2 [file 41598_2026_40742_MOESM2_ESM.zip › sample_data_yolov5/311218_105400_16717_zed_l_099.jpg]

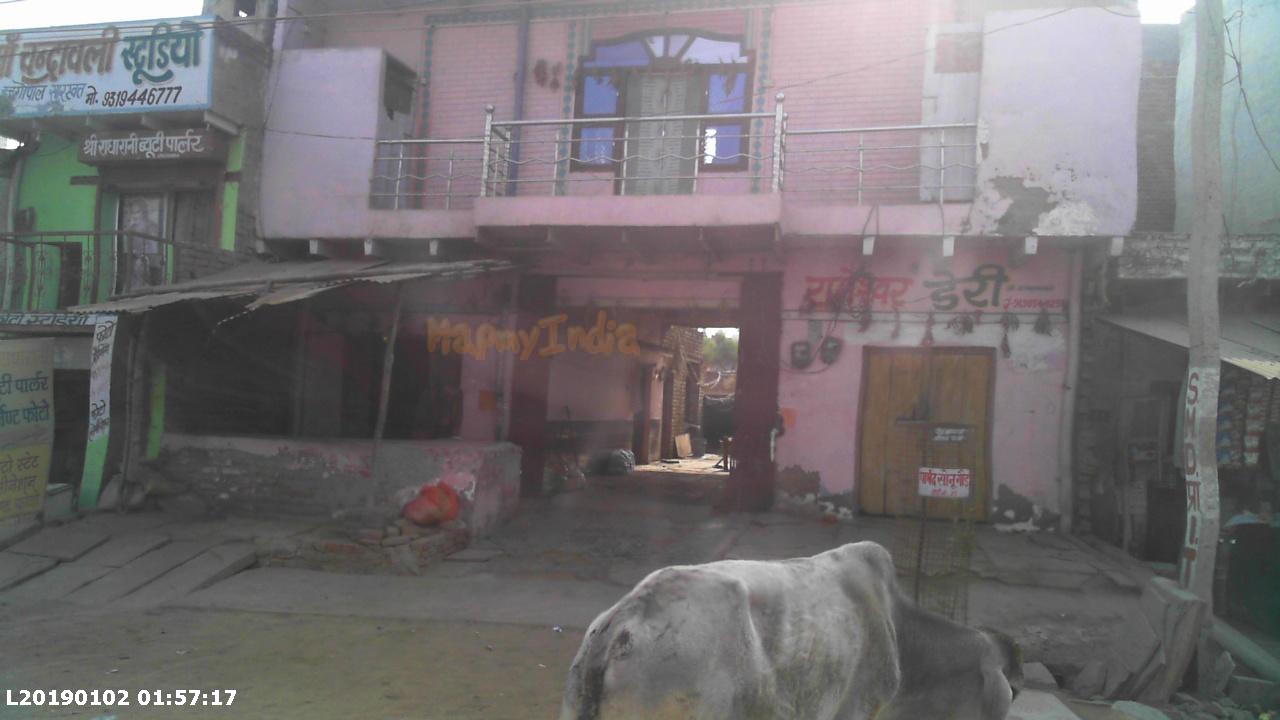

Supplement: Supplementary file 2 — Supplementary Material 2 [file 41598_2026_40742_MOESM2_ESM.zip › sample_data_yolov5/L_01-02_01.57.17.jpg]

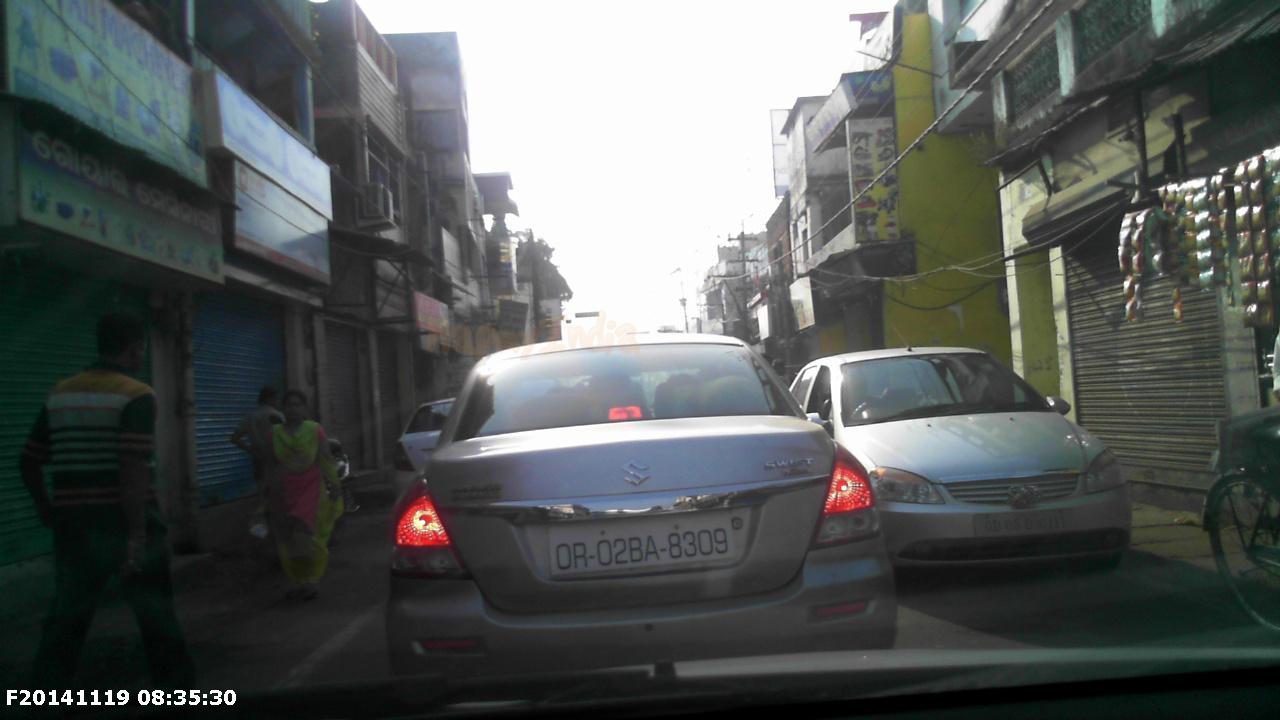

Supplement: Supplementary file 2 — Supplementary Material 2 [file 41598_2026_40742_MOESM2_ESM.zip › sample_data_yolov5/F_11-19_08.35.30.jpg]

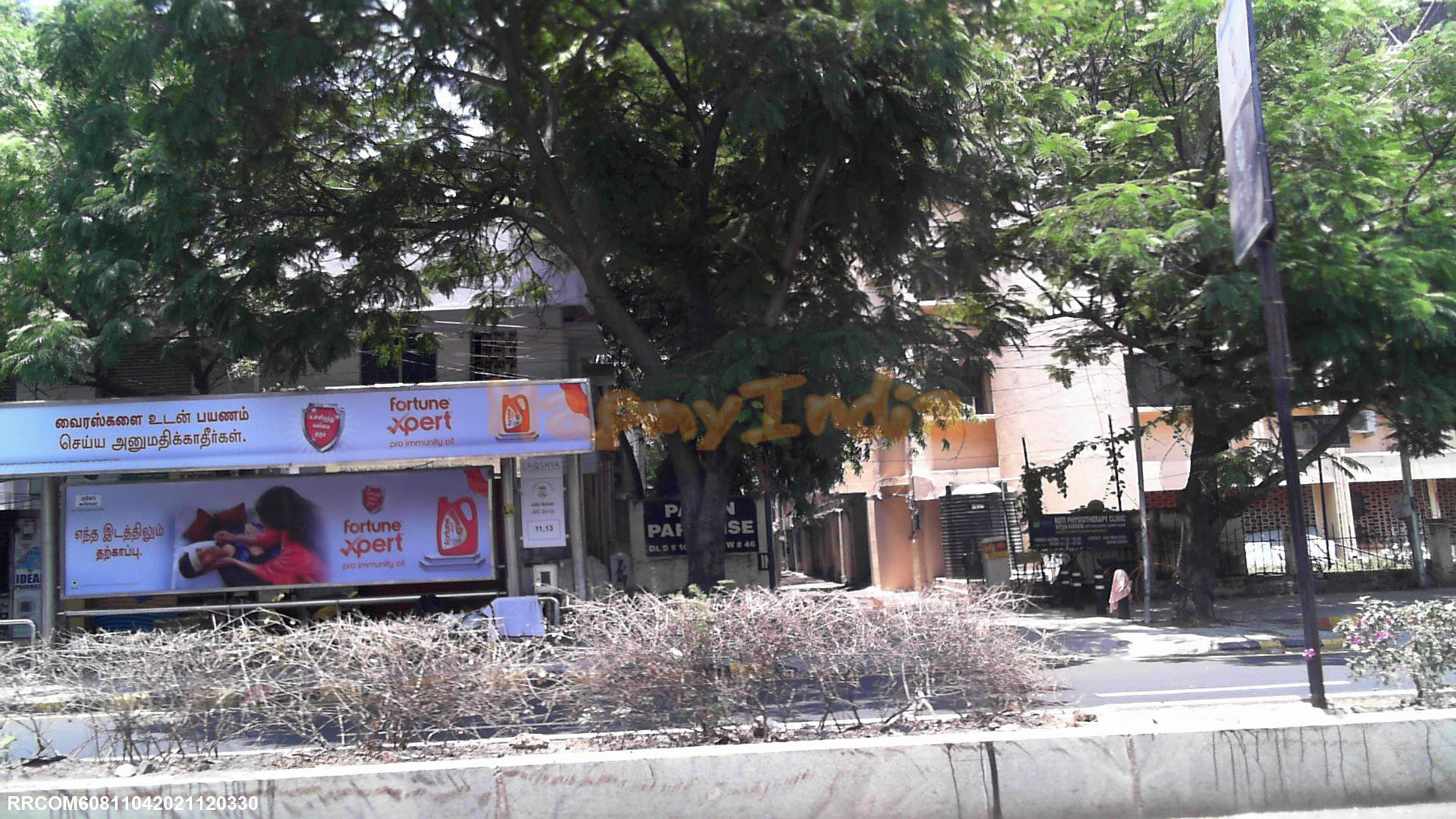

Supplement: Supplementary file 2 — Supplementary Material 2 [file 41598_2026_40742_MOESM2_ESM.zip › sample_data_yolov5/RCOM60811042021120330.jpg]

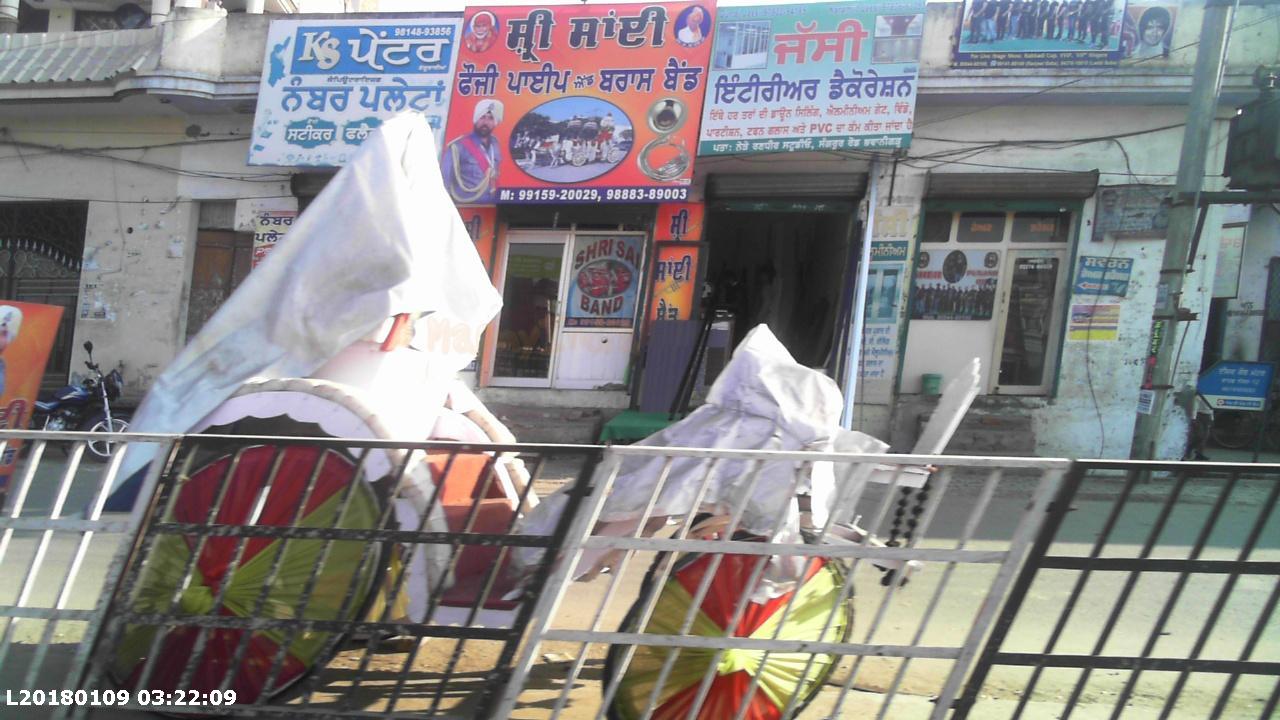

Supplement: Supplementary file 2 — Supplementary Material 2 [file 41598_2026_40742_MOESM2_ESM.zip › sample_data_yolov5/01-09 03.22.09.jpg]

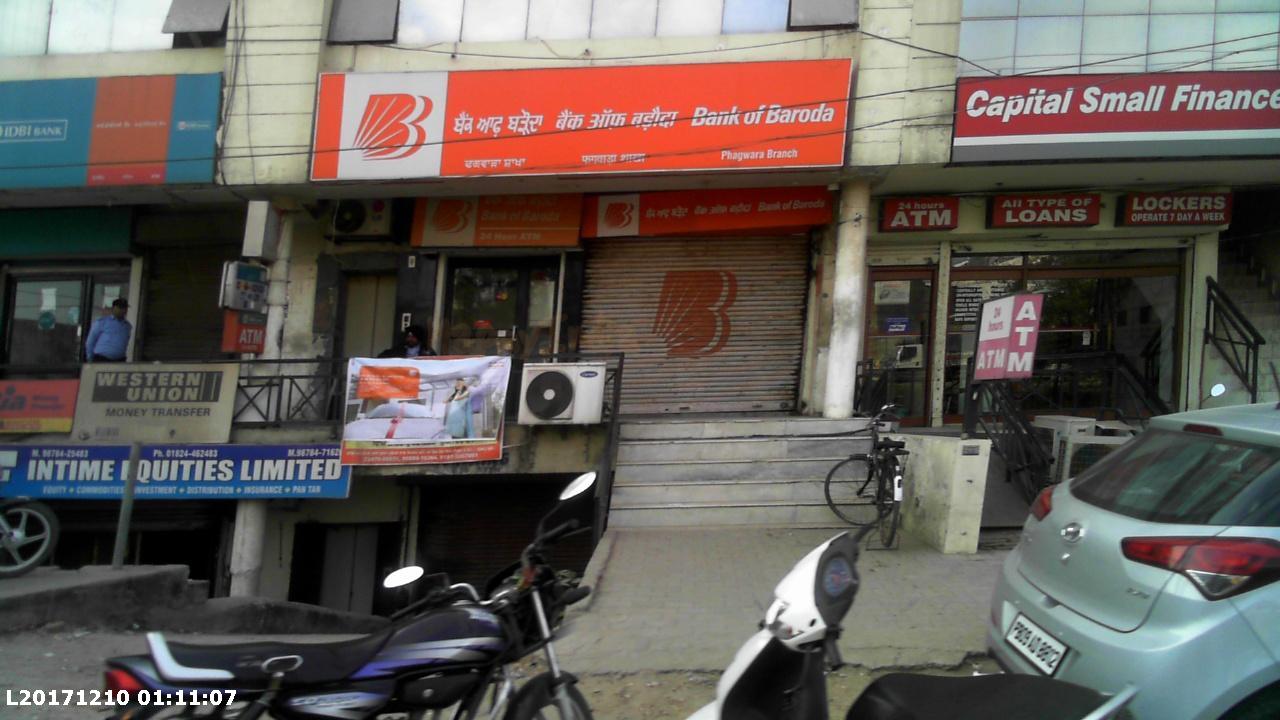

Supplement: Supplementary file 2 — Supplementary Material 2 [file 41598_2026_40742_MOESM2_ESM.zip › sample_data_yolov5/12-10 01.11.07.jpg]

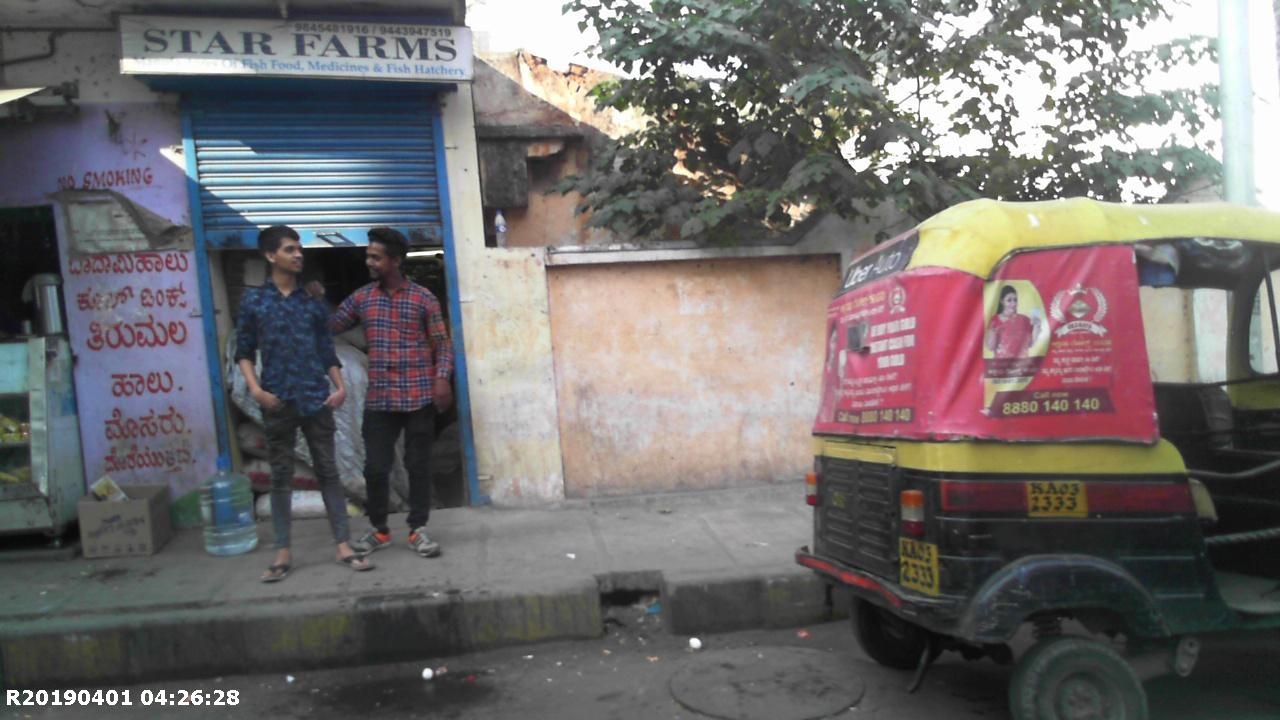

Supplement: Supplementary file 2 — Supplementary Material 2 [file 41598_2026_40742_MOESM2_ESM.zip › sample_data_yolov5/04-01_04.26.28.jpg]

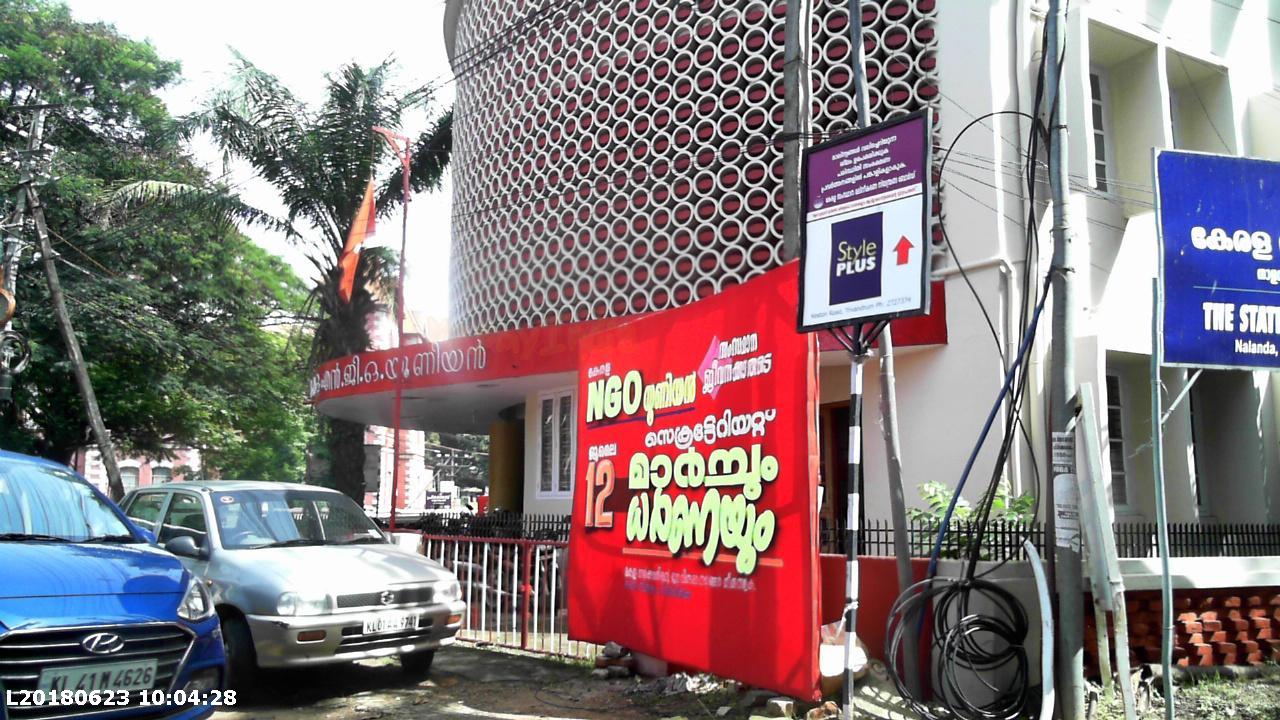

Supplement: Supplementary file 2 — Supplementary Material 2 [file 41598_2026_40742_MOESM2_ESM.zip › sample_data_yolov5/L_06-23_10.04.28.jpg]

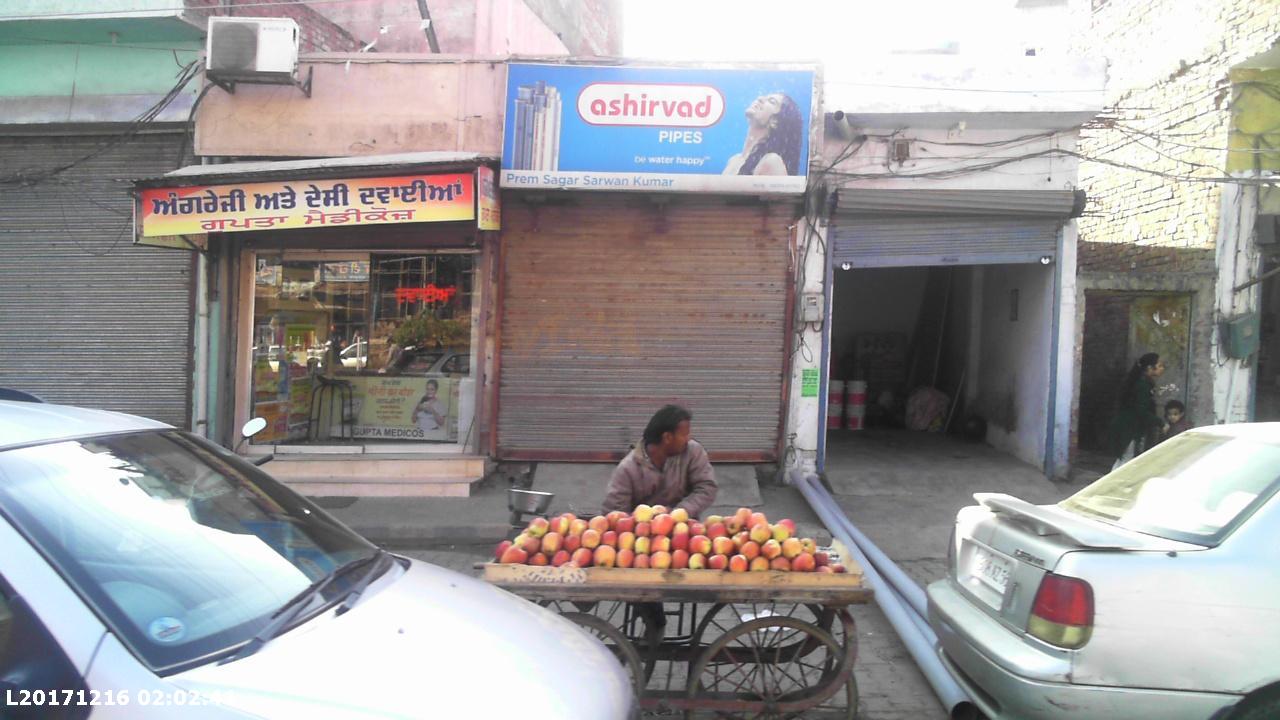

Supplement: Supplementary file 2 — Supplementary Material 2 [file 41598_2026_40742_MOESM2_ESM.zip › sample_data_yolov5/12-16 02.02.41.jpg]

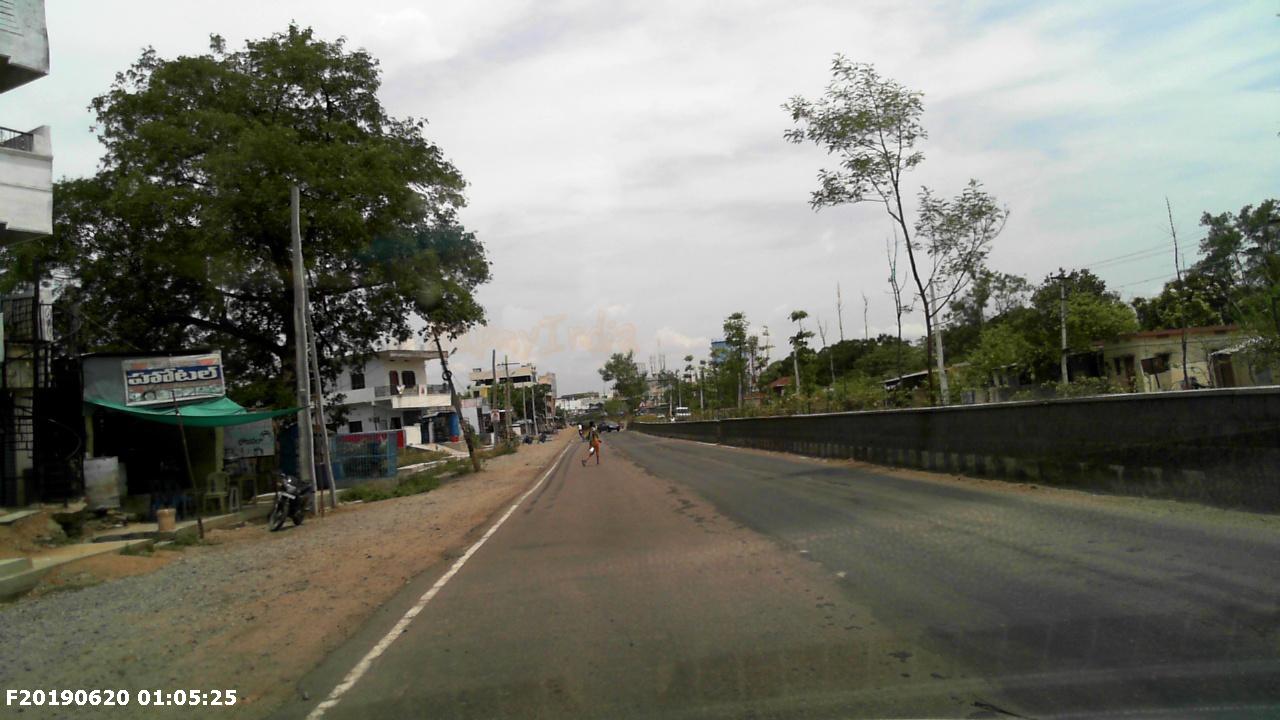

Supplement: Supplementary file 2 — Supplementary Material 2 [file 41598_2026_40742_MOESM2_ESM.zip › sample_data_yolov5/06-20 01.05.25.jpg]

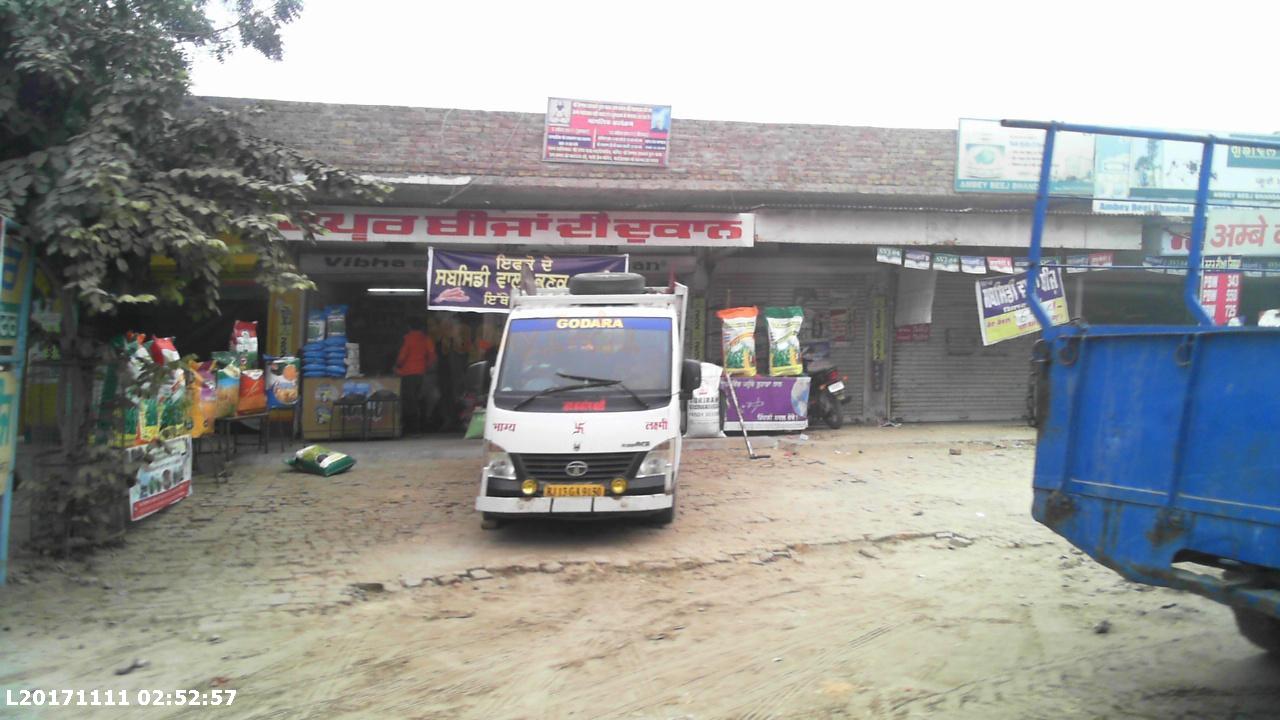

Supplement: Supplementary file 2 — Supplementary Material 2 [file 41598_2026_40742_MOESM2_ESM.zip › sample_data_yolov5/11-11 02.52.57.jpg]

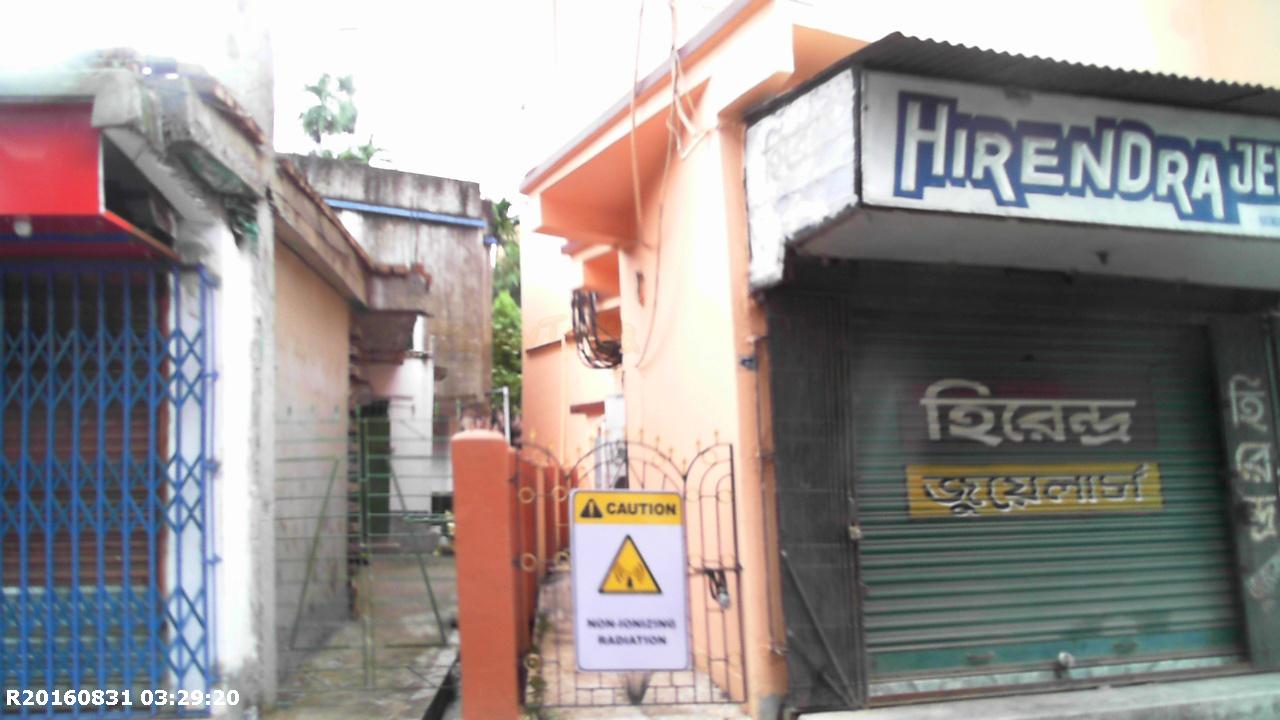

Supplement: Supplementary file 2 — Supplementary Material 2 [file 41598_2026_40742_MOESM2_ESM.zip › sample_data_yolov5/R_08-31_03.29.20.jpg]

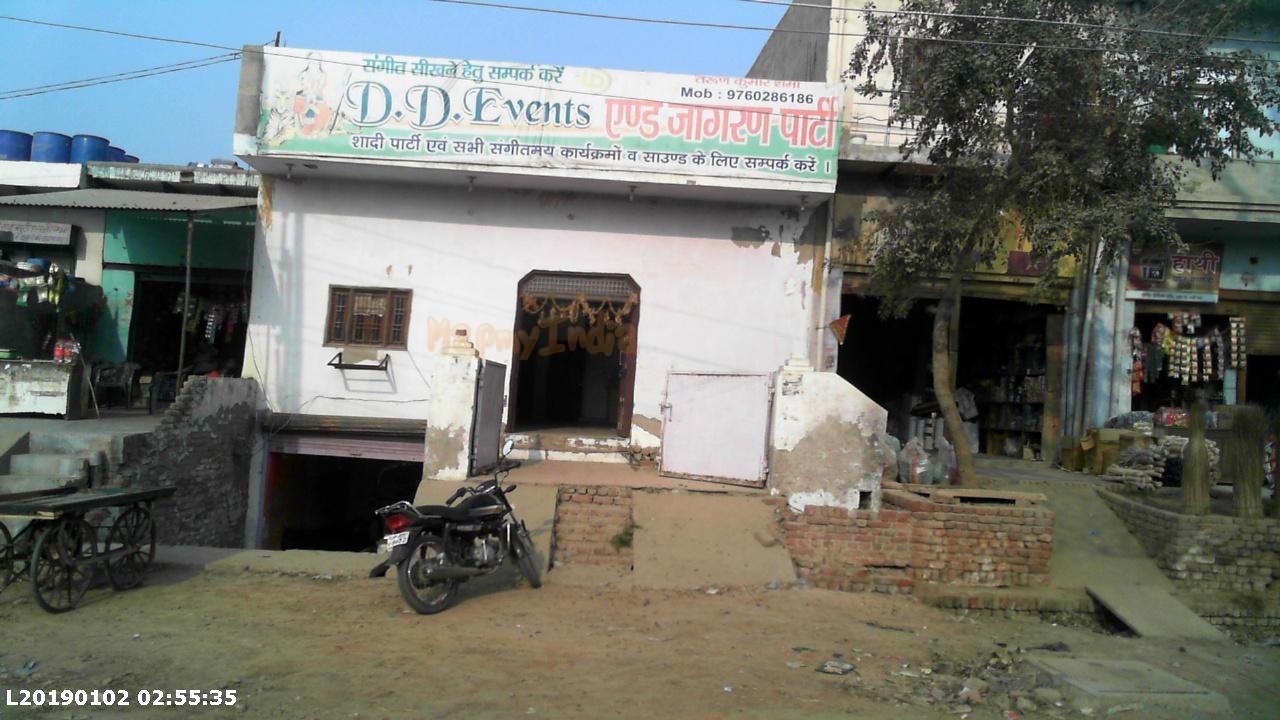

Supplement: Supplementary file 2 — Supplementary Material 2 [file 41598_2026_40742_MOESM2_ESM.zip › sample_data_yolov5/L_01-02_02.55.35.jpg]

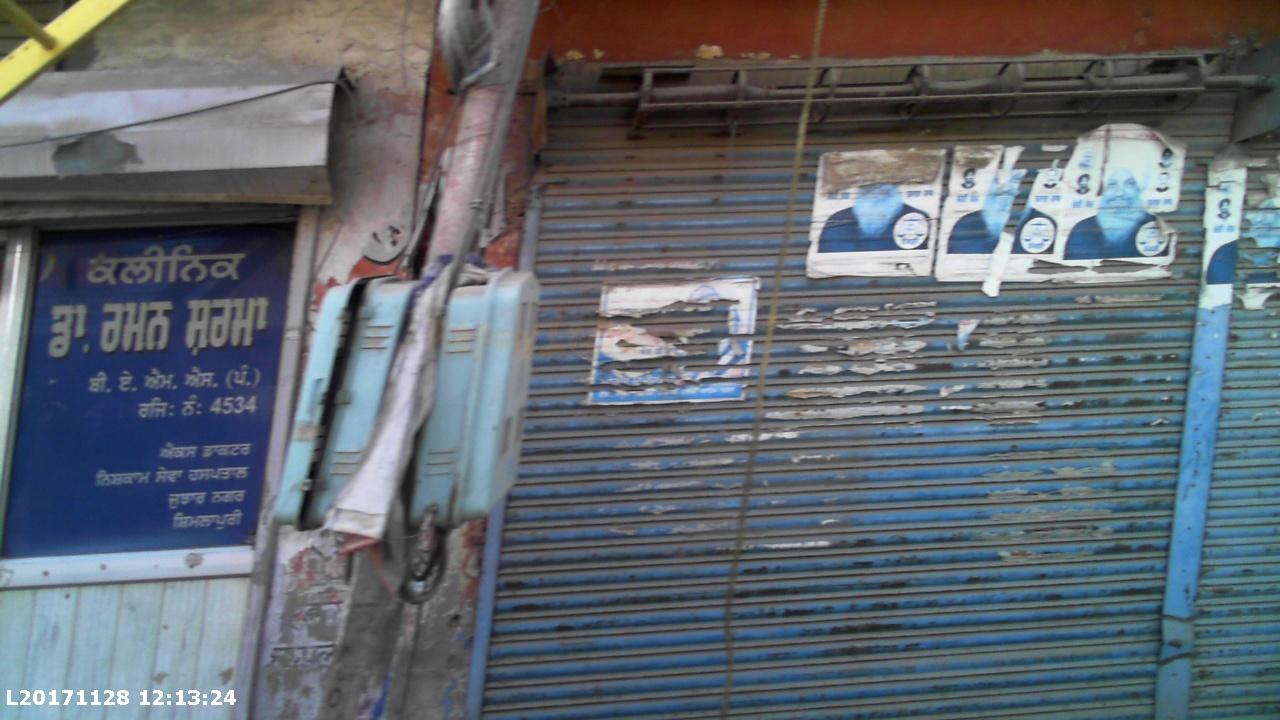

Supplement: Supplementary file 2 — Supplementary Material 2 [file 41598_2026_40742_MOESM2_ESM.zip › sample_data_yolov5/11-28 12.13.24.jpg]

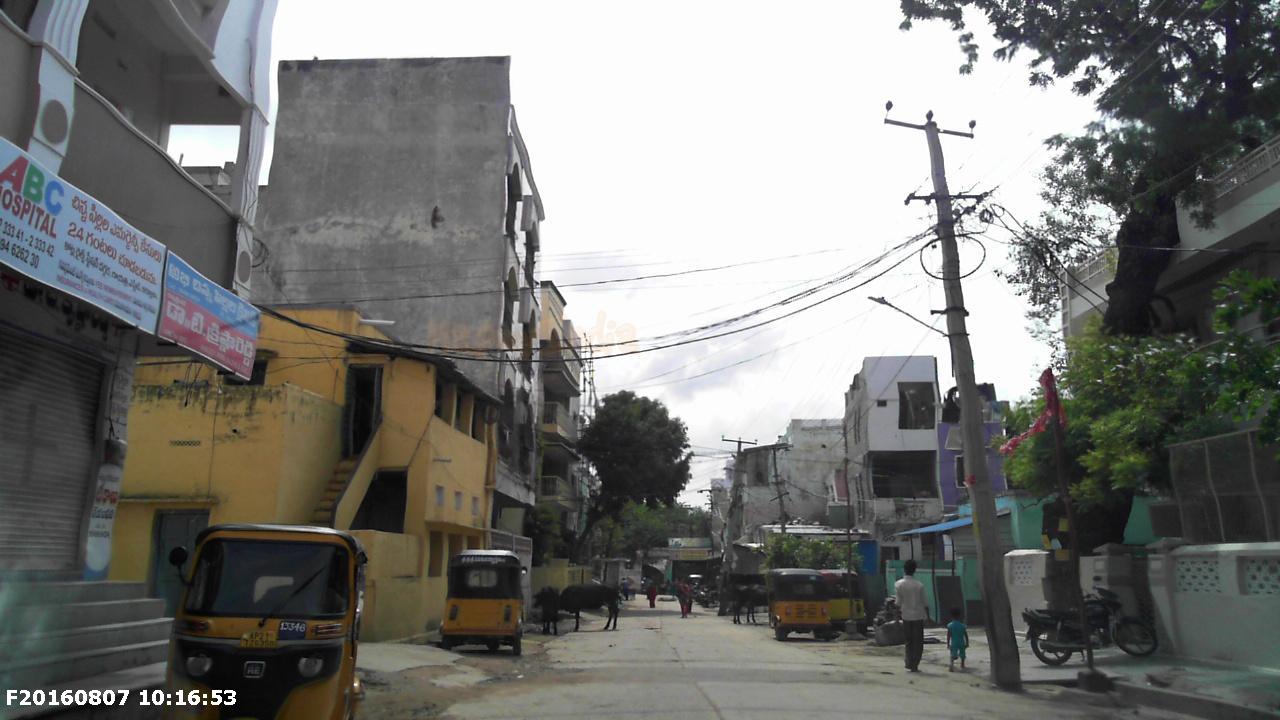

Supplement: Supplementary file 2 — Supplementary Material 2 [file 41598_2026_40742_MOESM2_ESM.zip › sample_data_yolov5/08-07 10.16.53.jpg]

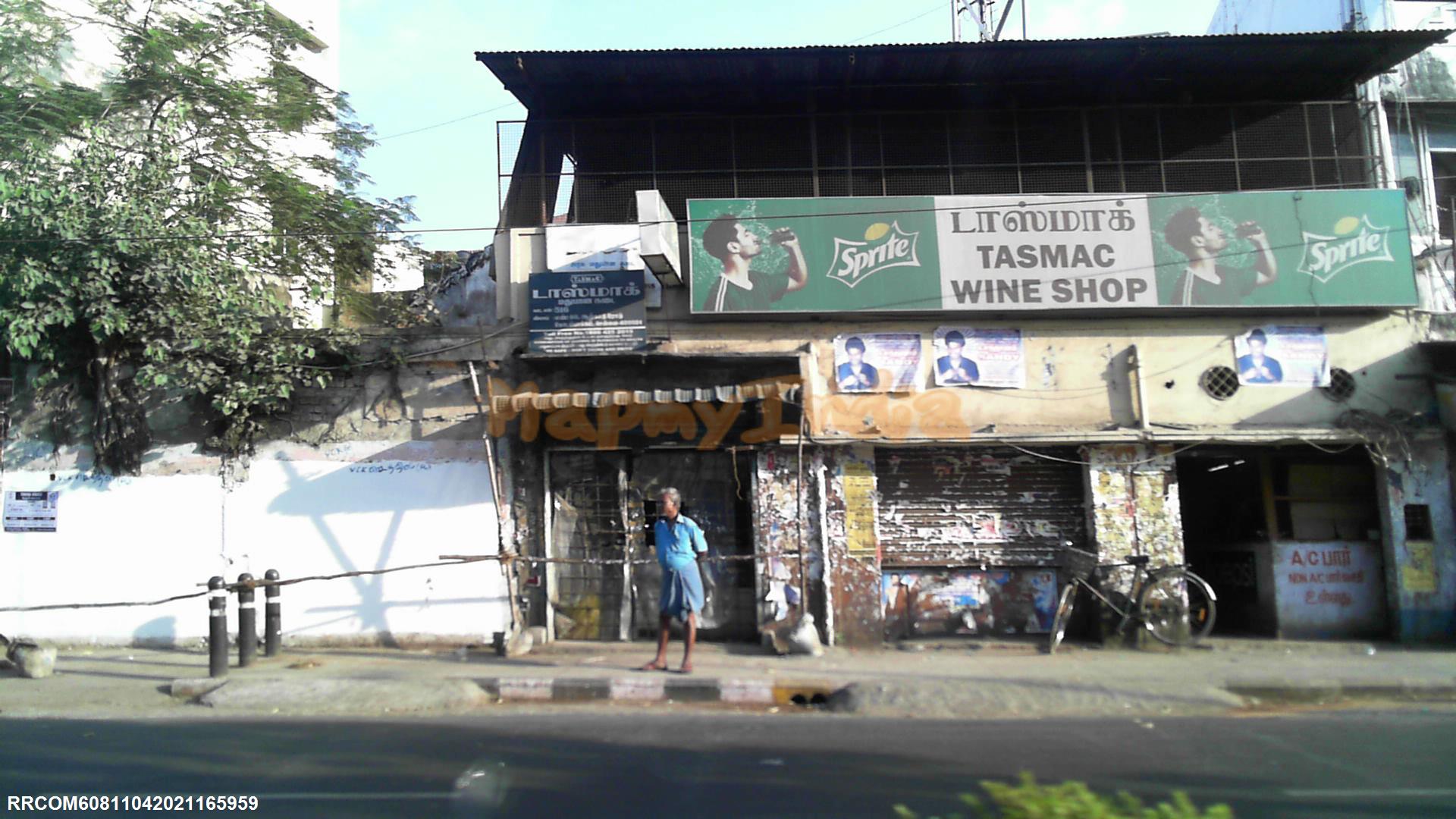

Supplement: Supplementary file 2 — Supplementary Material 2 [file 41598_2026_40742_MOESM2_ESM.zip › sample_data_yolov5/RCOM60811042021165959.jpg]
